# Review of the Eustrophinae (Coleoptera, Tetratomidae) of America north of Mexico

**DOI:** 10.3897/zookeys.188.2976

**Published:** 2012-05-02

**Authors:** Darren A. Pollock

**Affiliations:** 1 Department of Biology, Eastern New Mexico University, Portales NM USA

**Keywords:** Tetratomidae, new species, Arizona, natural history, Nearctic, faunal review

## Abstract

The Nearctic fauna (north of Mexico) of Eustrophinae is reviewed, and consists of the following five genera and 12 species: *Pseudoholostrophus (Pseudoholostrophus) impressicollis* (LeConte), *Pseudoholostrophus (Holostrophinus) discolor* (Horn), *Holostrophus bifasciatus* (Say), *Eustrophus tomentosus* Say, *Eustrophopsis confinis* (LeConte), *Eustrophopsis bicolor* (Fabricius), *Eustrophopsis brunneimarginatus* (Dury), *Eustrophopsis indistinctus* (LeConte), *Eustrophopsis arizonensis* (Horn), *Eustrophopsis ornatus* (Van Dyke), *Eustrophopsis crowdyi* **sp. n.**, and *Synstrophus repandus* (Horn). A lectotype is designated for *Eustrophus brunneimarginatus* Dury. A key is given to separate genera and species, supplemented with illustrations of relevant features, including aedeagi of all Nearctic species of *Eustrophopsis*. Detailed distribution (including Mexican records) and natural history data are provided.

## Introduction

The Eustrophinae, like many groups within Tenebrionoidea, have not had a stable family-level placement until fairly recently. Various authors have commented on the taxonomic history of the group (e.g. Lawrence 1991; Young and Pollock 2002; Pollock 2008; Lawrence and Leschen 2010); however, the recent placement of eustrophines within an enlarged Tetratomidae (as proposed by Nikitsky 1998), is based in large part on characteristics of larvae. Very few comprehensive phylogenetic analyses for Tenebrionoidea have been completed; the study by Lawrence et al. (2011), based on morphological characters of adults and larvae of the entire Coleoptera, used three exemplar taxa from Tetratomidae, including *Eustrophopsis bicolor* (Fabricius). Monophyly of Tetratomidae was not supported in this analysis; in fact, *Eustrophopsis* was shown to be the sister taxon to *Orchesia* (Melandryidae). *Tetratoma* (Tetratomidae: Tetratominae) and *Penthe* (Tetratomidae: Penthinae) were part of a monophyletic group that also contained *Dipsaconia* (Ulodidae) and *Chalcodrya* (Chalcodryidae). An earlier study by Beutel and Friedrich (2005) hypothesized Tetratomidae (data taken from the literature) as part of a very large, unresolved branch of basal Tenebrionoidea.

There have been no large-scale studies of the Eustrophinae specifically, although Nikitsky (1998) provided an overview of the classification of the entire family Tetratomidae. Also, Nikitsky (2007) revised the genus *Holostrophus*, representing the only published work on a single genus of Eustrophinae. Pollock (2008) reviewed the Canadian eustrophine fauna, as part of a volume covering biodiversity, systematics, and ecology of Canadian Coleoptera. The present study treats all taxa found in North America, north of Mexico, including the description of a new species from southern Arizona. Much of the descriptive, ecological and distributional data for species occurring in Canada, treated herein, were given previously by Pollock (2008). As in Pollock (2008), the present study is not meant to be a detailed revision; rather, it is a review of a specific biogeographic group of Eustrophinae. Therefore, descriptions are rather diagnostic in nature, except for the description of *Eustrophopsis crowdyi*, new species, which is more detailed and complete.

Nikitsky (1998) provided a key to the five recognized subfamilies of Tetratomidae. The Eustrophinae have the following characteristics: body elongate oval, often more distinctly narrowed posteriorly; all legs with simple, narrow tarsi; antennomeres 3-7 more or less broadened, at least some antennomeres transverse; pronotum with two sublinear impressions basally, metepisterna distinctly subdivided into two sections; metendosternite without laminae.

The world fauna of Eustrophinae comprises 5 genera and 86 species (compiled from various sources), distributed worldwide except for Australia and New Zealand. The greatest diversity occurs in tropical areas, especially in the Neotropical region. Members of the subfamily are relatively homogeneous structurally, and both larvae and adults are associated with various groups of fungi on dead trees (Lawrence 1991; Pollock 2008). No studies are available that detail the ecological habits of any specific taxon of Eustrophinae. Most information available is taken from collection records. Lawrence (1991: 506) gave the following fungal associates with some genera and species of Eustrophinae: *Eustrophopsis* spp. (*Polyporus squamosus*, *Tyromyces albellus*, *Dichomitus squalens*, *Laetiporus sulphureus*, *Bjerkandera adusta*, *Inonotus munzii*, *Inonotus vulpinus*, *Phellinus gilvus*, *Pleurotus sapidus*, *Panus rudis*, *Lentinus lepideus*); *Synstrophus repandus* (Horn) (*Pleurotus ostreatus*, *Phaeolus schweinitzii*, and *Coriolus versicolor*); *Eustrophus tomentosus* Say (*Polyporus squamosus*). Individual fungal/collection records are given below for each species (from label data).

Adults of Eustrophinae are active at night, when they emerge from daytime hiding places and occupy exposed surfaces, on dead wood or on associated fungal bodies. Very long series of specimens are somewhat rare; collecting during the day will yield few specimens, whereas night collecting or mass-trapping can be very productive and effective methods for obtaining long series. During the day, potentially good, fungus-infested dead trees can be marked (for example, by using thumbtacks with reflective paint) and returned to at night, to collect the active adults.

Adults of *Eustrophopsis* are seemingly attracted to Lindgren funnel traps, and/or the scolytine baits often deployed along with these devices. For example, a single locality (Arizona, Cochise Co., Chiricahua Mts., Turkey Creek, 2000 m, 31°51.280'N, 109°19.883'W) yielded the following specimens over a two-week period: 7.vii.2002 (*Eustrophopsis arizonensis*: 54♂♂, 104♀♀; *Eustrophopsis ornatus*: 7♂♂; *Eustrophopsis crowdyi*, sp. n.: 10♂♂, 11♀♀); 14.vii.2002 (*Eustrophopsis arizonensis*, 45♂♂, 92♀♀; *Eustrophopsis ornatus*: 7♂♂, 17♀♀; *Eustrophopsis crowdyi*, sp. n.: 16♂♂, 11♀♀).

As in the majority of Tenebrionoidea, immature stages of Eustrophinae are rather imperfectly known. Larvae are found in the same habitats as adults; however, whereas adults are often found on surfaces at night, larvae remain within the rotting wood/fungal substrate (Pollock pers. observ.). Nikitsky (1998) provided a key to subfamilies of tetratomid larvae; however, only *Eustrophopsis* and *Holostrophus* were included. A key to larvae of *Holostrophus* (three Oriental species only) was also given by Nikitsky (1998). The most detailed description of a larva of *Eustrophopsis* is that of Węgrzynowicz (1995) who described the larva of *Eustrophopsis quindecimmaculata* (Laporte), a Neotropical species.

Among Nearctic eustrophines, the only species reasonably well known in the larval stage is *Eustrophopsis bicolor* (Fabricius); this is the most common species, so perhaps this is not surprising. Weiss (1919) provided a brief description of the larva, along with some reasonably detailed information on host preference and natural history. Böving and Craighead (1931) treated the larva of *Eustrophopsis bicolor* also, but only illustrated the posterior end of the abdomen (based on larval characters, they removed *Eustrophus* from Melandryidae and transferred it to Dacnidae). Lawrence (1991) illustrated the dorsal habitus of the larva, along with an SEM of the larval mandible. Lawrence and Leschen (2010) provided a comprehensive description of the larvae of Tetratomidae, with various details given for Eustrophinae (based on *Eustrophopsis* and *Holostrophus*).

## Materials and methods

This study is based on 6,600 adult specimens examined and/or borrowed from the following private and public collections and museums:

**AMNH** American Museum of Natural History, New York, NY

**BBC** Brian Baldwin Collection (private)

**BYUC** Monte L. Bean Life Science Museum, Brigham Young University, Provo, UT

**CARR** J.B. and A. Carr Collection (now part of CNC)

**CAS** California Academy of Sciences, San Francisco, CA

**CBC** Cheryl Barr Collection (private)

**CGMC** Chris Majka Collection (private)

**CMNC** Canadian Museum of Nature, Gatineau, QC

**CMNH** Carnegie Museum of Natural History, Pittsburgh, PA

**CNC** Canadian National Collection of Insects, Ottawa, ON

**CNHM** Cincinnati Natural History Museum, Cincinnati, OH

**CUIC** Cornell University, Ithaca, NY

**DAPC** Darren A. Pollock Collection (private)

**DSSC** Derek Sikes Collection (private)

**EGRC** Edward G. Riley Collection (private)

**EIUC** Eastern Illinois University Collection, Charleston, IL

**FMNH** Field Museum of Natural History, Chicago, IL

**FSCA** Florida State Collection of Arthropods, Gainesville, FL

**INHS** Illinois Natural History Survey, Champaign, IL

**JBWM** J.B. Wallis Museum of Entomology, University of Manitoba, Winnipeg, MB

**LACM** Los Angeles County Museum, Los Angeles, CA

**LSU** Louisiana State Arthropod Museum, Louisiana State University, Baton Rouge, LA

**MCZ** Museum of Comparative Zoology, Harvard University, Cambridge, MA

**MKOC** M.K. Oliver Collection (private)

**MSUC** Michigan State University, East Lansing, MI

**MTEC** Montana State University, Bozeman, MT

**MUNC** Memorial University of Newfoundland, St. John’s, NF

**NDSU** North Dakota State University, Fargo, ND

**NSMC** Nova Scotia Museum Collection, Halifax, NS

**ODAC** Oregon Department of Agriculture, Salem, OR

**OSUC** Ohio State University, Columbus, OH

**PFC** Pacific Forestry Centre, Victoria, BC

**PKLC** Paul K. Lago Collection (private)

**PPCD** West Virginia Department of Agriculture, Charlestown, WV

**RBCM** Royal British Columbia Museum, Vancouver, BC

**RLC** Richard Leschen Collection (private)

**ROME** Royal Ontario Museum, Toronto, ON

**RTC** Robert Turnbow Collection (private)

**SLC** Serge LaPlante Collection (private)

**SMC** Scott McCleve Collection (private) [now in UAIC]

**TAMU** Texas A&M University, College Station, TX

**UAIC** University of Arizona, Tucson, AZ

**UBC** University of British Columbia, Vancouver, BC

**UCBC** Essig Museum of Entomology, Berkeley, CA

**UCFC** University of Central Florida, Orlando, FL

**UCMS** University of Connecticut, Storrs, CT

**UCRC** University of California, Riverside, CA

**UGAC** University of Georgia, Athens, GA

**UMIC** University of Mississippi, Oxford, MS

**UMMZ** University of Michigan, Ann Arbor, MI

**UMRM** W.R. Enns Entomology Museum, Columbia, MO

**USMN** University of Nebraska State Museum, Lincoln, NE

**USNM** National Museum of Natural History, Smithsonian Institution, Wa- shington, DC

**WFBM** W.F. Barr Entomological Collection, Moscow, ID

**WIRC** University of Wisconsin Insect Research Center, Madison, WI

**ZHFC** Zack H. Falin Collection (private)

Standard methods of examination and description of specimens were used in this study. Descriptions are somewhat diagnostic in nature, hopefully facilitating rapid identification of species. Details on the male genitalia, including illustrations, are presented for *Eustrophopsis* only, because 1) this genus is larger than the other genera of Eustrophinae and species are more difficult to distinguish; and 2) genital structures have already been described and/or illustrated for other taxa within Eustrophinae (e.g. Nikitsky 1998, 2007).

Annotations relating to natural history, as derived from specimen label data and published accounts, are given under each species. Locality data are summarized for each species using country, state (province) and county names (where available); complete data for non-type specimens are given in Appendix 1. In the descriptions, TL = length from anterior pronotal margin to apex of elytra (along midline); GEW = maximum width, across elytra. Type specimen label data are given verbatim, enclosed within quotation marks; individual labels are separated by a slash (/).

Digital images were taken with a Nikon Coolpix 5000^®^ digital camera fitted to a Leica MZ95 stereoscope. The 10–30 separate images taken for each specimen/structure (focus adjusted manually) were imported into Combine ZP (Hadley 2010), which stacked and aligned the individual images, creating a final digital image. The images of the *Eustrophopsis* genitalia (Figures 56–64) were not stacked; each represents a single image only.

The range maps are presented in two different formats: collection records for Canadian and Mexican specimens are presented as specific “dot localities” on maps. All United States records are depicted as county records, only.

## Systematics

### Key to genera and species of Nearctic Eustrophinae

**Table:** 

1	Outer faces of meso- and metatibiae smooth, without oblique ridges (Figures 53, 55)	2
–	Outer faces of meso- and metatibiae with numerous oblique, comb-like ridges (e.g. Fig. 54)	(Eustrophini), 5
2	Prosternal process narrowed distally, not extended posterad of procoxae (Fig. 49); elytral punctation coarse, forming distinct striae; eyes narrowly separated (Fig. 37)	*Synstrophus repandus* (Horn)
–	Prosternal process widened distally, separating procoxae (Figures 38–40); elytral punctation fine, not forming distinct striae; eyes widely separated (Figures 26–28)	(Holostrophini), 3
3	Eyes distinctly emarginate, distance between eyes less than transverse ocular diameter; elytra dark in color, with 4 lighter colored, subquadrate maculae (Fig. 3)	*Holostrophus bifasciatus* (Say)
–	Eyes only indistinctly emarginate, distance between eyes greater than transverse ocular diameter; elytra uniformly rufous to piceous, without distinctly lighter markings (Figures 1–2)	4
4	Dorsal surface finely punctate, setae short and apressed against surface; impressions along posterior pronotal margin linear, parallel, distinctly impressed (Fig. 1); anterior pronotal margin not elevated above level of head (Fig. 14); known from western North America (BC, WA, OR, CA)	*Pseudoholostrophus impressicollis* (LeConte)
–	Dorsal surface more coarsely punctate, setae more distinct and at least partially erect; impressions along posterior pronotal margin indistinct; anterior margin of pronotum elevated above level of head (Fig. 15); known from eastern North America (IN, MA, MD, NH, PA, QC, RI, VA, TN)	*Pseudoholostrophus discolor* (Horn)
5	Prothoracic episterna without transverse suture (Fig. 50); male without profemoral sensillar patch; annular antennal sensilla interrupted, not present around entire circumference of antennomeres	*Eustrophus tomentosus* Say
–	Prothoracic episterna each with distinct, transverse suture (Fig. 51); male with small sensillar patch on ventral edge of profemur; annular antennal sensilla present around entire circumference of antennomeres	*Eustrophopsis*, 6
6	Medial margins of eyes widely separated, distance between eyes equal to or greater than length of antennomere 1 (Fig. 30); body broadly ovate, slightly tapered posteriorly (Fig. 5)	*Eustrophopsis confinis* (LeConte)
–	Medial margins of eyes more narrowly separated, in some specimens virtually contiguous; if moderately widely separated, distance between medial margins less than length of antennomere 1; body more elongate oval, noticeably tapered posteriorly	7
7	Pronotum and elytra dark, piceous to near black, with distinctly contrasting lighter, rufous lateral margins (Figures 7, 20)	*Eustrophopsis brunneimarginatus* (Dury)
–	Pronotum and elytra uniform in color, from rufous to black; some specimens with indistinct to distinct lighter colored markings on elytral disc, but not restricted only to lateral margins	8
8	Prothoracic episterna with distinctly rugulose macrosculpturing, somewhat obscuring setae and punctation (Fig. 52)	*Eustrophopsis arizonensis* (Horn)
–	Prothoracic episterna smooth, without rugulose macrosculpturing, punctation and setae not obscured (e.g. Fig. 51)	9
9	Antennomeres 5–10 distinctly broad, wider than long (Figures 35–36); antennomeres 5–11 of males flattened on one side, with distinct, fine sensillar setae; body size larger (TL 5.3–7.9 mm); known only from Arizona, New Mexico and Mexico	10
–	Antennomeres 5–10 longer than wide; antennomeres 5–11 of males not flattened, without obvious sensillar setae; body size smaller (TL 4.2–6.5 mm); widespread in distribution, including Arizona, New Mexico and Mexico	11
10	Body, including venter, uniformly dark piceous to black, without distinctly contrasting lighter colored antennomeres 1–4 and 11 (Fig. 36); prosternal process relatively wide, broadly rounded distally (Fig. 48); eyes separated by approximately maximum width of antennomere 1	*Eustrophopsis crowdyi*, sp. n.
–	Body dorsally either uniformly black (Fig. 11), or with variously well developed reddish markings (typical form) (Fig. 10); antennomeres 1–4 dark rufous, contrasting with dark piceous to black antennomeres 5–10; antennomere 11 at least partially light reddish; prosternal process narrower, more acute distally (Fig. 47); eyes almost contiguous, separated by less than maximum width of antennomere 1	*Eustrophopsis ornatus* (Van Dyke)
11	Body dorsally and ventrally distinctly brown (Fig. 8), not piceous or black in color; antennomeres 1–4 and 11 only slightly lighter in color than 5–10, not distinctly contrasting; pronotal punctation relatively coarse, punctures larger in diameter than that of setae;known from southwestern U.S. only (Fig. 62)	*Eustrophopsis indistinctus* (LeConte)
–	Body dorsally dark piceous to black (Fig. 6); legs and antennomeres 1–4 distinctly reddish, contrasting dark color of elytra epipleura and prothorax; antennomere 11 orange-red, lighter than 1–4; pronotal punctation fine, punctures not larger in diameter than that of setae; very widespread in distribution (Fig. 60)	*Eustrophopsis bicolor* (Fabricius)

### Tribe Holostrophini Nikitsky, 1998

#### Pseudoholostrophus

Nikitsky 1983

http://species-id.net/wiki/Pseudoholostrophus

Pseudoholostrophus Nikitsky 1983: 37.—Type species: *Hallomenus klapperichi*Pic 1954 (orig. des.); Nikitsky 1998: 40; Young and Pollock 2002: 416; Nikitsky 2008: 63; Pollock 2008: 268.

##### Note.

This genus was described by Nikitsky (1983) on the basis of examination of *Hallomenus klapperichi* Pic, which now is included in (and is the type species of) *Pseudoholostrophus*. According to Nikitsky (1998), *Pseudoholostrophus* differs from *Holostrophus* in its smaller and more weakly emarginate eyes, and in the prosternal process not extending behind the posterior edge of the procoxae. There are four species in *Pseudoholostrophus* (Nikitsky 1998), two of which are Nearctic in distribution. The other two species, *Pseudoholostrophus klapperichi* (Pic) and *Pseudoholostrophus chinensis* Nikitsky, are known from China. Nikitsky (1998) divided the genus into two subgenera; all species other than *Pseudoholostrophus discolor* (Horn) are placed in the nominate subgenus. *Holostrophus discolor* (Horn) was transferred into the subgenus *Holostrophinus* of *Pseudoholostrophus* by Nikitsky (1998). There are two Nearctic species in this genus.

### Subgenus *Pseudoholostrophus* Nikitsky, 1983

#### Pseudoholostrophus (Pseudoholostrophus) impressicollis

(LeConte, 1874)

http://species-id.net/wiki/Pseudoholostrophus_impressicollis

**Figures 1–4.**
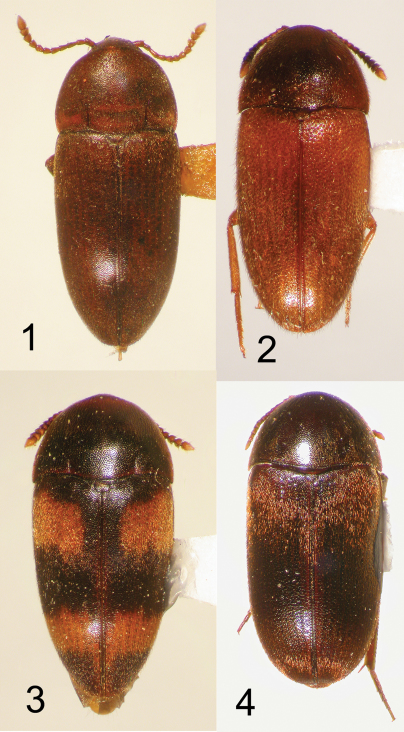
Nearctic Eustrophinae, dorsal habitus. **1** *Pseudoholostrophus impressicollis*, CA: Humboldt Co., TL = 4.9 mm **2** *Pseudoholostrophus discolor*, NH: Strafford Co., TL = 5.0 mm **3** *Holostrophus bifasciatus*, IL: Clark Co. TL = 5.0 mm **4** *Eustrophus tomentosus*, AZ: Coconino Co., TL = 5.5 mm.

Figures 1 14 26 38 53 65

Eustrophus impressicollis LeConte 1874: 69.—Canada, Vancouver [Island?]; Austin 1880: 40; Henshaw 1885: 124.
Holostrophus impressicollis (LeConte).— Horn 1888: 36; Champion 1898: 66; Leng 1920: 238; Csiki 1924: 10; Hatch 1965: 67, Plate IX, fig. 1; LeSage 1991: 246; Poole and Gentili 1996: 299.
Pseudoholostrophus (Pseudoholostrophus) impressicollis (LeConte).—Nikitsky 1998: 47; Young and Pollock 2002: 416; Pollock 2008: 268, 290.

##### Diagnosis.

This distinctive species may be separated by all other Nearctic Eustrophinae by the following combination of characters: color uniformly red-brown; dorsal setae very short, inconspicuous; eyes widely separated; meso- and metatibiae smooth, without oblique ridges; distribution in westernmost North America.

##### Description.

Nikitsky (1998: 47-48, plate 7, Figures 9-11) provided a fairly detailed description of *Pseudoholostrophus impressicollis*.The following abbreviated description was taken from Pollock (2008: 268). TL 6.0-6.2 mm; GEW 2.0–2.7 mm. Body elongate oval, moderately parallel sided (Fig. 1), moderately convex dorsally (Fig. 14); dorsal and ventral color uniformly dark rufous, including antennae and legs; dorsal pubescence very short, inconspicuous; eyes widely separated (> 3× length of first antennomere) (Fig. 26), medial margin of eye slightly emarginate; antennae moderately elongate, antennomeres 7–10 distinctly widened (Fig. 26); antennomere 7 triangular, 8–10 wider than long; antennal sensilla completely annular; last maxillary palpomere distinctly widened, securiform; prosternal process (Fig. 38) elongate, spatulate distally, extended past posterior margin of procoxae, bent dorsally at distal end; prothoracic episternal suture absent; elytral punctation fine, punctures not arranged in longitudinal striae; meso- and metatibiae with scattered short spines, without oblique ridges (Fig. 53).

##### Distribution

(Fig. 65). The range of this species is restricted to the western coast of North America, from the Queen Charlotte Islands in the north, to west central California. Hatch (1965) recorded it from Washington State, without giving a detailed locality. Only 21 specimens were examined, from the following: **CANADA**: BRITISH COLUMBIA. **UNITED STATES**: CALIFORNIA: Alpine, Humboldt, Siskiyou, Trinity. OREGON: Curry, Josephine, Lane, Lincoln, Polk. (Complete label data given in Appendix 1).

##### Types.

*Eustrophus impressicollis* LeConte. LECTOTYPE, sex unknown, labeled “Vanc. / Type 4781 / Eu. impressicollis Lec.”, in MCZ.

##### Natural history.

Label data: under bark of pine; in rotten log; fungus.

#### Subgenus Holostrophinus

Nikitsky 1998

http://species-id.net/wiki/Holostrophinus

Holostrophinus Nikitsky 1998: 40.—Type species: *Holostrophus discolor* Horn (orig. des.)

##### Note.

According to Nikitsky (1998), this subgenus differs from *Pseudoholostrophus* in its double pronotal punctation and the indistinct pair of pronotal impressions. There is only a single world species in subgenus *Holostrophinus*.

#### Pseudoholostrophus (Holostrophinus) discolor

(Horn, 1888)

http://species-id.net/wiki/Pseudoholostrophus_discolor

Figures 2 15 27 39 65

Holostrophus discolor Horn 1888: 36.—U.S.A., Virginia (“Two specimens collected in Virginia by Mr. Ulke...”); Henshaw 1889: 131; Champion 1898: 66; Leng 1920: 238; Csiki 1924: 10; LeSage 1991: 246; Poole and Gentili 1996: 299.
Pseudoholostrophus (Holostrophinus) discolor (Horn).—Nikitsky 1998: 43; Young and Pollock 2002: 416; Pollock 2008: 269, 290.

##### Diagnosis.

This species may be diagnosed on the following combination of characters: eyes widely separated; dorsal setae conspicuous; pronotum with anterior margin elevated above level of head; meso- and metatibiae smooth, without oblique ridges; distribution in eastern North America.

##### Description.

Nikitsky (1998: 43–44, plate 7, Figures 12–15) provided a description of the adult of *Pseudoholostrophus (Holostrophinus) discolor*, based on examination of a single specimen only. Pollock (2008: 269) provided the following shorter description: TL 3.6–5.7 mm; GEW 1.6–2.4 mm; body (Fig. 2) elongate oval, rather parallel-sided, moderately convex dorsally (Fig. 15); dorsal color rufous, pronotum in most specimens slightly darker than elytra; some specimens with lighter humeral area on elytra; antennomeres 1–5 rufous, 6–10 rufopiceous, 11 light rufous; venter uniformly red-brown; dorsal pubescence relatively long, conspicuous, with some erect hairs; eyes widely separated (space > 3× length of antennomere 1), inner eye margin slightly emarginate; antennae (Fig. 27) relatively short, antennomere 7–11 distinctly widened; antennomere 7 triangular, 8–10 distinctly wider than long; antennal sensilla completely annular; last maxillary palpomere slightly widened, subsecuriform; prosternal process (Fig. 39) elongate, spatulate distally, extended to past posterior margin of procoxae, bent dorsally at distal end; prothoracic episternal suture absent; elytral punctation relatively coarse, punctures not arranged in longitudinal striae; meso- and metatibiae with scattered short spines, oblique ridges absent.

##### Distribution

(Fig. 65). Relatively few specimens of this species have been collected and/or examined for this study; the known distribution is distinctly eastern, the westernmost locality being in western Indiana (Parke County). The 25 specimens examined are from the following: **CANADA**: NEW BRUNSWICK, QUEBEC. **UNITED STATES**: INDIANA: Parke. MARYLAND: Calvert, Prince Georges. MASSACHUSETTS: Middlesex. NEW HAMPSHIRE: Carroll, Grafton, Strafford. PENNSYLVANIA: Warren. RHODE ISLAND: Kent. TENNESSEE: Sevier. VIRGINIA. (complete label data in Appendix 1).

##### Types.

*Holostrophus discolor* Horn. LECTOTYPE, sex unknown, labeled “Va. / Henry Ulke Beetle Coll. CMNH Acc. No. 1645 / Holostrophus discolor Horn”, in CMNH. Paralectotype in MCZ.

##### Natural history.

Label data: in polypore fungus; malaise trap (NH, August); intercept trap (PA, July-August; RI, July-August); on or near fleshy polypore fungi on beech log. According to Chantal (1985), individuals of this species are captured on small polypores on trunks of trees, especially *Prunus pensylvanica* and maples (*Acer* spp.).

#### Holostrophus

Horn 1888

http://species-id.net/wiki/Holostrophus

Holostrophus Horn 1888: 32.—Type species: *Eustrophus bifasciatus* Say (subs. des.; Nikitsky 1998: 48); Blatchley 1910: 1293; Champion 1915: 139; Leng 1920: 238; Hatch 1965: 66; Nikitsky 1989: 17; LeSage 1991: 246; Nikitsky 1992: 434; Poole and Gentili 1996: 299; Young and Pollock 2002: 416; Nikitsky 2007: 13; Nikitsky 2008: 63; Pollock 2008: 270.

##### Note.

According to Nikitsky (2007), this genus comprises 19 world species, distributed in the Russian Far East, Korea, China, Japan, Oriental region, and in the Nearctic region. In North America, there is a single species: *Holostrophus bifasciatus* (Say). In further justifying his separation of the genera *Pseudoholostrophus* and *Holostrophus*, Nikitsky (1998: 40) stated that “it seems noteworthy that species of *Pseudoholostrophus* display the elytra either one-color but not black or with a light humeral spot only, or with a clarified diffused transverse fascia in basal part. A more clearly evident reddish-yellow or red spotty pattern of the elytra is characteristic of *Holostrophus*, not *Pseudoholostrophus*”. Nikitsky (2007) added a new subgenus (*Paraholostrophus*), based on three Oriental species. The sole Nearctic species is a representative of the nominative subgenus.

#### Holostrophus bifasciatus

(Say, 1824)

http://species-id.net/wiki/Holostrophus_bifasciatus

Figures 3 16 28 40 66

Mycetophagus 4 maculatus Melsheimer 1806: 14 [catalogue].—(“Pennsylvania”).
Eustrophus bifasciatus Say 1824: 282.—(“Inhabits United States...I obtained a specimen many years ago near Philadelphia, and we lately captured another in the North-Western Territory”); Melsheimer 1853: 143; Crotch 1873: 112; Henshaw 1885: 124.
Eustrophus 4-maculatus Melsheimer 1846: 58.—Melsheimer 1853: 143 (syn.); Leng 1920: 238; Csiki 1924: 9; Poole and Gentili 1996: 299; LeSage 1991: 246.
Holostrophus bifasciatus (Say).—Horn 1888: 36; Champion 1898: 66; Blatchley 1910: 1293; Leng 1920: 238; Csiki 1924: 9; Poole and Gentili 1996: 299; LeSage 1991: 246; Young and Pollock 2002: 416; Pollock 2008: 271, 290; Majka and Pollock 2010: 455.

##### Diagnosis.

*Holostrophus bifasciatus* is the only Nearctic species of Eustrophinae with a distinct, quadrimaculate elytral color pattern; other diagnostic features include the widely separated eyes and smooth meso- and metatibiae (without oblique ridges).

##### Description

(from Pollock 2008: 271) TL 4.1–5.5 mm; GEW 1.9–2.5 mm. Body elongate oval, distinctly tapered posteriorly (Fig. 3), distinctly convex dorsally (Fig. 16); dorsal color dark rufous to almost black; in most specimens, color of pronotum lighter than color of elytra; elytra with 4 yellow-red, subquadrate maculae (Figures 3, 16): anterior pair near humeri and not attaining suture, posterior pair in apical third of elytra, attaining suture in some specimens; antennomeres uniformly rufous in color, antennomere 11 slightly lighter in color than preceding articles; venter uniformly dark rufous; dorsal pubescence relatively short, inconspicuous; eyes widely separated (space > 3× length of antennomere 1), medial margin of eye moderately deeply emarginate (Fig. 28); antennae (Fig. 28) relatively short, antennomeres 7–11 distinctly widened; antennomere 7 triangular, 8–10 distinctly wider than long; antennal sensilla completely annular; last maxillary palpomere slightly widened, subsecuriform; prosternal process (Fig. 40) elongate, spatulate distally, extended to past posterior margin of procoxae, bent dorsally at distal end; prothoracic episternal suture absent; elytral punctation fine, not arranged in longitudinal striae; meso- and metatibiae with scattered short spines, oblique ridges absent.

##### Distribution

(Fig. 66). The range of this species encompasses much of eastern North America, with only several records from west of the Mississippi River in the United States. The westernmost record from Canada is from northwestern Ontario. The 557 specimens examined are from the following: **CANADA**: NOVA SCOTIA, ONTARIO, PRINCE EDWARD ISLAND, QUEBEC. **UNITED STATES**: ALABAMA: Dale, Lee, Shelby. ARKANSAS: Drew, Logan, Newton, Polk, Pulaski, Washington. CONNECTICUT: Fairfield, Hartford, Litchfield, New Haven. DELAWARE: Sussex. DISTRICT OF COLUMBIA. FLORIDA: Hamilton, Liberty, Monroe. GEORGIA: Bartow, Clarke, Fulton, Jackson, Taliaferro. ILLINOIS: Bond, Carroll, Champaign, Clark, Coles, Cook, DuPage, Edgar, Kendall, Knox, LaSalle, Peoria, Putnam, St. Clair, Sangamon, Union, Wabash, Will. INDIANA: Marion, Parke, Porter, Posey, Wayne. IOWA: Johnson. KANSAS: Shawnee. KENTUCKY: Butler, Jefferson. LOUISIANA: Natchitoches. MAINE: Kennebec. MARYLAND: Baltimore, Calvert, Cecil, Dorchester, Harford, Montgomery, Prince George’s, Talbot, Wicomico, Worcester. MASSACHUSETTS: Hampden, Hampshire, Middlesex, Norfolk, Suffolk. MICHIGAN: Livingston, Oakland, Wayne. MINNESOTA: Cook. MISSISSIPPI: George. MISSOURI: Hickory, Randolph, St. Louis. NEBRASKA: Sarpy. NEW HAMPSHIRE: Hillsborough, Rockingham, Strafford. NEW JERSEY: Bergen, Burlington, Cumberland, Essex, Middlesex, Ocean. NEW YORK: Bronx, Erie, Monroe, Onondaga, Orange, Richmond, Suffolk, Queens, Tompkins, Westchester. NORTH CAROLINA: Buncombe, Gaston, Moore, Wake. OHIO: Clark, Hamilton, Franklin, Ross, Wyandot. PENNSYLVANIA: Adams, Allegheny, Armstrong, Berks, Dauphin, Philadelphia, Pike, Westmoreland. SOUTH CAROLINA: Florence, Oconee, Pickens. TENNESSEE: Chester, Cumberland, Morgan. TEXAS: Sabine. VIRGINIA: Bath, Clarke, Fairfax, Lee. WEST VIRGINIA: Berkeley, Kanawha, Lewis, Morgan, Roane. WISCONSIN: Dane, Grant, Waukesha. (Complete label data given in Appendix 1).

##### Types.

*Eustrophus bifasciatus* Say. NEOTYPE (designated by Pollock 2008), sex unknown, labeled “[faded pink circle] / E. bifasciatus Say 4-maculatus Mels.”, in MCZ (LeConte collection).

##### Natural history.

Specimens have been collected in association with dead logs and/or associated fungi. Label data: *Laetiporus sulphureus* (AR); ex polypore in pine logs (AR); *Trametes versicolor* (AR); *Schizopora paradoxa* (AR); BLT (June, AL); ex polypore on cherry tree (AR); under loose pine bark (DE); under pine bark (GA); Malaise trap (March, June, GA); oak log (IL); sugar trap (May, IL); UV light (April, IL); flight trap (Aug-Sept, IL). Chantal (1985) stated that specimens have been collected from under bark of fallen *Pinus strobus*, on which polypores were growing; also, specimens are known from *Polyporus betulinus*.

### Tribe Eustrophini Gistel, 1848

#### Eustrophus

Illiger 1802

http://species-id.net/wiki/Eustrophus

Eustrophus Illiger 1802: 301.—Type species: *Mycetophagus dermestoides*Fabricius 1792 (monotypy); Horn 1888: 32; Blatchley 1910: 1292; Champion 1915: 138; Champion 1916: 1; Leng 1920: 238; Hatch 1965: 66; Nikitsky 1989: 20; LeSage 1991: 246; Nikitsky 1992: 435; Poole and Gentili 1996: 299; Nikitsky 1998: 54; Young and Pollock 2002: 416; Pollock 2008: 273.

##### Note.

This genus comprises four species, distributed in Europe, Russian Far East, Japan, southern China, and North America (Nikitsky 1998). All of the older species of Eustrophinae were originally placed in *Eustrophus*, before the description of *Holostrophus* and *Eustrophinus*. Now, only a single Nearctic species is placed in *Eustrophus*.

#### Eustrophus tomentosus

Say 1826

http://species-id.net/wiki/Eustrophus_tomentosus

Figures 4 17 29 41 50 67

Mycetophagus niger Melsheimer 1806: 14 [Catalogue]; Melsheimer 1846: 58.
Mycetophagus tomentosus Melsheimer 1806: 14 [catalogue].—(“Pennsylvania”); Say 1826: 239.
Eustrophus niger Melsheimer 1846: 58.—Melsheimer 1853: 143 (syn.); Leng 1920: 238; LeSage 1991: 246; Poole and Gentili 1996: 299.
Eustrophus tomentosus Say 1826: 239.—U.S.A., Illinois (no specific localities given in description); Melsheimer 1853: 143; Crotch 1873: 112; Provancher 1877: 466; Henshaw 1885: 124; Horn 1888: 35; Blatchley 1910: 1293; Leng 1920: 238; Csiki 1924:10; Hatch 1965: 66; LeSage 1991: 246; Poole and Gentili 1996: 299; Young and Pollock 2002: 416; Majka and Pollock 2006: 53; Pollock 2008: 273, 290; Majka and Pollock 2010: 455.

##### Diagnosis.

The following combination of characters is diagnostic for this species: widely separated eyes; antennal sensilla interrupted, not completely annular; dorsal setae distinctly golden to brown; meso- and metatibiae with oblique ridges.

##### Description

(from Pollock 2008: 273). TL 4.5–6.0 mm; GEW 2.1–3.0 mm. Body oval, parallel-sided (Fig. 4), distinctly convex dorsally (Fig. 17); dorsal color dark brown, with golden sheen due to dense pubescence; venter and antennae uniformly dark rufous, lighter than dorsal color; dorsal pubescence relatively short, but dense, giving distinct sheen (almost iridescent); eyes widely separated (space ~1.5 × length of first antennomere), inner margins deeply emarginate (Fig. 29); antennomeres 2–11 only slightly but evenly widened to apex, without distinct change in size between any 2 adjacent antennomeres; distal antennomeres subtriangular to nearly quadrate; antennal sensilla not completely annular, present on short sides of antennomeres only; last maxillary palpomere slightly securiform; prosternal process (Fig. 41) triangular, narrowed distally, extended to slightly short of posterior margin of procoxae; prothoracic episternal suture absent (Fig. 50); elytral punctation relatively fine, punctures arranged in longitudinal striae; meso- and metatibiae with oblique ridges present.

##### Distribution

(Fig. 67). The distribution of this species is virtually transcontinental, with a gap in the interior; for example, no specimens are known from Saskatchewan and Alberta. Most records from the United States are eastern, but specimens are also known from the Pacific Northwest, California, and Arizona. The 983 specimens examined in this study are from the following: **CANADA**: BRITISH COLUMBIA, MANITOBA, NOVA SCOTIA, ONTARIO, QUEBEC. **UNITED STATES**: ALABAMA: Lee, Madison, Marion, Mobile. ARIZONA: Coconino, Pima. ARKANSAS: Crawford, Faulkner, Fulton, Logan, Polk, Pulaski. CALIFORNIA: Butte, Calaveras, El Dorado, Placer, Yuba. COLORADO. CONNECTICUT: Litchfield. DELAWARE: Sussex. DISTRICT OF COLUMBIA. FLORIDA: Dixie, Monroe. GEORGIA: Bartow, Clarke, Echols. IDAHO: Boundary, Clearwater, Latah. IOWA: Johnson, Keokuk, Story. ILLINOIS: Bond, Champaign, Clark, Coles, Cook, LaSalle, McHenry, St. Clair, Union, Wabash. INDIANA: Montgomery, Parke, Porter, Tippecanoe, Vigo. KANSAS: Douglas. KENTUCKY: Butler, Henderson. LOUISIANA: Natchitoches. MARYLAND: Anne Arundel, Charles, Dorchester, Montgomery, Prince George’s, Talbot. MASSACHUSETTS. MICHIGAN: Berrien, Charlevoix, Fillmore, Gogebic, Ingham, Kalamazoo, Kent, Lapeer, Leelanau, Livingston, Macomb, Midland, Monroe, Oakland, St. Joseph, Shiawassee, Washtenaw, Wayne. MINNESOTA: Crow Wing, Hennepin, Sherburne, Washington. MISSISSIPPI: George, Pearl River. MISSOURI: Boone, Dent, Franklin, Randolph, St. Charles. MONTANA: Dawson, Lake, Lincoln, Missoula, Ravalli, Rosebud, Sanders. NEBRASKA: Douglas, Sarpy. NEW HAMPSHIRE: Carroll. NEW JERSEY: Burlington, Gloucester. NEW MEXICO: San Miguel. NEW YORK: Erie, Essex, Genesee, Niagara, Onondaga, Orleans, St. Lawrence, Tompkins. NORTH CAROLINA: Durham, Moore, Stokes, Wake. NORTH DAKOTA: Pembina, Richland. OHIO: Athens, Butler, Clinton, Delaware, Hocking, Ottawa, Portage, Scioto, Vinton, Wayne, Wyandot. OKLAHOMA: Latimer, Oklahoma. OREGON: Benton, Crook, Douglas, Harney, Jackson, Lane, Linn, Multnomah, Wallowa, Yamhill. PENNSYLVANIA: Allegheny, Tioga, Westmoreland. SOUTH CAROLINA: Anderson, Florence, Pickens. TENNESSEE: Shelby. TEXAS: Angelina, Brewster, Montgomery, Sabine, Walker. UTAH: Washington. VERMONT: Franklin. VIRGINIA: Alexandria, Hampton, Nelson, Shenandoah. WASHINGTON: Walla Walla. WEST VIRGINIA: Braxton, Doddridge, Greenbrier, Hampshire, Jackson, Kanawha, Lincoln, Mason, Mineral, Nicholas, Pendleton, Randolph, Ritchie, Roane, Taylor, Webster, Wirt, Wood. WISCONSIN: Dane, Racine, Sauk, Washington. (Complete label data given in Appendix 1).

##### Types.

*Eustrophus tomentosus* Say. NEOTYPE (designated by Pollock 2008), sex unknown, labeled “Ill. / E. tomentosus Say. niger Mels.”, in MCZ (LeConte collection).

##### Natural history.

Label data: *Pseudotsuga taxifolia* (BC), from fungus (BC), evening flight, 4.ix (BC), ex *Populus trichocarpa* (BC), in bark *Larix occidentalis* (BC), fleshy fungus on tree (ON), under bark of pine (ON), under wet moldy bark on dead tree (ON), elm (QC), uv light trap (QC), cut surface of stump (QC), *Ulmus americana* (QC), under bark of pine (AL), *Spongipellis unicolor* (AR), *Climacodes septentrionale* (AR), *Trametes versicolor* (AR), under pine bark (CA), Lindgren trap with turpentine bait (CA), on *Pinus ponderosa* (CA), ex. *Polyporus* fungus (FL), uv light in oak-maple forest (IA), fungus on dead pine (ID), under bark of old pine stump (MD), in *Peromyscus* nest debris under bark of dead standing *Liriodendron* (MD), fungus on oak (MN), funnel traps (MT), at black light, vi.1970 (NC), malaise trap, viii-ix (NE), window trap, 8–14.vi (NH), light trap, vi (NY), Lindgren funnel trap, alpha-pinene and ethanol lure (OR), stale molasses trap (SC), at wound on oak trunk (SC), malaise in mature hardwood forest, vi (SC), under bark of old dead decid. tree (VT), ex dead oak stump (WI), under bark of chestnut oak (WV). According to Chantal (1985), *Eustrophus tomentosus* are found under bark of dead trees, especially *Ulmus americanus*, as well as at sap exudations.

#### Eustrophopsis

Champion, 1889

http://species-id.net/wiki/Eustrophopsis

Eustrophopsis Champion 1889: 77.—Type species: *Orchesia quindecimmaculatus*Laporte 1840 (orig. des.); Champion 1915: 138; Champion 1916: 1, 138; Csiki 1924: 7; Blackwelder 1945: 494; Nikitsky 1998: 58; Young and Pollock 2002: 416; Pollock 2008: 276.
Eustrophinus Seidlitz 1898: 438, 440.—Type species: *Mycetophagus bicolor* Fabricius 1798 (orig. des.); Champion 1916: 3; Leng 1920: 238; Csiki 1924: 8; Leng and Mutchler 1933: 36; Blackwelder 1945: 495; Hatch, 1965: 66; LeSage 1991: 246; Poole and Gentili 1996: 299; Nikitsky 1998: 58; Young and Pollock 2002: 416; Pollock 2008: 276.
Eustrophus Champion 1889: 75, *nec*. Illiger 1802.

##### Note.

*Eustrophopsis* is the most diverse world genus of Eustrophinae, with approximately 55 species, known from Afrotropical, Neotropical and Nearctic regions. Descriptions of the species are scattered through the literature, and there does not exist a comprehensive work on the entire genus, which is in need of revision. Nikitsky (1998: 58) stated that it is “remarkable that *Eustrophopsis* seems to be absent both from the Oriental Region and Palaearctic. It may be so that it is replaced there by species of the genera *Holostrophus* and *Synstrophus* unknown from the Neotropical and Afrotropical regions”.

*Eustrophopsis* was described by Champion (1889) based on his examination of specimens collected in the Neotropics. Specimens that possessed a notched prosternal process were separated from *Eustrophinus* Seidlitz and placed in *Eustrophopsis*. Nikitsky (1998) synonymized these two genera, stating that the emarginated prosternal character was not satisfactory to separate them. Therefore, *Eustrophopsis* became a senior synonym of *Eustrophinus*.

Although admittedly preliminary, the Nearctic species of *Eustrophopsis* seem referable into a number of informal groupings: *Eustrophopsis confinis* (very widely separated eyes); *Eustrophopsis bicolor*, *Eustrophopsis brunneimarginatus*, and *Eustrophopsis indistinctus* (body shape, color pattern and shape of antennomeres); *Eustrophopsis arizonensis* (very rugose macrosculpture of proepisterna), and *Eustrophopsis ornatus* and *Eustrophopsis crowdyi*, sp. n. (enlarged, sexually dimorphic antennomeres, males with distinctive sensilla on antennomeres 5–10).

#### Eustrophopsis confinis

(LeConte, 1866)

http://species-id.net/wiki/Eustrophopsis_confinis

**Figures 5–8.**
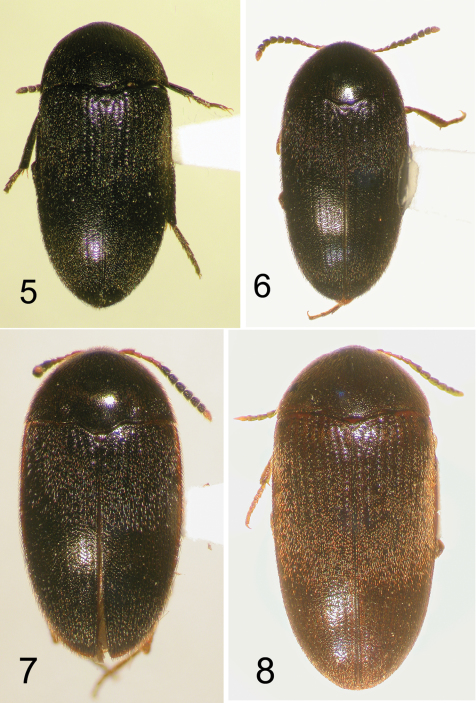
Nearctic Eustrophinae, dorsal habitus. **5** *Eustrophopsis confinis*, MB, TL = 5.4 mm **6** *Eustrophopsis bicolor*, MB: Lockport, TL = 5.7 mm **7** *Eustrophopsis brunneimarginatus*, FL: Liberty Co., TL = 4.5 mm **8** *Eustrophopsis indistinctus*, NM: Roosevelt Co., TL = 5.4 mm.

Figures 5 18 30 42 56 68

Eustrophus confinis LeConte 1866: 152.—(“Canada, Lake Superior, and Western States”); Crotch 1873: 112; Schwarz 1878: 463; Henshaw 1885: 124; Horn 1888: 35; Dury 1906b: 260; Csiki 1924: 10.
Eustrophinus confinis (LeConte).—Leng 1920: 238; Hatch 1965: 66; LeSage 1991: 246; Poole and Gentili 1996: 299.
Eustrophopsis confinis (LeConte).—Majka and Pollock 2006: 53; Pollock 2008: 280, 290; Majka and Pollock 2010: 455.

##### Diagnosis.

*Eustrophopsis confinis* is the only Nearctic member of this genus with very widely separated eyes dorsally. Other diagnostic features include: uniformly dark body; meso- and metatibiae with oblique ridges.

##### Description

(from Pollock 2008: 280). TL 4.8–6.1 mm; GEW 2.4–3.1 mm. Body ovate, subparallel-sided (Fig. 5), moderately convex dorsally (Fig. 18); dorsal and ventral color uniformly dark piceous to black; antennomeres 1–4 and 11 slightly lighter in color than antennomeres 5–10; dorsal pubescence relatively long, conspicuous; eyes widely separated (Fig. 30) (space 1–1.5 × length of antennomere 1), medial margin deeply emarginate; antennomeres 5–10 subquadrate; antennal sensilla completely annular; last maxillary palpomere unmodified, fusiform; prosternal process (Fig. 42) triangular, moderately narrowed distally, extended to slightly short of posterior margin of procoxae; prothoracic episternal suture present; elytral punctation coarse, punctures arranged in longitudinal striae; meso- and metatibiae with oblique ridges; male with small, ovate setiferous pit on ventral edge of profemur; aedeagus (Fig. 56) with basal and apical piece of segment subequal in length; struts on median lobe relatively wide and short, inner margins U-shaped; sternite 9 basally Y-shaped, with very short stem.

##### Distribution

(Fig. 68). Specimens of *Eustrophopsis confinis* are seemingly rarely collected, and localities are scattered across the northern United States and southern Canada. The record from northern Saskatchewan represents the northernmost extent of the entire subfamily in North America. The 32 specimens are from the following jurisdictions: **CANADA**: BRITISH COLUMBIA, MANITOBA, NOVA SCOTIA, ONTARIO, SASKATCHEWAN. **UNITED STATES**: IDAHO: Kootenai, Latah. MAINE: Androscoggin. MICHIGAN: Mackinac. MONTANA: Carter, Rosebud. NEBRASKA. NEW HAMPSHIRE: Coos, Hillsborough. SOUTH DAKOTA: Pennington. WASHINGTON: Stevens. WISCONSIN. (Complete label data given in Appendix 1).

##### Types.

*Eustrophus confinis* LeConte. LECTOTYPE (designated by Pollock 2008), sex unknown, labeled “Wis / Type 4780 / E. confinis Lec”, in MCZ.

##### Natural history.

Label data: *Polyporus anceps* (ME). Chantal (1985) stated that although no details were available for this species, it should probably be similar in habits and ecology to other members of the genus.

##### Notes.

As mentioned elsewhere, the very widely separated eyes of individuals of *Eustrophopsis confinis* seem to be unique within the genus, and are reminiscent of the condition seen in members of Holostrophini and *Eustrophus*.

#### Eustrophopsis bicolor

(Fabricius, 1792)

http://species-id.net/wiki/Eustrophopsis_bicolor

Figures 6 19 31 43 57 63 69

Mycetophagus bicolor Fabricius 1792: 497.—Fabricius 1801: 566; Olivier 1811: 70.
Eustrophus bicolor (Fabricius).—Melsheimer 1853: 143; Crotch 1873: 112; LeConte 1873: 335; Provancher 1877: 467; Schwarz 1878: 463; Henshaw 1885: 124; Horn 1888: 35; Weiss 1919: 133; Weiss and West 1920: 10; Weiss 1922: 47; Csiki 1924: 8.
Eustrophinus bicolor (Fabricius).—Leng 1920: 238; Poole and Gentili 1996: 299; LeSage 1991: 246; Viedma 1971.
Eustrophopsis bicolor (Fabricius).—Pollock 2008: 277, 290; Lawrence and Leschen 2010: 515, fig. 11.5.2.D.

##### Diagnosis.

This common species may be separated from the other Nearctic Eustrophinae based on the following combination of characters: antennomeres distinctly contrasting: 1–4 rufous, 5–10 dark piceous to black, 11 yellow-orange; pronotal punctation very fine, punctures not larger than diameter of setae; males with oval, setiferous pit on ventral edge of profemur; meso- and metatibiae with numerous, oblique ridges.

##### Description

(from Pollock 2008: 277). TL 4.2–6.5 mm; GEW 2.0–3.2 mm. Body elongate oval, moderately tapered posteriorly (Fig. 6), distinctly convex dorsally (Fig. 19); dorsal color dark piceous to black; antennae tricolored: antennomeres 1–4 red, 5–10 piceous, antennomere 11 yellow-red, distinctly contrasting against preceding darker antennomeres; maxillary palpi similar in color to basal antennomeres; venter a combination of dark (same as dorsum) and lighter colored sclerites (abdominal ventrites in most specimens contrasting darker color of thorax); dorsal pubescence relatively long, conspicuous; eyes narrowly separated (Fig. 31), or almost contiguous (space < length of antennomere 1), medial margin moderately emarginate; antennomeres 2–4 short, submoniliform, antennomeres 5–10 widened, becoming more triangular toward antennomeres 9–10; antennal sensilla completely annular; last maxillary palpomere not modified; prosternal process (Fig. 43) triangular, narrowed distally, extended to slightly short of posterior margin of procoxae; prothoracic episternal suture present; elytral punctation coarse, punctures arranged in longitudinal striae; meso- and metatibiae with oblique ridges present; male with small, ovate setiferous pit on ventral edge of profemur; aedeagus (Figures 57, 63) with basal and apical piece of tegmen subequal in length; struts on median lobe elongate, narrow, inner margins V-shaped; sternite 9 basally Y-shaped, with short stem.

##### Distribution

(Fig. 69). *Eustrophopsis bicolor* is the most commonly collected and/or geographically widespread species in the subfamily. It exhibits an eastern distribution in Canada, with no records west of Winnipeg, Manitoba. In the United States, most records are eastern; however, scattered localities are known from several western states. This species is the only otherwise Nearctic eustrophine recorded from the West Indies. The 2,799 examined specimens are from the following jurisdictions: **BAHAMAS**. **CANADA**: MANITOBA, ONTARIO, QUEBEC. **UNITED STATES**: ALABAMA: Colbert, Greene, Jefferson, Lee, Madison, Mobile, Tuscaloosa. ARIZONA. Santa Cruz. ARKANSAS: Faulkner, Fulton, Garland, Hempstead, Johnson, Polk, Pulaski, Washington. CALIFORNIA: Trinity. COLORADO: Larimer. CONNECTICUT: Fairfield, Litchfield, New Haven, Tolland. DELAWARE: New Castle, Sussex. DISTRICT OF COLUMBIA. FLORIDA: Alachua, Baker, Brevard, Calhoun, Dade, Duval, Hernando, Highlands, Hillsborough, Jackson, Liberty, Monroe, Okeechobee, Orange, Osceola, Pinellas, Polk, Seminole, St. Lucie, Volusia, Wakulla. GEORGIA: Baker, Bartow, Calhoun, Camden, Clarke, Dekalb, Dougherty, Floyd, Fulton, Greene, Gwinnett, Lowndes, McIntosh, Meriwether, Muscogee, Paulding, Rabun, Thomas. IDAHO: Jerome, Twin Falls. ILLINOIS: Adams, Alexander, Calhoun, Champaign, Clark, Clay, Clinton, Coles, Cook, DuPage, Edgar, Effingham, Jackson, Jasper, Jefferson, Jersey, Johnson, Kendall, lake, LaSalle, Lawrence, Macon, Marion, Mason, McHenry, McLean, Peoria, Pike, Platt, Pope, Putnam, Richland, Sangamon, Stephenson, Vermilion, Wabash, Washington, White, Whiteside, Will, Winnebago. INDIANA: Bartholomew, Crawford, Howard, Lake, Laporte, Monroe, Parke, Perry, Porter, Posey, Tippecanoe, Vigo, Wayne. IOWA: Benton, Henry, Keokuk, Johnson, Linn, Osceola, Story. KANSAS: Atchison, Douglas, Labette, Lyon, Miami, Riley, Sedgwick, Shawnee, Trego, Wilson, Wyandotte. KENTUCKY: Butler, Christian, Green, Henderson, Taylor. LOUISIANA: Bossier, Caddo, Claiborne, Concordia, East Baton Rouge, East Feliciana, Jefferson, Livingstone, Madison, Natchitoches, Orleans, Webster, West Feliciana. MARYLAND: Allegany, Anne Arundel, Baltimore, Calvert, Caroline, Cecil, Charles, Dorchester, Frederick, Montgomery, Prince George’s, Queen Anne’s, St. Mary’s, Talbot, Wicomico, Worcester. MASSACHUSETTS: Bristol, Dukes, Essex, Hampden, Hampshire, Middlesex, Norfolk, Suffolk, Worcester. MICHIGAN: Allegan, Berrien, Branch, Charlevoix, Clare, Clinton, Eaton, Ingham, Isabella, Kalamazoo, Kent, Lake, Lenawee, Livingston, Marquette, Midland, Monroe, Oakland, Ottawa, Saginaw, Schoolcraft, Shiawassee, Washtenaw, Wayne. MINNESOTA: Crow Wing, Hennepin, Washington. MISSISSIPPI: Bolivar, George, Issaquena, Jackson, Lafayette, Montgomery, Perry, Prentiss, Tallahatchie, Tishomingo. MISSOURI: Boone, Butler, Clay, Franklin, Greene, Jackson, Morgan, Randolph, Reynolds, St. Charles, St. Louis, Vernon. MONTANA: Richland, Rosebud, Valley. NEBRASKA: Cass, Colfax, Cuming, Douglas, Fillmore, Hall, Keith, Lancaster, Merrick, Nemaha, Sarpy, Saunders, Sheridan, Sioux, Thomas. NEW HAMPSHIRE: Rockingham, Strafford. NEW JERSEY: Bergen, Burlington, Cape May, Essex, Gloucester, Mercer, Middlesex, Monmouth, Morris, Ocean, Passaic, Salem, Union. NEW MEXICO: Roosevelt. NEW YORK: Bronx, Columbia, Erie, Nassau, Niagara, Onondaga, Queens, Richmond, Rockland, St. Lawrence, Schuyler, Suffolk, Tompkins, Westchester, Wyoming. NORTH CAROLINA: Bladen, Buncombe, Carteret, Columbus, Durham, Haywood, Henderson, Johnston, Macon, Moore, Robeson, Wake. NORTH DAKOTA: Burleigh, Cass, Ransom, Richland. OHIO: Ashland, Clark, Clermont, Clinton, Cuyahoga, Erie, Franklin, Greene, Hamilton, Hancock, Licking, Montgomery, Ottawa, Scioto, Union, Warren. OKLAHOMA: Adair, Atoka, Beckham, Bryan, Caddo, Cherokee, Cleveland, Garfield, Grady, Latimer, Lincoln, Love, Marshall, Mayes, Muskogee, Okfuskee, Oklahoma, Osage, Payne, Pontotoc, Roger Mills, Tillman, Tulsa, Wagoner. PENNSYLVANIA: Allegheny, Berks, Bucks, Butler, Centre, Chester, Cumberland, Dauphin, Delaware, Erie, Greene, Lancaster, Philadelphia, Westmoreland. RHODE ISLAND: Newport, Providence. SOUTH CAROLINA: Abbevilee, Beaufort, Chester, Clarendon, Dorchester, Florence, Greenville, Kershaw, Oconee, Pickens, Sumter. SOUTH DAKOTA: Brookings, Yankton. TENNESSEE: Cumberland, Davidson, Hardeman, Knox, Lake, Lauderdale, Madison, McMinn, Shelby. TEXAS: Anderson, Angelina, Aransas, Bastrop, Bexar, Blanco, Brazoria, Brazos, Brewster, Brown, Caldwell, Cameron, Cass, Cherokee, Comal, Dallas, Denton, Galveston, Gonzales, Hardin, Harris, Hidalgo, Houston, Jeff Davis, Kenedy, Kerr, Live Oak, Mason, McLennan, Montague, Montgomery, Palo Pinto, Polk, Robertson, Sabine, San Augustine, San Patricio, Smith, Tarrant, Throckmorton, Travis, Tyler, Walker, Williamson, Wood. UTAH: Utah, Washington. VIRGINIA: Bath, Chesapeake, Chesterfield, Clarke, Covington, Essex, Fairfax, Hampton, Loudoun, Newport News, Suffolk, Westmoreland, York. VERMONT: Bennington. WASHINGTON: Asotin. WISCONSIN: Columbia, Dane, Dodge, Green, Jackson,

Milwaukee, Racine, Sauk, Wood. WEST VIRGINIA. Barbour, Braxton, Greenbrier, Hancock, Jackson, Marshall, Mineral, Preston, Putnam, Ritchie, Roane, Tyler, Webster. WYOMING: Goshen. (Complete label data given in Appendix 1).

##### Types.

not examined. This species has been well characterized in the literature by authors such as Horn and LeConte.

##### Natural history.

Label data: underside of fungusy, dead log at night (MB), on piece of cut wood at night (MB), fungusy stump of *Acer negundo* (MB), bracket fungus (ON), in rotting *Pleurotus* (QC), on trunk of dead *Ulmus americana* (QC), under bark of dead maple (QC), under bark of rotting trunk of *Pinus eliottii* with polypore fungi (Bahamas), ex *Fomes* on *Salix* (Bahamas), under bark of *Fagus* (AR), *Trametes versicolor* (AR), *Meripilus giganteus* (AR), polypore tree fungus (AR), under rotting oak bark (CT), in litter at base of dying *Ailanthus* (CT), large orange polypore shelf fungus [? *Polyporus sulphureus*] on standing tree trunk (CT), *Omphalotus olearius* (CT), under bark (DE), shelf fungi (FL), *Griffolia fungus* (FL), *Polyporus hypnoides* (FL), *Polyporus sulphureus* (FL, MA), under bark of dead pine (FL), hibernating under bark, (GA), under bands of tar paper on apple trees (IL), *Armillariella mellea* (GA), *Pleurotus ostreatus* (IL), on shelf fungi (KS), in sweetgum stump (LA), *Polyporus adustus* (LA), *Panus rudis* (MA, NJ), under bark of dead *Pinus virginiana* (MA), under bark dead standing *Quercus* (MA), in *Peromyscus* nest debris under bark dead standing *Liriodendron* (MA), under bark of stump of *Prunus serotina* (MA), fungus on bark (MS), injured cypress (NC), oak (NC), under bark dead standing pine (NC), under oak bark (NC), *Pleurotus* sp. (NC, OK), in mushroom (NC), *Trametes hispida* (ND), under hardwood bark (NJ), ex fungus on *Mimosa* stump (NJ), on polypore on dead *Quercus* (TX), polypore fungus (UT), *Polyporus squamosus* (VT), under poplar bark (WI). Weiss and West (1920) recorded *Eustrophopsis bicolor* from *Pleurotus*, *Polyporus*, *Poria*, *Lentinus* and *Daedalia*. Chantal (1985) provided some details on fungal habitats on/in which adults were collected: *Pleurotus sapidus*, *Polyporus squamosus*, *Polyporus betulinus*, *Polyporus versicolor*, *Polyporus confragosa*. Chantal (1985) and Pollock (2008) observed that individuals of this species are often collected together with adults of *Synstrophus repandus* in the same microhabitats.

##### Notes.

According to LeConte (1873: 335), “the proper authority for this species is Say, its first describer; *Mycetophagus bicolor* Fabr. is probably a *Platydema*.” Pollock (2008) mentioned that no other reference to Say being the author of this species could be found, and in fact, Say himself (1826) considered Fabricius to be the correct author.

Taxonomically, the separation of southern specimens of *Eustrophopsis bicolor* from those of *Eustrophopsis indistinctus* proved to be the most difficult problem in this study. Specimens of *Eustrophopsis bicolor* from northern and eastern North America are very distinctive from the southern specimens of *Eustrophopsis indistinctus* (color and dorsal punctation). However, as the ranges approach one another, distinguishing features between individuals of the two species become somewhat less conclusive.

Analysis of the male genitalia has revealed several consistent differences between *Eustrophopsis bicolor* (Figures 57, 63) and *Eustrophopsis indistinctus* (Figures 59, 64), as follows: 1) tegmen relatively narrow in *Eustrophopsis bicolor*, with apical and basal piece subequal in length; relatively wider in *Eustrophopsis indistinctus*, with apical piece distinctly shorter than basal piece; 2) basal struts of median lobe long and narrow in *Eustrophopsis bicolor*, while shorter and wider in *Eustrophopsis indistinctus*; and 3) ring-like portion of sternite 9 U-shaped in *Eustrophopsis bicolor*, while Y-shaped in *Eustrophopsis indistinctus* (i.e. with a short basal extension).

There appears to be a rather narrow zone of sympatry between the two species, e.g. Roosevelt County, New Mexico and Randall County, Texas. Hybridization may be occurring in the southwestern United States; for example, multiple specimens from Hidalgo County, Texas seem to exhibit a combination of features of both species. Future studies using molecular methods may be fruitful in “fine-tuning” relationships between *Eustrophopsis bicolor* and *Eustrophopsis indistinctus*.

#### Eustrophopsis brunneimarginatus

(Dury, 1906a)

http://species-id.net/wiki/Eustrophopsis_brunneimarginatus

Figures 7 20 32 44 58 70

Eustrophus brunneimarginatus Dury 1906a: 254.—(“Two specimens, Kentucky, near Cincinnati, Ohio”); Dury 1906b: 260; Csiki 1924: 10.
Eustrophinus brunneimarginatus (Dury).— Leng 1920: 238; Poole and Gentili 1996: 299.
Eustrophopsis brunneimarginatus (Dury).—Pollock 2008: 290.

##### Diagnosis.

Specimens of this species may be distinguished from other members of Eustrophinae by the presence of the marginal bands of lighter color along the otherwise dark pronotum and elytra. It is possible that *Eustrophopsis marginatus* (Champion) also occurs in the Nearctic region (see “notes”, below); however, the marginal light markings are even more extensive than in *Eustrophopsis brunneimarginatus*.

##### Description.

TL 4.2–5.4 mm; GEW 2.0–2.8 mm. Body broadly ovate, moderately tapered posteriorly (Fig. 7), distinctly convex dorsally (Fig. 20); dorsal color from dark brown to near black, except for narrow lighter marginal band on pronotum and elytra (Figures 7, 20); light pronotal markings various, in some specimens restricted to extreme lateral margins only, in others extended further onto pronotal disc; light elytral band restricted to lateral margin, inward three striae at most; antennomeres 1–4 and 11 rufous, distinctly lighter than piceous antennomeres 5–10; ventral color uniformly dark rufous, without contrast between color of thoracic and abdominal sclerites; dorsal vestiture relatively long, conspicuous; eyes narrowly separated (Fig. 32), distance between less than maximum width of antennomere 1; eye deeply emarginated around antennal insertion; antennae relatively short, with no discernible sexual dimorphism; antennomeres 6–10 short, approximately equal in width and length, submoniliform; antennal sensilla completely annular; last maxillary palpomere unmodified, apex slightly oblique; pronotal punctation relatively coarse; prosternal process (Fig. 44) elongate, narrowly rounded distally, extended nearly to posterior margin of procoxae; prothoracic episternal suture present, surface of proepisternum smooth, punctures not obscured by rugose macrosculpture; coarse elytral punctures forming multiple striae; meso- and metatibiae with multiple, oblique ridges; male with small, ovate setiferous pit on ventral edge of profemur; aedeagus (Fig. 58) with basal piece of tegmen slightly shorter than apical piece; struts on median lobe long, relatively narrow, inner margins narrowly U-shaped; sternite 9 basally V-shaped to Y-shaped, with very short stem.

##### Distribution

(Fig. 70). Specimens of *Eustrophopsis brunneimarginatus* are known from scattered localities in eastern United States, west to east-central Texas. The 45 examined specimens are from the following jurisdictions: **UNITED STATES**: ALABAMA: Dale, Madison. ARKANSAS: Pulaski. FLORIDA: Liberty. GEORGIA: Monroe. ILLINOIS: Edgar, Wabash. INDIANA: Tippecanoe. LOUISIANA: Grant, Natchitoches. MISSISSIPPI: Tishomingo. MISSOURI: Randolph. NEW HAMPSHIRE: Coos. NORTH CAROLINA: Rockingham. OKLAHOMA: Latimer. SOUTH CAROLINA: Anderson. TEXAS: Anderson, Milam, Sabine, San Augustine, Tyler, Wood. VIRGINIA: Norfolk. WEST VIRGINIA: Hardy. (Complete label data given in Appendix 1).

##### Types.

*Eustrophus brunneimarginatus* Dury. LECTOTYPE (here designated), sex unknown, labeled “Ky near Cin. O. / [red, hand written] Type / [handwritten] Eustrophus brunneimarginatus Dury Type / CMNH E2065", in CNHM. Paralectotype, with same label data as lectotype, except with “June” written on top label.

##### Natural history.

Label data: flight intercept trap (AL); blacklight trap (AR); malaise trap, mixed hardwood/closed canopy seepage slope (FL); beach wash-up (NH); malaise, mature hardwood forest (SC); malaise trap in beech-magnolia forest (TX).

##### Notes.

Dury (1906a) described this new species based on two specimens, which differed from *Eustrophopsis bicolor* based on being smaller, broader, less shining, much less narrowed posteriorly, less distinctly striate, and the light colored border of the pronotum and elytra. However, in the next article in the same journal issue, Dury (1906b) seemingly synonymized his new species with *Eustrophopsis confinis* LeConte. These two species are quite different, however, and it is likely that Dury never examined actual specimen(s) of *Eustrophopsis confinis*.

A series of specimens from Hidalgo Co., Texas (TAMU) resemble *Eustrophopsis brunneimarginatus*, except that the marginal light markings are much more extensive on the pronotum and elytra. It is possible that these specimens represent *Eustrophopsis marginatus* Champion. In the description, Champion (1889) compared *Eustrophopsis marginatus* to *Eustrophopsis bicolor*, as *Eustrophopsis brunneimarginatus* was not yet described. Another species described by Champion (1889) – *Eustrophopsis ovatus* – possesses similar elytral and pronotal markings except that they are even more extensive than *Eustrophopsis marginatus*. I defer judgment on placement of the above mentioned specimens from southern Texas, as well as a possible synonymy of *Eustrophopsis brunneimarginatus* and *Eustrophopsis marginatus*, pending further study of the Neotropical eustrophines and detailed examination of Champion’s types.

#### Eustrophopsis indistinctus

(LeConte, 1851)

http://species-id.net/wiki/Eustrophopsis_indistinctus

Figures 8 21 33 45 51 54 59 64 71 76

Eustrophus indistinctus LeConte 1851: 151.—(“Colorado [River]”); Melsheimer 1853: 143; Crotch 1873: 112; Henshaw 1885: 124; Snow 1885: 69; Horn 1888: 34.
Eustrophinus indistinctus (LeConte).— Leng 1920: 238; Csiki 1924: 8; LeSage 1991: 246; Poole and Gentili 1996: 299.
Eustrophopsis indistinctus (LeConte).—Pollock 2008: 290.

##### Diagnosis.

This is the only species of *Eustrophopsis* with a distinctly brown dorsal color; other diagnostic features include: antennomeres 1–4 and 11 not very distinctly contrasting in color with 5–10; eyes distinctly separated dorsally; pronotal punctation (especially compared with *Eustrophopsis bicolor*) relatively coarse.

##### Description.

TL 4.9–6.2 mm; GEW 2.4–3.0 mm. Body elongate oval (Fig. 8), moderately tapered posteriorly, distinctly convex dorsally (Fig. 21); dorsal color light to dark brown, vestiture also brownish; antennomeres 1–4 and 11 light to dark rufous, antennomeres 5–10 dark, piceous; venter rufous to brown, legs somewhat lighter in color than that of ventral sclerites; eyes moderately widely separated dorsally (Fig. 33), distance between eyes greater than maximum width of antennomere 1; eyes deeply emarginated around antennal insertions; antennae relatively long, with no discernible sexual dimorphism; all antennomeres longer than wide; antennomeres 5–10 slightly widened, submoniliform; antennal sensilla completely annular; last maxillary palpomere elongate, unmodified, apex slightly oblique; pronotal punctation moderately coarse; prosternal process (Fig. 45) elongate, narrowly rounded distally, extended to slightly anterior of posterior margin of procoxae; prothoracic episternal suture present, surface of proepisternum smooth, punctures not obscured by rugose macrosculpture; coarse elytral punctures forming multiple striae; meso- and metatibiae with numerous, oblique ridges; male with small, ovate, setiferous pit on ventral edge of profemur; aedeagus (Figures 59, 64) with basal piece of tegmen distinctly longer than apical piece; struts on median lobe wide, long, inner margins V-shaped; sternite 9 basally broadly U-shaped.

##### Distribution

(Figures 71, 76). The range of this species is decidedly southwestern with most specimens from Arizona and New Mexico; southeastern Colorado is the northern extent. To the south, the species is known from northwestern Mexico. The 295 specimens examined were from the following jurisdictions: **MEXICO**: BAJA CALIFORNIA SUR, CHIHUAHUA. **UNITED STATES**: ARIZONA: Cochise, Gila, Graham, Greenlee, Maricopa, Pima, Pinal, Santa Cruz, Yavapai. COLORADO: Bent. NEW MEXICO: Bernalillo, Eddy, Roosevelt, Sierra. OKLAHOMA: Cimarron. TEXAS: Aransas, Blanco, Brewster, Cameron, Harris. (Complete label data given in Appendix 1).

##### Types.

*Eustrophus indistinctus* LeConte. SYNTYPE, sex unknown, labeled “gold disk / Type 4779 / Eustrophus indistinctus Lec.” Specimen with gold disk (indicative of California); the “Colorado” referred to in the description is actually the Colorado River, and not the state.

##### Natural history.

Fungi (AZ); on bracket fungus (AZ); ex fungus on dying willow (NM); on dead log at night (NM); at night on fungusy logs (NM); at fungi on burned *Populus* snags (NM); fungi on *Salix* (AZ).

##### Notes.

This species was one of the earliest described Nearctic eustrophines; however, it was synonymized early on with *Eustrophopsis bicolor* by Horn (1888: 34) who stated that “specimens collected by me in very early spring, in Arizona, have a decidedly brownish color above...These are probably merely less mature specimens as no other structural differences have been observed”. This synonymy was followed by subsequent authors, e.g. Csiki (1924) and Poole and Gentili (1996). In fact, the relatively lighter coloration is one of the diagnostic features of adults of *Eustrophopsis indistinctus*. Therefore, *Eustrophopsis indistinctus* has been removed from synonymy with *Eustrophopsis bicolor* and re-established as a distinct species. There remains some difficulty in distinguishing southern specimens of *Eustrophopsis bicolor* from *Eustrophopsis indistinctus* (see “notes” for *Eustrophopsis bicolor*, above).

#### Eustrophopsis arizonensis

(Horn, 1888)

http://species-id.net/wiki/Eustrophopsis_arizonensis

**Figures 9–13.**
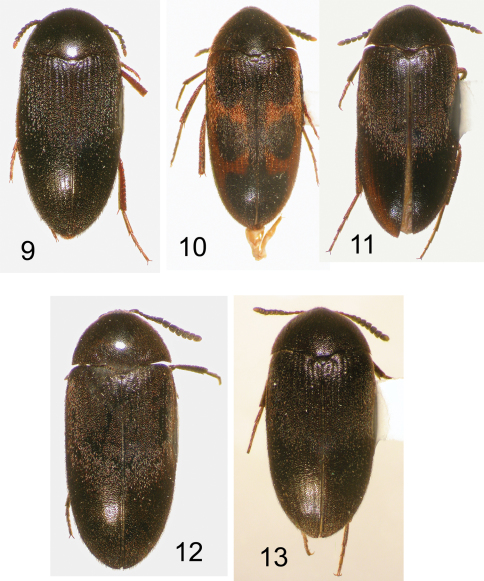
Nearctic Eustrophinae, dorsal habitus. **9** *Eustrophopsis arizonensis*, AZ: Coconino Co., TL = 7.2 mm **10** *Eustrophopsis ornatus* (typical form), Mexico: Chihuahua, TL = 7.3 mm **11** *Eustrophopsis ornatus* (dark form), AZ: Cochise Co., TL = 7.2 mm **12** *Eustrophopsis crowdyi*, sp. n. AZ: Cochise Co., TL = 7.4 mm **13** *Synstrophus repandus*, MB: Lockport, TL = 5.9 mm.

Figures 9 22 34 46 52 60 72 76

Eustrophus arizonensis Horn 1888: 34.—(“occurs in Arizona and New Mexico”); Champion 1889: 75; Henshaw 1889: 131; Horn 1894: 353; Wickham 1895: 168; Champion 1898: 65; Griffith 1900: 570.
Eustrophinus arizonensis (Horn).— Leng 1920: 238; Csiki 1924: 8; Blackwelder 1945: 495; Poole and Gentili 1996: 299.
Eustrophopsis arizonensis (Horn).—Pollock 2008: 290.

##### Diagnosis.

This distinctive species may be diagnosed on the following combination of features: eyes moderately widely separated; prothoracic episterna with distinctly rugose macrosculpture; meso- and metatibiae with oblique ridges; distribution: western United States.

##### Description.

TL 5.8–7.8 mm; GEW 2.6–3.6 mm. Body ovate (Fig. 9), slightly tapered posteriorly, moderately convex dorsally (Fig. 22); dorsal color uniform, piceous to near black; ventral color lighter than dorsal: venter rufous; legs, mouthparts and antennomeres 1–4 and 11 light to dark rufous; dorsal vestiture uniform, setae relatively short; eyes moderately widely separated dorsally (Fig. 34), distance between eyes greater than maximum width of antennomere 1; antennomeres 5–10 moderately wide, submoniliform, antennal sensilla annular; antennomeres 5–10 of male not flattened ventrally, without “accessory” setae; last maxillary palpomere somewhat widened distally, apex oblique; prosternal process (Fig. 46) acute, narrowly rounded distally, not reaching posterior margin of procoxae; coarse elytral punctation forming longitudinal striae; prothoracic episternal suture present, punctation of surface of proepisternum anterior of suture somewhat obscured by very coarse, rugose macrosculpturing; meso- and metatibiae with multiple, oblique ridges; male with small, ovate, setiferous pit on ventral edge of profemur; aedeagus (Fig. 60) with basal piece of tegmen slightly longer than apical piece; struts on median lobe elongate, inner margins subparallel to elongate oval; sternite 9 basally V-shaped.

**Figures 14–19.**
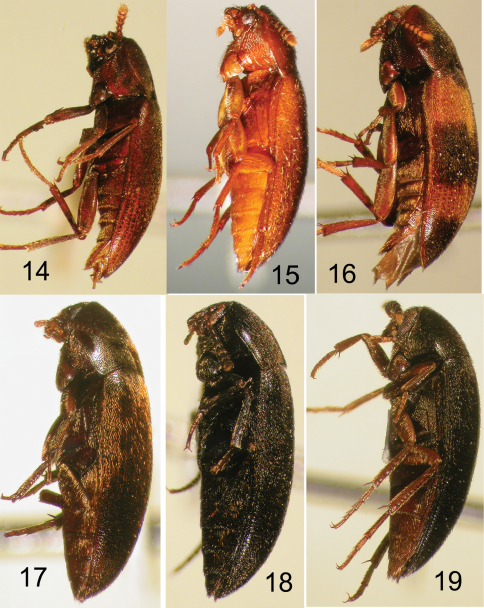
Nearctic Eustrophinae, left lateral view. **14** *Pseudoholostrophus impressicollis* **15** *Pseudoholostrophus discolor* **16** *Holostrophus bifasciatus* **17** *Eustrophus tomentosus* **18** *Eustrophopsis confinis* **19** *Eustrophopsis bicolor*.

**Figures 20–25.**
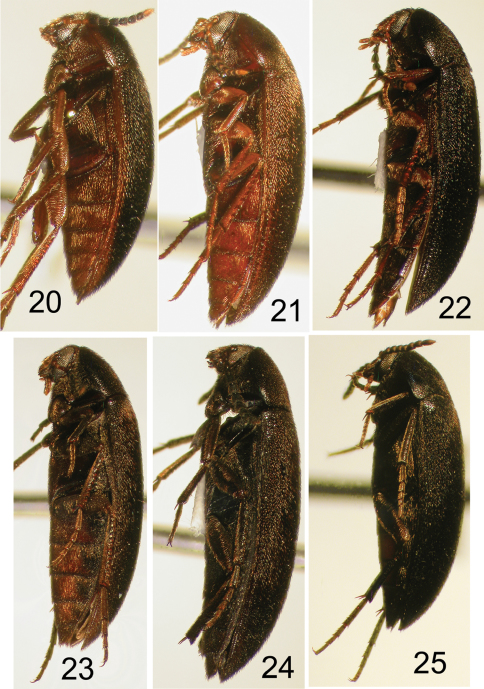
Nearctic Eustrophinae, left lateral view. **20** *Eustrophopsis brunneimarginatus* **21** *Eustrophopsis indistinctus* **22** *Eustrophopsis arizonensis* **23** *Eustrophopsis ornatus* (dark form) **24** *Eustrophopsis crowdyi*, sp. n. **25** *Synstrophus repandus*.

**Figures 26–28.**
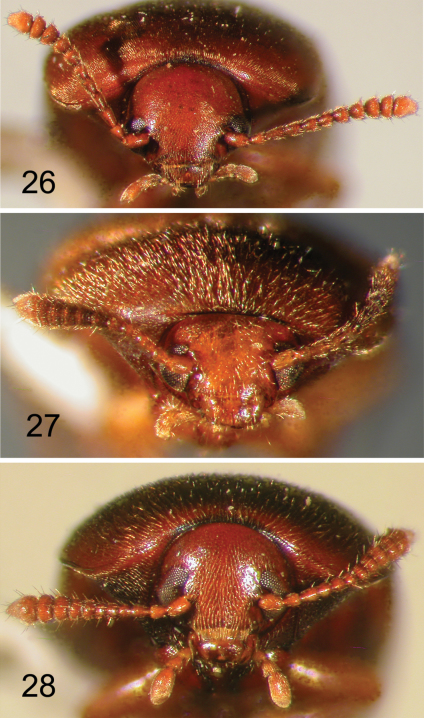
Nearctic Eustrophinae, frontal view of head. **26** *Pseudoholostrophus impressicollis* **27** *Pseudoholostrophus discolor* **28** *Holostrophus bifasciatus*.

**Figures 29–31.**
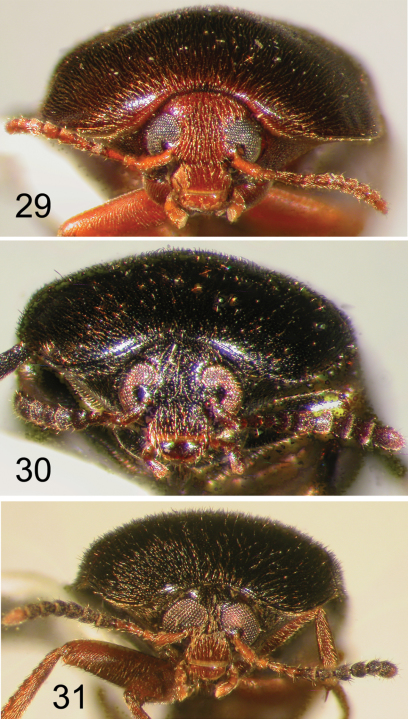
Nearctic Eustrophinae, frontal view of head. **29** *Eustrophus tomentosus* **30** *Eustrophopsis confinis* **31** *Eustrophopsis bicolor*.

**Figures 32–34.**
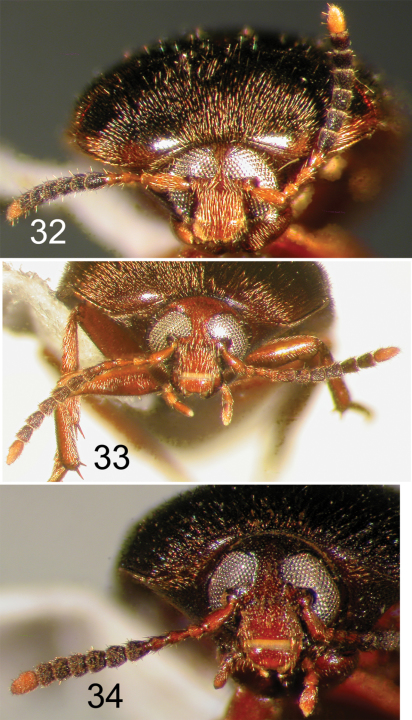
Nearctic Eustrophinae, frontal view of head. **32** *Eustrophopsis brunneimarginatus* **33** *Eustrophopsis indistinctus* **34** *Eustrophopsis arizonensis*.

##### Distribution

(Figures 72, 76). *Eustrophopsis arizonensis* is one of the most widespread species in the subfamily, when the Mexican distribution is included. Specimens are known from northern United States (southeast Montana) south to Oaxaca, Mexico. The 516 individuals examined are from the following: **MEXICO**: DURANGO, MORELOS, OAXACA, SONORA. **UNITED STATES**: ARIZONA: Apache, Cochise, Coconino, Gila, Graham, Navajo, Pima, Pinal, Santa Cruz, Yavapai. CALIFORNIA: Riverside, San Bernardino. COLORADO: Douglas, La Plata. MONTANA: Powder River. NEW MEXICO: Catron, Cibola, Grant, Hidalgo, Lincoln, Los Alamos, San Miguel, Torrance. SOUTH DAKOTA: Fall River. TEXAS: Brewster, Jeff Davis. UTAH: Washington. (Complete label data given in Appendix 1).

##### Types.

*Eustrophus arizonensis* Horn. LECTOTYPE, sex unknown, labeled “Ariz / LectoTYPE 8038 / E. arizonensis Horn / MCZ Type 34038", in MCZ. Paralectotypes (3) in MCZ.

##### Natural history.

Label data: under bark, *Quercus* (MX), under bark (AZ, MX), under pine bark (AZ), ex. fungi (AZ), dead oak at night (AZ), under log (AZ), pine stump (CA).

##### Notes.

The rugose nature of the prothoracic episterna of this species seems to be unique in the genus, perhaps in the entire subfamily. The Mexican specimens have been included in *Eustrophopsis arizonensis*, based on possession of this feature.

#### Eustrophopsis ornatus

(Van Dyke, 1928)

http://species-id.net/wiki/Eustrophopsis_ornatus

Figures 10–11 23 35 47 61 73 76

Eustrophinus ornatus Van Dyke 1928: 251.—U.S.A., Arizona, Cochise Co., Chiricahua Mts., near Cave Creek; Leng and Mutchler 1933: 36; Poole and Gentili 1996: 299.
Eustrophopsis ornatus (Van Dyke).—Pollock 2008: 290.

##### Diagnosis.

Individuals of *Eustrophopsis ornatus* share the following diagnostic features: antennomeres 5–10 distinctly widened, in males flattened on one side and with elongate, accessory setae; most specimens with at least faint indication of light elytral markings (seen in their maximum extent in Fig. 10). This species is a putative close relative of *Eustrophopsis crowdyi*; males of the two species can be separated on the elytral color. However, non-maculate, dark males of *Eustrophopsis ornatus* have elongate antennal sensilla, whereas males of *Eustrophopsis crowdyi* have short sensilla. As well, the prosternal process of *Eustrophopsis crowdyi* is relatively blunter and more rounded than that of *Eustrophopsis ornatus*. Finally, specimens of *Eustrophopsis ornatus* tend to have antennae with contrastingly colored antennomeres, while antennomeres of *Eustrophopsis crowdyi* are more or less similar in color.

##### Description.

**Figures 35–37.**
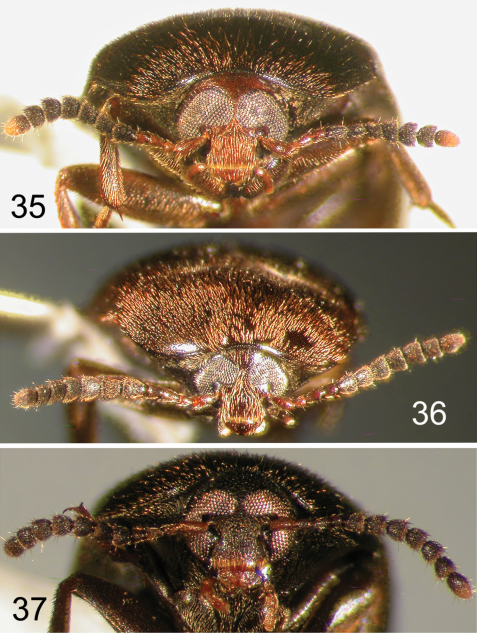
Nearctic Eustrophinae, frontal view of head. **35** *Eustrophopsis ornatus* **36** *Eustrophopsis crowdyi*, sp. n. **37** *Synstrophus repandus*.

TL 5.3–7.6 mm; GEW 2.3–3.3 mm. Body elongate oval, relatively narrow, moderately tapered posteriorly (Figures 10–11), moderately convex dorsally (Fig. 23); dorsal color various, some specimens uniformly dark, piceous to near black (Fig. 11); other specimens with very faint indications of light(er) elytral markings; some specimens (typical form) with distinct rufous elytral markings, along lateral margin and encroaching upon disc (Fig. 10); ventral surface in most specimens at least slightly lighter in color than dorsum; antennomeres 1–4 dark rufous; at least the distal half of antennomere 11 in most specimens distinctly contrasting in color, orange-red; eyes approximate dorsally (Fig. 35), either touching, or separated by a distance equal to 1–2 facet diameters; antennomeres 5–10 distinctly widened in both sexes, subquadrate, wider than long; antennomeres 5–11 of male with ventral sides flattened, with long, conspicuous “accessory” setae (sensilla) on ventral surface; last maxillary palpomere not widened distally, apex slightly oblique; prosternal process acute, narrowly rounded distally, not attaining posterior margin of procoxae; coarse elytral punctures forming striae; prothoracic episternal suture present, surface of proepisternum smooth, punctation not obscured by rugose macrosculpture; meso- and metatibiae with numerous, oblique ridges; male with small, ovate, setiferous pit on ventral edge of profemur; aedeagus (Fig. 61) with apical piece of tegmen longer than basal piece; struts on median lobe short, narrow, inner margins broadly U-shaped; sternite 9 basally V-shaped.

**Figures 38–43.**
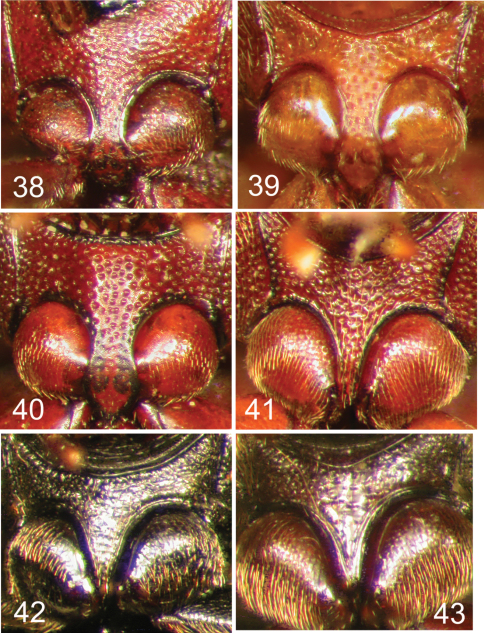
Nearctic Eustrophinae, prosternal process. **38** *Pseudoholostrophus impressicollis* **39** *Pseudoholostrophus discolor* **40** *Holostrophus bifasciatus* **41** *Eustrophus tomentosus* **42** *Eustrophopsis confinis* **43** *Eustrophopsis bicolor*.

**Figures 44–49.**
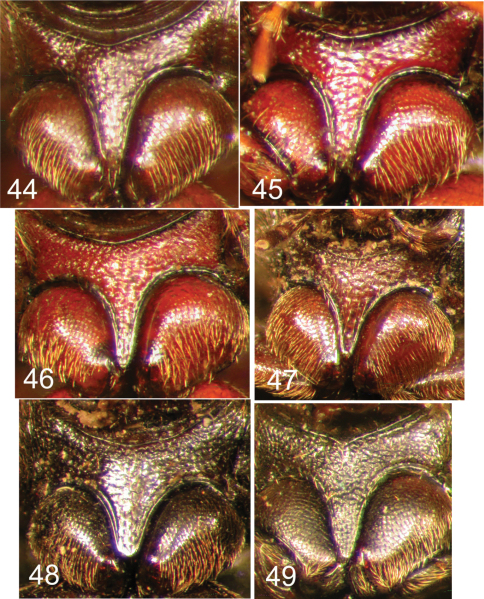
Nearctic Eustrophinae, prosternal process. **44** *Eustrophopsis brunneimarginatus* **45** *Eustrophopsis indistinctus* **46** *Eustrophopsis arizonensis* **47** *Eustrophopsis ornatus* **48** *Eustrophopsis crowdyi*, sp. n. **49** *Synstrophus repandus*.

**Figures 50–53.**
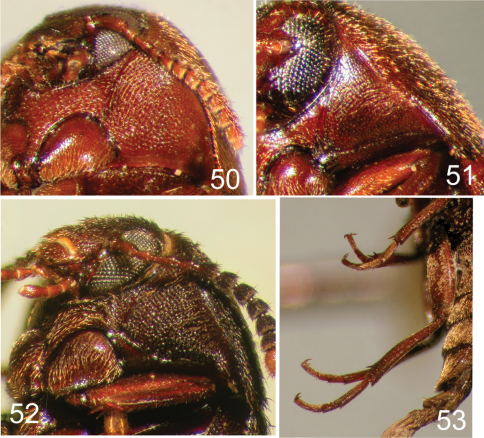
Prothorax (**50–52**) and hind leg (**53**) of selected Nearctic Eustrophinae. **50** *Eustrophus tomentosus* **51** *Eustrophopsis indistinctus* **52** *Eustrophopsis arizonensis* **53** *Pseudoholostrophus impressicollis*.

**Figures 54–55.**
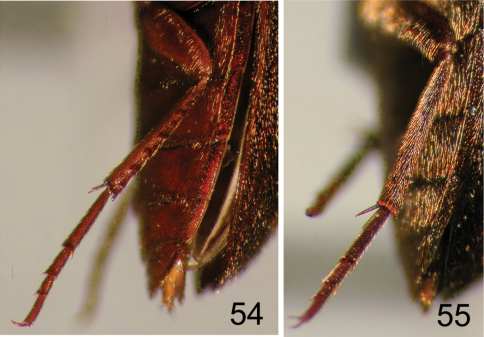
Hind leg of selected Nearctic Eustrophinae. **54** *Eustrophopsis indistinctus* **55** *Synstrophus repandus*.

**Figures 56–61.**
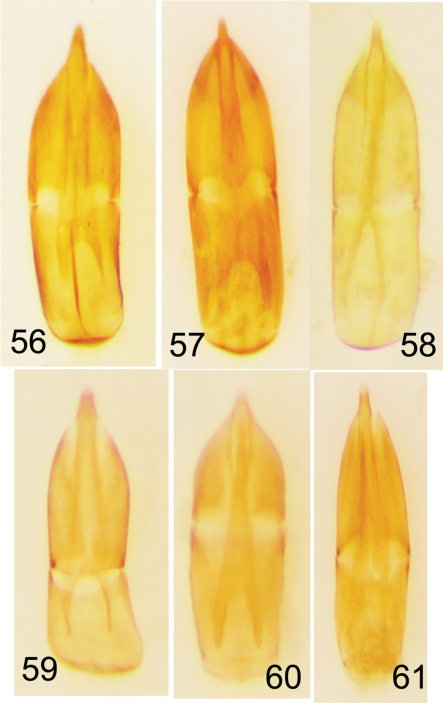
Aedeagi of Nearctic *Eustrophopsis* spp. **56** *Eustrophopsis confinis* **57** *Eustrophopsis bicolor* **58** *Eustrophopsis brunneimarginatus* **59** *Eustrophopsis indistinctus* **60** *Eustrophopsis arizonensis* **61** *Eustrophopsis ornatus*.

##### Distribution

(Figures 73, 76).The Nearctic distribution of this species is restricted to southernmost Arizona and New Mexico; the range also extends south into Mexico. It is perhaps more widespread in Mexico and Central America, with the U.S. localities representing the northern extent of its range. The 132 specimens are from the following jurisdictions: **MEXICO**: JALISCO, SONORA. **UNITED STATES**: ARIZONA: Cochise, Pima, Santa Cruz. NEW MEXICO: Luna. (Complete label data given in Appendix 1).

##### Types.

*Eustrophinus ornatus* Van Dyke. HOLOTYPE, sex unknown, labeled “Chiricahua Mts. Ariz 7000ft. June 21, 1927 / Cave Creek Cochise Co. / J.A. Kusche Collector / Van Dyke Collection / Holotype Eustrophinus ornatus Van Dyke / California Academy of Sciences Type No. 2581", in CASC.

##### Natural history.

Label data: under dead pine bark (AZ); WPB-baited Lindgren funnel trap (AZ); at UV light (AZ).

##### Notes.

Van Dyke described this species as “*ornatus*” due to the presence of distinctive, lighter colored markings on the dark elytra. In fact, of all specimens of *Eustrophopsis ornatus* examined for this study (other than the types), only very few specimens exhibited this “typical” coloration. Many specimens had only a very faint indication of the lighter coloration, while others were entirely dark. The remarkably modified male antennomeres of this species and *Eustrophopsis crowdyi* is an indication of their possible close relationship.

#### Eustrophopsis crowdyi sp. n.

urn:lsid:zoobank.org:act:309E2731-414C-4F7D-8B73-7D2F86D30F4D

http://species-id.net/wiki/Eustrophopsis_crowdyi

Figures 12 24 36 48 62 74

##### Types.

HOLOTYPE, male, labeled: "AZ, Cochise Co., Turkey Crk, 31°51.280'N, 109°19.883'W, 14.vii.02 ex. WPB-baited Lindgren funnel trap, ca. 2000 m. / HOLOTYPE ♂ *Eustrophopsis crowdyi* Pollock" (in USNM). ALLOTYPE, female, with same label data as holotype, except “ALLOTYPE ♀ *Eustrophopsis crowdyi* Pollock" (in USNM). Four male and 2 female paratypes, same label data as holotype; 5 male and 4 female paratypes, labeled: “AZ, Cochise Co., Turkey Crk, 31°51.280'N, 109°19.883'W, 7.vii.02 ex. WPB-baited Lindgren funnel trap, ca. 2000m. / near XPB-infested Chihuahua pine B. Fitzgibbon, coll."; 1 male and 1 female paratypes, labeled: “USA, AZ, Apache Co., Apache N.F., Luna Lk cmpgrd, ca. 5 mi. E. Alpine, 33°50'04"N, 109°05'03"W; 22.vi.2002 / collected at night under ponderosa pine bark, 7960ft. D.A. Pollock". (Paratypes in CASC, CUIC, DAPC, MTEC, UAIC, USNM)

##### Diagnosis.

Individuals of *Eustrophopsis crowdyi* may be diagnosed from other species of *Eustrophopsis* on the following characters: overall dark body color; eyes not approximate dorsally; males with short antennal sensilla; prosternal process rather blunt, rounded distally. As mentioned above for *Eustrophopsis ornatus*, that species and *Eustrophopsis crowdyi* are thought to be close relatives, based on the modified antennomeres of the males.

##### Description.

TL 5.6–7.9 mm; GEW 2.5–3.5 mm. Body elongate oval, relatively narrow, moderately tapered posteriorly (Fig. 12), moderately convex dorsally (Fig. 24); entire body piceous to nearly black, except for antennomeres 1–4 and tip of antennomere 11 somewhat lighter in color, very dark rufous.

Head with uniformly spaced, relatively coarse punctation; eyes (Fig. 36) deeply emarginated around antennal insertions, separated dorsally by distance approximately equal to maximum width of first antennomere; antennomeres 5–10 (Fig. 36) distinctly widened in both sexes, and with different microsculpturing than antennomeres 1–4; antennomeres 7–10 subquadrate, wider than long; male with antennomeres 5–11 flattened on ventral side, ventral surface of antennomeres 5–10 with short, slender “accessory” setae (sensilla); distal maxillary palpomere not widened distally, subparallel-sided, apex slightly oblique.

Pronotum with uniform, shallow punctation and short, apressed brownish setae; posterior margin with two clusters of coarser punctures, equidistant between middle and lateral margins; posterior margin of pronotum bisinuate, inner sinuation deeper than outer; bead distinct along lateral, anterior, and posterior margins, somewhat obscured on middle lobe of posterior margin; prothoracic episternal suture present; surface of proepisterna smooth, regularly punctuate, without coarse or rugose macrosculpture; prosternal process (Fig. 48) moderately elongate, not attaining posterior margin of procoxae, apex moderately broadly rounded; mesosternum short, distinctly keeled.

Elytra elongate, slightly convex; punctation of two types: coarse punctures forming 10 striae on each elytron; other punctures finer, setose, setae uniformly distributed, decumbent; epipleuron distinct, narrowed posteriorly, but traceable to elytral apex; flight wings full, functional, membrane darkly pigmented.

Legs all relatively similar in shape; metathoracic legs more elongate than meso- or prothoracic legs; all tarsi cylindrical, without ventral lobes; outer surfaces of meso- and metatibiae with numerous, oblique ridges; tibial spurs distinct, paired; tarsal claws slender, without basal tooth or expansion; male profemur with small, ovate, setiferous pit on ventral surface, approximately ¼ to 1/3 distance from base of femur.

**Figures 62–64.**
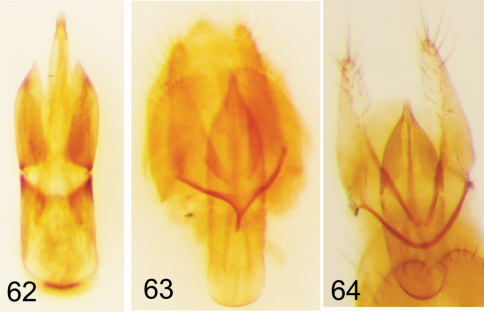
Aedeagus (**62**) and genital segments (**63–64**) of Nearctic *Eustrophopsis* spp. **62** *Eustrophopsis crowdyi* **63** *Eustrophopsis bicolor* **64** *Eustrophopsis indistinctus*.

Abdominal ventrites with uniform, relatively coarse punctation, setae decumbent; male genitalia darkly pigmented (Fig. 62) with apical and basal piece of tegmen subequal in length; struts on median lobe elongate, relatively narrow, inner margins V-shaped; sternite 9 basally Y-shaped, with long stem.

**Figure 65.**
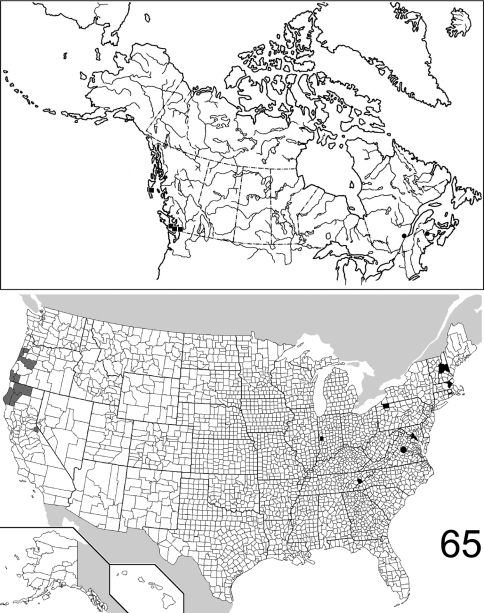
Known distributions of *Pseudoholostrophus impressicollis* (■ – Canada; grey shading – United States) and *Pseudoholostrophus discolor* (● – Canada; black shading – United States; dot represents state record)

**Figure 66.**
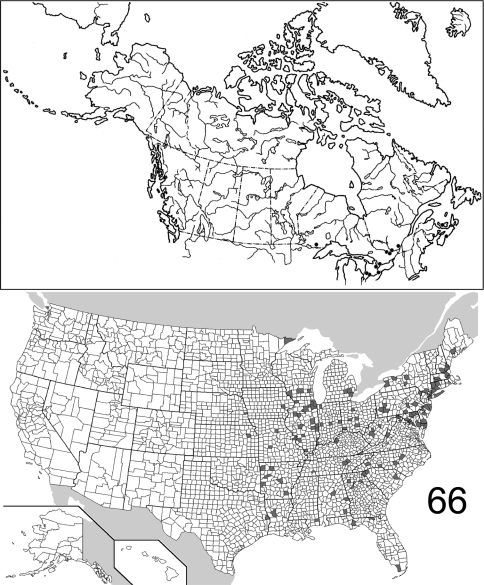
Known distribution of *Holostrophus bifasciatus*.

**Figure 67.**
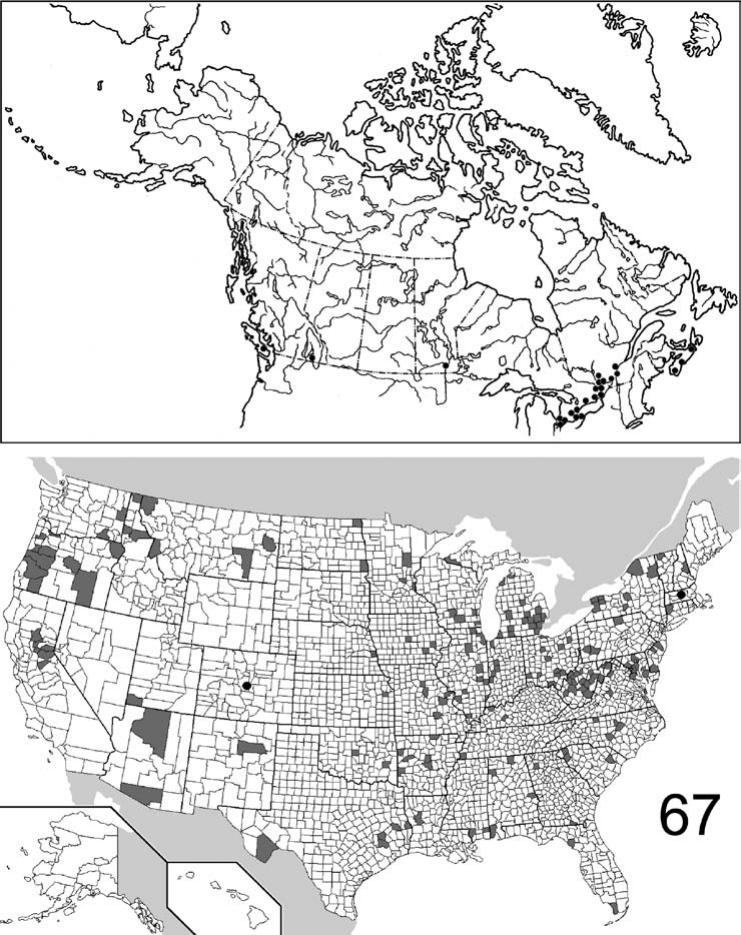
Known distribution of *Eustrophus tomentosus* (dots represent state records).

**Figure 68.**
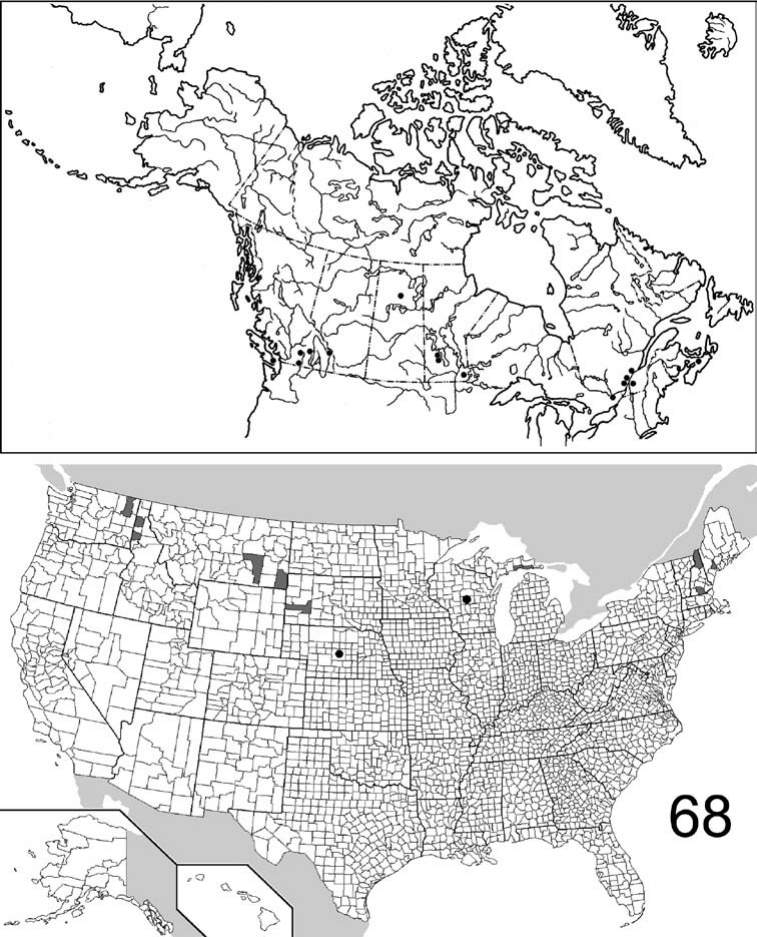
Known distribution of *Eustrophopsis confinis* (dots represent state records).

##### Distribution

(Fig. 74). This species is known only from the type locality, in southeastern Arizona. **UNITED STATES**: ARIZONA: Cochise.

##### Natural history.

All known specimens, except two, were collected using Lindgren funnel traps baited with western pine beetle (Curculionidae: Scolytinae) attractant; some specimens were labeled as having been collected near a scolytine-infested Chihuahua pine (*Pinus leiophylla*). Two specimens were collected at night under ponderosa pine bark.

##### Derivation of specific epithet.

I am very pleased to be able to name this new species after my oldest son, George “Crowdy” Pollock, who has accompanied me on many collecting expeditions and who has made many interesting discoveries along the way. In fact, he found larvae and adults of *Eustrophopsis indistinctus* in a neighbor’s fungus-colonized birch (*Betula*) stump, which were the first specimens of that species I had seen from New Mexico.

##### Notes.

*Eustrophopsis crowdyi* shares with *Eustrophopsis ornatus* conspicuously sexually dimorphic antennae, in males with a widening of antennomeres, one side of which is flattened and with setiform sensilla. While certainly not all world species of Eustrophinae have been studied in detail, this antennal modification is unique (so far) to these two species. As mentioned above, specimens of *Eustrophopsis crowdyi* were collected with many specimens of *Eustrophopsis arizonensis* and *Eustrophopsis ornatus* at the same locality in Cochise County, Arizona.

#### Synstrophus

Seidlitz 1898

http://species-id.net/wiki/Synstrophus

Synstrophus Seidlitz 1898: 438.—Type species: *Eustrophus macrophthalmus*Reitter 1877 (orig. descr.); Leng 1920: 238; Hatch 1965: 66; Nikitsky 1989: 18; LeSage 1991: 246; Nikitsky 1992a: 435; Poole and Gentili 1996: 300; Nikitsky 1998: 58; Young and Pollock 2002: 416; Nikitsky 2008: 63; Pollock 2008: 282.

##### Note.

There are five species of *Synstrophus* (Nikitsky 1998), distributed in China, Japan, Oriental region, and North America. Nikitsky (1998) mentioned that based on several characters, *Synstrophus repandus* (Horn) might eventually merit placement in a different genus-group taxon. A single species of *Synstrophus* is represented among Nearctic Eustrophinae.

#### Synstrophus repandus

(Horn, 1888)

http://species-id.net/wiki/Synstrophus_repandus

Figures 13 25 37 49 55 75

Eustrophus repandus Horn 1888: 33.—U.S.A., Pennsylvania (“Its distribution seems to be across the northern half of our country from Canada and New Hampshire to Virginia, and from these points through all the States to the Pacific coast as far south as the extreme north of California”); Henshaw 1889: 131; Champion 1898: 65.
Synstrophus repandus (Horn).—Leng 1920: 238; Csiki 1924: 9; Hatch 1965: 66, Plate VIII, fig. 9; LeSage 1991: 246; Poole and Gentili 1996: 300; Young and Pollock 2002: 416; Pollock 2008: 282; Majka and Pollock 2010: 455.

##### Diagnosis.

This species is easily diagnosed based on the following features: body color uniformly dark; eyes narrowly separated; meso- and metatibiae smooth, without oblique ridges.

##### Description

(from Pollock 2008: 282). TL 5.6–7.0 mm; GEW 2.5–3.4 mm. Body ovate (Fig. 13), only somewhat tapered posteriorly, distinctly convex dorsally (Fig. 25); dorsal color dark piceous to black; antennae with basal 4 antennomeres dark rufous, distal half of antennomere 11 rufous; venter at least slightly lighter in color than dorsum; mouthparts similar in color to antennomeres 1–4; legs and abdominal ventrites dark rufous to piceous; dorsal pubescence relatively long, conspicuous; eyes narrowly separated (Fig. 37), or almost contiguous (space < length of antennomere 1), medial margin moderately emarginate; antennomeres 2–4 short, submoniliform; antennomeres 5–10 widened, becoming more triangular toward antennomeres 9–10; antennal sensilla completely annular; last maxillary palpomere unmodified, fusiform; prosternal process (Fig. 49) triangular, narrowed distally, extended to slightly short of posterior margin of procoxae; prothoracic episternal suture absent; elytral punctation coarse, punctures arranged in longitudinal striae; meso- and metatibiae with scattered short spines, but distinct ridges absent; male lacking setiferous pit on ventral edge of profemur.

##### Distribution

(Fig. 75). Another widely distributed species, *Synstrophus repandus* exhibits a transcontinental range, similar to that of *Eustrophus tomentosus*. Most records are eastern, with a general gap in the interior of the continent, but with records from British Columbia, Pacific Northwest, and California. The 1,175 examined specimens are from the following jurisdictions: **CANADA**: BRITISH COLUMBIA, MANITOBA, ONTARIO, QUEBEC. **UNITED STATES**: ALABAMA: Bibb, Green, Lee, Madison, Mobile, Shelby. ARKANSAS: Faulkner, Franklin, Fulton, Hempstead, Pulaski. CALIFORNIA: Riverside, Shasta, Siskiyou. CONNECTICUT: Litchfield, New Haven. DISTRICT OF COLUMBIA. DELAWARE: New Castle. FLORIDA: Alachua, Clay, Franklin, Jackson, Liberty, Leon, Orange, Wakulla. GEORGIA: Baker, Bibb, Bulloch, Charlton, Chattahoochee, Clarke, Coweta, DeKalb, Floyd, Fulton, Greene, Henry, Oglethorpe, Paulding, Whitfield. IDAHO: Bonner, Kootenai. ILLINOIS: Alexander, Champaign, Clark, Coles, Cook, DeKalb, Edgar, Jackson, Jasper, LaSalle, Ogle, Piatt, St. Clair, Sangamon, Union, Vermilion, Wabash. INDIANA: Clinton, LaPorte, Monroe, Parke, Porter, Putnam, Spencer, Tippecanoe, Vigo. IOWA: Dickinson, Story. KANSAS. KENTUCKY: Bath, Henderson. LOUISIANA: East Baton Rouge, Rapides, Washington. MAINE: Androscoggin, Cumberland, Kennebec, York. MARYLAND: Anne Arundel, Calvert, Cecil, Montgomery, Prince George’s, Washington, Worcester. MASSACHUSETTS: Hampden, Hampshire, Middlesex. MICHIGAN: Alger, Allegan, Alpena, Arenac, Berrien, Charlevoix, Cheboygan, Eaton, Ingham, Lake, Lenawee, Livingston, Marquette, Midland, Oakland, Saginaw, Schoolcraft, Wayne. MINNESOTA: Crow Wing, Hennepin, Roseau, Sherborne, St. Louis. MISSISSIPPI: Adams, Lafayette, Perry, Stone. MISSOURI: Randolph, St. Charles, St. Louis. MONTANA: Flathead, Lake, Lincoln. NEBRASKA: Sarpy. NEW HAMPSHIRE: Carroll, Grafton, Rockingham, Strafford. NEW JERSEY: Bergen, Burlington, Monmouth. NEW YORK: Erie, Livingston, Monroe, Nassau, Niagara, Onondaga, St. Lawrence, Tompkins, Warren, Wayne, Wyoming. NORTH CAROLINA: Buncombe, Durham, Gaston, Henderson, Hertford, Jackson, Wake. NORTH DAKOTA: Bottineau, Cass, Richland. OHIO: Ashtabula, Butler, Clark, Clermont, Clinton, Cuyahoga, Delaware, Fairfield, Hocking, Jackson, Knox, Lake, Ross, Union, Vinton, Wayne. OKLAHOMA: Grady, Latimer, Pushmataha. OREGON: Douglas, Josephine, Klamath, Lane, Wasco. PENNSYLVANIA: Allegheny, Clearfield, Dauphin, Westmoreland, York. SOUTH CAROLINA: Abbeville, Beaufort, Florence, Lancaster, Pickens. SOUTH DAKOTA: Minnehaha, Yankton. TEXAS: Anderson, Angelina, Briscoe, Cass, Cherokee, Montgomery, Polk, Sabine, San Jacinto, Travis, Tyler, Walker. UTAH: Washington. VERMONT: Rutland. VIRGINIA: Chesterfield, Clarke, Essex, Fairfax, Falls Church, Lee, Loudoun. WASHINGTON: Whatcom. WEST VIRGINIA: Braxton, Greenbrier, Kanawha, Lewis, Mineral, Nicholas, Randolph. WISCONSIN: Bayfield, Dane, Grant, Iowa, Oconto, Racine, Vilas. WYOMING. (Complete label data given in Appendix 1).

**Figure 69.**
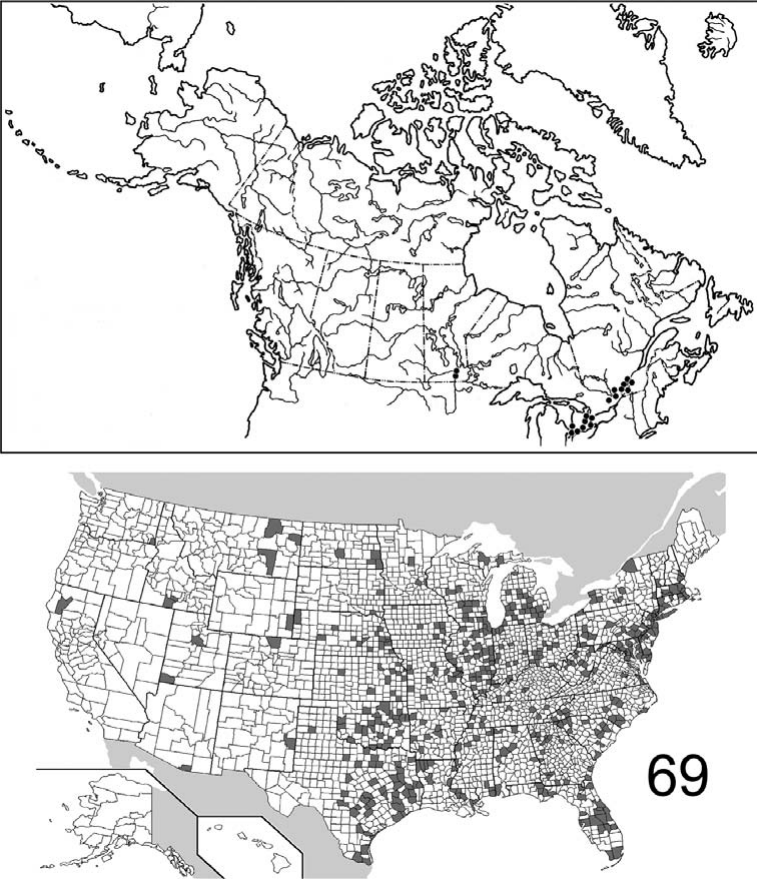
Known distribution of *Eustrophopsis bicolor*.

**Figure 70.**
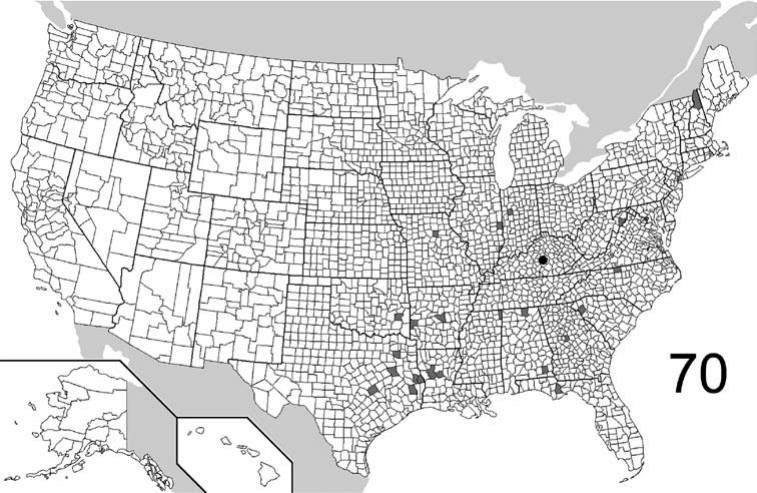
Known distribution of *Eustrophopsis brunneimarginatus* (dot represents state record).

**Figure 71.**
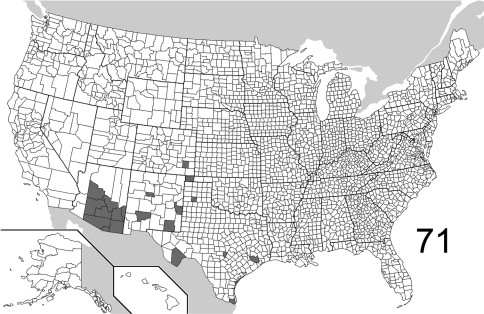
Known distribution of *Eustrophopsis indistinctus*.

**Figures 72–73.**
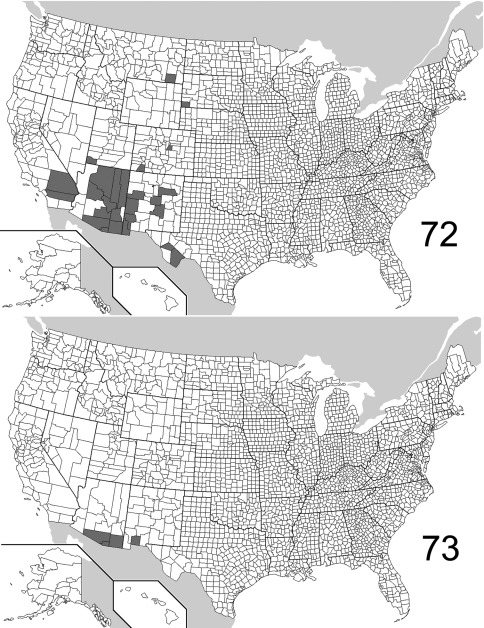
Known distribution of *Eustrophopsis arizonensis* (**72**) and *Eustrophopsis ornatus* (**73**).

**Figure 74.**
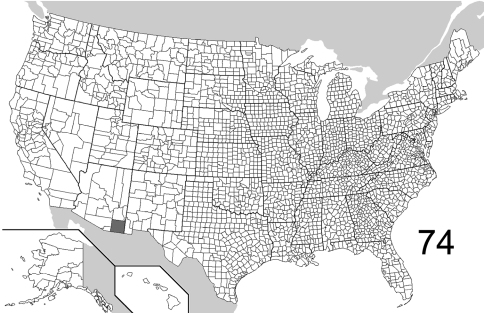
Known distribution of *Eustrophopsis crowdyi*, sp. n.

**Figure 75.**
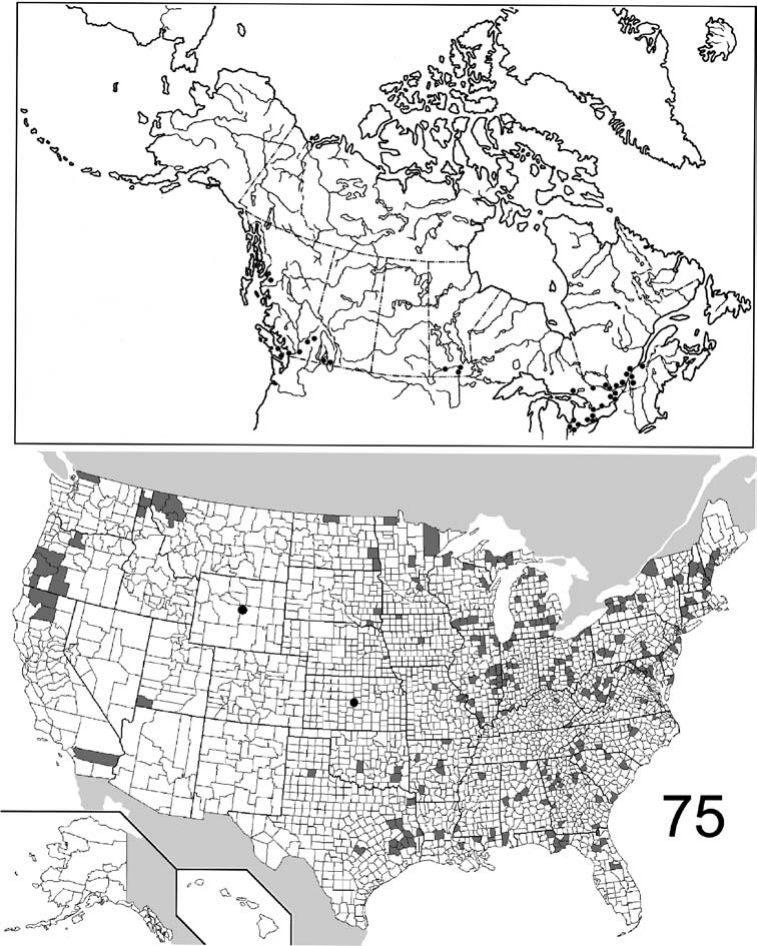
Known distribution of *Synstrophus repandus* (dots represent state records).

**Figure 76.**
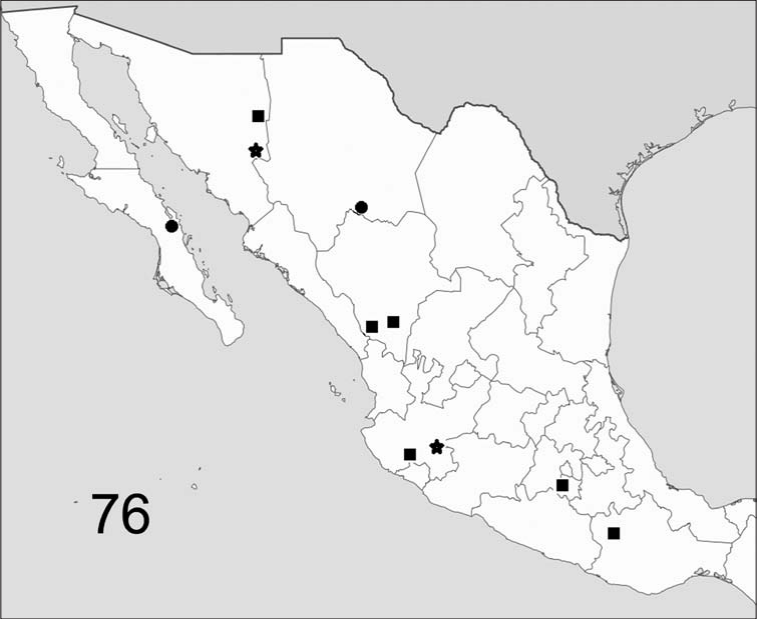
Known Mexican distributions of southern *Eustrophopsis* species: *Eustrophopsis arizonensis* (■) *Eustrophopsis indistinctus* (●) *Eustrophopsis ornatus* (★).

##### Types.

*Eustrophus repandus* Horn. LECTOTYPE, sex unknown, labeled “Pen / LectoTYPE / Eustrophus repandus Horn / MCZ Type 34039", in MCZ. Paralectotypes (3), in MCZ.

##### Natural history.

Label data: *Pinus ponderosa* bark (BC), shelf fungus on birch (BC), fungus on cottonwood (BC), *Populus trichocarpa* (BC), ex fungus on *Betula* (BC), underside of *Populus trichocarpa* log (BC), fleshy bracket fungus (ON), under wet moldy bark on dead tree (ON), *Pleurotus ostreatus* (IL, MA, QC), polypore on branch of old *Quercus rubrus* (QC), under dead pine bark (AL), *Meripilus giganteus* (AR), ex fungi and under bark (FL), large mushroom on log (IL), *Polyporus adustus* (IL), *Laetiporus sulphureus* (IL), under *Pinus* bark (LA), ex bracket fungi (NC), in slimy fungus under pine bark (NY), under dead bark (SC), ex. *Polyporus schweinitzii* (VT), under bark of pine (WY).

##### Notes.

Among specimens in the LeConte collection (MCZ) a specimen of *Synstrophus repandus* is labeled “Eustrophus concolor Linn.” This is the only known instance of this name to me, and it seems doubtful that it represents a described species.

## Discussion

The Eustrophinae is a good example of a group that is simultaneously rather obscure and poorly known to most coleopterists, and yet rather abundant, widespread and a conspicuous component of the saproxylic fauna associated with dead coarse, woody material. Saproxylic beetles are important members of the insect community associated with the dead tree habitat in most forest types; they can be associated with the dead wood itself, fruiting bodies of wood-decaying fungi, or with other saproxylic species (Hammond 1997). As indicated by Majka and Pollock (2006), few comprehensive studies have been done on the entirety of the saproxylic fauna of any region in North America. Saproxylic beetles undoubtedly have an important role in the ecology of forests, and likewise, forest structure and disturbance have a distinct effect on the presence (or absence) of saproxylics, including the Eustrophinae (Majka and Pollock 2006). Indeed, in forests where coarse woody material is allowed to persist (i.e., with limited management), eustrophines can be very common. However, this abundance can only be appreciated by collecting at night, on logs, snags or stumps infested by various groups of wood-rotting fungi. As well, Lindgren funnel traps have shown their value as collecting devices for very long series of eustrophines (see example in Introduction). Collecting on exposed surfaces during the day, or relying on black lights at night, will produce relatively few specimens; this is likely why there were relatively few very long series of specimens from single collecting efforts, seen among examined material for this study.

Biogeographically, the Nearctic fauna of Eustrophinae is an interesting combination of distribution patterns. Species of Holostrophini are either eastern or western; for example, individuals of *Pseudoholostrophus impressicollis* are restricted to westernmost North America, from southern British Columbia to California. Conversely, *Pseudoholostrophus discolor* is known only from northeastern regions. The range of *Holostrophus bifasciatus* is more extensive, but it is still restricted to the eastern United States and Canada; only a few records are known from west of the Mississippi River.

Specimens of *Eustrophus tomentosus* and *Synstrophus repandus* are among the most commonly collected in the subfamily, and have a transcontinental distribution with an interior gap. This relative paucity of specimens from the interior has much to do with lack of extensive forest habitats, except perhaps for forests associated with rivers. For example, a very large log along the Red River at Lockport, Manitoba yielded many specimens of *Synstrophus repandus* over a three-year period. This log was transported to its resting place from a location further south along the Red River during spring flooding. It is unknown whether the fungus (and consequent eustrophines) was post-depositional, or was initiated before the log rafted north. In any event, the relative shade and moisture of the riverbank allowed for the maintenance of the moisture critical to fungal growth and development of multiple generations of eustrophine beetles (both *Synstrophus repandus* and *Eustrophopsis bicolor*).

Species of *Eustrophopsis* exhibit a definite split between western and eastern faunas, except for *Eustrophopsis confinis*, which is the only species with a more or less continuous distribution across North America (admittedly, with relatively few known specimens); also, this species is restricted to northern latitudes, with specimens examined from more localities in Canada than the United States. The most common species of eustrophine, *Eustrophopsis bicolor*, is very widespread east of the central plains states, with only scattered records from the western United States. In Canada, no records are known from west of Manitoba. Another eastern species is *Eustrophopsis brunneimarginatus*, which seems restricted to the southeastern United States, west to south-central Texas.

The most “complex” region for *Eustrophopsis* is the southwestern United States, especially Arizona. The forested mountains and canyons in southern Arizona represent a zone of sympatry for five of the seven species of Nearctic *Eustrophopsis*. Among these five, relatively few specimens of *Eustrophopsis bicolor* are known from the southwest. The vast majority of specimens of another western species, *Eustrophopsis arizonensis*, are known from Arizona and New Mexico, but specimens are also known from as far north as southeastern Montana. This species also has a Mexican range as far south as Oaxaca. Specimens of *Eustrophopsis indistinctus* are known mainly from New Mexico and Arizona, with a northern range limit in southeastern Colorado, and southern limit in Mexico. The two remaining species of *Eustrophopsis*: *Eustrophopsis ornatus* and *Eustrophopsis crowdyi*, are known only from southernmost Arizona and New Mexico, with *Eustrophopsis ornatus* extending south into Mexico as well. This preliminary analysis of Eustrophinae biogeography suggests that the Nearctic fauna is a mixture of elements with close relatives in Europe and/or Asia (e.g. Holostrophini, *Eustrophus*, and *Synstrophus*) or in the Neotropics (e.g. some southwestern U.S. *Eustrophopsis*).

While most genera of the Eustrophinae are relatively well known taxonomically (e.g. *Pseudoholostrophus*, *Holostrophus*, *Eustrophus*, and *Synstrophus*), the one group in need of detailed revisionary work is *Eustrophopsis*. Many species have been described from Neotropical and Afrotropical regions (including Madagascar), with more undoubtedly remaining to be discovered and/or described. This represents a complex biogeographic scenario, and at this point it is unknown (but perhaps doubtful) whether *Eustrophopsis* is a monophyletic taxon.

The seeming ecological importance of this group of tenebrionoid beetles, combined with the relatively high species diversity (especially in the tropics) make the Eustrophinae a group deserving of further research. Future work on the group should concentrate not only on the nomenclature and taxonomy of adults, but also on the collection and description of immature stages, and possible host preferences / associations between the eustrophines and the wood-decaying fungi in which they occur. This will contribute not only to a better, more robust classification of the Tetratomidae, but also to the overall phylogeny and evolution of the “lower Tenebrionoidea”.

## Supplementary Material

XML Treatment for Pseudoholostrophus

XML Treatment for Pseudoholostrophus (Pseudoholostrophus) impressicollis

XML Treatment for Holostrophinus

XML Treatment for Pseudoholostrophus (Holostrophinus) discolor

XML Treatment for Holostrophus

XML Treatment for Holostrophus bifasciatus

XML Treatment for Eustrophus

XML Treatment for Eustrophus tomentosus

XML Treatment for Eustrophopsis

XML Treatment for Eustrophopsis confinis

XML Treatment for Eustrophopsis bicolor

XML Treatment for Eustrophopsis brunneimarginatus

XML Treatment for Eustrophopsis indistinctus

XML Treatment for Eustrophopsis arizonensis

XML Treatment for Eustrophopsis ornatus

XML Treatment for Eustrophopsis crowdyi

XML Treatment for Synstrophus

XML Treatment for Synstrophus repandus

## Appendix 1

Non-type specimen locality data for species of North American Eustrophinae

### *Pseudoholostrophus (Pseudoholostrophus) impressicollis* (LeConte)

**CANADA**

**BRITISH COLUMBIA**

Gabriola, 2.vi.1994, BF & JL Carr, lot 7, (CARR, 1); Qualicum, 16.ix.1962, BF & JL Carr, lot 1, (CARR, 1); Vancouver Island, Kye Bay, nr. Comox, 5 m, 2.vii.1980, H. & A. Howden, (CMNC, 1); Queen Charlotte Islands, Laskeek Bay, Haswell Island, 2–22.vi.2000, 52°51'42"N, 131°41'06"W, Allombert, Sylvain, (RBCM, 1).

**UNITED STATES OF AMERICA**

**CALIFORNIA**

*Alpine Co*.: 38.4036°N, 119.8005°W, Stanislaus NF, Amot Ck., 21.viii.2005, M. Caterino, CA Beetle Project #CBP0034773, (SBMNH, 1).

*Humboldt Co*.: Arcata, 17.vii.1969, C. Slobodchikoff, (UCBC, 1); Laribee, 15.viii.1937, B.P. Bliven, (CAS, 4); same locality, 18.vii.1937, B.P. Bliven, (CAS, 1); same locality, 7.viii.1937, B.P. Bliven, (CAS, 1); Laribee Valley, 15.viii.1937, B.P. Bliven, (DAPC, 1).

*Siskiyou Co*.: 9 mi. N. Bartle, 12–15.vi.1974, D. Green, wh. fir log, (UCBC, 1).

*Trinity Co*.: Trinity Alps Resort, 20.viii.1973, under bark of pine, (DAPC, 1); same locality, 20.viii.1973, under bark of pine, (UCBC, 1).

**OREGON**

*Curry Co*.: Indian Creek, 25.vi.1978, J. Sehuh, L. Herman, fungus, (AMNH, 1).

*Josephine Co*.: Oregon Caves Nat. Mon, 3600ft, 4.vi.1972, R.L. Westcott, (ODAC, 1).

*Lane Co*.: Oakridge, 29.viii.1966, S. McCleve, in rotten log, (SMC, 1).

*Lincoln Co*.: 8.vi.1941, F.W. Nunenmacher, (FMNH, 1).

*Polk Co*.: 4 mi. W Falls City, 25.iii.1969, R.L. Westcott, (ODAC, 1).

### *Pseudoholostrophus (Holostrophinus) discolor* (Horn)

**CANADA**

**NEW BRUNSWICK**

Carleton Co., Wakefield, “Bell Forest Preserve”, 46.2200°N, 67.7231°W, 16.ix.2006, R.P. Webster, hardwood forest, on or near fleshy polypore fungi on beech log, (CNC, 1).

**QUEBEC**

[or Quebec City], 7.viii.1962, J.-C. Aubé, (DAPC, 1); same locality, 7.viii.1962, (FMNH, 2); Ste-Foy Quebec, Co., 31.vii.1962, J.-C. Aube, (AMNH, 2).

**UNITED STATES OF AMERICA**

**INDIANA**

*Parke Co*.: Turkey Run Sp, 1.vii.2001, L.E. Crofutt, (EIUC, 1).

**MARYLAND**

*Calvert* Co.: Battle Creek Cypress Swamp, 22.ix.1987, A.&B. Norden, D. Williams, (USNM, 1).

*Prince Georges Co*.: 39°3'N, 76°47'W, 28.vi-5.vii.1992, J. Pickering, (MTEC, 2).

**MASSACHUSETTS**

*Middlesex Co*.: Tyngsboro, 27.vi.1897, (MCZ, 1).

**NEW HAMPSHIRE**

*Carroll Co*.: Moultonboro, 30.vii.1975, Malaise trap, (LSU, 1).

*Grafton Co*.: Bedell Bridge S.P. @ Oliverian Brook, 20.viii-8.ix.1992, D.S. Chandler, FIT, (DENH, 1).

*Strafford Co*.: 4 mi W Durham, 23–26.viii.1982, R.M Reeves, Malaise trap, (DENH, 1); same locality, 30.vii-2.ix.1982, R.M Reeves, window trap, (DENH, 1); same locality, 26–28.vii.1982, R.M Reeves, Malaise trap, (DENH, 1).

**PENNSYLVANIA**

*Warren Co*.: 8.6 km S. Irvine, Hedgehog Run, 530 m., 41–45–43N, 79–15–42W, 26.vii-1.viii.1995, J.R. Deeds, intercept, (DAPC, 1).

**RHODE ISLAND**

*Kent Co*.: West Greenwich, Alton Jones, 7.vii-18.viii.1995, D.S. Sikes, FI trap, (DAPC, 2); same locality, 7.vii-18.viii.1995, D.S. Sikes, FI trap, (DSSC, 4).

**TENNESSEE**

*Sevier Co*.: Great Smoky Mtns. Star Park, ix.1953, in polypore fungus, D. Kistner, (FMNH, 1).

**VIRGINIA**

Ulke collection, (CMNH, 1).

### *Holostrophus bifasciatus* Say

**CANADA**

**NOVA SCOTIA**

Halifax Co., Pt. Pleasant Park, 15.vii.2001, C.G. Majka, mixed forest, deciduous vegetation, (CGMC, 1).

**ONTARIO**

Arnprior, 26.v.1935, W.J. Brown, (CNC, 12); Essex Co., Windsor Ojibway Prairie, 7.viii.2001, S. Paiero, Sweeps, (DEBU, 1); Jeannette Creek, 3.viii.1965, (FSCA, 1); Ottawa, (CNC, 6); same locality, (CUIC, 1); Peel Co., Port Credit, 10.iv.1966, I.M. Smith, (ROME, 1); Tillsonburg, 11.vii.1958, (CNC, 1); Toronto, 20.xi.1895, R.J. Crew, (CUIC, 1); no other data, (DEBU, 3); London, W. Saunders, (DEBU, 1); Ridgeway, (DEBU, 2).

**PRINCE EDWARD ISLAND**

Queens Co., St. Patricks, 25.vi.2003, C.G. Majka, coniferous forest, (CGMC, 2).

**QUEBEC**

Gatineau, Aylmer, 17.v.1992, Y. Bousquet, in flight, (SLC, 1).

**UNITED STATES OF AMERICA**

**ALABAMA**

*Dale Co*.: Enterprise, 8–12.vi.1992, R. Turnbow, blacklight trap, (RTC, 1).

*Lee Co*.: Auburn, 17.viii.1968, E.J. Kiteley, (CNC, 1); same locality, 7.iv.1972, E.J. Kiteley, (CNC, 1).

*Shelby Co*.: nr. Helena, 31.iii.1957, H.R. Steeves jr., in rotten logs, (FMNH, 1).

**ARKANSAS**

State record only, (INHS, 1).

*Drew Co*.: 5 mi. N. of Collins, 7 Devils Swamp, 16.iii.1987, R.A.B. Leschen, *Laetiporus sulphureus*, (RLC, 1).

*Logan Co*.: Cove Lake, 9 mi. SW of Paris on Hwy 309, 19.iii.1986, R.A.B. Leschen, ex. Polypore in pine logs, (RLC, 1); Mt. Magazine, 17 mi. S. of Paris on hwy 309, electronic site, 3.iv.1986, R.A.B. Leschen, *Trametes versicolor*, (RLC, 1).

*Newton Co*.: Ozark National Forest, Pedestal Rocks, 12 km E. Pelsor on Hwy 16, 16.v.1986, D.A. Pollock, (DAPC, 1).

*Polk Co*.: Mena, 18.vii.2007, B. Baldwin, U.V. light trap, (BBC, 1).

*Pulaski Co*.: Little Rock, 1.v.1997, B. Baldwin, flight intercept trap, (UGAC, 1); same locality, 9.vi.1997, B. Baldwin, flight intercept trap, (UGAC, 1); same locality and collector, 20.vi.1998, FIT, (BBC, 1); 4.v.1998, FIT, (BBC, 1); 6.v.1998, FIT, (BBC, 1); 2.viii.1998, FIT, (BBC, 1); 15.viii.2000, FIT, (BBC, 1); 11.vii.2000, FIT, (BBC, 1); 14.v.1998, FIT, (BBC, 1); 26.viii.2002, Malaise trap, (BBC, 1); 22.vii.1999, Malaise trap, (BBC, 1); 5.ix.1999, Malaise trap, (BBC, 1); 7.ix.2000, Malaise trap, (BBC, 1); Pinnacle Mtn. State Park, 1.v.1986, D.A. Pollock, under bark, (DAPC, 2).

*Washington Co*.: L. Wedington, 19.iv.1986, R.A.B. Leschen, *Schizopora paradoxa*, (RLC, 1); Lk. Wedington, 26.x.1986, R.A.B. Leschen, ex polypore on cherry tree, (RLC, 1).

*Counties unknown*: South West, Chas Palm, (AMNH, 3).

**CONNECTICUT**

*Fairfield Co*.: Stamford, (AMNH, 1).

*Hartford Co*.: Newington, 13.v.1961, R.P. Zigas, (UCMS, 1).

*Litchfield Co*.: Sharon, 20.ii.1925, A. Wilcox, (CUIC, 2).

*New Haven Co*.:S. Meriden, H.L. Johnson, (MCZ, 2).

**DISTRICT** **OF** **COLUMBIA**

(INHS, 1); Ulke coll., (CMNH, 4); 30.viii.1905, (USNM, 2); 8.ix.1904, (USNM, 2).

**DELAWARE**

*Sussex Co*.: Middlesex Beach, 1.viii.1984, F. Guarnieri, under loose pine bark, (CUIC, 3).

**FLORIDA**

*Hamilton Co*.: E.B. Chope, (FMNH, 1).

*Liberty Co*.: Torreya St. Park, 27.xi.1989, J.T. Doyen, (UCBC, 1).

*Monroe Co*.: Key West, (CUIC, 1).

**GEORGIA**.

*Bartow Co*.: Cartersville, 26.iii.1939, P. W. Fattig, (UGAC, 4). *Clarke Co*.: 1.1 mi SW Winterville, 20–23.iii.1994, C.L. Smith, ex: malaise trap, (UGAC, 2); Athens, 1–7.vi.1976, J.F. MacDonald & R.W. Matthews, Malaise trap, (UGAC, 1); U. Ga. Bot. Gar., 17.xi.1974, R. M. Duffield, (UGAC, 2).

*Clarke Co*.: county record only, 25.v.1976, R.T. Franklin, (DAPC, 1).

*Fulton Co*.: Atlanta, Wickham, (USNM, 1).

*Jackson Co*.: Hardeman For., 16–20.viii.1975, R. Turnbow, (FMNH, 1).

*Taliaferro Co*.: Crawfordville, 8.x.1997, D.A. Pollock, at night under bark, (DAPC, 1).

**ILLINOIS**

State records only: (INHS, 10); x, (INHS, 1); (MCZ, 2); (NDSU, 2); (UMRM, 1).

*Bond Co*.: LaGrange, 10.v.1915, (FMNH, 2); same locality, 10.v.1915, Selinger, (AMNH, 3).

*Carroll Co*.: Miss Pallisades St. Pk., 10.vii.1947, Sanderson et al., (INHS, 1).

*Champaign Co*.: Carle Woods, 5.vii.1941, (FMNH, 1); same locality, 5.vii.1941, (FMNH, 2); Urbana, 9.5.32, (FMNH, 1); Urbana, Brownfield Woods, 5.ix.1932, (FMNH, 1); county record only, 3.v.1960, J.M. Campbell, (AMNH, 1). *Clark Co*.: Rocky Branch, 30.viii-3.ix.1992, R.S. Hanley, flight trap, (DAPC, 1); same locality, 19–27.v.1991, M.A. Goodrich, Malaise trap, (DAPC, 1); same locality, 4–8.v.1998, M.A. Goodrich, Sugar Trap, (EIUC, 1); same locality, 22–29.v.1997, M.A. Goodrich, Pitfall Trap, (EIUC, 1).

*Clark Co*.: Rocky Branch, 30.viii-3.ix.1992, R.S. Hanley, flight trap, (DAPC, 1).

*Coles Co*.: Charleston, 11–12.vi.2001, M.A. Goodrich, uv light, (EIUC, 1); Fox Ridge Sp, 2.v.1998, M.A. Goodrich, under bark of rotten log, (EIUC, 1).

*Cook Co*.: E.B. Chope, (FMNH, 1); Carlé Woods, Des Plaines, 25.v.1956, R.C. Graves, in fungus, (MCZ, 1). Chicago, 1.viii.1907, (FMNH, 2); Chicago, 9.vii.1904, (FMNH, 1); same locality, 15.vi.1904, (FMNH, 1); same locality, v, Blackwelder, (WIRC, 1); same locality, 21.iv.1910, E. Liljeblad, (UMMZ, 1); same locality, 1.vii.1909, E. Liljeblad, (UMMZ, 1); Palos Park, 15.x.1925, oak log, (FMNH, 2); same locality, 19.vi.1910, W.J. Gerhard, (FMNH, 2); Willow Spring, 20.vi.1914, (UMMZ, 1); Edgebrook, 8.vii.1913, (UMMZ, 1); Riverside, 5.vii.1915, (UMMZ, 1).

*DuPage Co*.: Glen Ellyn, 19.vi.1904, W.J. Gerhard, (FMNH, 1).

*Edgar Co*.: 4 mi SSE Kansas, 19–26.iv.2000, M.A. Goodrich, Malaise trap, (EIUC, 1); same locality, 28.v-5.vi.1999, M.A. Goodrich, Pitfall Trap, (EIUC, 1); same locality, 2.x.1998, M.A. Goodrich, under bark of rotten log, (EIUC, 1).

*Kendall Co*.: Plano, H. Dybas, (FMNH, 1).

*Knox Co*.: Galesburg, (INHS, 1).

*LaSalle Co*.: 14.xii.1935, F.G. Werner, (MCZ, 1); Starved Rock St. Pk., 23.vi.1940, F.G. Werner, (MCZ, 1).

*Peoria Co*.: Peoria, 25.xii.1931, (FMNH, 5).

*Putnam Co*.: 24.viii.1933, M.O. Glenn, (INHS, 1); 2.ii.1933, M.O. Glenn, (INHS, 1).

*St. Clair Co*.: Kahokia, 4.v.1907, G.W. Bock, (UMRM, 1); same locality, 12.ix.1898, (UMRM, 2).

*Sangamon Co*.: Springfield, 25.iv.1926, (FMNH, 1).

*Union Co*.: Pine Hills Rec. Area nr. Wolf Lake, 3.v.1987, P. Skelley, under bark, (DAPC, 1).

*Wabash Co*.: Beall Woods N.P., 20.ix.1996, M.A. Goodrich, (EIUC, 1); same locality, 13–14.vi.1996, M.A. Goodrich, uv light trap, (EIUC, 1).

*Will Co*.: Joliet, 20.vi.1926, (FMNH, 1).

*Counties unknown*: Lowanda [?], 18.v.1883, (INHS, 1); Southern, [14.viii.1890], H. Soltau, (USNM, 1); New Lenox, 27.x.1934, F. Wiercinski, (FMNH, 1).

**INDIANA**

*Marion Co*.: 3.vii.1930, W.S. Blatchley, (CUIC, 2).

*Parke Co*.: 25.v.1963, N.M. Downie, (FMNH, 1).

*Porter Co*.: Indiana Dunes N.L., Beverly Shores, 14–28.vi.1995, R. Grundel, flight intercept trap, (DAPC, 3); same locality, 18.viii.1933, H. Dybas, (FMNH, 1).

*Posey Co*.: 8.v.1912, (FMNH, 1).

*Wayne Co*.: Richmond, 23.viii.1925, (FMNH, 1).

*Counties unknown*: W. Pullman, 27.x.1912, (FMNH, 1).

**IOWA**

State record only: (MCZ, 2).

*Johnson Co*.: Iowa City, 14.iv.1900, Wickham, (USNM, 2); same locality, x.1912, Wickham, (USNM, 1); same locality, x.1910, Wickham, (USNM, 5); same locality, 30.v, Wickham, (USNM, 1); same locality, 17.v, Wickham, (USNM, 1).

**KANSAS**

State record only: (INHS, 1).

*Shawnee Co*.: Auburn, 11.viii.1933, C. Seevers, (FMNH, 1); Topeka, Hubbard & Schwarz, (USNM, 1); same locality, C.V. Riley, (USNM, 1).

**KENTUCKY**

State records only: (USNM, 1), (AMNH, 1); C.V. Riley, (USNM, 1).

*Butler Co*.: 11.v.1970, J.M. Campbell, (CNC, 1).

*Jefferson Co*.: Lou’vlle, [1.9], H. Soltau, (USNM, 1).

**LOUISIANA**.

*Natchitoches Par*.: Kisatchie Natl. For., red Bluff Camp., 1.iv.1989, E. Riley & L. Prochaska, berlese rotten log with fungus, (TAMU, 1).

**MAINE**

*Kennebec Co*.: Augusta, 9.ix.1944, A.E. Brower, in nest *Camponotus pennsylvanica* 25 ft up in oak, (DENH, 1); Monmouth, 20.vi.1907, C.A. Frost, (MCZ, 2).

**MARYLAND**

State records only: (USNM, 1); Ulke coll., (CMNH, 2).

*Baltimore Co*.: Chase, 22.iii.1953, G.H. Nelson, (AMNH, 1).

*Calvert Co*.: Battle Creek cypress swamp, 6.vii.1987, A. & B. Norden, D. Williams, (USNM, 1); Flag Ponds Nature Park, 12.v.1987, A. &B. Norden & D. Williams, (USNM, 1).

*Cecil Co*.: Port Deposit , 19.iv.1977, D. Jump, (USNM, 1).

*Dorchester Co*.: nr Lloyds, 10.vii.1907, H.S. Barber, (USNM, 1).

*Harford Co*.: Susquehana St. Pk., 24.iv.1988, CL & SL Staines, Taken under pine bark, (USNM, 1).

*Montgomery Co*.: 4 mi SW of Ashton 390630N 770130W, 15.iv.2002, G.F. Hevel, Malaise trap, (USNM, 1); same locality, 28.v.2002, G.F. Hevel, (USNM, 1); Fairland, 27.iii.1981, W.E. Steiner, (USNM, 1); Gaithersburg, 25.v.1972, W.E. Steiner, (USNM, 1); Potomac, 21.iv.1972, W.E. Steiner, (USNM, 1); Colesville, 16.xi.1951, D. Kissinger, (CNC, 1); near Plummers Id, 16.ix.1918, J.L. Wrenn, (USNM, 1); 2 mi. E. Silver Sprgs., on trunk beech, 25.iv.1951, C.H. Nelson, (AMNH, 1).

*Prince George’s Co*.: College Park, 22.iv.1948, (CMNC, 1); same locality, 26.iv.1948, B.K. Dozier, coll. on stump under tar paper, (FSCA, 3); same locality, 14.xii.1948, B.K. Dozier, under log in oakwoods, (FSCA, 1); same locality, 22.iv.1948, B.K. Dozier, in old log, (FSCA, 1); same locality, 5.iv.1951, G.H. Nelson, rotten pine log, (UCRC, 3); same locality, 5.iv.1951, G.H. Nelson, on rotten pine log, (WFBM, 1); Camp Springs, 6.viii.1980, Gary F. Hevel, Malaise trap, (USNM, 1); Cheverly 38°56’N, 76°55’W, 10.vii.1993, W.E. Steiner, (USNM, 1); same locality, 10.viii.1997, W.E. Steiner 7 J.M. Swearingen, (USNM, 1).

*Talbot Co*.: Easton (Seth St. For.), 8.iv.1981, W.E. Steiner, (USNM, 1); Wittman, 12.x.1975, W.E. Steiner, (USNM, 1).

*Wicomico Co*.: Allen, 27.v.1976, D. Jump, (USNM, 1).

*Worcester Co*.: 5 mi S Pocomoke, 25.iv.1970, M. Drunkenbrod, u. Bark, (USNM, 1); Snow Hill, 13.ix.1973, W.E. Steiner, (USNM, 1).

*Countries unknown*: Edgewood Arsenal, 1.vi.1969, A & A. Gillogly, (MCZ, 1). C Baltimore Homewood, 8.iv.1941, Dieke, (USNM, 1).

**MASSACHUSETTS**

State record only: (MCZ, 1).

*Hampden Co*.: Springfield, G. Dimmock, (MCZ, 4).

*Hampshire Co*.: Amherst, 14.iv.1942, Moulton, (UMMZ, 1).

*Middlesex Co*.: Natick, 26.v.1947, C.A. Frost, u. bark white pine stump, (MCZ, 1); Lexington, 14.v.1949, Chamberlain, (MCZ, 1); Lincoln, 2.v.1937, C.H. Blake, (MCZ, 1); Medford, (MCZ, 1); Sherborn, 3.v.1924, C.A. Frost, sifting rotten log, (MCZ, 1); Tyngsboro, (MCZ, 7); same locality, 20.v.1888, (MCZ, 2).

*Norfolk Co*.: Brookline, 27.v.1899, (MCZ, 1).

*Suffolk Co*.: Dorchester, 20.v, (MCZ, 1).

**MICHIGAN**

*State record only*: (CUIC, 3).

*Livingston Co*.: E.S. George Reserve, 7.viii.1950, K. Bohnsack, (UMMZ, 2); same locality, 16.vii.1933, A.W. Andrews, fungus, (UMMZ, 1).

*Oakland Co*.: county record only, 20.viii.1923, A.W. Andrews, (UMMZ, 1).

*Wayne Co*.: Detroit, G. Dimmock, (MCZ, 1); same locality, (USNM, 3); same locality, 27.v.1906, (UMMZ, 1); county record, 5.v.1930, (UMMZ, 1).

**MINNESOTA**

*Cook Co*.: Tofte, 11.vii.1957, E.J. Kiteley, (CNC, 1).

**MISSISSIPPI**

*George Co*.: Lucedale, 29.iii.1932, H. Dietrich, (CUIC, 4).

**MISSOURI**

State record only: (INHS, 1).

*Hickory Co*.: W. Quincy, 18.viii.1912, (FMNH, 2; UMMZ, 4); same locality, 16.vii.1912, (UMMZ, 1).

*Randolph Co*.: 1 mi. E. Moberly, 7.vi.1973, E.G. Riley, (EGRC, 1); same locality, 31.vii.1977, E.G. Riley, (EGRC, 1).

*St. Louis Co*.: St. Louis, (UMRM, 4).

**NORTH CAROLINA**

State records only: (CMNH, 9); (CUIC, 4); (INHS, 1); (MCZ, 5); H.W. Wenzel collection, (OSUC, 4); (USNM, 1); (USNM, 1); (USNM, 2).

*Buncombe Co*.: Asheville, 3.iv.1974, L.L. Lampert, (FSCA, 1).

*Gaston Co*.: Tryon, W.F. Fiske, (USNM, 1); same locality, W.F. Fiske, Castanea dentata, (USNM, 3); Pinnacle Mt., 20.v.1972, R. Ables, ex fungi on log, (TAMU, 1).

*Moore Co*.: Southern Pines, 1–9.1907, A.N. Mance [?], (UCRC, 1).

*Wake Co*.: Raleigh, 4.x.1949, H. & A. Howden, fungus, (CMNC, 1); same locality, 3.iv.1958, D.H. Habeck, (FSCA, 1); same locality, 23–25.vi.1982, J.B. Whitfield, (UCBC, 1).

**NEBRASKA**

*Sarpy Co*.: Fontenelle For., 2–9.v.1998, M.A. Goodrich & C.A. Springer, Malaise trap, (EIUC, 2).

**NEW** **HAMPSHIRE**

*Hillsborough Co*.: Wilton, 2.ix.1901, (MCZ, 1).

*Rockingham Co*.: Hampton, 3.vii.1932, S. Albert Shaw, (DENH, 1); same locality, 23.v.1932, S. Albert Shaw, (DENH, 1); same locality, 24.v.1931, S. Albert Shaw, (DENH, 1).

*Strafford Co*.: Durham, 28.v.1936, Basil G. Markos, (DENH, 1); same locality, W & F, hibernating, (DENH, 1); same locality, W&F, hibernating, (USNM, 1); same locality, W&F, (USNM, 1).

**NEW** **JERSEY**

State records only: (CUIC, 1); (MCZ, 3); (AMNH, 2); (USNM, 5); ML Linell, (USNM, 3).

*Bergen Co*.: Fort Lee, 15.iv.1911, A. Nicolay, (AMNH, 1); same locality, (AMNH, 2); Palisades, vi.12, (CUIC, 1); same locality, 15.iv.1911, A. Nicolay, (USNM, 1).

*Burlingron Co*.: Bridgeboro, 28.xi.1924, R.J. & M.B. Sim, (USNM, 1); N. Lisbon, 24.iii.1929, EJF Marx, (AMNH, 1); Medford Twp. Lake Pine, 25.vii.1975, D.C. Rentz, Malaise trap, (USNM, 1).

*Cumberland Co*. [Bridgeton] Bridgalon, 28.iii.1934, F.M. Schott, (AMNH, 1).

*Essex Co*.: Newark, 4.vii, GeoM Greene, (USNM, 2); Orange Mt., H.W. Wenzel collection, (OSUC, 3).

*Middlesex Co*.: Avenel, 7.ix.1916, (UAIC, 1); Morgan, iv, (CUIC, 1).

*Ocean Co*.: Manchester, 15.iv, (CUIC, 2); same locality, 15.iv, L.B. Woodruff, (AMNH, 1).

*Counties unknown*: Catskil Mts., L.B. Woodruff, (AMNH, 1); Forest Park, 28.iii.1903, (CUIC, 1); Harrison Park, 10.iv.1916, E.R. Kalmbach, (USNM, 1); Woodside, iii, E.L. Dickerson, (AMNH, 2).

**NEW YORK**

State records only: (CUIC, 2); (MCZ, 2).

*Bronx Co*.: Van C Pk, (AMNH, 3).

*Erie Co*.: Buffalo, (CUIC, 2).

*Monroe Co*.: Rochester, 3.viii.1944, F.C. Fletcher, (LACM, 1).

*Onondaga Co*.: 15.v.1946, N.M. Downie, (FMNH, 1); 11.vi.1946, N.M. Downie, (FMNH, 1).

*Orange Co*.: West Point, 14.v.1910, W. Robinson, (USNM, 1).

*Richmond Co*.: Staten Isld., 8.v.1921, (USNM, 1).

*Suffolk Co*.: Bellport L.I., 17.vi.1914, A. Nicolay, (USNM, 3); Yaphank, L.I., v-17, (CUIC, 1).

*Queens Co*.: Flushing, 5.v.1926, E.L. Bell, (AMNH, 1); Jamaica, L.I., (CUIC, 1). Jamaica L.I.N.Y., L.B. Woodruff, (AMNH, 2); L.I., Little Neck, 12.xii.1915, F.M.Schott, (AMNH, 1); Long Island, 22.xii.1912, F.M. Schott, (UAIC, 1).

*Tompkins Co*.: Town of Ulysses, N. of Jacksonville, 21.iv.1985, E.R. Hoebeke, (CUIC, 1); Trumansburg, H.A. Smith Woods, 26.viii.1987, E.R. Hoebeke, (CUIC, 1); Ithaca, V.M. Kirk, under willow bark, (NDSU, 1); same locality, (USNM, 2).

*Westchester Co*.: Peekskill, 3.12 1890, (CUIC, 2); Pelham, 12.iv.1931, L. Lacey, (AMNH, 1).

*Counties unknown*: Northwest, L.I., 1.vi.1942, (CUIC, 1).

**OHIO**

State records only: Ulke coll., (CMNH, 1); (CUIC, 6); (NDSU, 2); (USNM, 2); Hubbard & Schwarz, (USNM, 3); C.V. Riley, (USNM, 1).

*Clark Co*.: Clifton, 21.v, D.J. & J.N. Knull, (OSUC, 1).

*Franklin Co*.: Darbydale, 7.iv.1974, K. Stephan, (FSCA, 1).

*Hamilton Co*.: Cincinnati, (CUIC, 1); Cincinnati, 14.vi, H.S. Itau, (USNM, 1); same locality, H. Soltau, (USNM, 2).

*Ross Co*.: Scioto Trail S.P., 17.iv.1989, (TAMU, 2); Tar Hollow St. For., 24.v.1985, R.S. Miller, (MTEC, 1).

*Wyandot Co*.: Sycamore, 24.v.1919, P.S. Bartholomew, (CAS, 1).

**PENNSYLVANIA**

State records only: Ulke coll., (CMNH, 2); (MCZ, 5).

*Adams Co*.: Chestnut Hill, 3.vii, (MCZ, 1).

*Allegheny Co*.: county records only: (MCZ, 3); (CMNH, 9); 6.ix, (CMNH, 1); 1.xi.1904, E.A. Klages, (CUIC, 2); 29.vii.1888, E.A. Klages, (CUIC, 1); 1.v.1906, E.A. Klages, (CUIC, 1); 17.iii.1889, E.A. Klages, (CUIC, 1); 23.vii.1896, E.A. Klages, (CUIC, 1); Pittsburgh, (CMNH, 6); same locality, 21.vi, (CMNH, 2); same locality, vii, (CMNH, 1); same locality, 10.vi, (CMNH, 1); same locality, 4.vii, (CMNH, 1); same locality, vii, (CMNH, 1); same locality, 10.vii, (CMNH, 2).

*Armstrong Co*.: Crooked Creek, Wickham Collection, (USNM, 1).

*Berks Co*.: Neversink, 29.viii.1951, (FMNH, 1); 2.3 km W. Eckville, 270 m., 14.vii.1997, R.A. Androw, (DAPC, 2).

*Dauphin Co*.: Hummelstown, 1.v, J.N. Knull, (OSUC, 1).

*Philadelphia Co*.: Frankford, 23.x, GeoM Greene, (USNM, 1); Lawndale, 27.iv.1902, GeoM Greene, (USNM, 1)

*Pike Co*.: Milford, 30.v-1.vi.1941, B. Malkin, (FMNH, 1).

*Westmoreland Co*. Jeannette, 20.vi, H.G. Klages, (CMNH, 1); same locality, vi, (CMNH, 3); same locality, vii, (CMNH, 2); same locality, 4.vi, (CMNH, 1); same locality, 4.vii, H.G. Klages, (CMNH, 1); same locality, 5.vii, H.G. Klages, (CMNH, 1); same locality, 11.vi, (CMNH, 1).

*Counties unknown*: Timicum Is. , 20.x.1901, GeoM Greene, (USNM, 1); Mt. Lebanon, ix.1930, R.J. Simon, (CMNH, 1).

**SOUTH** **CAROLINA**

State record only: (MCZ, 1).

*Florence Co*.: Florence, 13.xi.1952, V.M. Kirk, under dead bark, (NDSU, 2).

*Oconee Co*.: Clemson College, 19.xi.1933, O.L. Cartwright, (USNM, 1).

*Pickens Co*.: Clemson, 30.xi.1951, G.M. Chamberlain, under bark, (TAMU, 1); same locality, 30.iv.1975, J.R. Ables, (TAMU, 1).

*Counties unknown*: Union, 5.iii.1961, (USNM, 2); same locality, 4.iii.1962, (USNM, 1).

**TENNESSEE**

State records only: Ulke coll., (CMNH, 2); (CUIC, 2).

*Chester Co*.: Chickasas St. Park, 27.xii.1977, R.D. Ward, (CMNH, 1).

*Cumberland Co*.: Black Mt., v-viii.1917, A.C. Gill, (CUIC, 1).

*Morgan Co*.: Burrville, 17.vii.1954, B. Benesh, (FMNH, 1).

**TEXAS**

State record only: (MCZ, 1).

*Sabine Co*.: 9 mi. E. Hemphill, “beech bottom”, 10–16.iv.1989, Anderson & Morris, flight intercept trap, (DAPC, 1).

**VIRGINIA**

*Bath Co*.: Warm Sprgs., x.1, (CUIC, 1).

*Clarke Co*.: U. Va. Blandy Exp. Farm, 2 mi S boyce 3905N 7810W, 11–25.v.1993, D.R. Smith, Malaise trap, (USNM, 1).

*Fairfax Co*.: Alexandria, Huntley Meadows Pk., 12–19.vi.1991, B.V. Brown, Malaise trap in oak/maple forest, (DAPC, 1); Springfield, 11.iv.1992, E.R. Fuller & B.V. Brown, under bark of dead trees, (DAPC, 1); Mt. Vernon, 23.vi.1916, (USNM, 1).

*Lee Co*.: Jone’s Cr, (USNM, 1).

*Counties unknown*: St. Falls, 17.ix.1913, (USNM, 1).

**WEST VIRGINIA**

*Berkeley Co*.: Martinsburg, 15–22.vi.1994, W.L. Drosselmeyer, Lindgren funnel trap, (PPCD, 1).

*Kanawha Co*.: 1 mi E Rocky Fork, 31.viii-15.ix.1994, S.M. Clark, Lindgren funnel trap, (PPCD, 1).

*Lewis Co*.: Berlin, 26.v-1.vi.1995, M.D. Bombardière, Lindgren funnel trap, (PPCD, 1).

*Morgan Co*.: 3 mi NNE Largent, 19–31.iii.1998, W.L. Drosselmeyer, Lindgren funnel trap, (PPCD, 1).

*Roane Co*.: 1 km E. Kettle, Hungry Beech Preserve, 38–34N, 81–28W, 270 m+A62, 5–8.vi.1995, A. Harrity, R. Davidson, G. Onore, light trap in decid. forest, (CMNH, 1).

**WISCONSIN**

State records only: (CAS, 1); (FMNH, 2); (FMNH, 2); (INHS, 3); (WIRC, 1).

*Dane Co*.: 13.v.1965, R. Baier, (WIRC, 1); 6.10.1957, Nicholls, (WIRC, 1); Madison, 30.vii.1931, (FMNH, 1); same locality, 12.x.1940, H.R. Dodge, from fungus, *Hypoloma perplexa*, (FSCA, 1); Madison, Picnic Point, 6.vi.1995, R. Otto, coll. on oak log, (DAPC, 1); Madison, U.W. campus, 12.vi.1995, R. Otto, coll. on oak log, (DAPC, 1).

*Grant Co*.: Blue River, 28.vii.1916, D.M. DeLong, (OSUC, 1).

*Oconto Co*.: Mountain, 20.vi.1935, H. Dybas, (FMNH, 2).

*Waukesha Co*.: 4.ix.1970, B. Rogan, (WIRC, 3).

### *Eustrophus tomentosus* Say

**CANADA**

**BRITISH COLUMBIA**

Canal Flats, 21.viii.1977, Carrs, lot 1, (CARR, 4); same locality, 21.viii.1977, B.F. & J.L. Carr, lot 1, (DAPC, 1); Creston, 17.v.1931, G. Stace Smith, (CNC, 5); same locality, 19.v.1956, G. Stace Smith, (CNC, 1); same locality, 17.v.1931, G. Stace Smith, (UBC, 11); same locality, 22.vii.1948, G. Stace Smith, from fungus, (UBC, 1); same locality, 12.vii.1946, G. Stace Smith, host: *Pseudotsuga taxifolia*, (UBC, 1); same locality, 3.viii.1945, G. Stace Smith, in fungi, (UBC, 1); same locality, 5.viii.1950, G. Stace Smith, from fungus, (UBC, 1); same locality, 17.v.1957, G. Stace Smith, evening flight, (UBC, 1); same locality, 4.ix.1951, G. Stace Smith, evening flight, (UBC, 1); same locality, 17.vi.1951, G. Stace Smith, ex fungus, (UBC, 1); same locality, 11.ix.1951, G. Stace Smith, ex fungus, (UBC, 1); same locality, 1.vi.1952, G. Stace Smith, ex fungus, (UBC, 1); same locality, 25.vi.1958, G. Stace Smith, ex fungus, (UBC, 1); same locality, 6.v.1953, G. Stace Smith, evening flight, (UBC, 1); same locality, 18.v.1951, G. Stace Smith, ex *Populus trichocarpa*, (UBC, 1); same locality, 15.vi.1948, G. Stace Smith, from fungus, (UBC, 1); same locality, 22.vii.1948, G. Stace Smith, from fungus, (UBC, 1); Creston, 1900 ft., 25.v.1933, G. Stace Smith, surface of pond, (UBC, 1); Creston, 2000 ft., 20.ii.1932, G. Stace Smith, in bark *Larix occidentalis*, (UBC, 1). Gabriola, 29.iv.1993, Carrs, lot 1, (CARR, 5).

**MANITOBA**

Winnipeg, Univ. of Manitoba campus, forest along Red River, 5–8.vii.1985, D.A. Pollock, suction trap, (DAPC, 1).

**NOVA SCOTIA**

Guysborough Co., Borneo, 28.vi.1995, C. Corkum, (NSMC, 1); Halifax Co., Big St. Margarets Bay, 29.vii-13.viii.1997, D.J. Bishop, in old red spruce habitat, (NSMC, 1); Queens Co., Tobeatic Lake, 3.vi.2003, P. Dollin, red spruce habitat (80–120 yrs.), (NSMC, 2).

**ONTARIO**

no other data, (MCZ, 1). Arnprior, 26.v.1935, W.J. Brown, (CNC, 1); same locality, 9.vi.1935, W.J. Brown, (CNC, 1); Bell’s Corners, 17.x.1962, D.D. Munroe, (CNC, 3); same locality, 10.vii.1963, D.D. Munroe, (CNC, 1); Chaffeys Locks Biol. Stn., 6.vi.1986, A. Davies, berlese fleshy fungi on tree, (CNC, 1); Constance Lake, 10.vi.1989, G. Hilchie, ex mushroom, (CARR, 1); DeCew Falls, 6.v.1942, S.D. Hicks, (CNC, 1); Hamilton, 15.vi.1982, M. Sanborne, (CNC, 1); same locality, 28.vi-14.vii.1982, M. Sanborne, (CNC, 2); same locality, 30.vi-8.vii.1981, M. Sanborne, (CNC, 1); same locality, 23.vii.1980, M. Sanborne, Malaise trap, (CNC, 1); Lambton Co., Pinery Prov. Park, 15–20.vi.1994, J. Skevington, Malaise Cedar trail oak savanna meadow, (DEBU, 2); same locality, 22.vi.1995, G. Vogg, Visitor Centre, (DEBU, 1); Lanark, 17.ix.1977, S.A. Marshall, (JBWM, 3); Leamington, 18.vi.1940, W.J. Brown, (CNC, 1); Merivale, 4.vi.1935, W.J. Brown, (CNC, 1); Ottawa, 14.viii.1912, Beaulieu, (CNC, 2); same locality, (CNC, 4); same locality, 17.v, under bk. of pine, (CNC, 3); same locality, 14.viii.1912, Beaulieu, (MCZ, 2); Prince Edward Co., 15.vi.1947, Brimley, (CARR, 3); same locality, 23.iv.1922, Brimley, (CNC, 1); same locality, 21.vii.1918, Brimley, (CNC, 1); same locality, 20.vii.1938, Brimley, (CNC, 1); same locality, 13.vi.1940, Brimley, (CNC, 1); same locality, 16.ix.1923, Brimley, (CNC, 1); same locality, 21.vii.1918, Brimley, (CNC, 1); same locality, Brimley, (UAIC, 3); same locality, Brimley, (UAIC, 3). Rondeau Pk., 2–6.vi.1985, L. LeSage & A. Smetana, flight intercept in white pine stand, (CNC, 3); Rondeau Pk., 2–6.vi.1985, L. LeSage & A. Smetana, int. trap in maple-beech forest, (CNC, 1); same locality, 14.vi-2.vii.1985, L. LeSage & D.M. Wood, (CNC, 1); Rondeau Pr. Pk., Black Oak Trail, 5.vi.1985, A. Davies & J.M. Campbell, under wet moldy bk. on dead tree, (CNC, 1); Toronto, R.J. Crew, (CUIC, 2); same locality, 2.i.1895, R.J. Crew, (CUIC, 4); same locality, iii, Crew, (WIRC, 1); Trenton, 25.v.1899, Evans, (CNC, 1); no other data, (DEBU, 2); Ariss, 18.vii.1976, S.A. Marshall, (DEBU, 1); Gananoque, 22.vi.1963, C.J. Edwards, (DEBU, 1); Grimsby, 14.vi.1977, W.A. Attwater, (DEBU, 2); Guelph, 17.vii.1976, S.A. Marshall, (DEBU, 5); same locality, 27.iii.1977, Kevin Barber, (DEBU, 10); Lanark, 2.viii.1976, M.J. Sharkey, (DEBU, 1); London, W. Saunders, (DEBU, 3); same locality, 28.vi.1976, W.A. Attwater, (DEBU, 1); Oakville, 21.vii.1977, W.A. Attwater, (DEBU, 1); Port Credit, 16.v.1993, C. Krupke, rotting wood, (DEBU, 1); Pr. Edw. Co., Brimley, 2.vi.1940, (AMNH, 1); Primrose, 27.vii.1977, W.A. Attwater, (DEBU, 1); Ridgeway, (DEBU, 1); Whitby, 21.iv.1974, G.J. Umphrey, (DEBU, 1).

**QUEBEC**

Aylmer, 12.v.1932, W.J. Brown, (CNC, 1); Gatineau Pk., 24.v.1970, J.M. Campbell, (CNC, 1); Montreal, 4.iv.1969, E.J. Kiteley, elm, (CNC, 1); same locality, 27.viii.1973, E.J. Kiteley, uv light trap, (CNC, 1); same locality, 21.vii.1974, E.J. Kiteley, uv light trap, (CNC, 1); same locality, 25.vi.1980, E.J. Kiteley, cut surface stump, (CNC, 1); same locality, 26.viii.1973, E.J. Kiteley, uv light trap, (CNC, 1); Old Chelsea, NCC woodpile, 27.v-5.vi.1987, L. Masner, pans, (CNC, 1); Riviera Touraine, 12.iv.1974, R. Sexton, *Ulmus americana*, (CNC, 15); same locality, 14.iv.1974, R. Sexton, *Ulmus americana*, (CNC, 19); Terrebonne, Saint-Hippolyte, 27.v.1978, S. Laplante, uv light, (SLC, 1). LOCALITY UNKNOWN. no other data, (CUIC, 1); Ulke collection, (CMNH, 1); no other data, (USNM, 1).

**UNITED STATES OF AMERICA**.

**ALABAMA**

*Lee Co*.: Auburn, 3.iv.1969, E.J. Kiteley, under loose bark of pine, (CNC, 1); same locality, 26.iv.1966, E.J. Kiteley, under bark, (CNC, 1); same locality, 26.iii.1972, E.J. Kiteley, under bark of dead pine, (CNC, 1).

*Madison Co*.: Monte Sano St. Pk., 3–4.ix.1983, N.M. Downie, (FMNH, 1).

*Marion Co*.: 1.8 mi. SW. Hackleburg, Clifty Creek, 6.vi.1983, S.C. Harris, (PKLC, 1).

*Mobile Co*.: Mobile, 22.vi.1919, G.W. Bock, (UMRM, 1); same locality, 25.x, H. Soltau, (USNM, 1).

**ARIZONA**

no other data, (INHS, 1).

*Coconino Co*.: Tusayan District, Kaibab Nat. Forest, 22.viii.1976, Carrs, lot 5, (CARR, 1); 1.5 mi. S. Hwy 40 & 3.8 mi. W. Flagstaff (off FSR 151), 35.1795, -111.7553, 17–19.vii.2007, D. & G. Pollock, coll. at night on ground or on dead pine logs, 7350 ft. elev., (DAPC, 4).

*Pima Co*.: Santa Catalina Mts. , 15.vi.1940, O. Bryant, lot 23, (CAS, 1); Tucson, 5.viii.1935, O. Bryant, lot 20, (CAS, 1); same locality, Wickham, (USNM, 1).

**ARKANSAS**

no other data, (CUIC, 3); no other data, (INHS, 1).

*Crawford Co*.: Ozark Nat’l Forest, 1.5 mi. S. of Natural Dam off Hwy 59, 31.viii.1986, R. Leschen, *Spongipellis unicolor*, (RLC, 1).

*Faulkner Co*.: Bell Slough Wildlife Management Area, Mayflower nr. Lake Conway Spillway, 24.viii.1986, R. Leschen, *Climacodes septentrionale*, (RLC, 2).

*Fulton Co*.: mammoth Spring, 28.v.1983, J.R. Heitzman, decid. forest & lowland flora, (FSCA, 2).

*Logan Co*.: Mt. Magazine, Brown spgns., 12.v.1986, R. Leschen, *Trametes versicolor*, (RLC, 1).

*Polk Co*.: Mena, 19.ix.2007, U.V. light traps, B. Baldwin, (BBC, 2).

*Pulaski Co*.: Little Rock, 30.iii.1997, Brian Baldwin, flight intercept trap, (UGAC, 1); same locality, 7.ix.1998, B. Baldwin, Malaise trap, (BBC, 1); same locality, 10.viii.1999, B. Baldwin, black light trap, (BBC, 1); same locality, 7.ix.2000, B. Baldwin, malaise trap, (BBC, 1); same locality, 22.vi.1997, B. Baldwin, flight intercept trap, (BBC, 1); same locality, 21.vi.1996, B. Baldwin, hanging sugar traps, (BBC, 1); same locality, 3.ix.1998, B. Baldwin, flight intercept trap, (BBC, 2); same locality, 29.v.2003, B. Baldwin, blacklight trap, (BBC, 1); same locality, 6.vii.1998, B. Baldwin, flight intercept trap, (BBC, 1); same locality, 17.viii.2000, B. Baldwin, black light trap, (BBC, 1); same locality, 8.v.1999, B. Baldwin, hanging sugar traps, (BBC, 1); same locality, 24.iv.2002, B. Baldwin, flight intercept, (BBC, 2); same locality, 3.ix.1998, B. Baldwin, flight intercept trap, (BBC, 2). *Counties unknown*: South West, Chas Palm, (AMNH, 2).

**CALIFORNIA**

no other data, (FMNH, 2).

*Butte Co*.: 5 mi. N. Forest Ranch, 5/9.1971, under pine bark, (UCRC, 3).

*Calaveras Co*.: 4.8 km S. West Point, 26.vii.1980, S.C. Williams, (CAS, 1).

*El Dorado Co*.: 1 mi. E. Pacific House, 9.v.1992, W.D. Shepard, carrion traps, (CAS, 1); Blodgett Forest, 22.xi.1986, K. Hobson, (UCBC, 1); same locality, 17–21.iv.1989, K. Hobson, (UCBC, 1); Blodgett FRS, 2.vi.1986, Hobson / Atkinson, Lindgren trap turpentine bait, logged area, (UCBC, 1); Somerset, 19.vi.1969, R. Wharton, (TAMU, 1); El Dorado Natl. For.; nr. Jct. of Hwy 50 & Icehouse Rd., 38°46’N, 120°26’W; el. ~3200’, 2.iv.2005, A.R. Cline, ex. berleseate, (DAPC, 2).

*Placer Co*.: Gold Run, 20.iii.1964, W.F. Barr & O.O. Fillmore, (WFBM, 2).

*Yuba Co*.: Challenge, 6.iv.1981, R.S. Miller, (MTEC, 1). Sierra Foothill Field Sta., 6 mi. N. Smartville, 1500–1900’, 4.v.1980, J.A. Powell, on *Pinus ponderosa*, (UCBC, 1).

**COLORADO**

Wickham, (USNM, 1).

**CONNECTICUT**

*Litchfield Co*.: Cornwall, 13.viii.1920, Chamberlain, (CUIC, 2).

**DELAWARE**

*Sussex Co*.: Ellendale Forest, 1.ii.1970, M. Drunkenbrod, under bark, (USNM, 1).

**DISTRICT OF COLUMBIA**

Ulke collection, (CMNH, 2); Washington, 22.ii.1886, (CUIC, 1).

**FLORIDA**

*Dixie Co*.: Suwanee R. St. Pk., Suwanee, 15.vi.1981, S. Peck, UV light, (CNC, 1).

*Monroe Co*.: Key West, (CUIC, 1). *Dixie Co*.: US 27 W. of Shamrock, 7.v.1981, J.K. Liebherr, ex Polyporus fungus, (UCBC, 1).

**GEORGIA**

*Bartow Co*.: Cartersville, 26.iii.1939, P. W. Fattig, (UGAC, 1).

*Clarke Co*.: 1.1 mi. SW Winterville, 18–22.iv.1994, C.L. Smith, ex: malaise trap, (UGAC, 2); 3 mi. N. Athens, 14.iv.1974, R. Turnbow, b.l. trap, (FSCA, 1); same locality, 2.iv.1974, R. Turnbow, b.l. trap, (FSCA, 1); 5 mi. W. Athens, 7.viii.1977, C. L. Smith, under oak bark, (UGAC, 3); Athens, 12.iii.1938, P. W. Fattig, (UGAC, 1); same locality, 8–14.v.1976, J. F. MacDonald & R. W. Matthews, malaise trap, (UGAC, 1); same locality, 12.iii.1938, P. W. Fattig, (UGAC, 1); Athens, 12.i.1964, L.J. Bayer, (WIRC, 4); same locality, 12.3.1922, P.W. Fattig, (MCZ, 1).

*Echols Co*.: Mayday, at Alapaha River, 25.ii.1991, W. Steiner & J. Hill, (USNM, 2). *Lamar Co*.: Barnsville, 1.v.1923, T.L. Bissell, tanglefoot Trap Posts, (USNM, 1).

**IOWA**

*Johnson Co*.: Iowa City, 11.x.1914, J.J. Stoner, (USNM, 2); same locality, 16.v.1896, Wickham, (USNM, 3).

*Keokuk Co*.: Atwood, B. Shivek, (USNM, 1).

*Story Co*.: Ames, 3.v.1941, L. Potter, (NDSU, 1); same locality, 22.v.1932, Louise Haas, (DENH, 1); same locality, 2.v.1932, Floyd Andre, (USNM, 2); same locality, 22.iv.1955, A.H. Barnum, (BYUC, 2); same locality, 1.vii.1989, M.E. Rice, UV light in oak-maple forest, (WFBM, 1). *Counties unknown*: Burl, (MCZ, 1).

**IDAHO**

*Boundary Co*.: N. of Bonners Ferry, 20.vi.1948, L.R. Gillogly, fungus on dead pine, (CAS, 1).

*Clearwater Co*.: 5 mi NW Pierce, 2.viii.1972, (USNM, 1).

*Latah Co*.: Moscow, 2560 ft., 5.vii.1930, P. Rice, (WFBM, 1). same locality, 12.vii.1930, P. Rice, (WFBM, 1).

**ILLINOIS**

*Bond Co*.: Lagrange, 25.v.1920, (UMMZ, 1).

*Champaign Co*.: Urbana, 20.iv.1907, (INHS, 3); no other data, 11.iv.1926, K.F. Auden, (INHS, 1); no other data, 20.iii.1960, J.M. Campbell, under bark, (CNC, 1).

*Clark Co*.: Rocky Branch, 30.vii-8.viii.1991, M.A. Goodrich, Malaise trap, (DAPC, 1); same locality, 1–7.ix.1988, M.A. Goodrich, (DAPC, 1).

*Coles Co*.: Charleston, 11–12.vi.2001, M.A. Goodrich, uv light, (EIUC, 1); same locality, 1–2.vii.2002, M.A. Goodrich, uv light, (EIUC, 1); same locality, 1.xi.1999, D. Scheiman & N. Dust, (EIUC, 1); vcty Charleston , 16.v.2001, N.L. Owens & M.A. Goodrich, (EIUC, 1).

*Cook Co*.: Edgebrook, 9.iv.1905, (FMNH, 1); Chicago, 16.v.1920, C. Selinger, (UMMZ, 6); Riverside, 13.v.1916, (UMMZ, 1).

*LaSalle Co*.: Starved Rock, 9.v.1937, H. Dybas, (FMNH, 1); Dayton, 17.v.1946, F. Werner, (UAIC, 1); same locality, 17.v.1946, F. Werner, (UAIC, 1).

*McHenry Co*.: Algonquin, Nason, (INHS, 1); Funks Grove, 23.iv.1926, (FMNH, 2); same locality, 8.v.1883, (INHS, 1).

*St. Clair Co*.: Kahokia, 2.ix.1919, G.W. Bock, (UMRM, 1).

*Union Co*.: Pine Hills, Wolf Lake, 25.i.1973, (FSCA, 3).

*Wabash Co*.: Beall Woods N.P., 5.iv.1996, M.A. Goodrich, sifted from woods trash, (EIUC, 1); same locality, 16–23.v.1996, M.A. Goodrich, malaise trap, (EIUC, 1); same locality, 22–23.vi.1995, M.A. Goodrich, UV light trap, (EIUC, 1).

*Counties unknown*: no other data, (INHS, 23); 17.vi.1929, B. Benesh, (INHS, 3); v, (INHS, 1); N. Illinois, (CAS, 1); Ulke collection, (CMNH, 1).

**INDIANA**

no other data, (UBC, 1).

*Montgomery Co*.: Shades State Park, 29.vi.1991, J. & M. Huether, (FSCA, 1).

*Parke Co*.: 8.v.1955, N.M. Downie, (FMNH, 1).

*Porter Co*.: Indiana Dunes N.L. Beverly Shores, 28.vi-17.vii.1995, R. Grundel, fit, (DAPC, 1); same locality, 9.xi.1995, R. Grundel, fit, (DAPC, 1); same locality, 11.ix-19.x.1995, R. Grundel, fit, (DAPC, 2); same locality, 28.vi-17.vii.1995, R. Grundel, flight intercept trap, (DAPC, 1); same locality, 11.ix-19.x.1995, R. Grundel, flight intercept trap, (DAPC, 2); same locality, 14–28.vi.1995, R. Grundel, flight intercept trap, (DAPC, 2); Indiana Dunes N.L., Inland Marsh, 14–28.vi.1995, R. Grundel, flight intercept trap, (DAPC, 1); same locality, 14–28.vi.1995, R. Grundel, fit, (DAPC, 1); Indiana Dunes N.L., Miller Woods, 27.vi-17.vii.1995, R. Grundel, flight intercept trap, (DAPC, 2); same locality, 17.vii-16.viii.1995, R. Grundel, flight intercept trap, (DAPC, 1); same locality, 27.vi-17.vii.1995, R. Grundel, fit, (DAPC, 2); Jasper-Pulaski W.A., Tellt Savanna, 27.vi-18.vii.1995, R. Grundel, fit, (DAPC, 1).

*Tippecanoe Co*.: 25.vi.1963, N.M. Downie, (FMNH, 1); 22.vi.1955, N.M. Downie, (FMNH, 1); 5.viii.1964, N.M. Downie, (FMNH, 1); 6.vi.1966, N.M. Downie, (FMNH, 1); 5.vi.1973, N.M. Downie, (FSCA, 1); 3.viii.1973, N.M. Downie, (FSCA, 1); 9.vi.1986, N. M. Downie, u-v light, (UGAC, 2); 7.ix.1983, N. M. Downie, (AMNH, 3); 22.vi.1983, N. M. Downie, (AMNH, 3); 22.vi.1955, N. M. Downie, (USNM, 13); Lafayette, 22.xi.1984, N. M. Downie, uv light, (DENH, 1); same locality, 6.viii.1984, N. M. Downie, uv light, (DENH, 1); same locality, N. M. Downie, uv light, (PKLC, 1).

*Vigo Co*.: Terre Haute Blatch, Wickham, (USNM, 1).

**KANSAS**

no other data, (MCZ, 1).

*Douglas Co*.: Lawrence, 20.iv.1938, W.J. Brown, (CNC, 1); 900’, F.H. Snow, (CUIC, 1); Lawrence, KU Nat. Res., 23.v.1991, C.W. Young, (CMNH, 1).

**KENTUCKY**

*Butler Co*.: 11.v.1970, J.M. Campbell, (CNC, 1).

*Henderson Co*.: Henderson, 15.v.1924, M.Y. Marshall, (UMMZ, 1).

**LOUISIANA**

*Natchituches Par*.: Vowell’s Mill, (CUIC, 3).

**MARYLAND**

Ulke collection, (CMNH, 5).

*Anne Arundel Co*.: E. Riverdale, 9.ii.1913, W.W. Wallis, (USNM, 2).

*Charles Co*.: Indian Head, 16.ii.1945, George B. Vogt, under bark of old pine stump, (USNM, 2); 3 Mi. W. Crossroads, 15.I.1972, M. Drunkenbrod, under bark, (USNM, 1).

*Dorchester Co*.: Church Creek, 23.vi.1984, F. Guarnieri, at light, (CUIC, 1).

*Montgomery Co*.: 19.ix.1911, E. Shoemaker, (USNM, 1); 18.ix.1913, E. Shoemaker, (USNM, 1); Gaithersburg, 25.v.1972, W.E. Steiner, (USNM, 1); Poolesville, 5.xi.1979, W.E. Steiner, (USNM, 2); Plummer’s I, 22.iv.1903, E.A. Schwarz, (USNM, 1).

*Prince George’s Co*.: Cheverly 3856N 7655W, 17.xi.1991, W.E. Steiner & J.M. Swearingen, (USNM, 1); Watkins Park, 3 km e. Largo, 4.i.1987, W.E. Steiner 7 M.J. Molineaux, In *Peromyscus* nest debris under bark of dead standing *Liriodendron*, (USNM, 1); 2 mi. W. Brandywine, 17.iv.1971, J. Powell, (UCBC, 2); Bladenbg, 26.iii.1903, A.N. Caudell, (USNM, 1); same locality, 27.v, H.S. Barber, (USNM, 1).

*Talbot Co*.: Wittman, 13.iii.1982, W.E. Steiner, (USNM, 1); same locality and collector, 19.vii.1980, (USNM, 1).

*Frederick/ Harford/ Montgomery and/or Prince George’s Co*.: Edgewood, 17.xii.1918, H. Dietrich, (CUIC, 1).

**MASSACHUSETTS**

*Counties unknown*: Ch’nt, v, (USNM, 1).

**MICHIGAN**

no other data, (FMNH, 5).

*Berrien Co*.: T 7S, R 20W, Sec. 20, 21.v.1966, E. Metzler, (FMNH, 1); PawPaw, 13.iv, Hubbard & Schwarz, (USNM, 2).

*Charlevoix Co*.: Beaver Isl., 8.vii.1924, S. Moore, (UMMZ, 2); High Isl., 5.x.1923, S. Moore, (UMMZ, 1).

*Gogebic Co*.: Hughitt-Rawson Preserve, 7.viii.1919, T.H. Hubbell, (UMMZ, 1).

*Ingham Co*.: East Lansing, 8.v.1975, J. Liebherr, (CUIC, 1).

*Kalamazoo Co*.: Gull Lake Bio. Sta., 2.vii.1963, R.L. Fischer, (MSUC, 1).

*Kent Co*.: Grand Rapids, (FMNH, 1).

*Lapeer Co*.: Deerfield Twp., 2.vi.1945, G. Steyskal, (UCRC, 2).

*Leelanau Co*.: South Fox Isl., 5.vi.1925, S. Moore, (UMMZ, 2).

*Livingston Co*.: E.S. George Reserve, 12.vii.1931, A.W. Andrews, (UMMZ, 1); same locality, 10.vii.1989, B.M. Oconnor, Malaise trap, (UMMZ, 1).

*Macomb Co*.: E. of Memphis, 30.iv.1973, Brivio, (MSUC, 1).

*Midland Co*.: 16.v.1948, R.R. Dreisbach, (MSUC, 1).

*Monroe Co*.: Petersburg, 2.ix.1965, J. Truchan, (MSUC, 1); T6S, R6E, Sec. 35, 7.viii.1965, J. Truchan, (MSUC, 1); same locality, 13.viii.1965, J. Truchan, (MSUC, 3); same locality, 26.vii.1965, J. Truchan, (MSUC, 3).

*Oakland Co*.: Southfield, 12.iv.1925, S. Moore, (UMMZ, 1); county record only, 4.vii.1930, A.W. Andrews, (UMMZ, 2); 14.vi.1931, A.W. Andrews, (UMMZ, 13); 21.vii.1921, A.W. Andrews, (UMMZ, 1).

*St. Joseph Co*.: Three Riv., Hubbard & Schwarz, (USNM, 2); Harwood Reserve, 31.iii.1957, D.C.L. Gosling, (UMMZ, 1).

*Shawassee Co*.: 18.v.1952, R.R. Dreisbach, (MSUC, 2).

*Washtenaw Co*.: 21.v.1924, S. Moore, (UMMZ, 1).

*Wayne Co*.: [Det.], viii, Hubbard & Schwarz, (USNM, 2); Detroit, Schwarz, (MCZ, 1); same locality, (AMNH, 3); same locality, Hubbard & Schwarz, (USNM, 3); 8 Mile Road, 1.v.1921, S. Moore, (UMMZ, 1).

*Counties unknown*: Ag Coll, H.E. Weed, (AMNH, 2); Ag. College, 22.iii.1928, (MSUC, 1).

**MINNESOTA**

*Crow Wing Co*.: Brainerd, 17.vi.1962, E.J. Kiteley, (CNC, 1).

*Fillmore Co*.: Preston, 25.v.1948, S. Parfin, (USNM, 1).

*Hennepin Co*.: Minneapolis, 21.ix.1958, A. Raske, fungus on oak, (CNC, 2); same locality, E.J. Kiteley, (CNC, 1).

*Sherburne Co*.: Elk River, 27.vi.1955, E.J. Kiteley, (CNC, 1).

*Washington Co*.: Afton, 7.v.1960, A. Raske, rotten log, (CAS, 1); same locality, 7.v.1960, A. Raske, rotten log, (CNC, 2); same locality, 7.v.1960, A. Raske, rotten log, (UCBC, 1).

**MISSISSIPPI**

*George Co*.: 7 mi. SSE Lucedale, 26.v.1987, P.K. Lago, (UMIC, 1).

*Pearl River Co*.: Picayune, 24.viii.1897, (UMRM, 1).

**MISSOURI**

*Boone Co*.: McBaine, 11.vi.1979, E.G. Riley, (EGRC, 1).

*Dent Co*.: 16 mi. SE Salem, 16.ii.1972, S.E. Thewke, (UMRM, 1); Salem, 20.v.1957, Buchanan, (FSCA, 1); same locality, 2.vi.1958, Holt, (FSCA, 1).

*Franklin Co*.: 1.vii.1967, F.J. Moore, (OSUC, 1).

*Randolph Co*.: 1 mi. E. Moberly, 22.vii.1972, E.G. Riley, (EGRC, 1); same locality, 1.vii.1973, E.G. Riley, (EGRC, 1); same locality, 13.viii.1972, E.G. Riley, (EGRC, 1); same locality, 19.vii.1976, E.G. Riley, (EGRC, 1); Rudolph Bennett Wildlife Area, 22.iv.1975, E.G. Riley, under bark, (EGRC, 1).

*St. Charles Co*.: St. Charles, 8.x.1902, G.W. Bock, (UMRM, 1).

**MONTANA**

*Dawson Co*.: Intake diversion, 27.vi.1988, D.L. Gustafson, uv light, (MTEC, 2); Glendive, Wickham (USNM, 3).

*Lake Co*.:Yellow Bay Bio. Station 47.8767°N, 114.0302°W, 30.vi-25.vi.2010, 2930 ft, Kraus; Bait Lin.Fnl, (MTEC, 2).

*Lincoln Co*.: Yaak River Mouth, 9.vi-12.ix.1994, D.L. Gustafson, funnel traps, (MTEC, 9); Hwy 95, Trego 48.7324°N, 114.8353°W, 26.vii-2.x.2010, 3120 ft, Kraus et al., Bait. LinFnl, (MTEC, 2); Rest stop at inter. of 2 and 56, W. of Libby, elev. 657 m, 48.43595 N, 115.85001 W, 2.vii-2.x.2010, Kraus, Etzler, Ivie, sticky trap, EAB#29a, (MTEC, 1).

*Missoula Co*.: Milltown, elev. 1008 m, 46.8727N, 113.8664 W, 2.vi-31.ix.2010, L Funnel w/ AP, EtOH and sirex, USDA, (MTEC, 1).

*Ravalli Co*.: Bitterroot R. - Darby, 2.vii.1991, D.L. Gustafson, (MTEC, 2).

*Rosebud Co*.: Ashland, 6.viii-12.x.1991, D.L. Gustafson, pitfall trap, (MTEC, 1); Rosebud - Far West, 13.vii.1991, D.L. Gustafson, (MTEC, 2); Tongue R. - Ashland, 6.vi.1991, D.L. Gustafson, (MTEC, 1).

*Sanders Co*.: N. Snider, Plum Crk. Rd 47.6147°N, 115.1830°W, 3.vii-1.x.2010, 3190 ft., Kraus et al.; bait. LinFnl, (MTEC, 5); White Pine, Beaver Crk 47.7167°N, 115.4800°W, 27.vii-1.x.2010, 2593 ft., P. Kraus et al.; bait. LinFnl, (MTEC, 1).

**NEBRASKA**

*Douglas Co*.: Omaha, 25.vi.1998, C.A. Springer, uv light, (EIUC, 1).

*Sarpy Co*.: fontenelle For., 23.viii.-6.ix.1997, M.A. Goodrich & C.A. Springer, malaise trap, (EIUC, 1).

**NEW HAMPSHIRE**

no other data, (MCZ, 1).

*Carroll Co*.: 2 mi NW Wonalancet, 8–14.vi.1984, D.S. Chandler, window trap, (DENH, 1).

**NEW JERSEY**

*Burlington Co*.: Medford Township Lake Pine, 11.ix.1974, D.C. Rentz, (USNM, 1).

*Gloucester Co*.: Malaga, 1.vi.1907, Geo M. Greene, (USNM, 1); Westville, (MCZ, 2).

**NEW** **MEXICO**

*San Miguel Co*.: Santa Fe Nat. For., Holyghost cmpgrnd, 16 mi. N. Pecos, 35°46'07.8", 105°42'03.5", 18–19.vi.2009, coll. at night on dead standing ponderosa pine, D.A. Pollock, (DAPC, 1).

**NEW YORK**

no other data, (CUIC, 3); no other data, (MCZ, 2); no other data, (NDSU, 3).

*Erie Co*.: Buffalo, (INHS, 2); same locality, (MCZ, 1); Buffalo, Fort Erie, 23.ix.1877, (MCZ, 1).

*Essex Co*.: Mt. Marcy, top, 1.ix.1942, H. Dietrich, (CUIC, 1).

*Genesee Co*.: Batavia, 2.vii.1913, H.H. Knight, (USNM, 3).

*Niagara Co*.: Olcott, 18.vi.1922, H. Dietrich, (CUIC, 9); same locality, 15.viii.1922, H. Dietrich, (CUIC, 2); same locality, 30.viii.1925, H. Dietrich, (CUIC, 1); same locality, 6.viii.1922, H. Dietrich, (CUIC, 4); same locality, 18.vi.1922, H. Dietrich, (MCZ, 1; UMMZ, 1); same locality, 15.viii.1921, H. Dietrich, (UAIC, 1); same locality, 15.viii.1922, H. Dietrich, (UAIC, 1).

*Onondaga Co*.: 5.vi.1942, N.M. Downie, (FMNH, 1); Westvale, 27.ii.1965, D.L. Stephan, in dead tree, (CUIC, 1).

*Orleans Co*.: Medina, 27.v.1955, L.L. Pechuman, (CUIC, 1); Lakeside Beach State Park, N. Watesport, 30.viii.1984, L. Herman, (AMNH, 4).

*St. Lawrence Co*.: Canton, 24.vii.1941, N.M. Downie, (FMNH, 4); same locality, 19.v.1935, N.M. Downie, (FMNH, 1).

*Tompkins Co*.: 27 Perry City Road, Ithaca, 16.vi.1981, G. Swenson, light trap, (CARR, 1). Ithaca, 2.i.1897, J.O. Martin, (CUIC, 1); same locality, 19.v.1966, D. Stephan, on attic window, (CUIC, 1); same locality, 6.vi.1951, V.M. Kirk, (NDSU, 1); same locality, 26.vi.1949, T.J. Spilman, (USNM, 1); Ithaca, Cascadilla Cr., 3.ix.1921, (CUIC, 1).

**NORTH CAROLINA**

no other data, (CMNH, 1); no other data, (MCZ, 4); no other data, (USNM, 2).

*Durham Co*.: Duke Forest, Durham, vi.1970, S. Hughes-Schrader, at black light, (UCRC, 2).

*Moore Co*.: Southern Pines, 13.iii.1911, A.H. Manee, (INHS, 1); same locality, 11.ii.1916, (AMNH, 1).

*Stokes Co*.: Hanging Rock St. Pk., 20 mi. N. Winston-Salem, 8–10.ix.1967, J.M. & B.A. Campbell, (CNC, 3).

*Wake Co*.: Raleigh, 5.iii.1953, H. & A. Howden, dead oak, (CMNC, 1). *Counties unknown*: Tyron, W.F. Fiske, (USNM, 1).

**NORTH DAKOTA**

*Pembina Co*.: T161N, R56W, Sec. 22, 25.vi.1975, P.K. & B.A. Lago, in fungi, (PKLC, 1).

*Richland Co*.: 6.v.1964, Robert Gordon, (USNM, 1).

**OHIO**

*Athens Co*.: 1.x.1954, (USNM, 1).

*Butler Co*.: Fairfield Barnebey Center, 29.iv.1986, (DAPC, 1).

*Clinton Co*.: 9.vi.1963, F.J. Moore, (OSUC, 1); 25.vi.1962, F.J. Moore, (OSUC, 1); 16.iii.1963, F.J. Moore, (OSUC, 1); 21.vi.1962, F.J. Moore, (OSUC, 1); 26.vi.1962, F.J. Moore, (OSUC, 1); 7.v.1965, F.J. Moore, (OSUC, 1); 1.viii.1961, F.J. Moore, (OSUC, 1).

*Delaware Co*.: 21.v, D.J. & J.N. Knull, (OSUC, 6); 8.viii.1947, D.J. & J.N. Knull, (OSUC, 2).

*Hocking Co*.: Conkles Jollow St. Pk., 26.v.1980, M.A. & L.L. Ivie, (MTEC, 1).

*Ottawa Co*.: S. Bass Isl., Fishery Bay, 2.vii.1983, S.W. Nichols, uv light trap, (CUIC, 1).

*Portage Co*.: Geauga Lake, 17.vi.1926, J.C. Pallister, (AMNH, 1).

*Scioto Co*.: 11.v.1963, F.J. Moore, (OSUC, 1).

*Vinton Co*.: Lake Hope St. Pk., 6–19.viii.1993, R.A. Androw, (DAPC, 3).

*Wayne Co*.: Wooster, 18.vi.1941, R. Rings, bait trap, (CAS, 1); same locality, 22.vi.1941, R. Rings, (CAS, 1).

*Wyandot Co*.: Sycamore, 4.iv.1917, P.S.Bartholomew, (CAS, 2); same locality, 1917, P.S.Bartholomew, (CAS, 1).

*Counties unknown*: no other data, (FMNH, 2); no other data, (UBC, 1). Ulke collection, (CMNH, 1).

**OKLAHOMA**

*Latimer Co*.: v.1983, K. Stephan, (WFBM, 1); xi.1982, K. Stephan, (WFBM, 1); 5 mi. W. Red Oak, v.1980, K. Stephan, (FSCA, 1); same locality, 27.iv.1978, K.H. Stephan, (FSCA, 1); same locality, iv.1981, K. Stephan, (FSCA, 1).

*Oklahoma Co*.: Oklahoma City, 1966, W. Rosenberg, (USNM, 1).

**OREGON**

*Benton Co*.: Corvallis, 9.iii.1936, E. Gray & J. Schuh, (AMNH, 1).

*Crook Co*.: Prineville, 28.vii.1998, alpha-pinene & ethanol lure, lindgren funnel trap, (ODAC, 1); same locality, 8.ix.1998, alpha-pinene & ethanol lure, lindgren funnel trap, (ODAC, 1); same locality, 8.ix.1998, Ips lure; Lindgren funnel trap, (ODAC, 8).

*Douglas Co*.: 7 mi. NW Roseburg, 12.vi.1966, J.D. Vertrees, lite, (AMNH, 1); same locality, 8.viii.1964, J.D. Vertrees, lite, (AMNH, 1).

*Harney Co*.: Alvord, 3.vi.1960, H. Foster, (ODAC, 1).

*Jackson Co*.: 6 mi N. Medford, a965, Cliff Cordy, lite, (AMNH, 1); Medford, 16.v.1970, (ODAC, 1).

*Lane Co*.: Eugene, 5.viii.1966, K. Goeden, blk. light trap, (ODAC, 1); same locality, 12.vii.1968, black light trap, (ODAC, 1); same locality, 7.viii.1941, B. Malkin, (FMNH, 1); Oakridge, 18.ix.1964, S. McCleve, (SMC, 1).

*Linn Co*.: Sweet Home, 16.vi.1998, alpha-pinene & ethanol lure, lindgren funnel trap, (ODAC, 2); same locality, 13.vii.1998, alpha-pinene & ethanol lure, lindgren funnel trap, (ODAC, 1).

*Multnomah Co*.: Portland, N. Columbia Blvd., 30.iv.1998, Ips lure; Lindgren funnel trap, (ODAC, 2); same locality, 6.vii.1998, alpha-pinene & ethanol lure, lindgren funnel trap, (ODAC, 1); same locality, 30.iv.1998, alpha-pinene & ethanol lure, lindgren funnel trap, (ODAC, 1); same locality, 18.vi.1998, alpha-pinene & ethanol lure, lindgren funnel trap, (ODAC, 1); same locality, 5.vi.1998, Ips lure; Lindgren funnel trap, (ODAC, 1); Portland, Terminal 2, 19.viii.1998, Ips lure; Lindgren funnel trap, (ODAC, 1).

*Wallowa Co*.: 20 mi. N. Homestead, 6.vi.1959, K. Goeden, under bark, (ODAC, 1).

*Yamhill Co*.: 6 mi. NE Newberg, 23.viii.1969, black light trap, (ODAC, 1).

*Counties unknown*: Salem, 24.vi.1960, K. Goeden, black light trap, (ODAC, 2).

**PENNSYLVANIA**

Ulke collection, (CMNH, 1); no other data, (CUIC, 1); no other data, (MCZ, 1).

*Allegheny Co*.: 24.viii.1895, E.A. Klages, (CUIC, 4); (CMNH, 5); 20.x, (CMNH, 2); Pittsburgh, (CMNH, 4); same locality, 7.vi, (CMNH, 1); same locality, 5.viii.1924, (CMNH, 1); same locality, vii, (CMNH, 1); same locality, 21.vi, (CMNH, 1); same locality, 1.v, (CMNH, 1); same locality, 16.viii, (CMNH, 1); same locality, vi, (CMNH, 1); same locality, 12.v, (CMNH, 1); same locality, 23.vii, (CMNH, 1); Allegheny, (CMNH, 3); same locality, (CNC, 4); same locality, (MCZ, 2); Aspinwall, 19.ix.1926, (CMNH, 1).

*Tioga Co*.: 4 km SW Tioga 4153N 7711W, 22.viii.1992, W.E. Steiner & J.M. Swearingen, (USNM, 1).

*Westmoreland Co*.: Jeannette, 2.ix, H.G. Klages, (CMNH, 1); same locality, (CMNH, 3); same locality, 3.ix, (CMNH, 6); same locality, 22.vi, H.G. Klages, (CMNH, 1); same locality, 27.v, (CMNH, 1); same locality, 14.vi, (CMNH, 1); same locality, 14.vii, H.G. Klages, (CMNH, 1); same locality, 4.vii, H.G. Klages, (CMNH, 2); same locality, 2.vi, (CMNH, 1); same locality, ix, (CMNH, 1); same locality, 14.vii, H.G. Klages, (CMNH, 1); same locality, v, (CMNH, 1); same locality, 2.vi, (CMNH, 1); same locality, 22.v, H.G. Klages, (CMNH, 1); same locality, 10.vi, H.G. Klages, (CMNH, 1); same locality, vii, (CMNH, 1).

*Counties unknown*: Mt. Lebanon, ix.1930, R.J. Simon, (CMNH, 7); St. Vinc., (CMNH, 4).

**SOUTH** **CAROLINA**

*Anderson Co*.: Pendleton, 250 m, 1–7.vi.1987, BRC Hym. team, malaise in mature hardwood forest, (CNC, 1).

*Florence Co*.: Florence, 21.vi.1962, V.M. Kirk, stale molasses trap, (NDSU, 1); same locality, 24.ii.1959, V.M. Kirk, trash in hollow tree, (NDSU, 1); same locality, 27.x.1961, V.M. Kirk, hollow tree, (NDSU, 2); same locality, 22.ix.1959, V.M. Kirk, at wound on oak trunk, (NDSU, 1); same locality, 7.v.1962, V.M. Kirk, Tanglefoot Screen, (USNM, 1).

*Pickens Co*.: Clemson, 30.ix.1951, W.F. Chamberlain, under bark, (TAMU, 1).

*Union Co*.: Union, 7.iii.1961, (USNM, 1).

**TENNESSEE**

*Shelby Co*.: Memphis, 20.iv.1955, (CNC, 1).

**TEXAS**

no other data, (MCZ, 2).

*Angelina Co*.: Angelina Nat’l. Forest, ca. 2.5 mi. SE Zavalla, 31°02'54"N, 94°23'14"W, 19.iv-2.v.1996, Clarke, Menard & Riley, hardwood bottom land, (TAMU, 1); same locality, 2–16.v.1996, Clarke, Menard & Riley, hardwood bottom land, (TAMU, 1); same locality, 31.v-13.vi.1996, Clarke, Menard & Riley, hardwood bottom land, (TAMU, 1); Angelina Nat'l. Forest, ca. 3 mi. NE Rockland, 31°03'19"N, 94°22'06"W, 2–16.v.1996, Clarke, Menard & Riley, unmanaged longleaf pine, (TAMU, 2); same locality, 19.iv-2.v.1996, Clarke, Menard & Riley, unmanaged longleaf pine, (TAMU, 2); same locality, 31.v-13.vi.1996, Clarke, Menard & Riley, unmanaged longleaf pine, (TAMU, 6); Angelina Nat'l. Forest, ca. 4 mi. NE Rockland, 31°07'40"N, 94°21'57"W, 2–16.v.1996, Clarke, Menard & Riley, unmanaged loblolly pine, burned 1996, (TAMU, 2); same locality, 31.v-13.vi.1996, Clarke, Menard & Riley, unmanaged loblolly pine, burned 1996, (TAMU, 1); Angelina Nat'l. Forest, ca. 5.5 mi. SE Zavalla, 31°06'59"N, 94°20'04"W, 31.v-13.vi.1996, Clarke, Menard & Riley, unmanaged loblolly pine, (DAPC, 2); same locality, 2–16.v.1996, Clarke, Menard & Riley, unmanaged loblolly pine, (TAMU, 8); same locality, 17–30.v.1996, Clarke, Menard & Riley, unmanaged loblolly pine, (TAMU, 2); same locality, 31.v-13.vi.1996, Clarke, Menard & Riley, unmanaged loblolly pine, (TAMU, 4); same locality, 31.v-13.vi.1996, Clarke, Menard & Riley, managed loblolly pine, (DAPC, 2); Angelina Nat'l. Forest, ca. 5.5 mi. SE Zavalla, 31°07'05"N, 94°20'01"W, 17–30.v.1996, Clarke, Menard & Riley, managed loblolly pine, (DAPC, 1); same locality, 2–16.v.1996, Clarke, Menard & Riley, managed loblolly pine, (TAMU, 5); same locality, 31.v-13.vi.1996, Clarke, Menard & Riley, managed loblolly pine, (TAMU, 4); Angelina Nat'l. Forest, ca. 7 mi. NE Rockland, 31°04'39"N, 94°16'40"W, 2–16.v.1996, Clarke, Menard & Riley, managed longleaf pine, (TAMU, 2); same locality, 31.v-13.vi.1996, Clarke, Menard & Riley, managed longleaf pine, (TAMU, 1); same locality, 17–30.v.1996, Clarke, Menard & Riley, managed longleaf pine, (TAMU, 1).

*Brewster Co*.: Big Bend Nat. Pk., North Rosillos Mts., Buttrill Spring, 10–17.vii.1991, R. Vogtsberger, malaise trap, (TAMU, 1).

*Montgomery Co*.: Jones St. Forest, 8 mi. S. Conroe, 12.ix.1987, Wharton, Wang & Praetorius, malaise trap, (TAMU, 1); Willis, [03], (USNM, 2).

*Sabine Co*.: 9 mi. E. Hemphill, “beech bottom”, 10–16.iv.1989, Anderson & Morris, flight intercept trap, (TAMU, 11); same locality, 25.iv-7.v.1989, R. Anderson & E. Morris, flight intercept trap, (TAMU, 1); same locality, 3–9.iv.1989, Anderson & Morris, flight intercept trap, (TAMU, 2); same locality, 17–24.iv.1989, R. Anderson & E. Morris, Malaise trap in beech-magnolia forest, (TAMU, 1); same locality, 13–24.vii.1989, R. Anderson & E. Morris, Malaise trap in beech-magnolia forest, (TAMU, 2); same locality, 22.v-4.vi.1989, R. Anderson & E. Morris, Malaise trap in beech-magnolia forest, (TAMU, 3); same locality, 18–23.vi.1989, R. Anderson & E. Morris, Malaise trap in beech-magnolia forest, (TAMU, 1); same locality, 4–9.iv.1989, R. Anderson & E. Morris, Malaise trap in beech-magnolia forest, (TAMU, 2).

*Walker Co*.: Sam Houston Nat’l. For., Stubblefield Lake Area, 7–14.ix.1996, E.G. Riley, ETOH trap; 386, (TAMU, 1).

**UTAH**

*Washington Co*.: Zion Park, 8/1/1924, C.C. Searl, (UMMZ, 1).

**VIRGINIA**

no other data, (MCZ, 2).

*Alexandria Co*.: Alexandria, 14.ix.1991, Ed Brodie, (USNM, 1).

*Hampton Co*.: Ft. Monroe, 19.iv, Hubbard & Schwarz, (USNM, 1).

*Nelson Co*.: 9.viii.1921, W. Robinson, (USNM, 1).

*Shenandoah Co*.: 16 km S. Strasburg, Elizabeth Furnace, 12.v-22.viii.1983, S. & J. Peck, forest intercept, (CNC, 1).

*Counties unknown*: St. Falls, 17.ix.1913, (USNM, 3).

**VERMONT**

*Franklin Co*.: Swanton, 8.ix.1974, E.J. Kiteley, under bk. of old dead decid. tree, (CNC, 2).

**WASHINGTON**

*Walla Walla Co*.: Walla Walla, 20.vii.1968, W.C. Cook, lite, (AMNH, 1).

**WEST** **VIRGINIA**

*Braxton Co*.: Gassaway, 23.vi.1992, S.F. Hutchinson, LFT, lumber yard, (PPCD, 4); same locality, 31.viii-1.ix.1992, S.F. Hutchinson, Coastal Lumber Yard, LFT, (PPCD, 4); Sutton, 5–11.vii.1994, M.D. Bombardiere, LFT, (PPCD, 1).

*Doddridge Co*.: Smithburg, 23.vi.1992, S.F. Hutchinson, Coastal Lumber Yard, LFT, (DAPC, 1); same locality, 17–18.viii.1992, S.F. Hutchinson, Coastal lumber yard, Lindgren funnel trap, (PPCD, 1); same locality, 8–9.vii.1992, S.F. Hutchinson, LFT, lumber yard, (PPCD, 1); same locality, 20–21.vii.1992, S.F. Hutchinson, Coastal Lumber Yard, LFT, (PPCD, 1); same locality, 23.vi.1992, S.F. Hutchinson, Coastal Lumber Yard, LFT, (PPCD, 1).

*Greenbrier Co*.: Rupert, 31.viii-1.ix.1992, S.F. Hutchinson, LFT, lumber yard, (PPCD, 2); same locality, 20–21.vii.1992, S.F. Hutchinson, LFT, lumber yard, (PPCD, 2); White Sulphur Springs, vii/viii, (CAS, 1; FMNH, 5).

*Hampshire Co*.: 2 mi S. Bloomery, 6–13.vi.1995, W.L. Drosselmeyer, LFT, (DAPC, 1); Forks of Cacapon, 13–20.v.1996, W.L. Drosselmeyer, LFT, (PPCD, 1).

*Jackson Co*.: Evans, 26.vi-6.vii.1990, T. Sutton, LFT, (PPCD, 1).

*Kanawha Co*.: Guthrie, 27–31.v.1988, M.C. Thomas, blacklight trap, (DAPC, 1); same locality, 27–31.v.1988, M.C. Thomas, blacklight trap, (PPCD, 1); same locality, So. Charleston, 1.iii.1980, P.H. Adler, under bark of chestnut oak, (PPCD, 1).

*Lincoln Co*.: Garretts Bend, 13.v.1988, B.E. Hagenbuch, black light trap, (PPCD, 1).

*Mason Co*.: Southside, 26.vi-5.vii.1995, R.A. Meyers, LFT, (PPCD, 1).

*Mineral Co*.: 1 mi. E. Burlington, 14–21.vi.1996, I. Lewis, LFT, (PPCD, 1).

*Nicholas Co*.: Richwood, 31.viii-1.ix.1992, S.F. Hutchinson, Mullican Lumber Yard, LFT, (PPCD, 1); Richwood, Georgia-Pacific Lumber Yard, 8–9.vii.1992, S.F. Hutchinson, LFT, (PPCD, 1).

*Pendleton Co*.: Riverton, 27.v-3.vi.1994, L.S. Mallow, LFT, (DAPC, 1); Upper Tract, 27.v.1994, L.S. Mallow, (PPCD, 1). *Putnam Co*.: 1 mi. SE Winfield, 28.vi-6.vii.1995, R.A. Meyers, LFT, (PPCD, 1); Winfield, 24–31.v.1995, R.A. Meyers, LFT, (DAPC, 1).

*Randolph Co*.: Dailey, Coastal Lumber Yard, 20–21.vii.1992, S.F. Hutchinson, LFT, (DAPC, 1).

*Ritchie Co*.: 2 mi. N. Oxbow, 19–26.v.1995, R.A. Meyers, LFT, (DAPC, 1).

*Roane Co*.: Spencer, 20–21.vii.1992, S.F. Hutchinson, LFT, lumber yard, (DAPC, 1).

*Taylor Co*.: 4 mi. N. Irontown, 4–11.vii.1994, W.L. Drosselmeyer, LFT, (PPCD, 1).

*Webster Co*.: Hacker Valley, 17–18.viii.1992, S.F. Hutchinson, LFT, lumber yard, (DAPC, 1; BYUC, 1); same locality, 31.viii-1.ix.1992, S.F. Hutchinson, LFT, lumber yard, (PPCD, 2); Hacker Valley, Coastal Lumber Yard, 23.vi.1992, S.F. Hutchinson, LFT, (PPCD, 1).

*Wirt Co*.: 2 mi. W. Munday, 19–26.v.1995, R.A. Meyers, LFT, (PPCD, 1).

*Wood Co*.: 1 mi. S. Walker, 19–26.v.1995, R.A. Meyers, Lindgren funnel trap, (PPCD, 1).

**WISCONSIN**

*Dane Co*.: 11.v.1954, D.H. Habeck, (FSCA, 19); 20.v.1950, (WIRC, 1); 6.v.1954, C.L. Fluke, (WIRC, 9); 22.v.1899, (WIRC, 1); 26.x.1900, W.S. Marshall, (WIRC, 1); Belleville, 6.v.1954, L.H. McMullen, ex dead oak stump, (WIRC, 17); Madison , 19.ix.1983, N. Ravdin, (WIRC, 2).

*Racine Co*.: 7.ix.1953, D.H. Habeck, (FSCA, 1).

*Sauk Co*.: Devils Lake, 14.x.1989, B. Anderson, (WIRC, 1).

*Washington Co*.: Cedar Lake, 17.viii.1902, (UMRM, 1); same locality, 13.viii.1902, (UMRM, 1); West-Bend, 17.viii.1902, G.W. Bock, (UMRM, 1).

*Counties unknown*: Mountain, 20.vi.1939, H. Dybas, (FMNH, 2).

### *Eustrophopsis confinis* (LeConte)

**CANADA**

**BRITISH COLUMBIA**

McIntyre Cr., Oliver, 6.vi.1959, R.E. Leech, (CNC, 1); Radium, 4.vi.1953, Carrs, lot 5, (CARR, 3); same locality, 4.vi.1953, Carrs, lot 5, (DAPC, 2); Trinity Valley, 16.viii.1927, J.R.L. Howell, (PFC, 1); Vernon, 11.v.1943, H. Leech, on grass, (UBC, 1); Victoria, (MCZ, 1).

**MANITOBA**

Duck Mtn. Prov. Pk., 19–24.vi.1981, Ashworth, Schwert & Keller, open gravelly mud with sparse vegetation by water’s edge, (DAPC, 1); Riding Mt. Pk., 2.vi.1938, W.J. Brown, (CNC, 1); Sandilands Forest, 15.viii.1993, N.J. Holliday, (JBWM, 1).

**NOVA SCOTIA**

Cumberland Co., Wentworth, 21.v-5.vii.1965, B. Wright, (NSMC, 2).

**ONTARIO**

Constance Bay, 16.v.1933, W.J. Brown, (CNC, 1).

**SASKATCHEWAN**

Road 914, 82 km N. Jct. Hwy. 165, 30.vii.1986, B.F. & J.L. Carr, lot 2, (DAPC, 1).

**UNITED STATES OF AMERICA**

**IDAHO**

no other data, (CUIC, 1).

*Kootenai Co*.: Coeur d’Alene, (CUIC, 1).

*Latah Co*.: Moscow Mt., Section 9 pond, 30.iv.1976, D.F. Veirs, (WFBM, 1).

**MAINE**

*Androscoggin Co*.: Poland, 8.vii.1970, ex. *Polyporus anceps*, (MCZ, 1).

**MICHIGAN**

no other data, (MCZ, 1).

*Mackinac Co*.: St. Ignace, 31.v.1921, S. Moore, (UMMZ, 3).

**MONTANA**

*Carter Co*.: Long Pines near Lantis Springs, 5.vii.1994, D.L. Gustafson, (MTEC, 1).

*Rosebud Co*., Tongue R. Ashland , 11.viii.1991, D.L. Gustafson, (MTEC, 1).

**NEBRASKA**

Ulke collection, (CMNH, 1).

**NEW** **HAMPSHIRE**

*Coos Co*., Mt. Washington, 5–6,000 ft., 11.vii.1895, (MCZ, 1).

*Hillsborough Co*.: Antrim, 23.vi.1933, C.A. Frost, (MCZ, 1).

**SOUTH DAKOTA**

*Pennington Co*.: Willow Cr., Willow Cr. Horse Cmpgd, Hwy 244, 5.vi.1995, Baumann & Huntsman, (BYUC, 1).

**WASHINGTON**

*Stevens Co*.: Colville, 17.v.1969, R. Baker, (TAMU, 1).

**WISCONSIN**

no other data, (CUIC, 1).

### *Eustrophopsis bicolor* (Fabricius)

**BAHAMAS**

Grand Bahama Island, Freeport, 20–27.vi.1987, W.E. Steiner, M.J. &R. Molineaux, (USNM, 2). Freeport 2630N 7841W, 27.x.1995, W.E. Steiner & J.M. Swearingen, under bark of rotting trunk of *Pinus Eliottii* with polypore fungi, (USNM, 4).

**CANADA**

Ulke Collection, (CMNH, 1); Noroton, (AMNH, 1); same locality, (AMNH, 6).

**MANITOBA**

Winnipeg, King’s Park, D.A. Pollock, sitting on underside of fungusy, dead log at night, (DAPC, 1); same locality, 13.viii.1998, D.A. Pollock, sitting on piece of cut wood at night, (DAPC, 1); Winnipeg, Univ. of Manitoba campus, nr. apiary, 49°48'30"N, 97°07'35"W, 27.vii.1999, D.A. Pollock, on thin white fungus on underside of small log, (DAPC, 7); Winnipeg, University of Manitoba campus, along Red River, 25–28.viii.1996, D.A. Pollock, at night, on fungusy stump of *Acer negundo*, (DAPC, 1); Winnipeg, University of Manitoba campus, forest along Red River (south), 11.vii.1999, D.A. Pollock, crawling on fungusy stump of *Acer negundo* at night, (DAPC, 1); Lockport, 50°05'13"N, 96°56'17"W; 19.vii.2010, on dead trees at night along shore of Red River, (DAPC, 2).

**ONTARIO**

Bolton, 4.v.1978, R.S. Anderson, (ROME, 1); Chatham, 23.viii.1929, G.M. Stirrett, (CNC, 1); Dunn Twp, 29.viii.1971, W.W. Judd, (CNC, 1); Dunnville, 24.viii.1938, S.D. Hicks, very teneral, (CNC, 2); Grand Bend, 6.vii.1939, T.N. Freeman, (CNC, 1); Guelph, 23.viii.1990, Heather Dewar, bracket fungus, (DEBU, 4); Lanark, 2.viii.1979, S.D. Hicks, (CNC, 1); same locality, 4.vii.1976, S.D. Hicks, (CNC, 1); Leamington, 18.vi.1940, W.J. Brown, (CNC, 1); same locality, 24.v.1970, T.D. Galloway, (JBWM, 1); Ottawa, 4.ix.1912, Beaulieu, 1 teneral, (CNC, 3); same locality, 2.x.1912, Beaulieu, (CNC, 1); same locality, 14.viii.1912, Beaulieu, (CNC, 1); same locality, 9.ix.1912, Beaulieu, (MCZ, 3); Peel Co., Port Credit, 6–11.viii.1965, I.M. Smith, (ROME, 1); Pelee Is., 3.vii.1931, W.J. Brown, (CNC, 1); Pelee Island, 1.vii.1940, W.J. Brown, slightly teneral, (CNC, 1); Pelee Island, Lake Erie, 17.vii.1981, S.W. Nichols, (CUIC, 4); Pelee Island, marsh on SE shore, 14.viii.1980, S.W. Nichols, flooding shore of sand spit, (CUIC, 1); Point Pelee, 26.vii-4.viii.1999, A. Tesolin, malaise & pan trap 41*59’N;82*27.5W, (DEBU, 2); Prince Edward Co., 21.v.1916, Brimley, (CNC, 1); same locality, 3.vi.1917, Brimley, (CNC, 1); same locality, 31.vii.1938, Brimley, (CNC, 1); same locality, 23.iv.1922, Brimley, (CNC, 1); same locality, 21.v.1916, Brimley, (CNC, 1); same locality, 20.v.1923, Brimley, (CNC, 1); same locality, 7.vii.1946, Brimley, (CNC, 1); same locality, 3.vi.1923, Brimley, (CNC, 1); same locality, 18.ix.1921, Brimley, (CNC, 3); Rondeau Prov. Pk., 2.viii.1982, L. LeSage, ex. *Polyporus*, (CNC, 9); Toronto, R.J. Crew, (CUIC, 2); same locality, 24.viii.1895, R.J. Crew, (CUIC, 1); same locality, 12.ix.1908, R.J. Crew, (ROME, 1); Wheatley, 22.vii.1965, (FSCA, 2); Windsor, 2–16.viii.1982, S. Marshall, (CNC, 1); Essex Co., Point Pelee Natl. Pk. Forested area by W beach, 21.viii.1999, O. Lonsdale, malaise/pan traps, (DEBU, 1); Pt. Pelee, 18.vii.1978, J.M. Cumming, (DEBU, 1); Dundas, 15.vii.1975, J.M. Cumming, (DEBU, 1); Guelph, 25.vii.1977, A.A. Konecny, (DEBU, 3); same locality, 3.viii.1976, Paul R. Heels, (DEBU, 1); same locality, 25.iv.1973, R.E. Roughley, (DEBU, 1); London, 19.v.1975, J.M. Heraty, (DEBU, 1); Pr. Edw. Co., Brimley, 2.vi.1940, (AMNH, 1); same locality, 31.vii.1938, (AMNH, 1); Pt. Pelee Nat. Pk., 13.viii.1983, S. Marshall, C. Logan, &S. Girigsby, (DEBU, 1); Tilbury, 30.ix.1965, Rosenberg collection, (USNM, 1); Toronto, 19.vii.1976, W.A. Attwater, (DEBU, 1); same locality, 10.vii.1977, W.A. Attwater, (DEBU, 1); same locality, 15.viii.1981, Lonny Coote, (DEBU, 1).

**QUEBEC**

Aylmer, 25.v.1934, W.J. Brown, (CNC, 1); Chambly, Longueuil, 14.viii.1976, S. Laplante, in rotting Pleurote, (SLC, 2); Deux-Montagnes, Parc Paul-Sauvé, 29.iv.1990, S. Laplante, (SLC, 1); Gatineau Pk. nr. Meach L., 1.ix.1982, A. Davies, berlese fleshy fungi on tree, (CNC, 1); Gatineau, Aylmer, 28.v.1991, S. Laplante, on trunk of dead *Ulmus americana* at 22:00, (SLC, 2); Huntingdon, Havelock, 14.v.1981, S. Laplante, (SLC, 1); Lacolle, 10.viii.1945, W.J. Brown, (CNC, 4); Missisquoi, Scottsmore, 9.vi.1979, S. Laplante, (SLC, 1); Montreal, 5.viii.1985, E.J. Kiteley, uv light trap, (CNC, 1); same locality, 16.vii.1967, E.J. Kiteley, (CNC, 1); same locality, 13.vi.1984, E.J. Kiteley, uv light trap, (CNC, 2); same locality, 2.ix.1969, E.J. Kiteley, (CNC, 1); same locality, 26.vi.1967, E.J. Kiteley, (CNC, 1); same locality, 28.v.1967, E.J. Kiteley, (CNC, 1); same locality, 17.ix.1972, E.J. Kiteley, (CNC, 1); same locality, 3.vii.1970, E.J. Kiteley, under bark, (CNC, 1); same locality, 6.vii.1976, E.J. Kiteley, UV light trap, (CNC, 1); same locality, 23.vi.1968, E.J. Kiteley, on log, (CNC, 1); same locality, 28.viii.1972, E.J. Kiteley, uv light trap, (CNC, 1); same locality, 8.vii.1978, E.J. Kiteley, uv light trap, (CNC, 1); same locality, 28.vii.1980, E.J. Kiteley, uv light trap, (CNC, 1); same locality, 17.vii.1977, E.J. Kiteley, uv light trap, (CNC, 1); Rigaud For. at Ottawa River, 11.v.1988, A. & Z. Smetana, (CNC, 2) Vaudreuil, Rigaud, 21.v.1982, P. Bélanger, under bark of dead maple, (CMNC, 1).

**UNITED STATES OF AMERICA**

**ALABAMA**

no other data, (CMNH, 4).

*Colbert Co*.: Wilson Dam, 15.vii.1953, R.X. Schick, (LACM, 1).

*Greene Co*.: Knoxville, 1.viii.1950, B.D. Valentine, (DAPC, 1).

*Jefferson Co*.: Birmingham, 9.vi.1953, (FMNH, 1); same locality, 13.vi.1955, H.R. Steeves Jr., at light, (FMNH, 1); same locality, 3.vi.1957, H.R. Steeves Jr., at light, (FMNH, 1).

*Lee Co*.: Auburn, 26.iv.1966, E.J. Kiteley, under bark, (CNC, 2); same locality, 20.viii.1969, E.J. Kiteley, (CNC, 1); same locality, 19.iv.1966, E.J. Kiteley, (CNC, 1).

*Madison Co*.: Monte Sano St. Pk. Huntsville, 6.vi.1975, Rosenberg collection, (USNM, 1).

*Mobile Co*.: Mobile, (MCZ, 1); same locality, vi.1927, Darlington, (MCZ, 1); same locality, 17.iv.1948, W. Rosenberg, (USNM, 1).

*Tuscaloosa Co*.: Tuscaloosa, 29–31.iii.1948, (UASM, 1).

**ARKANSAS**

Stromberg Colln., (INHS, 3).

*Faulkner Co*.: Camp J. Robinson, 25.viii.2003, FIT, B. Baldwin, (BBC, 1); same locality, 2.ix.2003, B. Baldwin, flight intercept trap, (BBC, 1).

*Fulton Co*.: Salem, 23.vi.1983, Carrs, lot 7, (CARR, 1); Salem, 23.vi.1983, Carrs, lot 8, (CARR, 7); same locality, 23.vi.1983, Carrs, lot 8, (DAPC, 1).

*Garland Co*.: Camp Clear Fork 3 mi. W. of Crystal Springs off Hwy 270, 20.vi.1986, R.A.B. Leschen, ex polypore, emerged 4 July, (RLC, 1).

*Hempstead Co*.: Hope, 25.iv.1922, (CUIC, 2); same locality, 1921, (CUIC, 1); same locality, 10.xii.1921, (CUIC, 2); same locality, 18.iii.1922, L. Knubel, (UMMZ, 1).

*Johnson Co*.: Ozark Nat. Forest, Haw Creek Falls cmpground on Hwy 123, check notes, D.A. Pollock, under bark of *Fagus*, (DAPC, 1).

*Polk Co*., S. of Board Camp, R29W, T3S, SE Sec. 22, 4–5.ix.1983, C.B. Barr, at blacklight, (CBC, 1).

*Pulaski Co*., Pinnacle Mt. St. Pk., Base Trail, 11.v.1986, J.M. Campbell, ex flood debris on bank, (CNC, 1); Pinnacle Mtn. St. Pk., 11.v.1986, D.A. Pollock, under bark, (DAPC, 4); Little Rock, B. Baldwin (all following): 10.vii.1998, Malaise trap, (BBC, 1); 16.v.2000, Malaise trap, (BBC, 1); 3.vii.1998, Malaise trap, (BBC, 1); 26.iv.1999, flight intercept trap, (BBC, 1); 29.viii.1999, Malaise trap, (BBC, 1); 7.ix.1998, Malaise trap, (BBC, 1); 8.xi.1998, Malaise trap, (BBC, 1); 5.ix.2002, (BBC, 1); 14.v.1998, Malaise trap, (BBC, 1); 13.v.1999, Malaise trap, (BBC, 1); 19.viii.2001, blacklight trap, (BBC, 1); 21.ix.1998, Malaise trap, (BBC, 1); 8.vii.2001, Malaise trap, (BBC, 1); 7.viii.1999, Malaise trap, (BBC, 1); 7.x.1999, Malaise trap, (BBC, 1); 6.vi.1998, Malaise trap, (BBC, 1); 24.viii.1996, ex. polypore fungi, (DAPC, 1).

*Washington Co*., 29.iv.1938, M.W. Sanderson, (INHS, 3); Fayetteville, 12–20.vi.1973, E.V. Gage, (TAMU, 2); Lk. Wedington, 6.v.1986, R.A.B. Leschen, *Trametes versicolor*, (RLC, 1); same locality, 14.vii.1986, R.A.B. Leschen, *Meripilus giganteus*, (RLC, 1); Savoy, 19.x.1974, A. Newton, polypore tree fungus, (MCZ, 4).

*Counties Unknown*.: South West, Chas Palm, (AMNH, 1).

**ARIZONA**

*Santa Cruz Co*.: Patagonia, 30.viii.1975, L. Bezark, G. Nishida, C. Kitayama, B. Tilden, (DAPC, 2); same locality and collectors, 25–26.viii.1975, (DAPC, 1; USNM, 1).

**CALIFORNIA**

Ulke Collection, (CMNH, 1).

*Trinity Co*.: Carrville, 12.v.1918, (FMNH, 1).

*Counties unknown*.: So. Calif., 1–3.iv.1927, (CUIC, 1).

**COLORADO**

Ulke Collection, (CMNH, 1).

*Larimer Co*., Ft. Collins, 4.vii.1989, J. Doyen, bl, (UCBC, 2); Ft. Collins, 18.ix.1975, D. Wagner, (UCBC, 1); Ft. Collins Fossil Creek Meadows, 13.vii.1988, P. Opler, bl, (UCBC, 1); Ft. Collins, Mail Crk., 8.viii.1988, P.A. Opler, bl, (UCBC, 1).

**CONNECTICUT**

*Fairfield Co*.: Darien Woodland Park, 8.iii.1975, F. Mantlik, under rotting oak bark, (UCMS, 7); Milford, 14.iii.1919, K.F. Chamberlain, (CUIC, 1).

*Litchfield Co*.: Cornwall, 13.viii.1920, Chamberlain, (CUIC, 8); Cornwall Bridge, 7.iv.1979, P.W. Kovarik & T.J. Kring, (TAMU, 2).

*New Haven Co*., Hamden (28 Robert St.) 41°21'43"N, 72°55'32"W, 18.v.1986, M.K. Oliver, at white incand. Light, (MKOC, 1); same locality, 11.v.1985, M.K. Oliver, in litter at base of dying *Ailanthus*, (MKOC, 1); same locality, 1–8.viii.1985, M.K. Oliver, vane trap 50% ETOH, (MKOC, 1); same locality, 13.vi.1988, M.K. Oliver, at porch light, (MKOC, 1); same locality, 29.v.1986, M.K. Oliver, at white incand. Light, ~ 9 PM, (MKOC, 1); New Haven (West Rock Ridge St. Pk.) approx. 41°21'N, 72°58'W, 28.vi.1986, M.K. Oliver, in large, orange polypore shelf fungus (? *Polyporus sulphureus*) on standing tree trunk, (MKOC, 1) North Branford (Northford: 22 Foote Hill Rd.) 41°23'23"N, 72°48'15"W, 26.vii.1990, M.K. Oliver, in jack-o-lantern fungus, *Omphalotus olearius* (DC. Ex Fr.) Sing., (MKOC, 1); North Branford (Northford: 22 Foote Hill Rd.) 41°23'23"N, 72°48'15"W, 26.vii.1990, M.K. Oliver, gilled tan mushroom, stump, (MKOC, 1); same locality, 1.vii.1989, M.K. Oliver, (MKOC, 1); same locality, 10.vi.1993, M.K. Oliver, porch light, (MKOC, 1); North Haven (480 Skiff St.) 41°22'08"N, 72°54'02"W, 26.vii.1982, M.K. Oliver, Gro-Lux, (MKOC, 1); North Haven (9 Kent Dr.) 41°22'05"N, 72°54'04"W, 27.ix.1985, M.K. Oliver, in woodpile, (MKOC, 1); New Haven, 24.vii.1922, M.P. Zappe, (MCZ, 1);

*Tolland Co*.: Mansfield Center, 22.vii.1997, ex fungus, (UCMS, 1); Storrs, 28.vii.1986, J.V. McHugh, ex flight intercept trap, (CUIC, 1).

**DISTRICT OF COLUMBIA**

Ulke Collection, (CMNH, 3); no other data, (MCZ, 1); Palisades Park along Potomac River 3855N 7706W, 15.xii.1996, W.E. Steiner & J.M. Swearingen, (USNM, 3).

**DELAWARE**

*New Castle Co*.: Newark, 21.iii.1969, (FSCA, 1).

*Sussex Co*.: Ellendale forest, 1.ii.1970, M. Druckenbrod, under bark, (USNM, 1).

**FLORIDA**

Ulke Collection, (CMNH, 1); no other data, (CUIC, 1); no other data, (INHS, 1); no other data, (MCZ, 1); no other data, (MCZ, 1).

*Alachua Co*.: 4 mi. N. of LaCrosse, J. & E. Ranch, 13–17.ix.1989, L.R. Davis, Jr., (FSCA, 1); Gainesville, 23.iii.1953, H.F. Howden, shelf fungi, (CMNC, 1); same locality, 24.i.1979, E.C. Becker, under bark, (CNC, 13); same locality, 3.iii.1923, A. Walter, (UMMZ, 1); Gainesville, San Felasco Hammock, 26.v.1977, G.B. Fairchild & H.V. Weems, Jr., insect flight trap, (FSCA, 1).

*Baker Co*.: Olustee, 11.vii.1966, E.F. Merkel, black light, (CNC, 1); Osceola Nat. For., 2 mi. S. Olustee, 10–15.iv.1980, G.B. Fairchild, insect flight trap, (FSCA, 1); Glen St. Mary, 30°18'00"N, 82°00'55"W, 5.iv.2007, rural yard / mixed woods malaise trap, E. Zoll, S. Fullerton, (UCFC, 1); same locality, 13.ix.2007, (UCFC, 1).

*Brevard Co*.: Titusville, SR 405, 30.v-13.vi.2001, P.J. Russell, Z. Prusak, S.M. Fullerton, Enchanted Forest Sanct. White Trail, xeric oak hammock malaise trap, (UCFC, 3); same locality, 21.iii-4.iv.2001, (UCFC, 1); 4–18.iv.2001, (UCFC, 1); 27.vi-11.vii.2001, (UCFC, 1); 11–25.vii.2001, (UCFC, 1); same locality, Z. Prusak, P.J. Russell, S.M. Fullerton, 15–31.viii.2000, (UCFC, 1); 31.viii-14.ix.2000, (UCFC, 1); 14–28.ix.2000, (UCFC, 1); Malabar, Malabar Rd., 30.iii-7.iv.2001, P.J. Russell, Z. Prusak, S.M. Fullerton, Malabar Scrub Sanct. Fire unit 16, xeric oak scrub, malaise trap, (UCFC, 1); same locality, 29.iv-10.v.2001, (UCFC, 1).

*Calhoun Co*.: Chipola Park, Dead Lake, 20.vi.1965, *Poria ambigua*, (MCZ, 2).

*Dade Co*.: Biscayne Bay, (CUIC, 2).

*Duval Co*.: Jacksonville, 8.xii.1962, G.T. Riegel, (EIUC, 6); Jachson., A.T. Slosson, (AMNH, 2).

*Hernando Co*.: Withlacoochee St. For., Richloam Tract, Goat Rd., 12.viii.1992, M.C. Thomas, (FSCA, 1).

*Highlands Co*.: 7 mi. SE of Lake Placid, 19.vi.1955, H.S. Dybas, (FMNH, 1); Archbold Biol. Sta., 3.v.1978, L.L. Lampert, Jr., uvl, (FSCA, 1); Highl. Hammock S.P., 23–25.vi.1981, S. Peck, *Griffolia* fungus; teneral, (CNC, 1); Highlands Hammock, 12.iv.1971, A. Newton, *Polyporus hypnoides*, (MCZ, 1); Highlands Hammock St. Pk., 12.iv.1971, A. Newton, ex *Polyporus sulphureus*, (MCZ, 2); same locality, 16.iv.1990, R. Turnbow, (RTC, 4).

*Hillsborough Co*.: Mac Dill Field, Tampa, 15.iv.1943, B. Malkin, (FMNH, 1); Tampa, Mac Dill Fd., 10–15.iii.1943, (CUIC, 1).

*Jackson Co*.: Florida Caverns S.P., 12.viii.1985, J. Negron, (LSU, 1); Torreya St. Pk., 25.iii.1986, Q.D. Wheeler, flood plain, ex fungi & under bark, Lot # 86012, (CUIC, 1).

*Liberty Co*.: Torreya St. Pk., 19–23.iv.1984, E.G. & M.A. Riley, (EGRC, 1); same locality, 25.vi.1989, R. Turnbow, (RTC, 1); same locality, 23.viii.1951, I.J. Cantrall, (UMMZ, 1); Apal. Blffs & Rav. Pres., N 30 29 42.66, W 84 58 42.46, 9.vii.2008, mxd hrdwd/closed canopy seepage slope/ ravine, 183 ft., malaise trap, (UCFC, 1).

*Monroe Co*.: Key West, (CUIC, 5).

*Okeechobee Co*.: Bell Glade, Lake Okeechobee, 9.xii.1925, M.D. Leonard, (CUIC, 1).

*Orange Co*.: Rk Spr Rn St Res, 10.ix.1994, J.C. Longhurst & S.M. Fullerton, spine/oak scrub malaise trap, (UCFC, 1); UCF Orlando , 30.viii.1993, S.M. Fullerton, Cypress dome malaise trap, (UCFC, 1); same locality, 17.x.1993, S.M. Fullerton, Cypress dome malaise trap, (UCFC, 1); same locality, 10.x.1992, S.M. Fullerton, LLP sand pine turkeyoak malaise trap, (UCFC, 1); same locality, 12.x.1995, S.M. Fullerton, LLP sand pine turkeyoak malaise trap, (UCFC, 1); same locality, 23.vi.1995, S.M. Fullerton, LLP sand pine turkeyoak malaise trap, (UCFC, 1); same locality, 25.viii.1992, S.M. Fullerton, LLP sand pine turkeyoak malaise trap, (UCFC, 1); same locality, 17.viii.1995, S.M. Fullerton, LLP sand pine turkeyoak malaise trap, (UCFC, 1); same locality, 19.vi.1993, S.M. Fullerton, LLP sand pine turkeyoak malaise trap, (UCFC, 1); same locality, 14.v.1993, S.M. Fullerton, LLP sand pine turkeyoak malaise trap, (UCFC, 1); same locality, 30.viii.1992, S.M. Fullerton, LLP sand pine turkeyoak malaise trap, (UCFC, 1); same locality, 26.v.1994, S.M. Fullerton, LLP sand pine turkeyoak malaise trap, (UCFC, 1); same locality, 11.vii.1005, S.M. Fullerton, LLP sand pine turkeyoak malaise trap, (UCFC, 1); same locality, 17.ix.1995, S.M. Fullerton, LLP sand pine turkeyoak malaise trap, (UCFC, 1); same locality, 27.ix.1992, S.M. Fullerton, LLP sand pine turkeyoak malaise trap, (UCFC, 1); same locality, 25.iv.1997, S.M. Fullerton, long leaf pine saw palmetto malaise trap, (UCFC, 1); same locality, 21.vi.1999, P. Russell & S. Fullerton, Cypress Forest malaise trap, (UCFC, 1); same locality, 30.viii.1992, S.M. Fullerton, LLP sand pine turkeyoak malaise trap, (UCFC, 1); same locality, 17.ix.1997, S.M. Fullerton, LLP sand pine turkeyoak malaise trap, (UCFC, 1); same locality, 5.iv.1999, P. Russell & S. Fullerton, Rond tine comm. Dahoon holly malaise trap, (UCFC, 1); same locality, 2.x.1997, S.M. Fullerton, long leaf pine saw palmetto malaise trap, (UCFC, 1); same locality, 17.ix.1997, S.M. Fullerton, LLP sand pine turkeyoak malaise trap, (UCFC, 1); same locality, 14.vi.1999, P. Russell & S. Fullerton, Cypress Forest malaise trap, (UCFC, 2); UCF Orlando Mackay tract, 12.iv.1999, P. Russell & S. Fullerton, Sawgrass marsh red maple malaise trap, (UCFC, 1); Orlando, iii, L.B. Woodruff, (AMNH, 1); Winter Park, (MCZ, 2); Orlando, 21.x.2002, S. Fullerton, T.C. Sims, Lob Bay/ wax myrtle seepage area, NE corner campus, malaise trap, (UCFC, 1); Wekiwa Springs State Park, S22 T20S R28E, burn zone 43/31, longleaf pine – turkey oak, malaise trap, P.J. Russell, S. M. Fullerton, 13.iv.2001 (UCFC, 2); 28.iv.2001, (UCFC, 1); 7.vii.2001, (UCFC, 1); 21.vii.2001, (UCFC, 2); 4.viii.2001, (UCFC, 2); 16.ix.2001, (UCFC, 1).

*Osceola Co*.: Walt Disney World, 5–12.v.1998, Z. Prusak & S. Fullerton, World Drive US 192 sand pine/rosemary malaise trap, (UCFC, 2); same locality, 30.vi.1998, Z. Prusak & S. Fullerton, Xeric oak/flatwoods malaise trap, (UCFC, 1); Kissimmee St. 2 TNC-Dis. Wild. Pres. 28.07N 81.26W, 27.viii-5.ix.2001, el. 67 ft., TNC staff, LL pine/palmetto/grass malaise trap, (UCFC, 1).

*Pinellas Co*.: Honeymoon Is., 15–21.vi.1999, M.A. Goodrich, Malaise trap, (EIUC, 1); same locality, 20–27.I.1999, M.A. Goodrich, Malaise trap, (EIUC, 1); same locality, 9–15.viii.2000, M.A. Goodrich, Malaise trap, (EIUC, 6); same locality, 19–26.vii.2000, M.A. Goodrich, Malaise trap, (EIUC, 5); same locality, 12–19.vii.2000, M.A. Goodrich, Malaise trap, (EIUC, 2); same locality, 2–9.viii.2000, M.A. Goodrich, Malaise trap, (EIUC, 5); same locality, 26.vii-2.viii.2000, M.A. Goodrich, Malaise trap, (EIUC, 5); Gulfport, 9.iii.1925, F.M. Gaige, (UMMZ, 1).

*Polk Co*.: Lakeland, 7.v.1912, (AMNH, 3); Lakeland, 7.v.1912, (AMNH, 1).

*Seminole Co*.: Econ. Wild. Area, 1.x.2000, T. Smith, P. Russell, S. Fullerton, scrub oak/saw palmetto (burned) malaise trap, (UCFC, 1); same locality, 2.ix.2000, T. Smith, P. Russell, S. Fullerton, scrub oak/saw palmetto (burned) malaise trap, (UCFC, 1); Lower Wekiva River St. Pres., burn zone LW-10, S39 T19S R29E, 5.viii.2001, LLP-turkey oak green pan traps 3 ft. above ground, P. Russell, S. Fullerton, (UCFC, 1); Lower Wekiva River St. Preserve, Burn Zone LW-5, S 39 T19S R29E, 5.iii.2001, longleaf pine – turkey oak malaise trap, P. J. Russell, S.M. Fullerton, (UCFC, 1); 4.viii.2001, (UCFC, 1).

*St. Lucie Co*.: Port St. Lucie, 9.ii.1988, D.A. Pollock & L.A. Reichert, under bark of dead pine, (DAPC, 1).

*Volusia Co*.: S. Daytona, 21.iv.1961, J.F. Brimley, (CNC, 1); Enterprise, ii.1917, (CUIC, 1); same locality, 30.x, (CUIC, 2); same locality, (CUIC, 1); same locality, 18.v, (MCZ, 2).

*Wakulla Co*.: Apalachicola N. Forest, S.W. Tallahassee, 9.v.1985, J. Doyen & W. Tschinkel, (UCBC, 4).

**GEORGIA**

*Baker Co*.: Emory Un. Field Sta, Newton, 24.vi.1952, (CMNC, 1); same locality, 25.vii.1952, (CMNC, 1); Newton, 30.iii.1956, H. Howden, (CMNC, 1); Newton, Emory Univ. Field Sta, 7–14.v.1952, (CNC, 1).

*Bartow Co*.: Cartersville, 26.iii.1939, P.W. Fattig, (UGAC, 1).

*Calhoun Co*. Arlington, 11.vi.1947, P. W. Fattig, (UGAC, 2).

*Camden Co*.: Little Cumberland I., 1.x.1982, W.E. Steiner, (USNM, 1); Little Cumberland I. 3058N 8125W, 2.vii.1996, W.E. Steiner, (USNM, 2).

*Clarke Co*.: 24.iv.1984, R. Morris, (UGAC, 1); 5/3/85, R. Morris, (UGAC, 1); 1.1 mi SW Winterville, 15–19.vii.1994, C.L. Smith, ex malaise trap, (UGAC, 2); same locality, 29.v-6.vi.1994, C.L. Smith, ex malaise trap, (UGAC, 1); 5 mi. W. Athens, 30.viii.1977, C. L. Smith, removed from light fixture, (UGAC, 1); same locality, 1–7.vi.1976, C. L. Smith, at light, (UGAC, 1); Athens, 14.viii.1999, W.B. Sikora, black light, (UGAC, 1); same locality, 5–6.viii.1997, W.B. Sikora, ex: malaise trap, (UGAC, 1); same locality, 8–14.v.1976, J.F. MacDonald & R.W. Matthews, Malaise trap, (UGAC, 1); same locality, 1–7.vi.1976, J.F. MacDonald & R.W. Matthews, Malaise trap, (UGAC, 1); same locality, 14.v.1980, S.N. Brown, bl. Light, (UGAC, 1); same locality, 26–28.iv.1981, S.N. Brown, blacklight, (UGAC, 1); same locality, VII.1976, (UGAC, 1); same locality, 24–31.iii.1977, R.W. Matthews, ex malaise trap, (UGAC, 1); same locality, 24.vi.1937, P. W. Fattig, (UGAC, 2); same locality, 2.ix.1937, P. W. Fattig, (UGAC, 1); same locality, 6.iv.1942, P. W. Fattig, (UGAC, 1); same locality, 1.v.1936, P. W. Fattig, (UGAC, 1); Atlanta, 7.iv.1946, P. W. Fattig, (UGAC, 1).

*Dekalb Co*.: 3.v.1972, J. Wappes, (TAMU, 1); Stone Mt., 28.iv.1927, P. W. Fattig, (UGAC, 1).

*Dougherty Co*.: Albany, 26.ii.1969, L.R. Davis, Jr., (FSCA, 7).

*Floyd Co*.: Rome, 23.xi.1977, Wharton, (TAMU, 1).

*Fulton Co*.: Atlanta, 13.vi.1938, P.W. Fattig, (FMNH, 1).

*Greene Co*.: Oconee Natl. Forest, 8-Jan-77, C.L. Smith, hibernating under bark, (UGAC, 1).

*Gwinnett Co*.: 4 mi SSE Dacula, 17.vii.1998, M. Seres, (UGAC, 1).

*Lowndes Co*.: 17.ix.1961, E.I. Hazard, (FSCA, 1); 20.vii.1962, E.I. Hazard, (FSCA, 6).

*McIntosh Co*.: Sapelo Island, 28.iv-9.v.1987, BRC Hym. team, oak forest, MT, (CMNC, 2).

*Meriwether Co*.: Warm Springs, 20.iii.1980, J.H. Chandler, Jr., (UGAC, 1).

*Muscogee Co*.: Ft. Benning, 11.v.1942, J.H. Kremer, (FMNH, 1).

*Paulding Co*.: Dallas, 19.vii.1942, P. W. Fattig, (UGAC, 1).

*Rabun Co*.: vii, (CUIC, 1).

*Thomas Co*.: Thomasville, 21.iii.1939, P.W. Fattig, (UGAC, 1).

**IDAHO**

*Jerome Co*.: 7 mi S. Jerome, 4.v.1976, A.A. Allen, (FMNH, 1).

*Twin Falls Co*.: Buhl, 1.i.1977, A.D. Allen, (AMNH, 3; TAMU, 1).

**ILLINOIS**

Stromberg Colln., (INHS, 3); no other data, (MCZ, 6).

*Adams Co*., Quincy, (MCZ, 3).

*Alexander Co*.: Cairo, 28.iv.1946, J.C. Bradley, (CUIC, 1).

*Champaign Co*.: 1.iv.1960, J.M. Campbell, under bark, (CNC, 1); 9.xii.1972, L. Davis, (FSCA, 1); 25.iv.1960, J.M. Campbell, (AMNH, 2); Brown Field Woods, 22.iv.1965, J.D. Pinto, (UCRC, 1); Brownfield Woods, 3 mi. NE Urbana, 18.iv.1977, C.T. Maier, (INHS, 1); Champaign, 9.x.1960, (UCBC, 3); Trelease Woods, 6.vi.1973, L.R. Davis, Jr., (FSCA, 1); Urbana, 18.v.1933, (FMNH, 1); Mayview, 3.xii.1944, J.L.C. Rapp, (INHS, 1); Urbana, 17.iv.1907, (INHS, 1); same locality, 14.iv.1907, (INHS, 2); same locality, 6.i.1907, (INHS, 2); same locality, 4.ix.1911, (INHS, 1); same locality, 14.x.1931, H.E. McClure, under bands of tar paper on apple trees, (USNM, 1); same locality, 9.x.1931, H.E. McClure, under bands of tar paper on apple trees, (USNM, 1).

*Clark Co*.: Rocky Branch, 14–21.v.1995, M.A. Goodrich, pitfall trap, (DAPC, 1) same locality, 10.v.1989, M.A. Goodrich and P. Skelley, (DAPC, 1); same locality, 7–12.v.1991, M.A. Goodrich, malaise trap, (EIUC, 3); same locality, 27.v-2.vi.1991, M.A. Goodrich, malaise trap, (EIUC, 1); same locality, 16.ix.1991, M.A. Goodrich & R.S. Hanley, *Armillariella mellea*, (EIUC, 1); same locality, 29.v-2.vi.1991, M.A. Goodrich, malaise trap, (EIUC, 1); same locality, 24.vi.1991, M.A. Goodrich, (EIUC, 1); same locality, 10.vii.1991, M.A. Goodrich, (EIUC, 3); Marshall, 14.ii.1932, Frison & Mohr, (INHS, 2).

*Clay Co*.: 3 mi SW Iols, 25.viii.1991, S. Fletcher, (EIUC, 1).

*Clinton Co*.: 4.vi.1975, K. Stephan, (FSCA, 2).

*Coles Co*.: 22.ix.1977, A.A. Brooks, (EIUC, 1); 12.v.1970, Decker, (EIUC, 1); 5.viii.1972, Sauer, (EIUC, 1); 2.x.1960, G.F. Reid, (EIUC, 1); 29.ix.1965, M.A. Goodrich, (EIUC, 1); 10.viii.1970, A. Decker, u-v light trap, (EIUC, 1); Charleston, 3.vii, (EIUC, 1); Embarras Bluff, 28.iv.1970, Decker, (EIUC, 1); Fox Ridge State Park, 9.vi.1973, L.R. Davis, Jr., (FSCA, 1).

*Cook Co*.: E.B. Chope, (FMNH, 7); v, Blackwelder, (WIRC, 1); 11.iv.1950, G.E. Haas, under bark, (INHS, 1); Chicago, v.1920, W.J. Gerhard, (FMNH, 1); same locality, 28.vii.1950, at light, (FMNH, 1); Crete, vii.1969, light trap, (FMNH, 1); Glen View, 1.x.1930, F. Psota, (FMNH, 1); same locality, 2.vii.1905, A.B. Wolcott, (FMNH, 4); Markham, vii.1969, light trap, (FMNH, 1); Palos Park, 20.vi.1937, H. Dybas, (FMNH, 1); same locality, 19.vi.1932, (FMNH, 1); same locality, 21.vi.1908, E. Liljeblad, (UMMZ, 1); same locality, 7.x.1905, E. Liljeblad, (UMMZ, 1); S. Chicago, 1.xi.1903, W.J. Gerhard, (FMNH, 1); South Holland, vii.1969, light trap, (FMNH, 1); same locality, vii.1969, light trap, (FMNH, 1); Willow Springs, 24.ix.1942, H.S. Dybas, (FMNH, 1); same locality, 13.viii.1905, A.B. Wolcott, (FMNH, 4); same locality, 31.vii.1904, A.B. Wolcott, (FMNH, 7); same locality, 17.vii.1907, E.B. Chope, (FMNH, 7); Winnetka, Wm. Erickson For. Pres., 7.vi.1971, V. Reaves & M. Hollander, under bark of log, (FMNH, 1); Bowmanville, 4.ix.1905, (FMNH, 2; UMMZ, 1); Edgebrook, 9.iv.1905, (FMNH, 1); So. Deering, 18.iv.1909, W.J. Gerhard, (FMNH, 1); Riverside, 8.vii.1913, (UMMZ, 3).

*DuPage Co*.: Glen Ellyn, 9.ix.1897, E.B. Chope, (FMNH, 3).

*Edgar Co*.: 4 mi SSE Kansas, 20–21.v.1999, M.A. Goodrich, UV light trap, (EIUC, 1); same locality, 14.iv.1999, M.A. Goodrich, (EIUC, 2); same locality, 3–10.v.2000, M.A. Goodrich, Malaise trap, (EIUC, 2); same locality, 1–8.ix.2000, M.A. Goodrich, Malaise trap, (EIUC, 1).

*Effingham Co*.: 6.vii.1964, Nardick, (EIUC, 1).

*Jackson Co*.: 7 mi. W. Carbondale, 22–29.v.1993, M.A. Goodrich & D.L. Wood, Malaise trap, (DAPC, 1); nr. Carterville, 11.ix.1962, G.T. Riegel, in sawdust, (EIUC, 1).

*Jasper Co*.: 3.5 mi. W. or Rose Hill, west bank of Embarras River, 5.v.1973, L.R. Davis, Jr., (FSCA, 1).

*Jefferson Co*.: 2 mi. SE Ina, 27.viii-3.ix.1994, M.A. Goodrich & T.K. Prosise, malaise trap, (EIUC, 1); 2 mi. SE Ina, 20–27.viii.1994, M.A. Goodrich & T.K. Prosise, malaise trap, (EIUC, 1).

*Jersey Co*.: Pere Marq. Wildlife Area, 3.v.1938, Ross & Burks, (INHS, 1).

*Johnson Co*.: Ferne Clyffe St. Pk., nr Goreville, 5–6.vi.1971, B. Wildie, (MSUC, 1).

*Kendall Co*.: Silver Springs St. Pk, 26.iv.1981, L. Watrous, ex debris ar. logs and under bark, (FMNH, 1).

*Lake Co*.: Libertyville, 11.x.1921, (FMNH, 1); Volo, 15.viii.1926, (FMNH, 1).

*LaSalle Co*.: Ottawa, 5.v.1946, F.G. Werner, (UAIC, 2).

*Macon Co*.: 20.vi.1984, P. Skelley, at lights, (EIUC, 1); Lincoln Trail, Homestead State Park, 6.v.1973, L.R. Davis, Jr., (FSCA, 2).

*Marion Co*.: Centralia, 2.viii.1922, W.B. Cartwright, (USNM, 1); Havana, 11.viii.1907, (INHS, 2); same locality, 16.viii.1907, (INHS, 1).

*Mason Co*.: 19.i.1973, (FSCA, 1).

*McHenry Co*.: Algonquin, Nason, (INHS, 2).

*McLean Co*.: Bloomington, 12.v.1883, (INHS, 1); Normal, 1.xi.1883, (INHS, 1).

*Peoria Co*.: Peoria, 8.vii.1943, F.F. Hasbrouck, at light on Main st., (INHS, 1).

*Platt Co*.: Allerton Park, 9.iii.1973, L. Davis, (FSCA, 1); White Heath, 21.vi.1917, (INHS, 1); same locality, 9.v.1926, K.F. Auden, (INHS, 2); same locality, 21.vi.1917, (INHS, 2); same locality, 24.iv.1926, K.F. Auden, (INHS, 1).

*Pope Co*.: 8.x.1965, A. Holt, (EIUC, 1).

*Putnam Co*.: 24.vi.1915, M.C. Glenn, (INHS, 1); 4.v.1932, M.C. Glenn, (INHS, 1).

*Sangamon Co*.: Springfield, 23.iii.1926, (FMNH, 1).

*Stephenson Co*.: Freeport, 21.vii.1949, W.F. Chamberlain, at light, (TAMU, 1).

*Vermilion Co*.: Forest Glen F.P., 5 mi. SE Westville, 19–20.iv.1977, D.W. Webb, (INHS, 2); Kickapoo St. Pk., 24.xi.1972, L. Davis, (FSCA, 1).

*Wabash Co*.: Beall Woods N.P, 11–18.vi.1997, M.A. Goodrich, Malaise trap, (EIUC, 1); Beall Woods N.P., 15.viii.1996, M.A. Goodrich, ex *Pleurotus ostreatus*, (EIUC, 1); same locality, 13–14.vi.1996, M.A. Goodrich, u-v light trap, (EIUC, 1).

*Washington Co*.: Dubois, 22.vi.1905, (INHS, 1).

*White Co*.: Maunie, 9.viii.1937, H. Dybas, (FMNH, 2).

*Whiteside Co*.: Mineral Springs, 30.xi.1911, A.B. Wolcott, (FMNH, 4); Hopkins, 1.ix.1939, O. Park, (FMNH, 15).

*Will Co*.: New Lenox, 17.iv.1937, D.C. Lowrie, (FMNH, 1).

*Winnebago Co*.: nr. Rockford (Cp. Grant), 26.vi.1944, H.S. Dybas, (FMNH, 1)

*Richland & Lawrence Co*.: Wabash Valley, v.1879, (MCZ, 1).

*Pike & Calhoun Co*.: 9–14.viii.1907, (INHS, 1).

*Counties unknown*: Romer Park, 11.vii.1927, T.H.F. & R.D.G., (INHS, 1); St. Joseph, 12.xi.1944, J.L.C. Rapp, (INHS, 1).

**INDIANA**

no other data, (FMNH, 4).

*Bartholomew Co*.: Columbus, 29.iv.1945, N.M. Downie, (LACM, 1); same locality, 29.iv.1945, N.M. Downie, (UCRC, 2).

*Crawford Co*.: 3 mi. N. English, 21.viii.1985, R.S. Miller, (MTEC, 1).

*Howard Co*.: NW Howard Co., 14.viii.1985, D.A. Rider, (LSU, 1); NW Howard Co., 16.viii.1985, D.A. Rider, (LSU, 1).

*Lake Co*.: Lake Station, 10–11.v.1909, E. Liljeblad, (UMMZ, 2); E. Gary, 30.vi.1911, (UMMZ, 1).

*Laporte Co*.: NE of Springville, Smith’s Woods, Smith Station, 10.viii.1940, B. Patterson, (FMNH, 1); Smith Sta., 29.viii.1947, H. Dybas, (FMNH, 1).

*Monroe Co*.: Bloomington, 3–4.ix.1983, F.N. Young, BLT, (FSCA, 1); same locality, 9–10.vii.1986, F.N. Young, BLT, (FSCA, 1); same locality, 28.vii.1987, F.N. Young, BLT, (FSCA, 2).

*Parke Co*.: 13.iv.1960, N.M. Downie, (FMNH, 1); Turkey Run Sp, 17.vi.2001, L.E. Crofutt, (EIUC, 1).

*Perry Co*.: T4S R1W s. 36, 22.iv.1978, J. Liebherr, under bark, (UCBC, 4).

*Porter Co*.: Dune Acres, 19.iv.1942, D.C. Lowrie, (FMNH, 1); Ogden Dunes, 23.ix.1928, (FMNH, 2).

*Posey Co*.: Harmonie St. Park 4 mi S New Harmony, 28.vi.1983, Grissell & Menke, (USNM, 1); Hwy 69, 1.5 mi. S. Hovey, 350’, 20.v.1973, D.H. Kavanaugh & H. Goulet, (CNC, 1); Hovey Lake, 16.v.1965, C.E. White, blacklight trap, (FSCA, 1); same locality, 10.iv.1965, C.E. White, (FSCA, 1).

*Tippecanoe Co*.: 3.vi.1963, N.M. Downie, (DEUN, 3); same locality, 30.vi.1962, N.M. Downie, (FMNH, 1); same locality, 29.ix.1964, N.M. Downie, (FMNH, 1); same locality, 10.x.1964, N.M. Downie, (FMNH, 1); same locality, 3.ix.1977, N.M. Downie, (FMNH, 1); same locality, 4.vii.1952, N.M. Downie, (FMNH, 3); same locality, 18.viii.1977, N.M. Downie, (TAMU, 1); same locality, 8.ix.1977, N.M. Downie, (TAMU, 1); same locality, 1.viii.1957, N.M. Downie, (AMNH, 3); McCormick’s Woods, 10.vii.1987, N.M. Downie, (PKLC, 6); West Lafayette, 4.vii.1977, H.O. Lund, (UGAC, 1); West Lafayette, McCormick Woods, 13.v.1980, C.W. O’Brien, (DAPC, 2); Lafayette, 22.vii.1987, uv light, (DENH, 1).

*Vigo Co*.: Terre Haute, 20.x.1907, G.A. Akerlind, (UMMZ, 1).

*Wayne Co*.: Richmond, 12.ix.1950, Ross et al., (INHS, 1).

*County unknown*: S. McAlester, 11.vi, Wickham, (MCZ, 1).

**IOWA**

no other data, (CUIC, 3); no other data, (INHS, 1).

*Benton Co*.: Vinton, 22.vii.1928, N.K. Bigelow, (UMMZ, 9).

*Henry Co*.: Mt. Pleasant, 16.iii.1934, Millspaugh, (USNM, 1); same locality, 1.x.1933, Throckmorta, (USNM, 1).

*Keokuk Co*.: Atwood, B. Shimek, (MTEC, 1).

*Johnson Co*.: Iowa City, Wickham, (CUIC, 1); same locality, 27.iii.1915, Stoner, (USNM, 4); same locality, (MTEC, 1).

*Linn Co*.: 3 mi. N. Viola, 260 m, 30.vii-5.viii.1975, E.L. Sleeper, blacklight, decid. woodland, (CAS, 1).

*Osceola Co*.: 4.5 mi. SSE Ocheyedan, 9.viii.1992, P.K. & E.B. Lago, (PKLC, 1).

*Story Co*.: Ames, 6.iv.1932, J.F. Kagy, (CAS, 1); Ames, 17.iv.1932, J.F. Kagy, (CAS, 1); same locality, 7.iv.1941, L. Potter, (NDSU, 1); same locality, 5.iv.1955, T. Schulz, (NDSU, 1); same locality, 8.v.1939, C.P. Haight, (MTEC, 2); same locality 15.iv.1929, CPL, (MTEC, 2); same locality, 12.iv.1951, A.H. Barnum, (BYUC, 6).

**KANSAS**

Ulke Collection, (CMNH, 1); no other data, (FMNH, 2); no other data, (INHS, 5); no other data, (INHS, 1); Chas Palm, (AMNH, 1).

*Atchison Co*.: Atchison, 24.iv.1957, D.J.B. McReynolds, (FSCA, 1).

*Douglas Co*.: ix.1949, Ronald H. Pine, (USNM, 1); 900’, F.H. Snow, (CUIC, 2); Lawrence, 1929, L.W. Brown, (CNC, 40); same locality, 20.iv.1938, W.J. Brown, (CNC, 1); same locality, iv.1928, W.J. Brown, (CNC, 1); Lawrence, KU campus, 30.vii.1996, Z.H. Falin, on shelf fungi, (ZHFC, 1); Lawrence: West Campus, 18.viii.1995, Steve Lingafelter, Sugar-bait trap, (USNM, 1); Shawnee Mission Park, 21.iv.1968, Robert Heitzman, (AMNH, 1); Lawrence, ix, Blackwelder, (WIRC, 2); Lone Star, 27.iv.1953, W.J. Brown, (CNC, 2).

*Labette Co*.: Oswego , 25.xi.1964, Gary F. Hevel, (USNM, 1); same locality, 8.iv.1968, Gary F. Hevel, (USNM, 1).

*Lyon Co*.: Emporia, 23.v.1959, H. Willis, (FSCA, 1).

*Miami Co*.: State Park, 1.xi.1952, P.J. Spangler, (UMRM, 4); same locality, 1.xi.1952, P.J. Spangler, (USNM, 14).

*Riley Co*.: 6.vi.1970, R.L. Bertwell, black light trap, (UAIC, 3); Manhattan, 17.iii.1935, (MSUC, 3).

*Sedgwick Co*.: iii, (MCZ, 1); Mt. Hope, 19.iv.1904, (INHS, 1).

*Shawnee Co*.: Auburn, 24.x.1933, C. Seevers, (FMNH, 1); same locality, 1.xi.1933, H. Seevers, (FMNH, 1).

*Trego Co*.: Cedar Bluff S.P., 12.vii.1989, Y. Bousquet, (CNC, 1).

*Wilson Co*.: Neodesha, 16.v.1959, H. Willis, (FSCA, 1).

*Wyandotte Co*.: Argentine,13.xi.1905, (UMRM, 1); same locality, 14.v.1904, (UMRM, 2); same locality, 6.11, (MCZ, 1); same locality, 19.v.1907, (MCZ, 2).

**KENTUCKY**

no other data, (CUIC, 3); no other data, (MCZ, 1); no other data, (AMNH, 1).

*Butler Co*.: 11.v.1970, J.M. Campbell, (CNC, 9).

*Christian Co*.: 5 mi W. Hopkinsville, 29.v.1986, D.A. Pollock, at black light, (DAPC, 1).

*Green Co*.: 8.viii.1947, B. Summnerville, (USNM, 1).

*Henderson Co*.: Henderson, 12.ii.1927, M.Y. Marshall, (CUIC, 1; UAIC, 3); same locality, 2.iv.1922, M.Y. Marshall, (UMMZ, 1); same locality, 12.ii.1921, M.Y. Marshall, (UMMZ, 1); same locality, 14.viii.1921, M.Y. Marshall, (UMMZ, 1).

*Taylor Co*.: 20.vii.1949, T.J. Spilman, (USNM, 2).

**LOUISIANA**

no other data, (MCZ, 1); no other data, (UMRM, 2).

*Bossier Par*.: Bodcau Sdlf. Mgt. Ar., 32°42’N, 93°30’W, 22.v.1996, E.G. Riley, uv light; 350, (TAMU, 3); Bossier City, 20.vii.1960, H. Howden, light, (CNC, 1).

*Caddo Par*.: Shreveport, 16.iv.1949, J.H. Robinson, (FMNH, 1).

*Claiborne Par*.: Lake Claiborne St. Pk., 6.vii.1983, E.G. Riley, (EGRC, 1); same locality, 6.v.1983, E.G. Riley, (EGRC, 1); Corney Lake, 27.vi.1983, Carrs, lot 4, (CARR, 1).

*Concordia Par*.: 5 mi W. Ferriday, 1.v.1976, A. Smetana, (CNC, 2).

*East Baton Rouge Par*.: Baton Rouge, 29.i.1921, O.W. Rosewell, (LSU, 1); same locality, 13.v.1941, S. Pulford, (LSU, 1); same locality, 6–4.1943, J.H. Roberts, (LSU, 1); same locality, 15.v.1978, M.D. Andis, in sweetgum stump, (LSU, 1); same locality, 24.v.1978, M.D. Andis, in sweetgum stump, (LSU, 2); same locality, 7.iv.1978, J.B. Chapin, lighted window screen, (LSU, 1); Baton Rouge Place Du Plantier Apts, 26.v.1985, E.G. Riley, (EGRC, 1); Baton Rouge, 29.vi.1982, E.G. Riley, (EGRC, 1); same locality, 18.vii.1984, E.G. Riley, (EGRC, 1); same locality, 24.vi.1982, E.G. Riley, (EGRC, 1); same locality, 16.vi.1982, E.G. Riley, (EGRC, 1); same locality, 12.v.1983, E.G. Riley, (EGRC, 1); same locality, 21.vi.1982, E.G. Riley, blacklight, (TAMU, 1); same locality, 5.iii.1991, M.S. Strother, under bark, (LSU, 1); LSU campus, 13.v.1985, D.A. Rider, at light, (LSU, 1).

*East Feliciana Par*.: Camp Avondale, 3 mi. E. Clinton, 19.v.1983, E.G. Riley, (EGRC, 1).

*Jefferson Par*.: Harahan, 19.vii.1944, F.G. Werner, (MCZ, 1); same locality, 9.vii.1944, F.G. Werner, (MCZ, 1); same locality, v.1944, F.G. Werner, (UAIC, 2); same locality, ix.1944, F.G. Werner, (UAIC, 2); same locality, viii.1944, H. Dybas, (FMNH, 1).

*Livingstone Par*.: 5 mi. S. Livingstone, 3.xii.1965, *Polyporus adustus*, (MCZ, 2).

*Madison Par*.: Tallulah, P.A. Glick, (CUIC, 1); same locality, 27.vi.1930, (TAMU, 4).

*Natchitoches Par*.: Kisatchie Nat. For., Red Bluff Campgrd., 22.v.1982, E.G. Riley, (EGRC, 1).

*Orleans Par*.: New Orleans, 24.ii.1923, T.H. Hubbell, (UMMZ, 1).

*Webster Par*.: 5.1 mi. N. of Dixie Inn on La Hwy 7, 9.ii.1991, M.S. Strother, (LSU, 1).

*West Feliciana Par*.: Tunica Hills W. of Weyanoke, 8.v.1986, C.B. & J.E. Barr, blacklight, (LSU, 1); same locality, T1S R4W, 18.v.1983, C.B. Barr, collected on fungus, (LSU, 4); same locality, T1S R4W, 18.v.1985, C.B. Barr, collected on fungus, (CBC, 1); Tunica Hills, 2 mi. W. Weyanoke, 26.vi.1987, C.B. Barr, mv & bl, (CBC, 1); Tunica Hills, 5 mi. W. Weyanoke, 15.viii.1986, E.G. Riley, (LSU, 1); Tunica Hills, W. of Weyanoke, 8.v.1986, C.B. & J.E. Barr, bl, (CBC, 1).

*parish unknown*: Camp Claiborne, 4.vii.1941, W.B. St. John, on fungi, (FSCA, 2).

**MARYLAND**

no other data, (MCZ, 1).

*Allegany Co*.: Homewood, 4.iv.1939, Dieke, (USNM, 1); same locality, 17.iii.1939, Dieke, (USNM, 1).

*Anne Arundel Co*.: Edgewater, 23–25.vi.1984, C.L. Staines, Jr., at blacklight, (USNM, 1).

*Baltimore Co*.: Baltimore, (MCZ, 1); same locality, 19.iv.1940, Dieke, (USNM, 1); same locality, 26.v.1940, Dieke, (USNM, 1); Batavia [park] , 18.vi.1913, H.H. Knight, (USNM, 1); Catonsville, 25.ii.1945, George B. Vogt , under bark of dead hickory, (USNM, 1).

*Calvert Co*.: Battle Creek Cypress Swamp, 1.vii.1987, A.&B. Norden, D. Williams, (USNM, 1).

*Caroline Co*.: 7 km S Denton 3856N 7655W, 24.xii.1995, W.E. Steiner & J.M. Swearingen, (USNM, 1).

*Cecil Co*.: Port Deposit , 21.vi.1977, D. Jump, (USNM, 2).

*Charles Co*.: Cederville St. For., 5.iv.1970, M. Druckenbrod, under bark, (USNM, 1).

*Dorchester Co*.: Church Creek, 23.vi.1984, F. Guarneri, at light, (CUIC, 1).

*Frederick Co*.: Catoctin Mts.W. of Thurmont, 3.ii.1991, M.A. Ivie & W. Steiner, (MTEC, 1); Edgewood, 7.ix.1918, H. Dietrich, (CUIC, 2).

*Montgomery Co*.: 4 mi SW of Ashton 390630N 770130W, 3.iii.2001, G.F. Hevel, (USNM, 1); same locality, 5.x.2000, G.F. Hevel, Malaise trap, (USNM, 1); 4mi. SW of Ashton, 17.v.1986, G.F. & J.F. Hevel, Malaise trap, (USNM, 1); C&O Canal near Plummers Island, 21.iv.1982, W.E. Steiner, (USNM, 1); Potomac, 14.xii.1975, W.E. Steiner, (USNM, 1); Rockville, 25.xii.1990, W.E. Steiner & J.M. Swearingen, under bark of dead standing *Quercus*, (USNM, 1); Sycamore Langing, near Poolesville, 6.xi.1979, T.J. Spilman, (USNM, 3); 9.vii.1918, E. Shoemaker, (USNM, 1); 2 mi. E Silver Spr. NW Branch, 30.vi.1950, G.H. Nelson, (WFBM, 1); Bear Island, W of DC, C&O canal, 6.vi.1971, Gary F. Hevel, (USNM, 4); Bethesda, 3.ix.1966, T.J. Spilman, (USNM, 1); Forest Gln, 25.iii.1923, M.T. VanHorn, (USNM, 1); Minnie I, nr Cabin John, 5.vi.1931, H.S. Barber, collected at light, (USNM, 1); Plummers I, 13.vii.1903, Barber & Schwarz, (USNM, 1); same locality, 10.ix, (USNM, 1); same locality, 28.vi.1906, E.A. Schwarz, (USNM, 1); Takoma Park, 12.xi.1950, G.H. Nelson, under bark dead hickory, (WFBM, 1).

*Prince George’s Co*.: Beltsville, 13.iv.1980, Scott Gross, (USNM, 1); Camp Springs, 12.vii.1979, Gary F. Hevel, Malaise trap, (USNM, 1); Watkins Park, 3 km E. Largo, 4.i.1987, W.E. Steiner, M.J. Molineaux, In *Peromyscus* nest debris under bark of dead standing *Liriodendron*, (USNM, 1); Oxon Hill, 23.iv.1972, Gary F. Hevel, (USNM, 1); Cheverly 38°56’N, 76°55’W, 2.ii.2000, W.E. Steiner, (USNM, 2); same locality, 25.vi.1996, W.E. Steiner & J.M. Swearingen, (USNM, 1); same locality and collectors, 30.v.2003, at black l ight at ground level, mixed broken forest and residential area, (USNM, 1); Bladensburg, 10.vi.1972, M. Druckenbrod, flying, (USNM, 1); same locality, 26.vi.1972, M. Druckenbrod, willow branch, (USNM, 1); Takoma Park, 7.xi.1970, M. Druckenbrod, under bark, (USNM, 2); College Park, 8.iv.1948, (CMNC, 1); same locality, 25.iii.1948, (CMNC, 3); same locality, 3.i.1949, H.F. Howden, dead standing pine, (CMNC, 1); same locality, 12.ii.1950, B.K. Dozier, under oak bark, (FSCA, 2); same locality, 2.x.1949, B.K. Dozier, under bark, (FSCA, 2); same locality, 18.v.1942, George B. Vogt , under bark of dead tulip popular, (USNM, 1); same locality, 30.xi.1940, George B. Vogt , under bark of dead *Pinus virginiana*, (USNM, 1); Greenbelt, 30.xii.1950, B.K. Dozier, under pine bark, (FSCA, 2).

*Queen Anne’s Co*.:Carmichael, 22.viii.1973, W.E. Steiner, (USNM, 2).

*St. Mary Co*.: Hays Beach, 4.vii.1949, H.F. Howden, fungus, (CMNC, 3).

*Talbot Co*.: Wittman , 12.iii.1983, W.E. Steiner, under bark of stump of *Prunus serotina*, (USNM, 1); same locality and collector, 14.iii.1982, (USNM, 1).

*Wicomico Co*.: Bivalve, 1.iv.1967, T.J. Spilman, (USNM, 4).

*Worcester Co*.: Pocomoke, 3.vii.1965, Robert Gordon, (USNM, 1).

**MASSACHUSETTS**

no other data, (MCZ, 6); 1869, (MCZ, 1).

*Bristol Co*.: Fall River, 31.v.1909, N.S. Easton, (MCZ, 1); same locality, 26.viii.1911, N.S. Easton, (MCZ, 1); Swansea, viii.1911, N.S. Easton, (MCZ, 1); same locality, 2.viii.1913, N.S. Easton, (MCZ, 1).

*Dukes Co*.: Naushon Islands, Elizabeth Island, 10.vii.1971, C.T. Parson, *Pleurotus*, (MCZ, 1); same locality, 19.viii.1971, C.T. Parsons, *Polyporus sulphureus*, (MCZ, 7).

*Essex Co*.: 2 mi. S. Newbury, 14.vi.1973, (MCZ, 1); Saugus, v, (MCZ, 1); Topsfield, 15.vi.1974, A. Newton, *Polyporus squamosus*, (MCZ, 3).

*Hampden Co*.: Springfield, G. Dimmock, (MCZ, 2).

*Hampshire Co*.: Amherst, 20.xi.1967, R. Duffield, (UGAC, 12); same locality, 18.xi.1967, R. Duffield, (UGAC, 1); same locality, 3/10/68, R. Duffield, (UGAC, 2); same locality, 20.xi.1967, R. Duffield, (AMNH, 10); Northampton, 16.vi.1969, E.J. Kiteley, (CNC, 1); same locality, 5.viii.1973, E.J. Kiteley, bracket fungus dead tree, (CNC, 4); same locality, 21.vi.1971, E.J. Kiteley, (CNC, 1); same locality, 12.viii.1973, E.J. Kiteley, reared from pupa coll. under bark 7.viii.73, (CNC, 1).

*Middlesex Co*.: Framingham, 17.viii.1958, C.A. Frost, elm stump, (MCZ, 1); same locality, 21.vi.1959, C.A. Frost, fungi on elm stub, (MCZ, 1); same locality, 20.v.1959, C.A. Frost, fungi on elm stub, (MCZ, 1); same locality, 1.ix.1961, C.A. Frost, fungus on elm stub, (MCZ, 2); same locality, 24.viii.1952, C.A. Frost, on fungus, elm stub [?], (UAIC, 1); same locality, 24.vii.1939, C.A. Frost, (WFBM, 1); same locality, 21.viii.1958, C.A. Frost, (AMNH, 1); Natick, 12.v.1929, C.A. Frost, under bark, (MCZ, 1); Newton Upper Falls at Boston, viii.1967, J.D. Crump, (USNM, 1); Tyngsboro, v.1871, (MCZ, 2); same locality, 2.iv.1893, (MCZ, 1); same locality, 4.vi.1920, (MCZ, 1); Wayland, 4.xi.1928, C.A. Frost, (MCZ, 1); Bedford, 1.vii.1973, *Panus rudis*, (MCZ, 1).

*Norfolk Co*.: Brookline, v.1874, (MCZ, 1); same locality, 2.vii.1875, (MCZ, 2); same locality, 21.vii.1961, J.I. Richardson, (UGAC, 1).

*Suffolk Co*.: Dorchester, 6.iv.1902, (MCZ, 1); same locality, (MCZ, 1); same locality, 16.vii.1916, Bolster, (MCZ, 1); W. Roxbury, 18.iv.1920, Bolster, (MCZ, 2).

*Worcester Co*.: Northboro, 29.viii.1945, C.A. Frost, (CUIC, 1).

**MICHIGAN**

*Allegan Co*.: Fennville, 17.vi.1959, R.L. Fischer, (MSUC, 1).

*Berrien Co*.: Berrien Springs, 5.v.1979, D. Jump, (USNM, 1); Watervliet, 30.vi.1957, J.B. Tatter, (MSUC, 1).

*Branch Co*.: nr. Tekonsha, 15.vii.1972, T.A. Bowling, (MSUC, 1).

*Charlevoix Co*.: High Isl., 9.viii.1922, S. Moore, (UMMZ, 1); same locality, 24.viii.1923, S. Moore (UMMZ, 4).

*Clare Co*.: 13.v.1950, R.R. Dreisbach, (MSUC, 1).

*Clinton Co*.: Bath, 29.v.1956, R.L. Fischer, (MSUC, 1).

*Eaton Co*.: Eaton Rapids, 6.viii.1975, J. Liebherr, (UCBC, 9); same locality, 23.viii.1975, J. Liebherr, (UCBC, 3); same locality, 13.viii.1975, J. Liebherr, (UCBC, 1); same locality, 3.viii.1975, J. Liebherr, (UCBC, 2); same locality, 18.iv.1976, J.K. Liebherr, (UCBC, 1); same locality, 3.viii.1975, J. Liebherr, (CUIC, 4).

*Ingham Co*.: E. Lansing, 29.v.1937, (MSUC, 1); E. Lansing, 26.iv.1938, (MSUC, 1); same locality, 20.ix.1976, J.K. Liebherr, (UCBC, 1); same locality, 19.ix.1965, E.D. Evans, (AMNH, 1); Aurelius, 15.vii.1938, (MSUC, 1); East Lansing, 3.vi.1938, (UAIC, 1); same locality, 26.v.1940, D.M. Tuttle, (UAIC, 1).

*Isabella Co*.: 11.vii.1954, R.R. Dreisbach, (MSUC, 1).

*Kalamazoo Co*.: Gull Lake Bio. Station, 4.vii.1955, R.L. Fischer, (MSUC, 1); same locality, 25.vi.1966, R.L. Fischer, (MSUC, 1); same locality, 2.viii.1982, R.L. Fischer, (MSUC, 1); same locality, 18.vii.1963, R.L. Fischer, (MSUC, 1); same locality, 3.vii.1957, R.L. Fischer, (MSUC, 1); T1S, R9N, sec. 10, 2.v.1968, N.T. Baker, (MSUC, 1); Galesburg, G. Dimmock, (MCZ, 5).

*Kent Co*.: Grand Rapids, R.H. Wolcott, (DEUN, 3); Grand Rapids, 14.ix.1946, (MSUC, 1).

*Lake Co*.: Branch, 15–19.vii.1974, T.A. Bowling, malaise trap, (MSUC, 1)

*Lenawee Co*.: T8S, R2E, Sec. 32, 9.iv.1966, M.C. Nielsen, (MSUC, 1).

*Livingston Co*.: George Reserve, 26.vi.1932, S. Moore, (UMMZ, 2); same locality, 29.vii.1933, S. Moore, taken at light, (UMMZ, 1).

*Marquette Co*.: Huron Mountain Club, 14.vi.1983, D.C.L. Gosling, (UMMZ, 1).

*Midland Co*.: 16.v.1948, R.R. Dreisbach, (MSUC, 1); 23.vi.1949, R.R. Dreisbach, (UMMZ, 1).

*Monroe Co*.: T6S, R6E, sec. 35, 4.vii.1965, J. Truchan, malaise trap, (MSUC, 1); same locality, 5.ix.1965, J. Truchan, malaise trap, (MSUC, 1); same locality, 18.ix.1965, J. Truchan, malaise trap, (MSUC, 1).

*Oakland Co*.: 18.v.1947, B. Summerville, (MSUC, 1); 19.xi.1922, S. Moore, (UMMZ, 1); Southfield, 4.viii.1931, S. Moore, (UMMZ, 1).

*Ottawa Co*.: Hudsonville, 23.v.1980, R.L. Fischer, blacklight, (MSUC, 1).

*Saginaw Co*.: 6.vi.1948, R.R. Dreisbach, (MSUC, 1).

*Schoolcraft Co*.: Manistique, 20.viii.1923, S. Moore, (UMMZ, 1); Thompson, 10.ix.1923, S. Moore, (UMMZ, 1).

*Shiawassee Co*.: 28.v.1966, E. Evans, T5n, R1E Sec 20–29, (AMNH, 2).

*Washtenaw Co*.: Ann Arbor, 11–14.viii.1988, M. O’Brien, Malaise trap, (UMMZ, 2); same locality, 19–21.vi.1988, M. O’Brien, (UMMZ, 1); Ann Arbor, 3^rd^ Woods, 29.v.1921, T.H. Hubbell, (UMMZ, 2).

*Wayne Co*.: Grosse Ile., 11.vii.1948, G. Steyskal, (LACM, 1); Detroit, 6.ix.1942, G. Steyskal, (LACM, 1); Detroit, G. Dimmock, (MCZ, 5); same locality, 21.v.1041, Geo. Steyskal, (USNM, 1); same locality, 22.iii.1938, G. Steyskal, (UMMZ, 1); 8 Mile Road, 30.iv.1922, S. Moore, (UMMZ, 1).

*Counties unknown*: Ag. College, (NDSU, 2).

**MINNESOTA**

no other data, (CUIC, 1); no other data, (FMNH, 1).

*Crow Wing Co*.: Brainerd, 4.vii.1957, E.J. Kiteley, (CNC, 1).

*Hennepin Co*.: Minneapolis, 5.ix.1947, E.J. Kiteley, (CNC, 1); same locality, 12.vii.1955, E.J. Kiteley, (CNC, 2); same locality, 26.viii.1948, E.J. Kiteley, (CNC, 1); same locality, 5.ix.1947, E.J. Kiteley, (CNC, 1); same locality, 21.vi.1953, E.J. Kiteley, (CNC, 1).

*Washington Co*.: 3 mi. S. Afton, 9.v.1942, R.A. Flock, (UCRC, 2).

**MISSISSIPPPI**

ix.1869, (MCZ, 1).

*Bolivar Co*.: 2 mi. W. Rosedale, 11.iv.1986, P.K. Lago, (UMIC, 1).

*George Co*.: Lucedale, 22.iii.1932, H. Dietrich, (CUIC, 1); same locality, 24.iv.1929, H. Dietrich, (CUIC, 4); same locality, 10.ii.1931, H. Dietrich, (CUIC, 2); same locality, 26.i.1931, H. Dietrich, (CUIC, 3); same locality, 30.xii.1929, H. Dietrich, (CUIC, 1); same locality, 8.viii.1929, H. Dietrich, (CUIC, 1); same locality, 3.vii.1930, H. Dietrich, (CUIC, 1); same locality, 28.xi.1929, H. Dietrich, (CUIC, 1); same locality, 14.vii.1929, H. Dietrich, (CUIC, 2); same locality, 27.ii.1931, H. Dietrich, (CUIC, 1); same locality, 22.i.1931, H. Dietrich, (CUIC, 1).

*Issaquena Co*.: 2 mi. SW Shipland, 20.vi.1992, P.K. Lago, (UMIC, 3).

*Jackson Co*.: 23.viii.1931, H. Dietrich, (CUIC, 1).

*Lafayette Co*.: 11 mi. NW Oxford, 21.iv.1978, P.K. Lago, (UMIC, 3); same locality, 4.iv.1978, S. Hurdle, (UMIC, 1); same locality, 25.vii.1978, S. Hurdle, (UMIC, 1); T7S - R4W - Sec 20, 27.ix.1977, D.F. Stanford, (UMIC, 8); same locality, 20.x.1977, D.F. Stanford, (UMIC, 8); same locality, 27.x.1977, D.F. Stanford, (UMIC, 12); T7S - R4W - Sec. 17, 21.x.1977, A.E. Zuccaro, (UMIC, 5); 11 mi. NW Oxford, 27.x.1977, P.K. Lago, (UMIC, 56); same locality, 25.vii.1978, P.K. Lago, (UMIC, 9); same locality, 19.ii.1978, M.O. Mann, (UMIC, 9); 4 mi. E. Abbeville, 11.iv.1994, P.K. Lago, (UMIC, 2); 6 mi. NW Oxford, 22.i.1977, D.F. Stanford & H. Schuster, (UMIC, 2); 7 mi NW Oxford, 3.iii.1993, B.C. Dillard, fungus on birch, (UMIC, 1); same locality, 18.ii.1993, B.C. Dillard, fungus on bark, (UMIC, 1); same locality, 19.vii.1984, P.K. Lago, (UMIC, 1); same locality, 11.ii.1978, P.K. Lago, (UMIC, 3); same locality, 7.vi.1992, P.K. Lago, (UMIC, 3); Oxford, 7.ix.1973, C.F. Ely, (UMIC, 1); same locality, 28.iv.1983, P.K. Lago, (UMIC, 1); same locality, 2.v.1983, P.K. Lago, (UMIC, 1); same locality, 24.ix.1979, W.M. Wall, (UMIC, 1); same locality, 25.iv.1989, P.K. Lago, (UMIC, 1); same locality, 30.vii.1988, E.B. Lago, (UMIC, 1); same locality, 27.viii.1988, P.K. Lago, (UMIC, 1).

*Montgomery Co*.: 3 mi. NE Winona, 21.vi.1995, J.P. Key, (UMIC, 1).

*Perry Co*.: New Augusta, 12.ii.1931, H. Dietrich, (CUIC, 1); same locality, 27.x.1931, H. Dietrich, (CUIC, 1); same locality, 12.ii.1931, H. Dietrich, (CUIC, 2).

*Prentiss Co*.: Bonneville, 2.ix.1979, J. Goddard, (UMIC, 1).

*Tallahatchie Co*.: 8 mi ESE Charleston, 14.iv.1992, M.S. Caterino, (UMIC, 2).

*Tishomingo Co*.: Tishomingo St. Pk., 22.vi.1981, P.K. Lago, (UMIC, 1).

*Counties unknown*: Saugus, 7.vi.1914, C.A. Frost, (USNM, 1).

**MISSOURI**

no other data, (CUIC, 1).

*Boone Co*.: Columbia, 30.vii.1967, S. Poe, (FSCA, 3); same locality, 23.iv.1976, S.E. Thewke, black light trap, (UMRM, 2); same locality, 11–4, 1974, J. Aldrich, (UGAC, 9); same locality, 10.iv.1941, W.R. Enns, (UMRM, 1); same locality, 9.xii.1967, W.S. Clark, (UMRM, 1); same locality, 9.xii.1939, D. Eschenberg, (UMRM, 4); same locality, 22.iv.1937, H.E. Brown, (UMRM, 1); same locality, 18.iv.1940, W.S. Craig, (UMRM, 1); same locality, 23.viii.1921, A.C. Burrill, at light, (USNM, 1); same locality, 10.vii.1963, F.E. Wood, (USNM, 1).

*Butler Co*.: Poplar Bluff, 14.vi.1957, Enns & Wood, (UMRM, 1).

*Clay Co*.: Coolie Lake, 2.v.1968, R.L. Heitzman, UV light, (FSCA, 1).

*Franklin Co*.: Stanton-Meramec Caverns, 30.v.1985, W.B. Warner, UVBL, (FSCA, 1).

*Greene Co*.: Willard, 4.vii, (UASM, 3).

*Jackson Co*.: vi.1953, R.H. Pine, (TAMU, 1); 3.v.1956, R.H. Pine, (TAMU, 1); Independence, 14.v.1968, J.R. Heitzman, taken at uv light, (SCA, 1); Kansas City , 21.iv.1968, Robert Heitzman, (AMNH, 1).

*Morgan Co*.: Laurie. Bass Point, 21.v.1991, C.W. Young, (CMNH, 1).

*Randolph Co*.: 1 mi. E. Moberly, 25.vi.1973, E.G. Riley, (EGRC, 1); same locality, 17.ix.1972, E.G. Riley, (EGRC, 1); same locality, 18.v.1972, E.G. Riley, (EGRC, 1); same locality, 13.vi.1972, E.G. Riley, (EGRC, 1); same locality, 9.vi.1973, E.G. Riley, (EGRC, 1); same locality, 22.vii.1976, E.G. Riley, (EGRC, 1); same locality, 22.v.1972, E.G. Riley, (EGRC, 1); same locality, 18.v.1972, E.G. Riley, (EGRC, 1); same locality, 11.viii.1976, E.G. Riley, (EGRC, 1); same locality, 29.vii.1972, E.G. Riley, (EGRC, 1); same locality, 25.vii.1973, E.G. Riley, (EGRC, 1); same locality, 4.vii.1972, E.G. Riley, (EGRC, 1); same locality, 4.vii.1984, E.G. Riley, (EGRC, 1); same locality, 6.viii.1971, E.G. Riley, (EGRC, 1); same locality, 4.vii.1984, E.G. Riley, (EGRC, 1); same locality, 22.vii.1972, E.G. Riley, (EGRC, 2); same locality, 15.vi.1972, E.G. Riley, (EGRC, 2); same locality, 18.vii.1976, E.G. Riley, (EGRC, 2); same locality, 29.v.1976, E.G. Riley, (EGRC, 4).

*Reynolds Co*.: Bunker, 6.vi.1972, S.E. Thewke, (UMRM, 1).

*St. Charles Co*.: St. Charles, 20.vi.1899, (WIRC, 3).

*St. Louis Co*.: Ranken, 11.xi.1934, E.P. Meiners, (UMRM, 2); same locality, 23.ix.1934, E.P. Meiners, (UMRM, 2); St. Louis, (DEUN, 2); same locality, (FSCA, 3); same locality, 23.v.1899, (MCZ, 1); same locality, 30.v.1891, (UMRM, 1); same locality, 24.viii.1935, USDA traps, (UMRM, 1); same locality, E.P. Meiners, (WFBM, 1); same locality, M. Schuster, (DEBU, 2); same locality, 4.vii.1904, E. Liljeblad, (UMMZ, 1).

*Vernon Co*.: 12.v.1958, D.J.B. McReynolds, (FSCA, 1).

**MONTANA**

*Hill Co*.:Havre, elev. 757m 48.553909N 109.673566W, 1.vi-16.ix.2010, L Funnel w/ sirex, USDA, (MTEC, 1).

*Richland Co*.: near Fairview @ blt 104.0705W 47.8223N, 11.vi-8.vii.1993, R.G. Bramblett, (MTEC, 2); same locality, 26.v-24.vi.1992, R.G. Bramblett, (MTEC, 1).

*Rosebud Co*.: Forsyth, 20.vi.1956, R.C. Froeschner, (MTEC, 2); Rosebud- Far West, 19.vii.1990, D.L. Gustafson, (MTEC, 2); same locality, 13.vii.1990, D.L. Gustafson, (MTEC, 4); Tongue R. - Ashland, 6.vi.1991, D.L. Gustafson, (MTEC, 2); same locality, 11.viii.1991, D.L. Gustafson, (MTEC, 2).

*Valley Co*.: Bjornberg Bridge, 8.viii.1991, D.L. Gustafson, (MTEC, 2).

**NEBRASKA**

no other data, (MCZ, 2).

*Cass Co*.: Camp Kitaki, nr. Louisville, 1.v.1987, (DEUN, 1); Plattsmouth, (DAPC, 1).

*Colfax Co*.: 4 mi W & 3 mi S. Schuyler, 18.vi.1990, D. Schmidt, incandescent light trap, (DEUN, 1); 4 mi W & 4 mi S. Schuyler, 10.vi.1990, D. Schmidt, pitfall trap, (DEUN, 1).

*Cuming Co*.: West Point, 19.ix, (DEUN, 1); same locality, (DEUN, 5); same locality, 14.x, (DEUN, 1); same locality, v.1888, (DEUN, 1).

*Douglas Co*.: Omaha, 7.vii.1913, L.T. Williams, (DAPC, 1).

*Fillmore Co*.: Fairmont, 3.vii.1912, G.W. Deming, (DEUN, 26); same locality, 4.viii.1912, G.W. Deming, (DEUN, 6).

*Hall Co*.: Mormon Island Refuge, 11.v.1980, B.C. Ratcliffe, (DEUN, 17).

*Keith Co*.: Lake McConaughy Rec. Area, 27.vii.1990, Carrs, lot 5, (CARR, 1).

*Lancaster Co*.: Lincoln, 10.ix.1969, B.C. Ratcliffe, (DEUN, 1); same locality, iii, (DEUN, 6); same locality, vi, (DEUN, 1); same locality, 14.iv.1900, R.H. Wolcott, (DEUN, 3); same locality, 2.v.1900, R.H. Wolcott, (DEUN, 5); same localtiy, 20.iv.1901, R.H.Wolcott, (DEUN, 1); same locality, 9.vi.1900, R.H. Wolcott, (DEUN, 1); same locality, 18.viii.1935, Darlington, (DEUN, 1); same locality, 21.iii.1912, L. Bruner, (DEUN, 1); same locality, 24.ii.1909, J.T. Zimmer, (DEUN, 1); Roca, (DEUN, 2); Waverley, 3.iv.1912, L.H. Gates, (DEUN, 1).

*Merrick Co*.: Mormon Trail State Wayside, 4.viii.1996, R.S. Miller family, (MTEC, 24).

*Nemaha Co*.: Brownville, 9.v.1967, B.C. Ratcliffe, taken under bark, (DEUN, 1).

*Sarpy Co*.: Fontanelle Forest, 11.vii.1969, B.C. Ratcliffe, (DEUN, 2); Pontenelle For., 20–27.vi.1998, M.A. Goodrich & C.A. Springer, Malaise trap, (EIUC, 1).

*Saunders Co*.: Venice, 14.vi.1957, R.C. Funk, (EIUC, 1); Ashland, (DEUN, 1).

*Sheridan Co*.: Rushville, 26.vii.1990, Carrs, lot 1, (CARR, 1).

*Sioux Co*.: Monroe Canon [sic?], 15.viii.1903, J.T. Zimmer, (DEUN, 1).

*Thomas Co*.: Halsey, 29–31.vii.1973, H. & A. Howden, (CNC, 1).

**NEW HAMPSHIRE**

*Rockingham Co*.: Hampton, 3.vii.1932, S. Albert Shaw, (DENH, 1); 1 mi W Odiorne Pt. , 17–19.viii.1983, D.S. Chandler, Malaise trap, (DENH, 1); same locality, 20–23.viii.1983, D.S. Chandler, Malaise trap, (DENH, 1); 4 mi W Durham, 17–23.v.1982, R.M. Reeves, uv trap, (DENH, 1); same locality, 6–8.vii.1982, R.M. Reeves, Malaise trap, (DENH, 1).

*Strafford Co*.: Durham, 10.v.1970, W.J. Morse, lights, (DENH, 1).

*Counties unknown*: Dorex, 1.vii.1933, D.G. Mathos, (DENH, 1).

**NEW JERSEY**

no other data, (LACM, 3); no other data, (MCZ, 1); no other data, (AMNH, 3).

*Bergen Co*.: Oradell Res., 27.x.1968, S. Malcolm, (AMNH, 2); Oakland, 29.vi.1963, *Panus rudis*, (MCZ, 1); Oradell Res., 27.x.1968, S. Malcolm, (UCMS, 4); Fort Lee, 30.iv.1926, (MCZ, 1); Ft. Lee, iv, (CUIC, 2); same locality, (CUIC, 1); River Edge, 26.vi.1957, Rosenberg collection, (USNM, 1); Rutherford, 28.vii.1973, R.L. Jacques, Jr., (FSCA, 1).

*Burlington Co*.: Medford, 20.v.1939, E.J.F. Marx, (AMNH, 1); Mt. Holly, 4.vii.1928, E.J.F. Marx, (AMNH, 1); same locality, 20.v.1927, E.J.F. Marx, (AMNH, 1); same locality, 3.vii.1927, E.J.F. Marx, (AMNH, 1); Riverton, H.B. Weiss, (AMNH, 1); same locality, 5.v.1921, R.J. & M.B. Sim, (USNM, 1); same locality, 18.ii.1925, J.B. Cronin, (USNM, 1); Chatsworth, 4.iv.1929, (AMNH, 1); Medford Township Lake Pine, 14.viii.1974, D.C. Rentz, (USNM, 1); Retreat, 3.vi.1974, A. Newton, under hdwd. bark, (MCZ, 2); Rancocas Woods, 23.iii.1987, S.M. Clark, (BYUC, 2)

*Cape May Co*.: 1 mi. W. Rio Grande, 27.vii.1975, C. L. Smith, ex: fungus on *Mimosa* stump, (UGAC, 1).

*Essex Co*.: Irvington, 28.vii, E.L. Dickerson, (AMNH, 2); same locality, 28.vii, (AMNH, 1); Newark, (MCZ, 6).

*Gloucester Co*.: Paulsboro, 28.vi.1958, H.L. Dozier, (FSCA, 1); Malaga, 14.vi.1913, (USNM, 1).

*Mercer Co*.: 24.v, H.B. Weiss, (AMNH, 1).

*Middlesex Co*.: Monmth Jc, H.B. Weiss, (AMNH, 1); [.i] , E.L. Dickerson, (AMNH, 1).

*Monmouth Co*.: SandyHook, 28.ix.1910, (AMNH, 1); same locality, 28.ix.1910, (AMNH, 1).

*Morris Co*.: Chatham, 22.i.1942, W.F. Rapp, Jr., (INHS, 4).

*Ocean Co*.: New Egypt, 9.iv.1969, S. Malcolm, (UCMS, 1); Lakehurst, 3.v.1970, V. Sapienza, Pine barrens under bark of log, (AMNH, 1); same locality, 28.iv.1940, (FMNH, 1); Wrangle Brook Rd., Lakehurst, 23.viii.1955, D.M. Anderson, (CUIC, 3); same locality, 13.viii.1955, D.M. Anderson, (CUIC, 1); same locality, 17.viii.1955, D.M. Anderson, (CUIC, 1); same locality, 11.viii.1955, D.M. Anderson, (CUIC, 3); same locality, 6.viii.1955, D.M. Anderson, (CUIC, 1); same locality, 8.viii.1955, D.M. Anderson, (CUIC, 1); same locality, 27.viii.1955, D.M. Anderson, (CUIC, 1).

*Passaic Co*.: Paterson, 23.iii, J.A. Grossbeck, (AMNH, 1); same locality, 7.vii, J.A. Grossbeck, (AMNH, 1).

*Salem Co*.: Auburn, 10.vii.1978, P. Bélanger, (CMNC, 1).

*Union Co*.: Rahway, 18.iv., (AMNH, 1); same locality, 1.viii, (AMNH, 1).

*Counties unknown*: Huguenot, 18.ix.1939, (FMNH, 1); ProSpeytcwn, 5.xii.1915, Schalt, (AMNH, 1); Sprnofld, H.B. Weiss, (AMNH, 2).

**NEW MEXICO**

Ulke Collection, (CMNH, 2).

*Roosevelt Co*.:Portales, Eastern New Mexico University campus, 12.ix.2009, ex. fungus on dying willow, at night, D.A. Pollock, (DAPC, 1).

**NEW YORK**

no other data, (CUIC, 2), (AMNH, 1); 23.viii.1939, (FMNH, 1).

*Bronx Co*.: Bronx, 27.iii.1896, (CUIC, 1); NY van Cortlant Pk., 14.ix.1963, T. Hlavac, (AMNH, 1); V.Cortlandt Pk., 25.iii.1951, P. Vaurie, (AMNH, 1); Van C Pk, (AMNH, 1); same locality, (AMNH, 1); Van Cortland Park, 18.xi.1939, (FMNH, 1); same locality, 2.v.1939, (FMNH, 1); same locality, 19.v.1939, (FMNH, 1); same locality, 23.viii.1939, (FMNH, 1); same locality, 12.v.1939, (FMNH, 1); same locality, 26.ix.1939, (FMNH, 2); same locality, 10.v.1941, (FMNH, 2); same locality, L.B. Woodruff, (AMNH, 1).

*Columbia Co*.: Clermont, 5.xi.1934, H. Dietrich, (CUIC, 4); Nevis, 5.xi.1934, H. Dietrich, (CUIC, 1).

*Erie Co*.: Buffalo, (CUIC, 1).

*Nassau Co*.: [Roslyn, L.I.], 8.ix.1930, J.N. Belkin, (USNM, 1); Garden City, 10.viii.1979, Burke Smith Jr., (FMNH, 1).

*Niagara Co*.: Olcott, 10.v.1925, H. Dietrich, (CUIC, 7); same locality, 10.viii.1922, H. Dietrich, (CUIC, 1); same locality, 9.xii.1923, H. Dietrich, (CUIC, 5); same locality, 14.viii.1923, H. Dietrich, (CUIC, 2); same locality, 15.viii.1922, H. Dietrich, (CUIC, 1); same locality, 14.viii.1927, H. Dietrich, (CUIC, 1); same locality, 4.ix.1927, H. Dietrich, (CUIC, 1); same locality, 6.v.1928, H. Dietrich, (CUIC, 1); same locality, 4.vii.1925, E.W. Mank, (CUIC, 1).

*Onondaga Co*.: Elbridge, 13.vi.1940, N.M. Downie, (FMNH, 1); same locality, 21.vi.1940, N.M. Downie, (FMNH, 1); 22.v.1948, N.M. Downie, (AMNH, 1); Syracuse, 4.vi.1965, D.L. Stephan, (CUIC, 1); same locality, 10.v.1980, A. M. O’Brien, (UMMZ, 1).

*Queens Co*.: Corona LI, 17.vii.1930, C.L. Ragot, (FMNH, 1); Flushing, 29.xii.1918, L.B. Woodruff, (AMNH, 1); Jamaica L.I., L.B. Woodruff, (AMNH, 6); same locality, 18.v., (CUIC, 1).

*Richmond Co*.: Staten Island, v, (CUIC, 2); same locality, 15.ix, (CUIC, 1); Staten Island, Great Kills, 1.iv.1939, (FMNH, 1); same locality, 5.v.1940, (FMNH, 1); same locality, 25.ii.1939, (FMNH, 1); Staten Island, Richmond Val., 23.iv.1939, (FMNH, 1).

*Rockland Co*.: Antrim, 26.vi.1940, C.A. Frost, coll. on fungus, (CUIC, 1).

*St. Lawrence Co*.: Rossie, 11.viii.1959, N.M. Downie, (FMNH, 1); same locality, 1.ix.1941, N.M. Downie, (UCRC, 2).

*Schuyler Co*.: Watkins Glen, 26.v.1975, E.R. Hoebeke, (CUIC, 1).

*Suffolk Co*.: Bellport L.I., A. Nicolay, (AMNH, 1); Greenpoint, L.I., 25.i.1933, R. Latham, (CUIC, 1); same locality, 1.vi.1940, R. Latham, (CUIC, 1); same locality, 6.v.1940, R. Latham, (CUIC, 1); Long Island, Riverhead, 5.vi.1950, V.M. Kirk, (NDSU, 1); Monlauk, L.I., 24.x.1945, R. Latham, (CUIC, 1); Napeague, L.I., 6.viii.1945, R. Latham, (CUIC, 1); Noyack, L.I., 12.v.1932, R. Latham, (CUIC, 1); Orient, R. Latham, (CUIC, 2); Orient, L.I., 24.vii.1937, R. Latham, (CUIC, 1); same locality, 10.v.1936, R. Latham, (CUIC, 1); same locality Orient, L.I., 2.iv.1933, R. Latham, (CUIC, 1); same locality, 9.v.1937, R. Latham, (CUIC, 1); Riverhead, L.I., 8.ix.1950, R. Latham, (CUIC, 1); same locality, 9.vi.1946, R. Latham, (CUIC, 1); Sag Harbor, L.I., 1.viii.1929, R. Latham, (CUIC, 1). Wyandanch L.I., 18.iv.1915, F.M. Scholl, (AMNH, 1); East Hampton, L.I., 27.vi.1966, A.J. Kistler, (DENH, 1); same locality, 30.v.1966, A.J. Kistler, (DENH, 1).

*Tompkins Co*.: 27 Perry City Road, Ithaca, 3.vii.1980, G. Swenson, light trap, (CARR, 1); Freeville, 22.vi.1916, H. Dietrich, (CUIC, 1); Ithaca, 25.vi.1949, (USNM, 1); Ithaca, Lick Brook, 23.v.1929, (CUIC, 1); Ithaca, Six Mile Creek, 21.vi.1956, C.B. Knowlton Jr., (CUIC, 1); Renwick, Ithaca, 17.xi.1917, H. Dietrich, (CUIC, 2); Cornell Campus, Ithaca, 29.v.1982, S.W. Nichols, (CUIC, 1); Cornell University, Beebe Lake, 21.iv.1987, F. Guarneri, under loose bark, (CUIC, 2); Ithaca, 25.ix.1977, E.R. Hoebeke, ex fungus, (CUIC, 24); same locality, 10.vii.1987, E.R. Hoebeke, (CUIC, 1); same locality, 20.vii.1975, (CUIC, 1); same locality, 21.vii.1976, (CUIC, 1); same locality, 12.vii.1986, E.R. Hoebeke, (CUIC, 2); same locality, 17.vii.1984, E.R. Hoebeke, (CUIC, 1); Ithaca, von Cramm Hall, 16.v.1991, Z.H. Falin, at UV light, (ZHFC, 1).

*Westchester Co*.: Fleetwood, 3.vi.1934, C.L. Ragot, (FMNH, 1); Rochelle, 21.vii.1953, L. Lacey, (AMNH, 1); same locality, 21.vi.1953, L. Lacey, (AMNH, 1); Yonkers, 15.iv.1936, H. Dietrich, (CUIC, 1); same locality, 2.xi.1939, (FMNH, 1).

*Wyoming Co*.: Pike, (FMNH, 4).

*Counties unknown*: Bear Mt., 4.vii.1948, C.H. Curran, (AMNH, 1); same locality, 30.vii.1945, C.H. Curran, (AMNH, 2); same locality, 19.vii.1945, C.H. Curran, (EIUC, 1); same locality, 28.vii.1945, C.H. Curran, (AMNH, 1); same locality, 30.vii.1945, C.H. Curran, (AMNH, 1); Cook Falls, 6.viii.1929, C.L. Ragot, (FMNH, 1).

**NORTH CAROLINA**

no other data, (CMNH, 2); no other data, (CUIC, 1); no other data, (MCZ, 2).

*Bladen Co*.: White Lake, 14.iii.1953, H. & A. Howden, injured cypress, (CMNC, 1).

*Buncombe Co*.: Swannanoa Val., 21.vi, (AMNH, 1); same locality, 8.vi, (AMNH, 1).

*Carteret Co*.: Morehead City, 18.v.1966, C. Parsons, *Pleurotus* sp., (MCZ, 4).

*Columbus Co*.: Waccamaw Dam, 18.v.2001, K. Gwillian, swamp forest 34/15/40N 78/31/26W, (DEBU, 1).

*Durham Co*.: 23.ix.1987, S.Y. Lee, (UCBC, 1); Chapel Hill, 25.v.1984, C.B. Barr, mv & bl, (LSU, 1).

*Haywood Co*.: 3 mi N. Dellwood, 19.viii.1972, A. Smetana, (CNC, 1).

*Henderson Co*.: 1952, W. Rosenberg, (USNM, 1).

*Johnston Co*.: Central Crops Res. Station, 26.vii.1981, S.P. Whitney, in mushroom, (FSCA, 2).

*Macon Co*.: Highlands, vi.1888, (MCZ, 3).

*Moore Co*.: Southern Pines, 16.v.1952, H. & A. Howden, oak, (CMNC, 1).

*Rebeson Co*.: Lumberton, 25.ii.1953, H. & A. Howden, (CMNC, 1).

*Wake Co*.: 9.v.1958, D.H. Habeck, (FSCA, 1); Raleigh, St. Road 1371, 15.vii.1981, S.P. Whitney, at uv light, (FSCA, 1); Raleigh, 3.v.1953, H. & A. Howden, (CMNC, 5); same locality, 22.i.1953, H. & A. Howden, under bark dead standing pine, (CMNC, 1); same locality, 19.ii.1953, B.K. Dozier, under bark oak, (FSCA, 2).

**NORTH DAKOTA**

*Burleigh Co*.: 24.vii.1963, (USNM, 1).

*Cass Co*.: 16.ix.1962, D.G. Aarhus, (NDSU, 4); 10.iv.1960, A.W. Anderson, (NDSU, 1); 16.ix.1962, Robert Gordon, (USNM, 7).

*Ransom Co*.: 1 mi. SE McLeod, 19.vii.1963, J.R. Powers, ex *Trametes hispida*, (MCZ, 2).

*Richland Co*.: Walcott, 15.vi.1966, Robert Gordon, uv light, (USNM, 1).

**OHIO**

no other data, (CUIC, 1); Chas Palm, (AMNH, 1).

*Ashland Co*.: 27.vi.1974, (CUIC, 1); Ashland, 28.vi.1958, P.D. Harwood, (PPCD, 3).

*Clark Co*.: Clifton, 19.viii, D.J. & J.N. Knull, (CUIC, 1); Shadywilde Selma, 10.viii.1971, R.S. Boone, Malaise trap se corner big woods, (OSUC, 1); same locality, 1.vi.1971, R.S. Boone, Malaise trap se corner big woods, (OSUC, 1); same locality, 2.vii.1966, R.S. Boone, Hg lamp in yard, (OSUC, 1).

*Clermont Co*.: 18.v.1966, (OSUC, 6); 17.iii.1962, (OSUC, 1).

*Clinton Co*.: 8.viii.1962, R.D. Mathis, (OSUC, 5); 9.vi.1963, F.J. Moore, (OSUC, 1); 18.v.1963, F.J. Moore, (OSUC, 1); 16.iii.1963, F.J. Moore, (OSUC, 1).

*Cuyahoga Co*.: Cleveland, 26.x.1925, J.C. Pallister, (AMNH, 1).

*Erie Co*.: Kelleys Island St. Pk., 8.vii.1981, S.W. Nichols, (CUIC, 9).

*Franklin Co*.: Columbus, 11.vii.1981, M.A. Ivie, under bark, (MTEC, 1); same locality, 21.vii.1917, E. Liljeblad, (UMMZ, 1); Columbus, OSU, ix.1984, R.S. Miller, (MTEC, 1).

*Greene Co*.: 29.iv.1960, D.J. & J.N. Knull, (OSUC, 1).

*Hamilton Co*.: 30.vii.1966, (OSUC, 4); 16.vii.1966, (OSUC, 15); 6.iv.1967, (OSUC, 1).

*Hancock Co*.: Van Buren Land Lab, 23.iv.1988, R.S. Miller family, (MTEC, 2).

*Licking Co*.: 24.iv.1963, F.J. Moore, (OSUC, 1).

*Montgomery Co*.: Dayton, 15.iv.1899, (MCZ, 1).

*Ottawa Co*.: Gibraltar Island, 28.vi.1980, S.W. Nichols, under bark, (CUIC, 3); same locality, 28.vi.1980, S.W. Nichols, at light, (CUIC, 1); same locality, 24.vi.1981, S.W. Nichols, at light, (CUIC, 1); same locality, 13.vii.1979, S.W. Nichols, at light, (CUIC, 1); Middle Bass Island, Haunk’s Pd, 9.vi.1980, S.W. Nichols, (CUIC, 1); S. Bass Isl., Fishery Bay, 30.vi.1983, S.W. Nichols, at light, (CUIC, 1).

*Scioto Co*.: Shawnee St. Pk, 15.vii.1989, R.A. Androw, (DAPC, 1).

*Union Co*.: 1974, Q.D. Wheeler, (CUIC, 4).

*Warren Co*.: 19.v.1963, F.J. Moore, (OSUC, 3).

**OKLAHOMA**

*Adiar Co*.: Westville, 15.vi.1939, Kaiser-Nailon, (CUIC, 1).

*Atoka Co*.: Hwy 69, 13 mi. S. of Atoka, 19.vi.1983, Carrs, lot 5, (DAPC, 2).

*Beckham Co*.: Sayre, 8.vii.1937, Standish-Kaiser, (FMNH, 1).

*Bryan Co*.: Blue, 10.vi.1939, Kaiser-Nailon, (CUIC, 2); same locality, 10.vi.1939, Kaiser-Nailon, (MCZ, 1).

*Caddo Co*.: 5 Mi. S. Hinton red Rock Cyn. State Park, 5.v.1986, L. Herman, (AMNH, 3).

*Cherokee Co*.: Ellerville, 20.vi.1937, Standish-Kaiser, (FMNH, 1).

*Cleveland Co*.: 1 mi. NW Noble, 4.iv.1976, W.D. Shepard, ex *Pleurotis* sp., (CAS, 3); Norman, 9.viii.1976, W.D. Shepard, light trap, (CAS, 1).

*Garfield Co*.: 10.vii.1977, John F. Reinert, (USNM, 1); 12.vii.1977, John F. Reinert, (USNM, 1).

*Grady Co*.: Tuttle, 8.v.2005, B. Baldwin, Malaise trap, (BBC, 1); same locality, 20.viii.2005, B. Baldwin, Malaise trap, (BBC, 2); same locality, 17.iv.2005, Malaise trap, (BBC, 1); same locality, 29.iv.2004, B. Baldwin, (BBC, 1); same locality, 18.v.2006, U.V. light trap, B. Baldwin, (BBC, 1); same locality, 27.ix.2006, Malaise trap, B. Baldwin, (BBC, 1); same locality, 1.viii.2005, U.V. light trap, B. Baldwin, (BBC, 1).

*Latimer Co*.: 3 mi SW Red Oak, 2.vii.1987, D.S. Chandler & K. Stephan, sift oak treehole, (DENH, 1); Gaines Creek, 3 mi SW Damon, 4.vii.1987, D.S. Chandler & K. Stephan, box elder treehole, (DENH, 1); 11–12.xii.1995, M.A. Goodrich & K.H. Stephan, (EIUC, 1); i.1982, K. Stephan, (FSCA, 1); iv.1983, K. Stephan, (FSCA, 1); ix.1981, K. Stephan, (FSCA, 1); xi.1982, K. Stephan, (FSCA, 1); ii.1982, K. Stephan, (FSCA, 1); 11–12.xii.1995, M.A. Goodrich & K.H. Stephan, (EIUC, 1); 5 mi. W. Red Oak, x.1980, K. Stephan, (FSCA, 2); county records only, all K. Stephan, FIT, viii.2002, (TAMU, 3); ix.2002, (TAMU, 2).

*Lincoln Co*.: Eby’s Ranch, 1 mi. N, 1 mi W. Sparks, 8.v.1990, J.D. McCarty, (UCBC, 1).

*Love Co*.: Oswalt, 3.vii.1937, Standish-Kaiser, (FMNH, 1).

*Marshall Co*.: 8.vii.1969, R.H. Arnett, Jr., lot 1000, blacklight, (FSCA, 1); Willis, 1.x.1977, W.D. Shepard, on fungus under bark, (CAS, 1).

*Mayes Co*.: Spavinaw, 18.vi.1937, Standish-Kaiser, (FMNH, 1).

*Muskogee Co*.: Ft. Gibson, 21.vii.1937, Standish-Kaiser, (FMNH, 2).

*Okfuskee Co*.: Okemah, 11.iv.1972, Joan Jottius, marsh, (AMNH, 1).

*Oklahoma Co*.: Oklahoma City, 1966, Rosenberg collection, (USNM, 1).

*Osage Co*.: West Bartlesville, 11.vii.1981, K. Burnham, (BYUC, 1)

*Payne Co*.: 1960, John F. Reinert, (USNM, 1); 9.xi.1926, W.J. Brown, (CNC, 1); Stillwater, 20.ii.1936, M. Maxwell, (FMNH, 1); same locality, 6.iii.1936, M. Maxwell, (FMNH, 1); same locality, 29.ii.1936, M. Maxwell, (FMNH, 1); same locality, 6.iii.1936, M. Maxwell, (FMNH, 1); same locality, 10.v.1954, G.P. Carpenter, (FSCA, 1).

*Pontotoc Co*.: Ada, 16.vii.1937, Standish-Kaiser, (FMNH, 1).

*Roger Mills Co*.: Cheyenne, 7.vi.1937, Standish-Kaiser, (FMNH, 1).

*Tillman Co*.: Grandfield, 5.vii.1937, Standish-Kaiser, (FMNH, 1).

*Tulsa Co*.: Tulsa, 21.vi.1963, Carrs, (CARR, 5) same locality, 21.vi.1963, Carrs, (DAPC, 1); same locality, 12.ii.1939, (FSCA, 1).

*Wagoner Co*.: Ft. Gibson Res., 23.vii.1976, W.D. Shepard, at gasoline lantern, (CAS, 1).

*Counties unknown*: Texoma State Park, 11.vi.1994, W.F. Chamberlain, at light, (TAMU, 1).

**PENNSYLVANIA**

Ulke Collection, (CMNH, 1); no other data, (CUIC, 1); no other data, (LACM, 3); no other data, (MCZ, 6).

*Allegheny Co*.: no other data, (CMNH, 5); 24.viii.1895, E.A. Klages, (CUIC, 3); Mt. Lebanon, vii.1932, (CMNH, 1); Pittsburgh, 20.vi, (CMNH, 7); same locality, 16.vi, (CMNH, 1); same locality, vii, (CMNH, 2); same locality, 21.vi, (CMNH, 1); same locality, 25.vi, (CMNH, 1); same locality, 2.vi, (CMNH, 1); same locality, 30.vii, (CMNH, 1); same locality, 27.vi, (CMNH, 1); same locality, 15.vii, (CMNH, 1); same locality, 20.vii, (CMNH, 1); same locality, 7.vi, (CMNH, 2); same locality, 28.vi, (CMNH, 1); same locality, 12.vi, (CMNH, 1); same locality, (CMNH, 12); same locality, 30.v, (CMNH, 1); same locality, vii, (CMNH, 1); same locality, 20.vi, (CMNH, 1); same locality, ix, (CMNH, 1); same locality, 16.vii, (CMNH, 1); same locality, 6.viii.1904, H. Kahl, (CMNH, 1); Pittsburgh, Banksville, 27.vi.1996, D.A. Pollock, on fungus at night, (DAPC, 3); same locality, 6.vii.1996, D.A. Pollock, on dead log at night, (DAPC, 4); same locality, 14.vii.1996, D.A. Pollock, on fungus on dead trees at night, (DAPC, 1); Pittsburgh, Beechview, 17–19.v.1996, D.A. & C.G.W. Pollock, at uv light, (DAPC, 1); Pittsburgh, Fern Hollow, 13.viii.1904, H. Kahl, (CMNH, 2); Pittsburgh, Guyasuta Run, 12.ix.1912, H. Kahl, (CMNH, 1); Upper St. Clair Twp., 13.ix.1958, (UCBC, 5); same locality, 8.iv.1959, (UCBC, 1); same locality, 28.iii.1959, (UCBC, 1); Allegheny, (CUIC, 1); same locality, (MCZ, 1); Mt. Lebanon [= Pittsburgh], ix.1930, R.J. Simon, (CMNH, 3); Wall, 26.vii.1925, (CNC, 2).

*Berks Co*.: Lenhartsville, 9.vii.1946, P. Vaurie, (AMNH, 1).

*Bucks Co*.: 7.x, (CMNH, 3).

*Butler Co*.: Renfrew, 5 km SW Butler, 6.vi.1992, R. Walters, (CMNH, 1).

*Centre Co*.: Bellefonte, 12.v.1937, V.M. Kirk, (NDSU, 1); Spring Mills, 11.vi.1979, P.H. Adler, under bark, (USNM, 1).

*Chester Co*.: Chadds Ford, 6.vii.1987, M. & A. O’Brien, (UMMZ, 3).

*Cumberland Co*.: New Cumb[erland], 6.vii.1941, V.M. Kirk, (NDSU, 2).

*Dauphin Co*.: Harrisburg, 13.vi.1908, (INHS, 1); same locality, 10.v.1941, V.M. Kirk, (NDSU, 2); Hershey, 25.iv.1990, W.H. Yackley, (CMNH, 1).

*Delaware Co*.: Haverford College, 28.ii.1960, J.I. Richardson, (UGAC, 1); same locality, 18.iii.1960, J.I. Richardson, (UGAC, 1); same locality, 21.ii.1960, J.I. Richardson, (UGAC, 1).

*Erie Co*.: Erie, 17.x.1963, W.H. Yackley, (CMNH, 1).

*Greene Co*.: 5 km SSW W. Finley, Enlow Fork Wheeling Creek, 260 m, 18.v.1989, J.E. Rawlins, (CMNH, 1).

*Lancaster Co*.: 6.viii.1941, W.H. Yackley, (CMNH, 1); Pequea, 9.vi, (INHS, 1).

*Philadelphia Co*.: Philadelphia, 3.i.1897, (FMNH, 1); same locality, 23.iii.1895, (FMNH, 1); same locality, 24.ii.1895, (FMNH, 2); Philadelphia, Frankford, (WIRC, 2); Philadelphia, Fairmont Park, 11.x.1941, W.H. Yackley, (CMNH, 1).

*Westmoreland Co*.: Jeannette, H.G. Klages, (CMNH, 25); same locality, (CMNH, 8); same locality, vii, (CMNH, 6); same locality, 20.vi, H.G. Klages, (CMNH, 1); same locality, 6.vii, H.G. Klages, (CMNH, 1); same locality, 4.vii, (CMNH, 1); same locality, vi, (CMNH, 1); same locality, 14.ix, H.G. Klages, (CMNH, 1); same locality, 3.vii, (CMNH, 1); same locality, 24.v, (CMNH, 1); same locality, 6.ix, H.G. Klages, (CMNH, 1); same locality, 10.v, (CMNH, 1); same locality, 21.vi, H.G. Klages, (CMNH, 1); same locality, 3.ix, H.G. Klages, (CMNH, 1); same locality, 2.ix, H.G. Klages, (CMNH, 1); same locality, 10.vi, H.G. Klages, (CMNH, 1); same locality, 21.vi, (CMNH, 6); same locality, vii, (CMNH, 2); same locality, 14.ix, H.G. Klages, (CMNH, 1); same locality, 23.vi, (CMNH, 1); same locality, 4.vii, H.G. Klages, (CMNH, 1); same locality, vii, (CMNH, 2); same locality, 9.ix, H.G. Klages, (CMNH, 1); same locality, 14.vi, H.G. Klages, (CMNH, 1); same locality, 16.vi, (CMNH, 1); same locality, v, (CMNH, 1); same locality, 20.vi, H.G. Klages, (CMNH, 1); same locality, Jeannette, vi, (CMNH, 1); same locality, vii, Knechtel, (UMRM, 3).

*Counties unknown*: Chestnut Hill, vi.29, (MCZ, 1); St. Vinc., (CMNH, 2).

**RHODE ISLAND**

*Newport Co*.: Portsmouth, 18.v.1907, N.S. Easton, (MCZ, 1); Tiverton, 27.viii.1942, N.S. Easton, (MCZ, 1).

*Providence Co*.: Cranston, suburbia, 25.v.1995, D.S. Sikes, (DSSC, 1); same locality, 20–24.v.1995, D.S. Sikes, (DSSC, 1); same locality, 16–20.v.1995, D.S. Sikes, (DSSC, 1); same locality, 4.viii-1.ix.1995, D.S. Sikes, (DSSC, 2).

**SOUTH CAROLINA**

no other data, (MCZ, 3).

*Abbeville Co*.: Antreville, 28.iv.1973, L.L. Lampert, under bark, (FSCA, 1).

*Beaufort Co*.: Beaufort, 18.iv.1893, G.D. Bradford, (AMNH, 4).

*Chester Co*.: Great Falls, 16.iv.1965, V.M. Kirk, fungus, (NDSU, 1).

*Clarendon Co*.: Manning, 12.viii.1945, (MSUC, 6).

*Dorchester Co*.: Summerville, 3.vi.1950, W.F. Chamberlain, (TAMU, 4).

*Florence Co*.: Florence, 4.v.1953, V.M. Kirk, (NDSU, 1); same locality, 12.ii.1963, V.M. Kirk, hollow tree, (NDSU, 4); same locality, 23.x.1961, V.M. Kirk, under dead oak bark, (NDSU, 3); same locality, 30.v.1954, V.M. Kirk, (NDSU, 1); same locality, 27.x.1961, V.M. Kirk, hollow tree, (NDSU, 1); same locality, 15.v.1957, V.M. Kirk, fungus, (NDSU, 3); same locality, 20.v.1963, V.M. Kirk, (NDSU, 1).

*Greenville Co*.: Greenville, 5.v.1982, R.S. Peigler, at light, (LACM, 1); same locality, 15.v.1982, R.S. Peigler, at light, (LACM, 1).

*Kershaw Co*.: Camden, 23.vi.1929, Darlington, (MCZ, 1).

*Oconee Co*.: W. Union, 24.v.1967, C. Parsons, (MCZ, 2).

*Pickens Co*.: Clemson, 30.iv.1975, J.R. Ables, (TAMU, 1); Calhoun, Clemson, 7.vi.1950, W.F. Chamberlain, (TAMU, 1).

*Sumter Co*.: Sumter, 12.v.1973, P.M. Choate, (DENH, 1).

**SOUTH DAKOTA**

*Brookings Co*.: Brookings, 3.v.1966, V.M. Kirk, under log, (NDSU, 1).

*Yankton Co*.: Yankton, 27.vii.1965, V.M. Kirk, (NDSU, 1).

**TENNESSEE**

Chas Palm, (AMNH, 1).

*Cumberland Co*.: 8 km NW Rockwood, 16.xii.1984, W. Steinger, R. Gordon, & D. Whitehead, (USNM, 1).

*Davidson Co*.: Nashville, 4.viii.1915, Wickham, (USNM, 1).

*Hardeman Co*.: Bolivar, 20–30.v.1996, R.D. Ward, (CMNH, 1).

*Knox Co*.: Knoxville, 16.iv.1950, D.W. Pfitzer, (CMNC, 1).

*Lake Co*.: Reelfoot Lake, Walnut Log, 9.v.1986, D.A. Pollock, (DAPC, 1).

*Lauderdale Co*.: Hales Point, Obion River, 19.vi.1996, R.D. Ward, (CMNH, 3).

*Madison Co*.: E. of Jackson, 1959, A.F. Archer, (AMNH, 2).

*McMinn Co*.: Calhoun, 4.xii.1980, R.S. Miller, (MTEC, 1); same locality, 4.xii.1980, M.A. Ivie, rotten log, (MTEC, 1).

*Shelby Co*.: Meeman Biolog. Sta., 20 km N Memphis, 19–25.iv.1990, M. Ritke, (CMNH, 2); Memphis, 19.viii.1947, B. Summerville, (MSUC, 1); Barrville [Barretville?], 19.v.1957, H. & A. Howden, (CMNC, 1); Memphis, 19.vii.1975, L.E. Watrous, (CUIC, 2).

**TEXAS**

Ulke Collection, (CMNH, 1); no other data, (CUIC, 2); no other data, (MCZ, 23); vi-vii.1962, Gary Nichols, (AMNH, 1).

*Anderson Co*.: Engeling Wdlf. Mgt. Ar., 3.vi.1995, A.J. Gilbert and F.G. Andrews, coll. on polypore on dead *Quercus*, (DAPC, 3); same locality, 3.vi.1995, E.G. Riley, (EGRC, 9); same locality, 3.vi.1995, E.G. Riley, (EGRC, 1); Salmon, 10.v.1980, H.R. Burke, (TAMU, 1).

*Angelina Co*.: Angelina Nat’l. Forest, ca. 5.5 mi. SE Zavalla, 31°07'05"N, 94°20'01"W, funnel trap, managed loblolly pine, Clarke, Menard, & Riley, 2–16.v.1996 (TAMU, 1); same data, 17–30.v.1996, (TAMU, 2); same data, 31.v–13.vi.1996, (TAMU, 1).

*Aransas Co*.: Lamar, 7.iv.1987, Carrs, lot 2, (CARR, 1).

*Bastrop Co*.: Bastrop St. Park, 7–21.x.1990, R. Wharton, (DAPC, 1); same locality, 7–21.x.1990, R. Wharton, (TAMU, 4).

*Bexar Co*.: San Antonio, 22.iii.1958, J.F. Lawrence, (MCZ, 1); same locality, 22.v.1981, R.R. Thomas, (TAMU, 13); same locality, 22.iii.1958, J.F. Lawrence, (UCBC, 1); same locality, 16.vii.1955, D.H. Habeck, (FSCA, 1); county only, 12.iv.1958, R. & C. Kendall, (TAMU, 2).

*Blanco Co*.: Cypress Mills, 15.v, (CUIC, 1).

*Brazoria Co*.: 7.4 mi. S. Angleton, Mickey Clement Farm, 6.vi.1975, M.L. Holcomb, J.S. Ashe, (TAMU, 2).

*Brazos Co*.: Bryan, 21.iv.1971, J.C. Schaffner, at light, (TAMU, 1); same locality, 12.v.1974, J.C. Schaffner, at light, (TAMU, 1); same locality, 12.iv.1974, J.C. Schaffner, at light, (TAMU, 1); same locality, 29.iv.1966, J.C. Schaffner, at light, (TAMU, 1); College Station, 4.x.1950, H.J. Reinhard, (TAMU, 1); same locality, 14.ix.1933, H.J. Reinhard, (TAMU, 1); same locality, 29.iii.1951, H.J. Reinhard, (TAMU, 6); College Station, Lick Ck. Pk., 10.vi.1995, E.G. Riley, (TAMU, 1); same locality, 17.vi.1995, E.G. Riley, (TAMU, 1); same locality, 27.iv.1996, E.G. Riley, uv in bottomland forest; 293.2, (TAMU, 2); College Station, Pebble Creek Subdivision, 23.iv.1995, E.G. Riley, Berlese oak duff, (TAMU, 1); Koppe’s Bridge, 5 mi. S. College Station, 4.vi.1995, E.G. Riley, on fungus-covered log, (TAMU, 1); Koppe’s Bridge, 5 mi. S. College Station, 4.vi.1995, E.G. Riley, on fungus-covered log, (TAMU, 1); White Crk Rd. 5 mi. SW College Station, 17–26.vii.1981, R.H. Turnbow, ex malaise trap, (TAMU, 1); College Sta., Riley Estate, 30.58849°N, 96.25366°W, 25–31.v.2009, E. Riley, UV, (EGRC, 1); same locality, 25.iii-2.iv.2010, E. Riley, (EGRC, 1); same locality, 21.iii.2009, E. Riley, UV, (EGRC, 1); same locality, 8–12.ix.2010, E. Riley, UV, (EGRC, 1); College Sta., Riley Estate, 30°35'18"N, 96°15'12"W, 15.ix.2001, E.G. Riley, (EGRC, 1); same locality, 23.v.2002, E.G. Riley, (EGRC, 1); College St., Lick Ck. Pk., 30.56234°N, 96.21333°W, 26.iv.2009, E.G. Riley, (EGRC, 1).

*Brewster Co*.: Big Bend Natl. Pk., North Rosillos Mts., Buttrill Spring, 10–17.vii.1991, R. Vogtsberger, malaise trap, (DAPC, 1); Big Bend Nat. Pk., Hot Springs, 1900’, 29.v.1959, Howden & Becker, taken at light, (CNC, 1).

*Brown Co*.: Lake Brownwood St. Pk., 29.iv.1995, E.G. Riley, uv light; 80, (TAMU, 2).

*Caldwell Co*.: Palmer St. Pk. Ohine, 15.vi.1982, R.S. Miller, at light, (MTEC, 1).

*Cameron Co*.: nr. Sabal Palm Grove Sanct., Paloma Blanca Road, 31.x.1991, T. Carlow & E. Riley, (DAPC, 1); Brownsville, iv, (CUIC, 1); same locality, Wickham, (MCZ, 1); same locality, [Espaga Rch], vi.1928, (USNM, 1).

*Cass Co*.: Daingerfeild St. Pk., 27.v.1977, Riley & LeDoux, blacklight, (EGRC, 1).

*Cherokee Co*.: 6.vi.1952, D.E. Martin, (TAMU, 3).

*Comal Co*.: New Braunfels, (CUIC, 1).

*Dallas Co*.: Dallas, (MCZ, 14); same locality, (MCZ, 3).

*Denton Co*.: Lewisville Dam, Oakland Park, 6.vi.1961, (TAMU, 1); same locality, 6.vi.1961, (TAMU, 1).

*Fort Bend Co*.: Brazos Bend St. Pk., 18.iv.1999, uv light buckeye-sycamore forest, E.G. Riley, (TAMU, 2); same locality, 10.iv.1999, E.G. Riley, Lindgren funnel trap, yaupon-oak forest, (TAMU, 1); same locality, 3.vii.1999, B. Raber, FIT, (TAMU, 1).

*Galveston Co*.: Friendswood 2 mi. NE, 13.i.1945, (UCBC, 24).

*Gonzales Co*.: Palmetto St. Park, 14.v.1985, J. E. Rawlins, (CMNH, 1); same locality, 1–2.x.1973, A. Newton, on gilled mushrooms, (FMNH, 1); same locality, 30.v.1977, Riley & LeDoux, (EGRC, 3); Palmetto St. Pk., 18.vi.1973, A. Newton, (MCZ, 1); Palmetto State Park, 30.v.1983, C.B. Barr, at blacklight, (CBC, 2); same locality, 21.vii.1983, C.B. Barr, at blacklight, (CBC, 1); same locality, 30.v.1983, C.B. Barr, at blacklight, (LSU, 16); same locality, 21.vii.1983, C.B. Barr, at blacklight, (LSU, 1).

*Hardin Co*.: 11.v.1940, R.E. Maxwell, (INHS, 1); 5.5 mi. NW Saratoga, 24.iii.1996, E.G. Riley, Berlese forest litter; 258, (TAMU, 1).

*Harris Co*.: Houston, (DENH, 1); same locality, Wickham, (AMNH, 1).

*Hidalgo Co*.: 8.vi.1958, D.J. & J.N. Knull, (OSUC, 12); Anzalduas Co. Pk., 27.x.1980, Wappes & Downie, (FMNH, 1); Bentsen Rio Grande St. Pk., 23.v.1981, J. Doyen & J. Liebherr, (UCBC, 3); Bentsen-Rio Grande Va. St. Pk., 15–16.x.1988, E.G. Riley, (DAPC, 2); same locality, 15–16.x.1988, E.G. Riley, (TAMU, 8); same locality, 17–18.v.1979, E.G. Riley, (EGRC, 3); McAllen, Valley Bot. Garden, xi.1973, C.C. Porter, malaise trap, (FSCA, 1); Bentsen R.G.V.S.P. (site 1), 26.17830°N, 98.38577°W, 6–19.ix.2008, FIT-ground, J. King & E. Riley, cedar elm forest, (TAMU, 1); Santa Ana NWR (site 4), Observation Tower, 26.07760°N, 98.13805°W, 17.x.2008, E.G. Riley, (TAMU, 1).

*Houston Co*.: 11 mi E. Ratcliff, 24.iv.1976, A. Smetana, (CNC, 8); Big Slough Wndss. Ar., FR 511 & Four C’s trail, Crocket Nat’l Forest, 31°28'N, 95°08'W, 15.iii.1995, E.G. Riley, 251, (TAMU, 1); Ratcliff, 23.iii.1972, R.L. Fischer, (MSUC, 1).

*Jeff Davis Co*.: Fort Davis, 31.v.1959, Howden & Becker, ex fungus, (CNC, 1).

*Kenedy Co*.: just S. of Sarita, 26.x.1992, E.G. Riley, on fungus, (DAPC, 1); same locality, 26.x.1992, E.G. Riley, on fungus, (EGRC, 7).

*Kerr Co*.: Kerrville, 7.iv.1959, Becker & Howden, light trap, (CNC, 1); same locality, 8.iv.1959, Becker & Howden, beating *Quercus*, (CNC, 1); same locality, 17.iv.1959, Becker & Howden, light trap, (CNC, 1); same locality, 5.iv.1959, Becker & Howden, (CNC, 1); same locality, 15.ix.1986, W.F. Chamberlain, (TAMU, 1).

*Live Oak Co*.: Three Rivers, 11.v.1978, UVL, J.E. Wappes, (TAMU, 1).

*Mason Co*.: Streeter, 7.iii.1965, P. Wagner, (TAMU, 1).

*McLennan Co*.: Crawford, 18.iv.1942, O. Sanders, (FMNH, 1).

*Montague Co*.: 4 mi. W. Nocona, 20.vi.1989, R. Morris, (UGAC, 2).

*Montgomery Co*.: Sam Houston Natl. Forest, Stubblefield L., 12.iv.1964, H.R. Burke, (TAMU, 1); Stubblefield Lake Rec Area, 1.v.1980, P.W. Kovarik, (TAMU, 7); Woodlands, 28.iv.1978, J.E. Wappes, (TAMU, 1); same locality, 19.v.1979, J.E. Wappes, (TAMU, 1).

*Palo Pinto Co*.: Possum Kingdom State Park, 14.vi.2002, at light, W.F. Chamberlain, (TAMU, 1).

*Polk Co*.: Seven Oaks, 24.v.1979, N.M. Downie, (FMNH, 2).

*Robertson Co*.: 8 mi. E. Hearne, 1–21.iv.1991, M. Hallmark, Malaise trap, (TAMU, 1).

*Sabine Co*.: 9 mi. E. Hemphill, “Beech Bottom”, 24.vii-6.viii.1989, R. Anderson & E. Morris, Malaise trap in beech-magnolia forest, (TAMU, 1); same locality, 4–9.iv.1989, R. Anderson & E. Morris, Malaise trap in beech-magnolia forest, (TAMU, 2); same locality, 22.v-4.vi.1989, R. Anderson & E. Morris, Malaise trap in beech-magnolia forest, (TAMU, 5); same locality, 13–24.vii.1989, R. Anderson & E. Morris, Malaise trap in beech-magnolia forest, (TAMU, 1); same locality, 17–24.iv.1989, R. Anderson & E. Morris, Malaise trap in beech-magnolia forest, (TAMU, 2); same locality, 10–23.ix.1989, R. Anderson & E. Morris, Malaise trap in beech-magnolia forest, (TAMU, 1); same locality, 13–18.iii.1989, R. Anderson & E. Morris, Malaise trap in beech-magnolia forest, (TAMU, 1); same locality, 29.iv-3.v.1990, R. Anderson & E. Morris, Malaise trap in beech-magnolia forest, (TAMU, 1); same locality, 20.iii-29.iv.1990, R. Anderson & E. Morris, Malaise trap in beech-magnolia forest, (TAMU, 1); same locality, 7–26.x.1989, R. Anderson & E. Morris, Malaise trap in beech-magnolia forest, (TAMU, 1); Moss Creek, Hwy 87, 2 mi. N. of Milam, 23.iv.1990, R.W. Baumann, (BYUC, 2).

*San Augustine Co*.: Piney Woods Conserv. Ctr., 14 mi. SE Broaddus, 15–21.vii.1993, E.G. Riley, malaise trap, (DAPC, 1); same locality, 15–21.vii.1993, E.G. Riley, malaise trap, (TAMU, 4).

*San Patricio Co*.: Welder Wildlife Ref., nr. Sinton, 19.v-9.vi.1975, V. Nealis, (CMNC, 1); Welder Wildlife Refuge, 17 km NE Sinton, 17–25.v.1985, H. & A. Howden, C. Scholtz, (CMNC, 1).

*Smith Co*.: Tyler St. Pk., 10–11.iv.1992, E. Riley & C. Wolfe, (EGRC, 1); Tyler, 11.vii.1937, (TAMU, 1).

*Tarrant Co*.: Benbrook Res. Dutch Branch Pk., 16.vii.1961, (TAMU, 1); same locality, 23.vii.1961, (TAMU, 1); same locality, 16.vii.1961, (TAMU, 1).

*Throckmorton Co*.: Throckmorton, 16.vi.1983, Carrs, lot 3, (CARR, 1).

*Travis Co*.: Austin, 550 ft., 8.x.1983, J.E. Rawlins, (CMNH, 1); Austin, Brackenridge Fld. Sta., 9.x.1984, J.E. Rawlins, (CMNH, 1); same locality, 11–16.x.1985, J. Rawlins & R. Davidson, (CMNH, 1); Austin, Breckenridge Field Laboratory, 140 m, 13.x.1984, J.E. Rawlins, (CMNH, 1); same locality, 8.x.1985, J. Rawlins, (CMNH, 1).

*Tyler Co*.: Kirby State Forest, 30°34'30"N, 94°25'03"W, 27.iv-18.v.2003, E.G. Riley, ground-level flight intercept, (TAMU, 1); same locality, 30.iii-27.iv.2003, E. Riley, ground-level flight intercept, (TAMU, 1); same locality, 20.vii-24.viii.2003, E.G. Riley, elevated FIT, (TAMU, 1).

*Walker Co*., Ellis Prison, 12.vii.1977, W.L. Sterling, mod. malaise trap, (TAMU, 1); same locality, 23.viii.1977, W.L. Sterling, mod. malaise trap, (TAMU, 1); Stubblefield Lake Rec. Area, 1.v.1980, P. Kovarik, (CUIC, 1); Stubblefield Lk. St. Rec. Area, 10.xii.1980, P.W. Kovarik & G.W. Brooks, (TAMU, 1); same locality, 1.xi.1981, P.W. Kovarik, (TAMU, 1).

*Williamson Co*.: Taylor, 5.v.1968, J.E. Hafernik, (TAMU, 1); same locality, 27.iv.1968, J.E. Hafernik, (TAMU, 1); same locality, 7.ix.1968, J.E. Hafernik, (TAMU, 1).

*Wood Co*.: Butler Oxbow Lake, 5.liv.1992, W. Godwin & M. Prater, under rotten bark, (TAMU, 3); ca. 18 mi. N. Hawkins, nr. Little Cypress Creek, 32°49'34"N, 95°09'45"W, 15.v-20.vii.1999, flight intercept trap, sandy area, Gillogly, Godwin & Riley, (TAMU, 1); Godwin Woods, 3.5 mi SW Hainsville, 32°42'30"N, 95°24'36"W, 6.v.2001, W.B. Godwin, (TAMU, 2).

*Countries unknown*:Bastrop St. Pk., 14.iv.1959, H.F. Howden, (CNC, 23); same locality, 26.iii.1950, D.J. & J.N. Knull, (OSUC, 1); Hiwy 37 at Red R, 9.iv.1971, R & E. Froeschner, (USNM, 1); San Marcos, Pecan Park, 23.viii.1969, G.S. Tullock, light trap, (TAMU, 1).

**UTAH**

*Utah Co*.: East Utah Lake, 1.iv.1967, W. Clark, polypore fungus, (DAPC, 1); same locality, 30.vii.1966, W. Clark, (TAMU, 1); same locality, 1.iv.1967, W. Clark, polypore fungus, (TAMU, 1); same locality, 6.iv.1967, D.R. Harris, (BYUC, 1); same locality, 27.iv.1957, under bark and fungi, (BYUC, 5); same locality, 27.ii.1968, W.N. Mathis, rotten log, (BYUC, 1); W. Utah Lake, 10.iv.1966, B.A. Croft, (BYUC, 2); Mapleton, 3.viii.1983, C. Porter, (BYUC, 1); Provo, 14W 5N, 10.v.1984, C.R. Nelson, (BYUC, 1); Provo, 4/10/1962, S.K. Baker, (BYUC, 1); Lehi, Mill Pond, 13.vi.1981, S.M. Clark, (BYUC, 1); Utah Lake shore near the Provo airport, 16.v.1979, S.L. Wood & S.M. Clark, (BYUC, 2).

*Washington Co*.: Zion Natl Park, Oak Creek, 11–13.viii.1981, C.R. Nelson, light, (BYUC, 1)

**VIRGINIA**

no other data, (CUIC, 1).

*Bath Co*.: Warm Spgs., 1.x, (CUIC, 1).

*Chesapeake Co*.: Northwest Riv. Pk, 22.vii.1984, P. Bélanger, (CMNC, 1); Chesapeake, Northwest River Park, 9.vii.1993, S. Laplante, uv light, (SLC, 2).

*Chesterfield Co*.: Pocahontas St. Pk., 27–28.vi.1992, S. Laplante, uv light, (SLC, 2); Afton, 27.vii.1945, (USNM, 2).

*Clarke Co*.: U. Va. Blandy Exp. Farm 2 mi S Boyce 3905N 7810W, 11–25.v.1993, D.R. Smith, Malaise trap, (USNM, 3).

*Covington Co*.: Covington, 6.v.1950, G.H. Nelson, fungus, (WFBM, 1).

*Essex Co*.: 1 mi SE Dunnsville 3752N 7648W, 4–14.v.1993, D.R. Smith, Malaise trap, (USNM, 1); same locality, 26.vii.1991, D.R. Smith, Malaise trap, (USNM, 1); same locality, 1–19.iv.1993, D.R. Smith, Malaise trap, (USNM, 1); same locality, 24.vi-9.vii.1991, D.R. Smith, Malaise trap, (USNM, 1); same locality, 24.vi-9.vii.1992, D.R. Smith, Malaise trap, (USNM, 4); same locality, 14.viii-2.ix.1993, D.R. Smith, Malaise trap, (USNM, 8); same locality, 23.ix-19.x.1993, D.R. Smith, Malaise trap, (USNM, 2); same locality, 29.v-9.vi.1993, D.R. Smith, Malaise trap, (USNM, 1).

*Fairfax Co*.: Alexandria, Holmes Run, 21.ix.1970, J. Powell, (UCBC, 1); Alexandria, Huntley Meadows, 6–16.iv.1991, B.V. Brown, Malaise in oak-maple forest, (DAPC, 1); Burke Center, 25–29.vii.1987, R.D. Ward, (CMNH, 1); Fairfax [George Mason University], 22.vi.1974, R.W. Baumann, (USNM, 2); Turkey Run, 26.viii.1976, Newton & Thayer, under *Tilia* bark, (MCZ, 1).

*Hampton Co*.: Hampton, 2.v.1944, N.M. Downie, (FMNH, 1).

*Loudoun Co*.: Purcellville, 20.vi.1964, E.J. Hambleton, (OSUC, 1); same locality, 4.vi.1970, E.J. Hambleton, (OSUC, 1); same locality, 12.vi.1968, E.J. Hambleton, (OSUC, 1).

*Newport News Co*.: Newport News Pk, 24.vii.1984, P. Bélanger, (CMNC, 1).

*Suffolk Co*.: Suffolk, 1936, W.M. Mann, (USNM, 1).

*Westmoreland Co*.: Oak Grove 3811N 7701W, 11.iv.1992, J.A. Lewis, taken at UV light, (USNM, 1).

*York Co*.: Big Bethel Res., Fire Sta. #2, 1.iv.1978, B.C. Heckert, (BYUC, 3).

*Countries unknown*: Potomac R. shore 10 mi NW Wash DC, 12.iv.1933, H.S. Barber, (USNM, 1).

**VERMONT**

*Bennington Co*.: Manchester, 30.vi.1968, C. Parsons, (MCZ, 3); same locality, 9.vi.1968, C. Parsons, *Polyporus squamosus*, (MCZ, 11).

**WASHINGTON**

*Asotin Co*.: Asotin, 23.vii.1963, W.D. Hunt, under rock, (WFBM, 1).

**WISCONSIN**

no other data, (INHS, 3); vi, (MCZ, 3); no other data, (MCZ, 1).

*Columbia Co*.: Wyocena, 24.v.1917, L.G. Gentner, under bark of poplar, (WIRC, 1).

*Dane Co*.: 11.v.1954, D.H. Habeck, (FSCA, 1); 10.v.1898, (WIRC, 1); no other data, (WIRC, 1); 24.vi.1954, G.E. Haas , light trap, (WIRC, 1); 9.vi.1954, G.E. Haas, light trap, (WIRC, 1); 4.vi.1898, (WIRC, 1); 15.ix.1898, (WIRC, 1); 26.ix.1898, (WIRC, 1); Madison, ix.1968, W. Sieker, (UGAC, 1); Madison, 24.v.1914, A.C. Burrill, (WIRC, 1).

*Dodge Co*.: Beaver Dam, 21.viii.1910, W.E. Snyder, (MCZ, 1; UMMZ, 2); same locality, 22.vi.1917, W.E. Snyder, (AMNH, 3); same locality, 28.viii.1910, W.E. Snyder, (AMNH, 1).

*Green Co*.: Brodhead, 28.iv.1924, S. Moore, (UMMZ, 1).

*Jackson Co*.: T21N, R4W, S33, 16–23.viii.1976, gypsy moth M.T., (WIRC, 1).

*Milwaukee Co*.: Milwaukee, (CUIC, 1); same locality, 14.ix.1915, G.A. Chandler, (WIRC, 1).

*Racine Co*.: 5.viii.1953, D.H. Habeck, (FSCA, 1).

*Sauk Co*.: Sauk City - hills across river, 14.v.1961, G. Lachmund, (UGAC, 1).

*Wood Co*.: Griffith St. Nursery, 4.vii.1947, R.D. Shenefelt, (WIRC, 1); same locality, 2.vi.1949, R.D. Shenefelt, (WIRC, 1); Grand Rapids, 15.vii.1914, J.G. Sanders, at light, (WIRC, 1).

**WEST VIRGINIA**

*Barbour Co*.: Philippi, 17–24.vi.1994, M.D. Bombardiere, Lindgren funnel trap, (DAPC, 1).

*Braxton Co*.: Heaters, 23.vi.1992, S.F. Hutchinson, Lindgren funnel, lumber yard, (DAPC, 1); same locality, 31.viii-1.ix.1992, S.F. Hutchinson, Lindgren funnel trap, lumber yard, (PPCD, 1); same locality, 23.vi.1992, S.F. Hutchinson, Lindgren funnel, lumber yard, (PPCD, 2).

*Greenbrier Co*.: Rupert, 20–21.vii.1992, S.F. Hutchinson, Lindgren funnel trap, lumber yard, (DAPC, 1); same locality, 20–21.vii.1992, S.F. Hutchinson, Lindgren funnel trap, lumber yard, (PPCD, 1); White Sulphur, A. Fenyes, (MCZ, 1).

*Hancock Co*.: Congo, 15.vii-7.viii.1996, M.D. Bombardiere, Lindgren funnel trap, (PPCD, 1).

*Jackson Co*.: Cottageville, 1.vi.1989, T. Sutton, bark beetle trap, (PPCD, 1); Evans, 4–5.viii.1992, S.F. Hutchinson, Lindgren funnel trap, lumber yard, (PPCD, 1); same locality, 17–18.viii.1992, S.F. Hutchinson, Lindgren funnel trap, lumber yard, (PPCD, 1); same locality, 20–21.vii.1992, S.F. Hutchinson, Lindgren funnel trap, lumber yard, (PPCD, 1); same locality, 1.ix.1992, S.F. Hutchinson, Lindgren funnel trap, lumber yard, (PPCD, 1).

*Marshall Co*.: Moundsville, 10.vii-6.viii.1996, M.D. Bombardiere, Lindgren funnel trap, (PPCD, 1).

*Mineral Co*.: New Creek, 2–9.vi.1995, C.E. Ingle, Lindgren funnel trap, (PPCD, 1).

*Preston Co*.: Horseshoe Run, 23.vi.1992, S.F. Hutchinson, Lindgren funnel trap, Coastal Lumber Yard, (PPCD, 1).

*Putnam Co*.: 1 mi. SE Winfield, 13–21.vi.1995, R.A. Meyers, Lindgren funnel trap, (DAPC, 1); Winfield, 24–31.v.1995, R.A. Meyers, Lindgren funnel trap, (PPCD, 2).

*Ritchie Co*.: 2 mi. N. Oxbow, 8–14.vi.1995, R.A. Meyers, Lindgren funnel trap, (DAPC, 1).

*Roane Co*.: Spencer, 1.ix.1992, S.F. Hutchinson, Lindgren funnel trap, lumber yard, (DAPC, 1); same locality, 20–21.vii.1992, S.F. Hutchinson, Lindgren funnel trap, lumber yard, (PPCD, 1); same locality, 1.ix.1992, S.F. Hutchinson, Lindgren funnel trap, lumber yard, (PPCD, 2).

*Tyler Co*.: 2 mi. N. Kidwell, 21.iii-23.iv.1997, L. Miller, Theysohn trap, (PPCD, 1).

*Webster Co*.: Hacker Valley, 17–18.viii.1992, S.F. Hutchinson, Lindgren funnel trap, lumber yard, (PPCD, 2).

**WYOMING**

*Goshen Co*.: Torrington, 29.v.1991, D. Schmidt, under log, (DEUN, 1).

### *Eustrophopsis brunneimarginatus* (Dury)

**UNITED STATES OF AMERICA**.

**ALABAMA**

*Dale Co*.: Ft. Rucker Mil. Res., 1–4.viii.1994, R. Turnbow, flight intercept trap, (RTC, 1).

*Madison Co*.: Huntsville, Monte Sano St. Pk.1965, Rosenburg collection, (USNM, 1).

**ARKANSAS**

*Polk Co*.: Mena, 24.viii.2010, Malaise trap, B. Baldwin, (BBC, 1); same locality, 2.ix.2010, Malaise trap, B. Baldwin, (BBC, 1).

*Pulaski Co*.: Little Rock, 14.viii.2003, B. Baldwin, blacklight trap, (BBC, 1).

**FLORIDA**

*Liberty Co*.: Torreya St. Pk., 24.vi.1989, R. Turnbow, (RTC, 1); Apal. Blffs & Rav. Pres., N 30 29 42.66, W 84 58 42.46, 183 ft., malaise trap mxd hrdwd/closed canopy seepage slope / ravine, coll. Preserve Staff, 9.vii.2008, (UCFC, 5); 24.vii.2008, (UCFC, 2), 29.vii.2008, (UCFC, 3); 14.vi.2009, (UCFC, 1); 12.vii.2009, (UCFC, 3); 16.viii.2009, (UCFC, 4).

**GEORGIA**

*Monroe Co*.: Forsyth, 1.vi.1970, F.T. Naumann, Malaise trap, (CNC, 1).

**ILLINOIS**

*Edgar Co*.: 4mi SSE Kansas, 4–11.viii.2000, M.A. Goodrich & H.W. Montgomery, Malaise trap, (DAPC, 1).

*Wabash Co*.: Beall Woods N. P. 15–23.viii.1996, M.A. Goodrich, Malaise trap, (DAPC, 1); same locality, 11–25.vi.1997, M.A. Goodrich, Malaise trap, (EIUC, 1).

**INDIANA**

*Tippecanoe Co*.: 13.viii.1958, N.M. Downie, (FMNH, 1).

**LOUISIANA**

*Grant Par*.: Stuart Lake Rec. Area, 22–29.vii.1982, E.G. Riley, black light trap, (LSU, 1).

*Natchitoches Par*.: Kisatchie Nat. For., nr. Red Bluff campgrd., 15.v.1982, E.G. Riley, (EGRC, 1).

**MISSISSIPPI**

*Tishomingo Co*.: Tishomingo St. Pk., 29.vii.1977, S.E. Hurdle, (UMIC, 1).

**MISSOURI**

*Randolph Co*.: 1 mi. E. Moberly, 5.vi.1972, E.G. Riley, (DAPC, 1); same locality, 11.viii.1972, E.G. Riley, (EGRC, 1).

**NORTH** **CAROLINA**

*Rockingham Co*.: Reidsville, 2–12.viii.1973, J.S. Ashe, at light, (TAMU, 1).

**NEW** **HAMPSHIRE**

*Coos Co*.: Rockaway, 14.vii.1917, L.B. Woodruff, beach washed up, (AMNH, 1).

**OKLAHOMA**

*Latimer Co*.: xi.1981, K. Stephan, (FMNH, 1); vi.2001, K. Stephan, UV, (TAMU, 1); Red Oak, 26.ix.1976, K. Stephan, (TAMU, 1).

**SOUTH** **CAROLINA**

*Anderson Co*.: Pendleton 250 m, 17–27.vi.1987, malaise, mature hardwood forest, (DENH, 1).

**TEXAS**

*Anderson Co*.: Salmon, 10.v.1980, H. R. Burke, (TAMU, 1).

*Milam Co*.: 9.vii.1983, B.F. & J.L. Carr, lot 4, (DAPC, 1).

*Sabine Co*.: 9 mi. E. Hemphill, “Beech Bottom”, 8–21.v.1989, R.Anderson & E. Morris, Malaise trap in beech-magnolia forest, (TAMU, 2).

*San Augustine Co*.: Piney Woods Conserv. Ctr., 14 mi. SE Broaddus, 15–21.vii.1993, E.G. Riley, Malaise trap, (TAMU, 1).

*Tyler Co*.: Kirby State Forest, 30°34'30"N, 94°25'03"W, 19.v-8.vi.2003, E.G. Riley, elevated FIT, (EGRC, 1).

*Wood Co*.: 5 mi. S. Hawkins at Jct Hwy 14 & FM 2869, 6.vii-18.viii.1996, W. Goodwin, FIT, (TAMU, 3; DAPC, 1).

**VIRGINIA**

*Norfolk Co*.: Norfolk, 23.vi.1978, J.M. Cumming, (DEBU, 1).

**WEST VIRGINIA**

*Hardy Co*.: Moorefield, 23.vii.1975, C.C. Coffman, Malaise trap, (DAPC, 1).

### *Eustrophopsis indistinctus* (LeConte)

**MEXICO**

**BAJA CALIFORNIA SUR**

10 mi. SW Canipole, 28.viii.1959, K.W. Radford & F.G. Werner, light trap, (UAIC, 2).

**CHIHUAHUA**

6 mi S. Villa Matamoros, 8.viii.1967, R.C. Gardner, C.R. Kovacic & K. Lorenzen, (AMNH, 1).

**UNITED STATES OF AMERICA**

**ARIZONA**

*Cochise Co*.: Portal, 5.viii.1972, S.I. and S.L. Frommer, (UCRC, 1); 5 mi. W. Portal, 2.iv.1970, J.D. Pinto, (UCRC, 1); Huachuca Mtns., Miller Canyon, 29.vii.1974, E.R. Hoebeke, (DAPC, 2); same locality, 8.vii.1974, E.R. Hoebeke, (DAPC, 1); same locality, 3.vii.1974, E.R. Hoebeke, (CUIC, 2); 9.viii.1974, E.R. Hoebeke, (CUIC, 1); Chiricahua Mts., Cave Cr. Ranch, 5000’, 6.viii.1965, G.W. Forister, (UCRC, 1); Guadalupe Cyn., el. 4300’, 1.vii.1976, P.M. Jump, (LACM, 1); Leslie Cany., 27.viii.1976, fungi, S. McCleve, (DAPC, 1; AMNH, 4); Cochise Stronghold, Dragoon Mts., 11.viii.1954, F.G. Werner, (UAIC, 1); Southwestern Research Station, nr. Portal, 7.viii.1977, Rosenberg, (USNM, 1); nr. Hereford, San Pedro R., 22.vii.1974, E.R. Hoebeke, (DAPC, 1); same data, 9.vii.1974, (CUIC, 1); 11.viii.1974, (CUIC, 2); Coronado Natl. For. Idlewild Campground vic. Portal, 7.viii.1972, Saul Frommer UV, (UCRC, 1); Guadalupe Cany., 15.vii.1978, S. McCleve, (AMNH, 1); 2.viii.1977, lite, S. McCleve, (AMNH, 1); Guadalupe Can., 1.vii.1976, at lite, S. McCleve, (UAIC, 4); same locality, 7.viii.1976, at lite, S. McCleve, (UAIC, 2); Guadalupe Can, 1286m, 6.vi.1978, at lite, S. McCleve, (UAIC, 1); S.W.R.S. [Southwestern Research Station], 5 mi. W. Portal, 5400 ft., M. Statham, 2.vii.1957, (AMNH, 3); 12.vii.1957, (AMNH, 1); 20.vii.1957, (AMNH, 2); 25–26.vii.1957, (AMNH, 3); 28.vii.1957, (AMNH, 1); 3.vii.1959, (AMNH, 1); 8.vii.1959, (AMNH, 1); 14.vii.1959, (AMNH, 2); 8.viii.1959, (AMNH, 1); 11.viii.1959, (AMNH, 1); same locality, E. Ordway, 22.vii.1956, (AMNH, 2); same locality, C. & M. Cazier, 9.vii.1956, (AMNH, 3); 18.vii.1956, (AMNH, 1); 16.vii.1956, (AMNH, 1); 5.viii.1956, (AMNH, 1); 16–17.vii.1960, (AMNH, 1); 20–21.vii.1960, (AMNH, 1); same locality, M.A. Cazier, (AMNH, 4); Chiricahua M[ts], 26.vii.1952, D.J. & J.N. Knull, (OSUC, 5); same locality and collectors, 22.vii.1957, (OSUC, 1); 12.vii.1953, (OSUC, 1); 15.vii.1953, (OSUC, 1); 20.vii.1953, (OSUC, 1); Chiricahua Mts., 27.vii.1957, D.J. & J.N. Knull, (OSUC, 4); near Portal, lite, 27.vi-7.vii.1977, J. Daneker, (AMNH, 1); Portal, 16.vii.1964, W.J. Gertsch and J.A. Woods, (AMNH, 1); same locality, 15.viii.1974, D.J. & J.N. Knull, (OSUC, 1); Huachuca Mts., 19.viii.1950, D.J. & J.N. Knull, (OSUC, 1); Palmerlee, vii, (CUIC, 1); Texas Pass, Dragoon Mts., 19–21.vii.1917, CU Biol. Exped. Lot 542, Sub 50, (CUIC, 2); Huachuca Mts., 7 mi. W. Palominas, Ash Canyon, 5100’, 21.viii.1995, Cate & Quinn, UV light, (TAMU, 1); Cave Creek Cyn., Chiricahua Mtns., 20.vii.1968, D.E. Bright, (CNC, 1); Huachuca Mts., Sierra Vista, v.1965, R.F. Sternitzky, (CNC, 1); same locality, vi.1966, R.F. Sternitzky, (CNC, 1); San Bernardino Ranch, SE Ariz., 5.vii.1975, S. McCleve, (UAIC, 6); same locality, 14.vii.1975, S. McCleve, at lite, (UAIC, 2); same locality, 9.viii.1976, fungi on *Salix*, McCleve, (UAIC, 1);

*Gila Co*.: Pinal Mts., Shooter Cn., 12–22.vii.1984, F.H. Parker, UV, (UAIC, 1); Sierra Ancha Mts, vii, D.K. Duncan, (UAIC, 1); Globe, 10.viii.1952, F.H. Parker, light, (UAIC, 1); same locality, 24.viii.1935, W. Parker, (UAIC, 1); same locality, D.K. Duncan, (CUIC, 1); Wheatfields near Globe, 11.iv.1926, D.K. Duncan, (CUIC, 1).

*Graham Co*.: Aravaipa Canyon, 3050’, 24.viii.1976, D.S. Chandler, uv light, (UAIC, 1; AMNH, 1); Aravaipa Cyn., 900 m., 24.vii.1976, J. M. Campbell, (CNC, 2); east end of Aravaipa Can, 24–25.vii.1974, at light, S. McCleve, (UAIC, 1); Aravaipa Can., 12.viii.1975, at lite, S. McCleve, (UAIC, 3); same locality, 21.vi.1976, at lite, S. McCleve, (UAIC, 2).

*Greenlee Co*.: Eagle Creek, 3550’, 15.ix.1976, along stream bank, D.S. Chandler, (UAIC, 1).

*Maricopa Co*.: Phoenix, 1.x.1939, G.F. Maughmer, (UCRC, 1); 11.ix.1940, Parker, on bracket fungus, (UAIC, 2); 15.v.1941, Parker, (UAIC, 1); 27.iii.1941, P.C. Grassman, (UAIC, 1); 10.viii.1942, P.C. Grassman, (UAIC, 12); 31.xii.1929, O.L. Barnes, cottonwood, (UAIC, 1); 31.xii.1929, O.L. Barnes, cottonwood, (CUIC, 1).

*Pima Co*.: Sta. Rita Exp. Range, 4.viii.1988, T.K. Philips, light, (MTEC, 1); Florida Canyon, 5.viii.1988, D.L. Gustafson, (MTEC, 1); mouth of Bear Cn., Sta. Catalina Mts., 3.vii.1961, l.t., Werner, Nutting, (UAIC, 1); Near Kits Peak, Baboquivari Mts., 7–9.viii.1916, 32°0’N, 111°36’W., about 3600 ft., (AMNH, 4); Baboquivari Mts, 1924, O.C. Poling, (CUIC, 3); Arivaca, 5 mi. W., 4.ix.1965, P.M. Jump, (LACM, 1); Catalina State Park, 27.vii.1983, at light, C.B. & J.E. Barr, (LSU, 1).

*Pinal Co*.: Picacho Peak St. Pk., 4.viii.1983, C.B. Barr, coll. at light, (LSU, 1); Copper Basin, 10.vi.1902, (CUIC, 1).

*Santa Cruz Co*.: Atasco Mts., el. 3975’, Sycamore Canyon, 12.vii.1977, P. Jump, (LACM, 1); Nogales, 7.viii.1952, D.J. & J.N. Knull, (OSUC, 2); Canelo, 29.ix.1943, J. Hendrickson, (UAIC, 1); same locality, 31.vii.1956, G.D. Butler, (UAIC, 1); Patagonia, 26.vii.1954, R. Schmitt, NJ light trap, (UAIC, 1); 1 mi. S. Patagonia, 29–30.vii.1964, D.R. Davis, (USNM, 1); Madera Canyon, 5100’, Bog Spring Cp. Grd. Santa Rita Mts, 10–26.vii.1964, D. Davis, (USNM, 2); Madera Canyon 4880’, Santa Rita Mts., J.C. Franclemont, 20.vii.1959, (CUIC, 1); 30.vi.1959, (CUIC, 2); 2.vii.1959, (CUIC, 1); 4.vii.1959, (CUIC, 2); Fraguita Wash, 4 mi. S. Arivaca, lite, 10.vii.1977, S. McCleve, (AMNH, 3); Nogales, D.J. & J.N. Knull, 8.viii.1950, (OSUC, 1); 2.viii.1955, (OSUC, 1); 4.viii.1953, (OSUC, 1); Pena Blanca Lake, 20.vii.1985, P.K. Lago, (PKLC, 2); Madera Cyn., 4880 ft. 10.vii.1963, V.L. Vesterby, (AMNH, 1); Madera Can., Sta. Rita Mtns., 13.viii.1968, E.C. Becker, (CNC, 1); Pajarito Mts., Sycamore Canyon, 24.viii.1968, (CNC, 1); 4 mi. W. Patagonia, 24.viii.1954, W.A. McDonald, (LACM, 2).

*Yavapai Co*.: Prescott, 16.viii.1939, F.H. Parker, (UAIC, 1); Kirkland, 26.viii-1.ix.1970, J.E. May, UV trap, (UAIC, 1).

**COLORADO**

*Bent Co*.: Hasty, 8.vi.1983, B.F. & J.L. Carr, lot 9, (CARR, 1); same locality, 13.vi.1982, B.F. & J.L. Carr, lot 2, (CARR, 2).

**NEW** **MEXICO**

*Bernalillo Co*.: Tijeras Cn., 3 mi. E. Albuquerque, 8–11.vii.1969, A. Smetana, (CNC, 1).

*Eddy Co*.: 31 km WNW Whites City, 32–15.31N, 104–41.84W, Sitting Bull Canyon, 1386 m, 17.vii.2000, R. Acciavatti, J. Glaser, uv light, dry, rocky creek bed near water in tree cover, (DAPC, 1).

*Roosevelt Co*.: Portales (173 Yucca Drive), 34°09'50.8"N, 103°20'33.6"W, uv light in backyard, 9.v.2006, D.A. Pollock, (DAPC, 1); Portales, 34°09'51.4"N, 103°20'32.8W, 7.vii.2002, Crowdy Pollock, coll. ex. fungusy birch stump, (DAPC, 1); Portales, ENMU campus, 12.ix.2009, ex fungus on dying willow (at night), D.A. Pollock, (DAPC, 1); “Melrose Woods", ca. 9 mi W. Melrose on Hwy 84, 34°25'59.8"N, 103°48'02.0"W, 18.vi.2005, on dead log at night, D.A. Pollock, (DAPC, 11); same locality and collector, 29.vi.2005, uv light, 29.vi.2005, (DAPC, 2); “Boone's Draw”, ca. 10 mi SW Portales, 34°06'56.8"N, 103°31'46.2"W, elev. 4129 ft., 26–27.vi.2009, coll. at night on fungusy poplar logs, D.A. Pollock, (DAPC, 23); same locality, 6.vi.2011, at night on charred, fungusy poplar snags, D.A. and G.W. Pollock, (DAPC, 12).

*Sierra Co*.: Arrey, 13.ix.1989, B.F. & J.L. Carr, lot 2, (CARR, 1).

*Locality unknown*: Cienega L. ca. 1.5 mi. N. Cienega Rch., 15.iv.1963, M.L. Noller, Coleman lt., (UAIC, 1).

**OKLAHOMA**

*Cimarron Co*.: Felt, 20.vi.1982, B.F. & J.L. Carr, (DAPC, 3; CARR, 17).

**TEXAS**

*Aransas Co*.: Lamar, 7.iv.1987, B.F. & J.L. Carr, lot 2, (CARR, 1).

*Blanco Co*.: Cyp[ress] Mill, Chittenden, (USNM, 1).

*Brewster Co*.: Big Bend N.P., Rio Grande Village, 29°10'50.5", 102°57'14.9", elev. 1840 ft., Pollocks, coll. at night on cut fungusy logs, (DAPC, 15); Big Bend Natl. Pk., North Rosillos Mts., Buttrill Spring, 10–17.vii.1991, R. Vogtsberger, Malaise trap, (TAMU, 6).

*Cameron Co*.: Brownsville, iv, (CUIC, 1).

*Harris Co*.: Cypress Mills [suburb of Houston?], 15.v, (CUIC, 1).

*Hidalgo Co*.: Mission, Bentsen State Park, 18.vii.1981, W.E. Steiner, under bark of Celtis laevigata, (USNM, 2).

*State record only*: (AMNH, 2).

### *Eustrophopsis arizonensis* (Horn)

**MEXICO**

**DURANGO**

1.6 mi W. Tepalcates Rd, Meadow, 6.viii.1972, D. Veirs, J. Powell, C.D. MacNeill, under bark, (UCBC, 2); 25 mi. W. Durango, 29.vi.1964, H.F. Howden, (CNC, 2); 10 mi. W. El Salto, 9000’, 20.vi.1964, J.E.H. Martin, (CNC, 1).

**MORELOS**

Cuernavaca, 6 mi. N., 31.vii.1963, J. Doyen, (DAPC, 1; CNC, 2); same locality, 31.vii.1963, J. Doyen, (UCBC, 4); Morelos, 9 mi S. Juchiltepec, 29.vi.1982, M.A. Ivie, under pine bark, (MTEC, 15); same locality, 1500–2000 ft., 29.vi.1982, M.A. Ivie, (MTEC, 5); 8 mi. S. Juchiltepec, 29.vi.1982, R.S. Miller, (MTEC, 10); same locality, 29.vi.1982, M.A. Ivie, (MTEC, 4).

**OAXACA**

26 km S Yolomecatl, 2385 m., 10.viii.1988, Doyen & Stockwell, (UCBC, 1); Sierra de Manantlan, Jalisco 1800–1900 m, 18.vii.1985, J. Doyen, bosque mesofilo, (UCBC, 2); Sierra de Manantlan, Jalisco Las Joyas, 1870 m, 18.vii.1985, J. Doyen, under bark, (UCBC, 1); Sierra de Manantlan, Jalisco, 1–2 km N. Las Joyas, 1600–1700m, 19.vii.1985, J. Doyen, under bark, *Quercus*, (UCBC, 1).

**SONORA**

36 km NE Nacori Chico, 1840 m, 7.viii.1982, S. McCleve, G.E. & K.E. Ball, (SMC, 2).

**UNITED STATES OF AMERICA**.

**ARIZONA**

no other data, (CNC, 4); no other data, (MCZ, 3); no other data, Chas Palm, (AMNH, 2); no other data, L.B. Woodruff, (AMNH, 1).

*Apache Co*.: Ganado, 30.vii.1962, S. McCleve, (SMC, 1); Greer, 8000’, 5.vii.1953, A. & H. Dietrich, (CUIC, 3).

*Cochise Co*.: (CUIC, 3); 5 mi SSW Portal, 4.viii.1961, J.F. Lawrence, (MCZ, 1); 5 mi. W. Portal, 25.vi.1958, W.F. Barr, (WFBM, 2); AMS WRS, 5 mi. W. Portal 5400 ft., 2.viii.1969, R.L. Wenzel, (FMNH, 1); same locality, 26.vii.1969, R.L. Wenzel, (FMNH, 1); Ash Canyon, 24.vii.1968, D.E. Bright, (CNC, 2); Cave Cr. Ranch, 5000’, 6.viii.1965, K.W. Brown, uv light, (TAMU, 1); Cave Creek Ranch, Chiricahua Mtns., 5000’, 9.viii.1965, G.W. Forister, (UCRC, 1); Chiricahua Mts., 4–8.vii.1973, M.A. Goodrich & W.W. Boyle, (EIUC, 1); Chiricahua Mts. South Fork Canyon, 30.vi.1965, R.H. Arnett, Jr., blacklight trap, (FSCA, 1); Chiricahua Mts., Barfoot Park, 2535 m, 24.vi.1980, S. McCleve, under pine bark, (SMC, 1); Chiricahua Mts., Cave Creek Cyn., 1–5.viii.1996, M.A. Goodrich, malaise trap, (DAPC, 1); same locality, 1–5.viii.1996, M.A. Goodrich, malaise trap, (EIUC, 2); Chiricahua Mts., Coronado Natl. Forest, Sunny Flat, 26.vii.1983, C.B. Barr, at blacklight, (LSU, 1); Chiricahua Mts., E. Turkey Creek, 2633 m, 22.vi.1978, S. McCleve, at light, (SMC, 1); Chiricahua Mts., Herb Martyr Tr., 10.viii.1993, S. O’keefe & M. Caterino, (UCBC, 3); Chiricahua Mts., Horseshoe Cn, 1511 m, 27.vi.1978, S. McCleve, at light, (SMC, 1); same locality, 27.vi.1978, S. McCleve, lite, (AMNH, 1); Chiricahua Mts., N. Fk.Rucker Cn. 1908 M., 1.vii.1978, S. McCleve, lite, (AMNH, 1); Chiricahua Mts., South Fork Canyon, 20–21.viii.1970, R.E. Woodruff, (FSCA, 1); Cottonwood Cn, 33 mi. E. Douglas, 28.vii.1976, S. McCleve, at light, (SMC, 3); Guadalupe Canyon, 26.vi.1977, P. M. Jump, (LACM, 1); same locality, 15.vii.1978, Scott McCleve, (AMNH, 1); same locality, 15.vii.1978, Scott McCleve, (AMNH, 1); Horseshoe Cn. 4600’, 30.vii.1976, McCleve & Topham, lite, (AMNH, 1); Huachuca Mts. Carr Cyn., Clark Spr., el. 6150’, 17.vii.1976, P.M. Jump, (LACM, 2); Huachuca Mts. Copper Cn., 5.vii.1977, McCleve & Topham, lite, (AMNH, 2); Huachuca Mts., Miller Canyon, 7.vii.1974, E.R. Hoebeke, (CUIC, 4); same locality, 30.vii.1974, E.R. Hoebeke, (CUIC, 2); same locality, 8.vii.1974, E.R. Hoebeke, (CUIC, 2); same locality, 23.vii.1974, E.R. Hoebeke, (CUIC, 2); same locality, 10.vii.1974, E.R. Hoebeke, (CUIC, 1); same locality, 16.viii.1974, E.R. Hoebeke, (CUIC, 1); same locality, 21.vii.1974, E.R. Hoebeke, blacklight, (CUIC, 1); same locality, 8.viii.1974, E.R. Hoebeke, (CUIC, 1); same locality, 5.vii.1974, E.R. Hoebeke, (CUIC, 1); Huachuca Mts., Miller Canyon, 5000’, 23.vii.1974, T.L. McCabe, (CUIC, 2); same locality, 7.vii.1974, T.L. McCabe, (CUIC, 3); same locality, 2.vii.1974, T.L. McCabe, (CUIC, 1); same locality, 18.vii.1974, T.L. McCabe, (CUIC, 1); same locality, 8.vii.1974, T.L. McCabe, (CUIC, 2); same locality, 27.vii.1974, T.L. McCabe, (CUIC, 1); same locality, 4.vii.1974, T.L. McCabe, (CUIC, 1); same locality, 10.vii.1974, T.L. McCabe, (CUIC, 1); same locality, 30.vii.1974, T.L. McCabe, (CUIC, 1); same locality, 17.vii.1974, T.L. McCabe, (CUIC, 2); Huachuca Mts., Miller Cn, 4.viii.1976, S. McCleve, at light, (SMC, 3); Huachuca Mts., Miller Cyn, ca. 6000’, 28.vii.1989, W.B. Warner, at light, (RLC, 1); Huachuca Mts., Ramsey Cyn, viii-ix.1968, R.F. Sternitsky, (CNC, 2); Miller Cyn., 4.vii.1973, (TAMU, 1); Portal, 26.viii.1957, W.F. Barr, (WFBM, 1); Portal, Southwest R.S., 13.vii.1985, Bush Faculty Dev. Grant, (NDSU, 1); Rustler Park, 8 mi. W. Portal, 3.viii.1961, J.F. Lawrence, (MCZ, 1); S. Fork Canyon, 10.viii.1959, R.H. Arnett, Jr., lot 444, (FSCA, 1); Southwest Res. Sta, 6.viii.1973, L.L. Lampert, uvl, (DAPC, 2); same locality, 7.viii.1973, L.L. Lampert, (DAPC, 1); same locality, 15.viii.1973, L.L. Lampert, uvl, (DAPC, 1); same locaity, 8.viii.1973, L.L. Lampert, uvl, (FSCA, 1); same locality, 12–17.viii.1976, L.L. Lampert, Jr., uv light, (FSCA, 1); Southwest Research Station, Chiricahua Mts., 2.viii.1967, D.J. Culver, at blacklight, (UCRC, 1); same locality, 26.viii.1967, E.I. Schlinger, (UCRC, 1); Southwestern Research Station Nr. Portal, 6.viii.1973, Rosenburg collection, (USNM, 1); same locality, 27.viii.1977, Rosenburg collection, (USNM, 1); same locality, 25.viii.1973, Rosenburg collection, (USNM, 1) same locality, 24.viii.1977, Rosenburg collection, (USNM, 1); Stewart Camp, 1 mi. S. Portal, 18–20.vii.1971, J.T. Doyen, light trap, (UCBC, 1); SW Res. Sta, Portal, 2.vii.1956, H. & A. Howden, to light, (CMNC, 1; CNC, 1); same locality, 15.vi.1956, H. & A. Howden, light, (CMNC, 1); same locality, 30.vi.1956, H. & A. Howden, light, (CMNC, 1); same locality, 24.vi.1956, H. & A. Howden, light, (CMNC, 2); same locality, 9.vii.1956, H. & A. Howden, (CMNC, 1); SW Research Sta. 5 mi. W Portal, 20.viii.1955, Gertsch & Ordway, (AMNH, 1); SW Research Station of AMNH, 5 mi. W Portal 5400’, 15.viii.1955, W.J. Gertsch, (AMNH, 1); same locality, 19.vii.1955, W.J. Gertsch, (AMNH, 2); same locality, 18.vii.1955, W.J. Gertsch, (AMNH, 2); same locality, 19.vii.1971, (FSCA, 1); SWRS, 5 mi. W. Portal, 5400’, 16.vii.1956, C&M Cazier, (AMNH, 1); same locality, 9.vii.1956, E. Ordway, (AMNH, 1); same locality, 9.vii.1956, C&M Cazier, (AMNH, 2); same locality, 5400’, 20.viii.1956, E. Ordway, (AMNH, 1); same locality, viii.1956, A. Archer, (AMNH, 1); same locality, 19.vii.1956, M. Cazier, (AMNH, 1); same locality, 2.vii.1956, F.A. Blagdea, (AMNH, 4); same locality, 2.vii.1956, C&M Cazier, (AMNH, 1); same locality, 8.vii.1959, M. Statham, (AMNH, 1); same locality, 12.vii.1957, M. Statham, (AMNH, 1); same locality, 19.vii.1957, M. Statham, (AMNH, 1); same locality, 8.vii.1955, E. Ordway, (AMNH, 1); same locality, 5.vii.1956, E. Ordway, (AMNH, 1); same locality, 10.vii.1956, C&M Cazier, (AMNH, 1); same locality, 12.vii.1956, E. Ordway, (AMNH, 2); same locality, 10.vii.1959, M. Statham, (AMNH, 1); same locality, 20–21.vii.1960, C&M Cazier, (AMNH, 1); same locality, 8.vii.1963, J.G. Rozen, D.K. Oliver, A.R. Moldenke, J.A. Woods , (AMNH, 2); same locality, 13.vi.1957, M. Statham, (AMNH, 1); same locality, 7.vii.1956, M. Cazier, (AMNH, 1); same locality, 20–21.vii.1960, C&M Cazier, (AMNH, 1); same locality, 5.vii.1959, C&M Cazier, (AMNH, 1); same locality, 7.vii.1956, C&M Cazier, (AMNH, 1); same locality, 4.vii.1956, Blagden & Eaton, (AMNH, 1); same locality, 6.vii.1956, E. Ordway, (AMNH, 1); same locality, 11.vii.1958, M. Cazier, (AMNH, 1); same locality, 5400’, 2.viii.1961, J.G. Rozen, (AMNH, 1); same locality, 22.vi.1955, M. Statham, (AMNH, 1); same locality, 28.vii.1958, M. Cazier, (AMNH, 1); same locality, 22.vii.1957, M. Statham, (AMNH, 1); same locality, 8.vii.1963, J.G. Rozen, D.K. Oliver, A.R. Moldenke, J.A. Woods , (AMNH, 1); same locality, 15.vii.1956, C&M Cazier, (AMNH, 1); same locality, 1.viii.1956, E. Ordway, (AMNH, 1); same locality, 22.vii.1956, E. Ordway, (AMNH, 1); same locality, 8.viii.1956, E. Ordway, (AMNH, 1); W. Turkey Cr., 16.vii.1976, McCleve & Topham, lite, (AMNH, 1); Whetstone Mts., Cottonwood Can., 1670 m, 7.viii.1978, S. McCleve, at light, (SMC, 2); same locality, 7.viii.1978, S. McCleve, lite, (AMNH, 1); 5 mi. W. Portal; SWRS 5400’, 10.viii.1988, R.S. Miller & family, (MTEC, 1); Portal, 11.viii.1988, R.S. Miller & family, at light, (MTEC, 1). Chiricahua M., 9.vii.1959, D.J. & J.N. Knull, (OSUC, 6); same locality18.vii.1961, D.J. & J.N. Knull, (OSUC, 2); same locality, 16.vii.1959, D.J. & J.N. Knull, (OSUC, 5); same locality, 16.vii.1929, D.J. & J.N. Knull, (OSUC, 2); same locality, 9.vii.1929, D.J. & J.N. Knull, (OSUC, 2); same locality, 20.vii.1955, D.J. & J.N. Knull, (OSUC, 2); same locality, 28.viii.1962, D.J. & J.N. Knull, (OSUC, 1); same locality, 30.vii.1959, D.J. & J.N. Knull, (OSUC, 2); same locality, 25.vii.1961, D.J. & J.N. Knull, (OSUC, 1); same locality, 24.vii.1955, D.J. & J.N. Knull, (OSUC, 1); Chiricahua M., SW Res. Sta., 28.viii.1960, C.A. Triplehorn, (OSUC, 1); Chiricahua Mtns., Pinery Cyn., 7000’, 9.vii.1956, H. & A. Howden, at light, (CNC, 1); Pinery Canyon Campground, 7000 ft., 31°55'58", 109°16'23.8", 13.vii.2005, at u-v light, D.A., G.W. and H.J. Pollock, (DAPC, 1); Chiricahua Mts. Stewart Cmp. Gr., 13.vii.1963, G.H. Nelson, (AMNH, 1); Chiricahua Mts., Herb Martyr Dam, 6200’, 14–15.viii.1962, F. Werner, Coleman lt. trap, (UAIC, 1); Chiricahua Mts., Tex Cn, 5300’, 12.viii.1974, S. McCleve, at light, (SMC, 1); Cochise Stnghld., Dragoon Mts., 16–17.viii.1962, F. Werner, coleman lt. trap, (UAIC, 1); Guadalupe Cn., 31.vii.1975, S. McCleve, at light, (SMC, 1); nr. Portal, SW Research Station, 14–22.vii.1957, R.H. Arnett, lot 289, (FSCA, 2); Peloncillo Mtns., 33 mi E. Douglas, 17.vii.1973, S. McCleve, at light, (SMC, 1); Pinery Canyon, Chiracahua Mts. 7000’, 9.vii.1956, H. & A. Howden, at light, (CMNC, 3); Portal, 5.viii.1965, B.K. Dozier, (FSCA, 1); S.W. Res. Stn., 5 mi. W. Portal, 7–14.ix.1960, H. Howden, (CNC, 1); South fork Cave Creek 5 Mi. W. Portal 5000–5500ft., 11.ix.1959, W. Gertsch, M. Cazier, (AMNH, 1); SW Res. Sta., elev. 5–6000 ft., 2.ix.1958, H.V. Weems, Jr., at light, (FSCA, 1); SW Res. Sta., elev. 5–6000 ft., 7.ix.1958, H.V. Weems, Jr., at light, (FSCA, 1); SW Res. Stat., Portal, 5400 ft., 2.viii.1955, R.R. Dreisbach, (MSUC, 1); SW Res. Stn., 5400 ft., 31.viii.1959, C.P. Alexander, (MCZ, 1); Texas Pass, 19.vii.1917, (CUIC, 1); Texas Pass, Dragoon Mts., 21.vii.1917, C.U. Biol. Exped. lot 542, Sub 50, (CUIC, 1); Ft. Huachuca, Graveyard Gulch, 16–18.viii.1995, Cate & Quinn, elev. 1284 m., (TAMU, 1).

*Coconino Co*.: Slate Mtn. Loop Rd 6900’, 20 mi NW Flagstaff, 15.vii.1965, R.W. Poole, (CUIC, 1); Walnut Canyon 6500’, 6 1/3 mi ESE Flagstaff, 28.viii.1965, J.G. Franclemong, (CUIC, 1). Flagstaff, 1–3.vii.1953, B. Malkin & J. Farmer, (FMNH, 1); Grand Canyon, North Rim 8000–9100 ft, 17.vii.1931, D. Rockefeller, (AMNH, 1); Mormon Lake, 7000’, 8–15.vii.1956, F. Werner, at light, (DAPC, 1); same locality, 15.vii.1956, F. Werner, light, (UAIC, 2); Williams, (CUIC, 2); same locality, L.B. Woodruff, (AMNH, 1); 1.5 mi. S. Hwy 40 & 3.8 mi W. Flagstaff (off FSR 151), 35.1795, -111.7553, 17–19.vii.2007; coll. at night on dead pine logs, D. and G. Pollock, (DAPC, 38); Grand Canyon, Havasupai Cmpgrnd, 17–19.x.2003, ex polypore fungus, Brown & Mathison, (DAPC, 1).

*Gila Co*.: Pinal Mts., 4.viii.1939, Parker, (UAIC, 1).

*Graham Co*.: Swift Trail, 7400’, Pinaleño Mts., 6.vii.1973, T.P. Sluss & D.S. Chandler, (OSUC, 2).

*Maricopa Co*.: Mazatzal Mnt., Bushnell Tanks, 4.x.2003, under Populus bark, Harris & Mathison, (DAPC, 1); same locality, 9.v.2004, under Populus bark, Harris & Mathison, (DAPC, 2); same locality, 26.ix.2004, fungus on dead Populus, Harris & Mathison, (DAPC, 2).

*Navajo Co*.: Pinetop, 7.3.1950, E.C. Johnston, (CNC, 1); Tonto Nat’l Forest, Upper Tonto Cr. campground, 24.v.1987, R. Leschen, under bark, (RLC, 1).

*Pima Co*.: Bear Canyon, Santa Catalina Mtns., 15.viii.1968, D.E. Bright, (CNC, 1); Catalina Mts., Htchk. Hwy. Mi. 5–6, 14.vii.1955, G.D. Butler & F.G. Werner, at light, (UAIC, 3); Mt. Lemmon, Tucson, 17.vii.1970, C.A. Triplehorn, (OSUC, 3); Sta. Catalina Mts., Hk. Hwy. Mi. 10, 12.xi.1956, R. Simpson, (UAIC, 1); Sta. Catalina Mts., Mt. Lemmon, 12.vii.1970, C.A. Triplehorn, (OSUC, 2); Pinal Mts., Pinal Pk., 2304 m, 27.vi.1979, S. McCleve, at light, (SMC, 1); Sierra Ancha Exp. Sta., 27.iv.1958, W.E. Currie, (FSCA, 1); Sierra Ancha Mts., Workman Ck, 6000’, 4.viii.1977, S. McCleve, at light, (SMC, 2); Tonto Creek, 15 mi. E. Payson, 1640 m, 26.vii.1976, J. M. Campbell, (CNC, 2); Bear Canyon, 6.vii.1959, R.H. Arnett, Jr., lot 395, (FSCA, 6); same locality, 11.vii.1959, R.H. Arnett, Jr., lot 408, (FSCA, 1); same locality, 2.vii.1959, R.H. Arnett, Jr., lot 387, (FSCA, 2); Coronado Nat. Forest, Madera Canyon, Bog Springs Campground, 17.viii.1989, D.A. Pollock, (DAPC, 1); Kitt Peak, 2.viii.1968, D.E. Bright, (CNC, 1); Madera Canyon, 31.viii.1977, C.Y. Kitayama, (UCBC, 1); Madera Cyn., 11.vii.1973, (TAMU, 1); Sta. Catalina Mts., Bear Cn, 1828 m, 16.vi.1980, S. McCleve, at light, (SMC, 1); Sta. Rita Mts., Madera Cyn. 5100’, 6.ix.1952, G.M. Bradt, (AMNH, 1); same locality, 13.viii.1952, M. Cazier, R. Schrammel, C. & P. Vaurie, (AMNH, 3); Bogs Springs, 12.viii.1978, M.A. Ivie, (MTEC, 1); Madera Canyon , 8.viii.1986, R.S. Miller, (MTEC, 1).

*Pinal Co*.: 18.viii.1940, F.W. Nunenmacher, (FMNH, 2).

*Santa Cruz Co*.: Canelo, 31.vii.1956, G.D. Butler, (UAIC, 3); Canelo, 10.vii.1957, G.D. Butler, (UAIC, 1); same locality, 8.viii.1956, Morley, at light, (UAIC, 1); Nogales, 2.viii.1955, D.J. & J.N. Knull, (OSUC, 1); Santa Rita Mountains, 5–8,000 ft., vii, F.H. Snow, (UMRM, 2); Santa Rita Mts., (CUIC, 2); same locality, (MCZ, 1); Santa Rita Mts., Madera Canyon, 4600–5600’, 29.vii.1978, M. Hatz, (DAPC, 1); Santa Rita Mts., Madera Cyn, 5500’, 3.viii.1969, A. Smetana, at light, (CNC, 1); Sycamore Canyon, nr. Ruby, 16–17.viii.1961, Werner, Bequaert, (UAIC, 1); same locality, 16–17.viii.1961, Werner, Bequaert, (UAIC, 1); Madera Canyon 5400’, 3.viii.1988, D.L. Gustafson, (MTEC, 1); same locality, 3.viii.1988, M.A. Ivie, (MTEC, 1); 3.2 km S. Pena Blanca Lake, 1250 m., 28.vii.1989, R.S. Anderson & S. McCleve, uv light, (TAMU, 1); Atasco Mts., el. 3975’, Sycamore Canyon, 12.vii.1977, P. Jump, (LACM, 2); Atascosa Mts., Sycamore Cn, 12.vii.1977, S. McCleve, ex fungi, (SMC, 3); Coronado N.F., Santa Rita Mtns., Madera Cyn, 12–13.viii.1987, C.B. & J.E. Barr, mv/bl, (CBC, 1); same locality, 12–13.viii.1987, C.B. & J.E. Barr, mv/bl, (LSU, 1); same locality, 28.vii.1992, D.R. Ganaway, mv/bl, (LSU, 2); Coronado Nat. Forest, Madera Canyon Rec Area, Santa Rita Lodge, 14.viii.1989, D.A. Pollock, uv light, (DAPC, 1); Hwy 289, Pena Blanca to Sycamore Canyon, 6.viii.1993, O’Keefe & Caterino, blacklight, (UCBC, 1); Madera Canyon, 25–28.vii.1982, N.M. Downie, (FMNH, 2); same locality, 31.vii.1965, G. Ballmer & K. Brown, uv light, (UCRC, 1); same locality, 26.vii.1972, B.C. Ratcliffe, (UNSM, 1); Madera Canyon, 4880’, 10.viii.1965, D.N. Harrington, (DAPC, 1); Madera Canyon, 4880’, 2.vii.1965, D.N. Harrington, (DAPC, 1); Madera Canyon, Sta. Rita Mtns., 4880’, 29.vi.1963, J.D. Marshall, (CUIC, 1); Madera Canyon, Sta. Rita Mts., 5600 ft., 6.vii.1963, J.D. Marshall, (CUIC, 1); Madera Cyn., 5400’, 18–29.vi.1979, F.T. Hovore, (PKLC, 1); Nogales, 12.viii.1906, F.W. Nunenmacher, (FMNH, 1); Pajarito Mts., Peña Blanca Cn., 1158 m, 30.vii.1979, S. McCleve, (SMC, 1); Pajarito Mts., Pena Blanca Cn., 1191 m, 27.vii.1978, S. McCleve, lite, (AMNH, 1); Pajarito Mts., Sycamore Canyon, 24.viii.1968, (CNC, 1); Pajarito Mts., Walker Cn, 1191 m, 28.vii.1978, S. McCleve, dead oak at night, (SMC, 3); same locality, 28.vii.1978, Scott McCleve, dead oak at night, (AMNH, 7); Pena Blanca, 21.vii.1959, R.H. Arnett, Jr., lot 421, at light, (FSCA, 1); Peña Blanca, 3900’, 5.viii.1979, J.K. Liebherr, at light, (UCBC, 1); Pena Blanca, Pajarito Mts., 30.vii.1961, Arnett & Van Tassell, lot 534, (FSCA, 3); same locality, 31.vii.1961, Arnett & Van Tassell, lot 536, (FSCA, 3); same locality, 6.viii.1961, Arnett & Van Tassell, lot 560, (FSCA, 1); same locality, 19.vii.1962, Arnett & Van Tassell, lot 600, (FSCA, 1); same locality, 24.vii.1963, Arnett & Van Tassell, lot 763, (FSCA, 1); same locality, 9.viii.1961, Arnett & Van Tassell, lot 568, (FSCA, 1); same locality, 24.vii.1961, Arnett & Van Tassell, lot 504, (FSCA, 1); same locality, 5.viii.1961, Arnett & Van Tassell, lot 557, (FSCA, 1); same locality, 26.vii.1961, Arnett & Van Tassell, lot 511, (FSCA, 2); same locality, 1.viii.1961, Arnett & Van Tassell, lot 540, (FSCA, 1); Pence Blanca Canyon, 27–28.vii.1964, D.R. Davis, (USNM, 2); Santa Rita Mts., Madera Cyn, 20.vii.1990, W.B. Warner, at light, (RLC, 1).

*Yavapai Co*.: Prescott, (CUIC, 1); same locality, vi-vii.1894, (MCZ, 2); same locality, 27.viii.1968, J. & J. McCleve, at light, (SMC, 1); same locality, 26.viii.1968, S. McCleve, at light, (SMC, 1); Granite Basin Lake, 7 mi. NW Prescott, 17.vii.1985, P.K. Lago, (PKLC, 2); Prescott, 22.vi.1959, A. Raske, under log, (CNC, 2); same locality, 30.vi.1959, A. Raske, under log, (CNC, 2 ).

**CALIFORNIA**

*Riverside Co*.: near Lake Hemet, 3.iii.1968, S. McCleve, pine stump, (SMC, 1).

*San Bernardino Co*.: Forest Home, Mill Creek Cn., 31.iii.1963, J.C. Ball, (UCRC, 1); 34.1609°N, 116.9179°W, San Bernardino NF, W. of Barton Flats, 28.v.2004, M. Caterino, CA Beetle Proj #CBP0020675, (SBMNH, 1); 34.3017°N, 117.1796°W, SBNF: Willow Ck., 16.v.2006, Caterino & Chatzimanolis, CA Beetle Proj #CBP0046889, (SBMNH, 1); 34.1819°N, 116.8884°W, San Bernardino NF: Sta. Ana R., 14.vii.2006, Caterino & Chatzimanolis, CA Beetle Proj #CBP0054418, (SBMNH, 1).

**COLORADO**

*Douglas Co*.: 7–6 1915, W.D. Edmonston, (CUIC, 2).

*La Plata Co*.: vic. of Durango, 23.vii-8.viii.1885, (MCZ, 5).

**MONTANA**

*Powder River Co*.: Camps Pass, 4.viii.1991, D.L. Gustafson, blt, (MTEC, 1).

**NEW** **MEXICO**

Ulke collection, (CMNH, 2); F.H. Snow, (CUIC, 1); no other data (MCZ, 1).

*Catron Co*.: CCH, (AMNH, 1).

*Cibola Co*.: 13.5 mi. S. Grants, 17.vii.1986, C.A. Triplehorn, (OSUC, 1).

*Grant Co*., McMillaan Camp 13 mi. N. Silver City 6800’, 24.vii.1961, F., P. &J. Rindge, (AMNH, 1); Pinos Altos, 4.vii.1975, K. Stephan, (FSCA, 1); same locality, 16.viii.2005, B. Baldwin, UV light, (BBC, 1).

*Hidalgo Co*.: Animas Mts., Indian Creek, 1737 m, 5–6.viii.1979, S. McCleve, at light, (SMC, 2); Clanton Cyn. 5600 ft., 14.vii.1960, K. John, (AMNH, 1); nr. Rodeo, cienega lake, 28.vii.1965, R.H. Arnett, Jr., (FSCA, 1); Rodeo 2 mi. S, 30.viii.1973, W. Rosenberg, (USNM, 1).

*Lincoln Co*.: Eagle Crk. CG, near Ruidoso, 33°23'31.7", 105°43'30.7", 28.v.2003, 7680 ft., D.A. Pollock, (DAPC, 1).

*Los Alamos Co*.: Los Alamos, 11.viii.1963, J. Doyen, (DAPC, 1); same locality, 11.viii.1963, J. Doyen, (UCBC, 2). *McKinley Co*., Ft. Wingate, 8.viii.1990, Carrs, lot 1, (CARR, 1).

*San Miguel Co*., Las Vegas, vii.1900, (CMNH, 1).

*Torrance Co*., Clayton Hoff, (AMNH, 1).

**SOUTH** **DAKOTA**

*Fall River Co*.: Angostura Dam, S. of Hot Springs, 9.vii.1968, A.T. Howden,(CNC, 2).

**TEXAS**

*Brewster Co*.: Alpine, 15–30.v.1926, O.C. Poling, (CUIC, 1).

*Jeff Davis Co*.: Davis Mountains 6700’ Mount Locke, 4.vii.1969, A. & ME Blanchard, (USNM, 6); Fort Davis, 31.v.1959, Howden & Becker, fungus, (CNC, 1); same locality, 1.vi.1959, Howden & Becker, fungus, (CNC, 1); 2 mi. N. Fort Davis, 7.viii.1963, J. Doyen, (UCBC, 1); Davis Mts. Resort, 24.vii.1989, E. Riley & C. Wolfe, (EGRC, 2); Davis Mts. Resort, D. Marqua residence, 5800 ft., 28.vi-5.vii.1993, D. Marqua, Malaise trap, (TAMU, 1); Davis Mts. St. Pk., 12.vii.1961, Arnett & Van Tassell, lot 483, (DAPC, 1); same locality, 12.vii.1961, Arnett & Van Tassell, lot 483, (FSCA, 10); same locality, 15.viii.1982, R. Turnbow, blacklight, (TAMU, 1); Davis Mts. Resort (Marqua residence), 5,800 ft., 30.62842°N, 104.08360°W, 4–5.vii.2009, E. & M.L. Riley, (EGRC, 3); Ft. Davis, Lympia Canyon, 4–6.vii.1978, J.E. Wappes, (TAMU, 1).

**UTAH**

*Washington Co*.: Zion Park, 1.viii.1929, C.C. Searl, (LACM, 2; UMMZ, 2).

### *Eustrophopsis ornatus* (Van Dyke)

**MEXICO**

**JALISCO**

15 km S. Mazamitla, 5500’, 29–31.vii.1952, F.W. & F.G. Werner, (UAIC, 1)

**SONORA**

6 mi. S. Yecora, Son. 5740’, 2–3.vii.1990, S. McCleve, u-v light, (DAPC, 2); creek W. Maycoba (@ 3 mi W.), 21.viii.1986, Baumann, Sargent & Kondratieff, (BYUC, 1).

**UNITED STATES OF AMERICA**

**ARIZONA**

*Cochise Co*.: 5 mi. W. Portal; SWRS 5400’ , 23.vii.1988, R.S. Miller & family, (MTEC, 1); same locality, 10.viii.1988, R.S. Miller & family, (MTEC, 1); same locality, 5.viii.1988, R.S. Miller & family, (MTEC, 1). Huachuca Mts. Copper Canyon 1882 M., 24.vii.1978, S. McCleve, lite, (AMNH, 1); Chiricahua Mts. 6400 ‘, Onion Saddle Rd. at East Turkey Creek, 3.viii.1967, R.C. Beard, at uv light, (CUIC, 1); Chiricahua Mts., Horseshoe Cn. 6000’, 4.vii.1977, S. McCleve, at light, (SMC, 1); Chiricahua Mts., N. Fork Rucker Cn, 1908 m, 1.vii.1978, S. McCleve, at light, (SMC, 4); same locality, 1.vii.1978, S. McCleve, lite, (AMNH, 3); Chiricahua Mts., South Fork Canyon, 30.vii.1965, R.H. Arnett, Jr., blacklight trap, (FSCA, 1); same locality, 16.vii.1965, R.H. Arnett, Jr., blacklight trap, (FSCA, 1); same locality, 20–21.viii.1970, R.E. Woodruff, (FSCA, 2); same locality, 16.vii.1964, Arnett & Van Tassell, lot 832, (FSCA, 1); Coronado Nat. For., Pinery Canyon, 5 mi. E. Hwy 181 on Forest Service Rd. 42, 6.viii.1989, D.A. Pollock, under dead pine bark, (DAPC, 1); Dragoon Mts., Sorin Cn 1926 m, 23–24.vii.1979, S. McCleve, at light, (SMC, 1); Huachuca Mts., 5354 Ash Cyn. Rd., 0.5 mi w Hwy 92 E1 5100ft, 21.viii.1992, N. McFarland, (USNM, 1); Huachuca Mts., Carr Canyon, 10,000 ft., 23.viii.1970, R.E. Woodruff, coll. at night, (FSCA, 1); Huachuca Mts., Copper Cn., 5.vii.1977, S. McCleve, at light, (SMC, 2); same locality, 5.vii.1977, McCleve & Topham, lite, (AMNH, 6); Huachuca Mts., Miller Canyon, 21.vii.1974, E.R. Hoebeke, blacklight, (CUIC, 1); Leslie Cyn., 12 mi. E. McNeal, 4500’, 25.viii.1981, J. Liebherr, (UCBC, 1); Miller Canyon, 28.viii.1983, A. Strong, (UCRC, 2); nr. Portal, SW Research Station, 14–22.1957, R.H. Arnett, lot 289, (DAPC, 1); Palmerlee, 17.viii, (CUIC, 2); Portal, 21.viii.1973, L.L. Lampert, I.L., (FSCA, 1); same locality, 2.viii.1965, B.K. Dozier, (FSCA, 1); Southwest Res. Sta., 3.viii.1973, L.L. Lampert, uvl, (FSCA, 1); same locality, 7.viii.1973, L.L. Lampert, uvl, (FSCA, 1); same locality, 8.viii.1973, L.L. Lampert, uvl, (FSCA, 1); Southwest Research Station, Chiricahua Mts., 31.viii.1966, G.R. Ballmer, (UCRC, 1); same locality, 21.viii.1970, L.D. and M. Anderson, (UCRC, 1); Southwestern Research Station Nr. Portal, 27.viii.1973, Rosenberg collection, (USNM, 2); same locality, 19.viii.1973, Rosenberg collection, (USNM, 1); same locality, 23.viii.1973, Rosenberg collection, (USNM, 1); Stewart Camp, 1 mi. S. Portal, 18–20.vii.1971, J.T. Doyen, blacklight, (UCBC, 1); SWRS 5 mi. W Portal 5400 ft., 24.vi.1956, E. Ordway, (AMNH, 1); same locality, 19.vii.1955, M. Cazier, (AMNH, 1); same locality, 1.vii.1959, M. Cazier, (AMNH, 2); same locality, 5.vii.1963, J.G. Rozen, D.K. Oliver, A.R. Moldenke, J.A. Woods, (AMNH, 1); same locality, 26.vii.1957, M. Statham, (AMNH, 1); same locality, 5.vii.1956, E. Ordway, (AMNH, 1); same locality, 4.viii.1956, C&M Cazier, (AMNH, 1); same locality, 16.vii.1956, C&M Cazier, (AMNH, 1); same locality, 11–14.vii.1960, C&M Cazier, (AMNH, 1); same locality, 27.vii.1956, C&M Cazier, (AMNH, 1); same locality, 15.vii.1956, C&M Cazier, (AMNH, 1); same locality, 7.vii.1956, M. Cazier, (AMNH, 1); same locality, 12.vii.1956, Blagden & Eaton, (AMNH, 1); same locality, 4.vii.1956, Blagden & Eaton, (AMNH, 1); W. Turkey Cr., 16.vii.1976, McCleve & Topham, (AMNH, 3); Huachuca Mts., 5200 ft., 26.vii.1949, Werner & Nutting, (UAIC, 1); Huachuca Mts., Ramsey Canyon, 28.vii.1959, Werner, Radford, Nutting, Samuelson, Patterson, blacklight trap, (DAPC, 1); Portal, 21.vii.1965, W. Rosenberg, (USNM, 1); same locality, 12.ix.1957, W. Rosenberg, (USNM, 1); Ransey Canyon, Huachuca Mts., 28.vii.1959, Werner et al., blacklight trap, (UAIC, 1); same locality, 13.vii.1955, F.G. Werner & G.D. Butler, (UAIC, 1); SW Res. Sta., 5 mi W. Portal 5400’, 1–3.viii.1959, H.E. Evans, (CUIC, 1); Turkey Crk., 31°51.280N, 109°19.883W, 14.vii.2002, ex. WPB-baited Lindgren funnel trap, ca. 2000m, (DAPC, 8); Ramsey Cyn, 5200’, 15 mi. S. Sierra Vista, Huachuca Mts., vii.1968, R.F. Sternitzky, (CNC, 5); same locality and collector, iv.v.1968, (CNC, 2); Sierra Vista, ix-x.1967, R.F. Sternitzky, (CNC, 2); Ash Canyon, 24.vii.1968, D.E. Bright, (CNC, 2).

*Pima Co*.: Bear Canyon, 6.vii.1959, R.H. Arnett, Jr., lot 391, (FSCA, 1); Madera Canyon, viii.1978, (FMNH, 1); Mt. Lemmon, Tucson, 17.vii.1970, C.A. Triplehorn, (OSUC, 1); Santa Catalina Mts., Mt. Lemmon, 12.vii.1970, C.A. Triplehorn, (OSUC, 2).

*Santa Cruz Co*.: Madera Cyn., 7–8.viii.1986, M.A. Ivie, (MTEC, 1); 5 mi. S. Canelo, 20.viii.1965, K. Brown, (UCRC, 1); Coronado N.F., Santa Rita Mts., madera Cyn, 12–13.viii.1987, C.B. & J.E. Barr, MV/BL, (LSU, 1); Coronado Nat. Forest, Madera Cyn Rec Area, Sta. Rita Lodge, 14.viii.1989, D.A. Pollock, (DAPC, 1); Madera Canyon, 1–2.viii.1988, (DAPC, 1); same locality, 12.vii.1977, Rosenberg collection, (USNM, 1); same locality, 11.vii.1977, Rosenberg collection, (USNM, 1); Madera Canyon, 4880’, 1.vii.1959, J.C. Franclemont, (CUIC, 2); same locality, 30.vi.1959, J.G. Franclemont, (CUIC, 4); same locality, 20.vii.1959, J.C. Franclemont, (CUIC, 1); same locality, 11.vii.1963, V.L. Vesterby, (DAPC, 1); same locality, 10.vii.1963, V.L. Vesterby, (DAPC, 1); same locality, 7.vii.1965, D.N. Harrington, (DAPC, 1); same locality, 19.vi.1965, D.N. Harrington, (DAPC, 1); Madera Canyon, 5100’ Bog Spring Cp. Grd. Santa Rita Mts., 10–26.vii.1964, D. Davis, (USNM, 2); Madera Canyon, 5500 feet, 1–2.viii.1988, R.A. Androw, (DAPC, 1); Patagonia, 25–26.viii.1975, L. Bezark, G. Nishida, C. Kitayama, B. Tilden, (UCBC, 1); Pena Blanca, Pajarito Mts., 2.viii.1961, Arnett & van Tassell, lot 543, (FSCA, 1); Sta Rita Mts., Madera Cyn., at amphitheater, 27.vii.1989, W.B. Warner, at uv light, (RLC, 1); Nogales, Oslar, (CUIC, 1); Patagonia, Huachuca Mts., 8.ix.1928, E.R. Leach, Eustrophus sp. n. ? det. Fall, (CMNH, 1); Santa Rita Mts., Madera Cyn., 4600–5600’, 29.vii.1978, M. Hetz, (UAIC, 1); Sta. Rita Mts., Madera Can., 8.vii.1974, C.A. Triplehorn, (OSUC, 1).

**NEW MEXICO**

*Luna Co*.: Deming, 27.vii.1977, Rosenberg collection, (USNM, 1).

### *Synstrophus repandus* (Horn)

**CANADA**

**BRITISH COLUMBIA**

5 km SW Radium, 24.viii.1987, Carrs, lot 1, (CARR, 2); same locality, 24.viii.1987, Carrs, lot 1, (DAPC, 3); 7 mi E. Terrace, 26–27.vi.1968, Campbell & Smetana, ex river debris, (CNC, 1); Copper Mountain, 21.viii.1927, G. Stace Smith, (JBWM, 3); Copper Mtn., 21.viii.1927, G. Stace Smith, *Pinus ponderosa* bark, (UBC, 4); Creston, 11.v.1958, H. & A. Howden, shelf fungus on birch; 1 spec. slightly teneral, (CNC, 8); same locality, 14.v.1958, H. & A. Howden, fungus on cottonwood, (CNC, 1); same locality, 6.ix.1951, G. Stace Smith, ex fungus, (UBC, 1); same locality, 16.viii.1951, G. Stace Smith, ex fungus, (UBC, 1); same locality, 7.ix.1951, G. Stace Smith, ex fungus, (UBC, 1); same locality, 25.x.1951, G. Stace Smith, under log, (UBC, 1); same locality, 21.v.1951, G. Stace Smith, *Populus trichocarpa*, (UBC, 1); same locality, 12.x.1951, G. Stace Smith, ex fungus, (UBC, 1); same locality, 17.v.1951, G. Stace Smith, ex fungus, (UBC, 1); same locality, 20.x.1951, G. Stace Smith, logs, (UBC, 1); same locality, 23.v.1951, G. Stace Smith, ex fungus on *Betula*, (UBC, 1); same locality, 26.v.1951, G. Stace Smith, ex fungus on *Betula*, (UBC, 2); same locality, 9.ix.1951, G. Stace Smith, ex fungus, (UBC, 1); same locality, 22.vii.1933, G. Stace Smith, underside of *Populus trichocarpa* log, (UBC, 1); same locality, 12.x.1946, G. Stace Smith, in fungus, (UBC, 1); same locality, 8.vi.1950, G. Stace Smith, ex fungus *Populus trichocarpa*, (UBC, 1); same locality, 23.vii.1948, G. Stace Smith, from fungus, (UBC, 1); same locality, 18.v.1948, G. Stace Smith, from fungus, (UBC, 2); same locality, 7.xii.1931, G. Stace Smith, on cordwood, (UBC, 1); same locality, 22.vii.1948, G. Stace Smith, from fungus, (UBC, 2); Creston, 2200 ft., 18.iii.1933, G. Stace Smith, decaying wood of *Pinus ponderosa*, (UBC, 1); Gabriola, 2.vi.1994, Carrs, lot 7, (CARR, 1); same locality, 29.iv.1993, Carrs, lot 1, (CARR, 1); same locality, 29.v.1994, Carrs, lot 8, (CARR, 3); same locality, 10.ix.1992, Carrs, lot 2, (CARR, 1); Haney, 2.vii.1962, Carrs, lot 1, (CARR, 1); Lockhart Beach, Kootenay Lk., 12.vi.1962, C.W. O’Brien, (MCZ, 1); Midday Val. Merritt, 21.vi.1926, W. Mathers, (CNC, 1); same locality, 20.iv.1926, R. Hopping, old stumps of *P. ponderosa*, (CNC, 1); same locality, 12.vii.1925, K.F. Auden, (CNC, 2); Midday Valley, Merritt, 4–7.vi.1924, K.F. Auden, (INHS, 4); same locality, 7.vi.1924, K.F. Auden, (MCZ, 1); same locality, 4.vi.1924, K.F. Auden, (PFC, 1); same locality, 11.vii.1925, K.F. Auden, (PFC, 1); same locality, 3.ix.1925, W. Mathers, *Pinus ponderosa*, (PFC, 1); same locality, 10.vi.1926, H.H. Thomas, (PFC, 1); Mirror Lake, 10.vi.1984, Carrs, lot 2, (CARR, 1); Salmon Arm, 27.iii.1929, H.B. Leech, (UBC, 1); same locality, v.1934, D.H. Leech, (UBC, 1); same locality, 22.v.1932, H. Leech, fungus, (UBC, 1); Sanca, 8.viii.1933, G. Stace Smith, on stone wall, (UBC, 1); Sleeping Beauty Mtn., NE Terrace, 1800 m., 1.vii.1987, D. & K. Larson, (MUNC, 1); Tappen, 3.vi.1978, Carrs, lot 1, (CARR, 9); same locality, 3.vi.1978, Carrs, lot 1, (DAPC, 1); Vancouver, 15.iii.1896, (CMNH, 5); Vancouver Island, 15.iii.1899, (UMRM, 1); Victoria, (INHS, 2); Wynndel, 13.v.1958, H. & A. Howden, (CNC, 1).

**MANITOBA**

Aubigny, 24.viii.1987, J.E. Blatta, (JBWM, 2); Aweme, 20.vi.1910, E. Criddle, (JBWM, 1); Sandilands Res, 5 J 1938, D.N. Smith, (CNC, 1); Winnipeg, University of Manitoba campus, honey house, 10–17.vi.1991, R.E. Roughley, suction trap, (JBWM, 1); Nopiming Prov. Pk., near Long Lake 50°52'05"N, 95°27'25"W; 18.vii.1997, C. Wytrykush, blacklight trap, (DAPC, 1); Lockport, 50°05'13"N, 96°56'17"W, on dead trees/logs at night along shore of Red River, 4.vii.2008, D.A. Pollock, (DAPC, 2); same locality and collector, 2.vii.2009, (DAPC, 1); same locality and collector, 19.vii.2010, (DAPC, 32).

**ONTARIO**

no other data, (MCZ, 1); Bell’s Corners, 17.x.1962, D.D. Munroe, (CNC, 1); Brant Co., Pinehurst Cons. Area, Hwy 24A, n. of Paris, 21.v.1971, H. Frania, in fleshy bracket fungus in woods, (ROME, 5); Chalk River, 18.vii.1985, J.V.R. Chenier, (CMNC, 1); Constance Bay, 4.ix.1965, D.D. Munroe, (CNC, 1); Dunn Twp, 1.ix.1971, W.W. Judd, (CNC, 1); Essex Co., Point Pelee Natl. Pk. East beach, 8.vi.2000, E. Reichert, (DEBU, 1); same locality, [8.vi.2000], O. Lonsdale, under bark, (DEBU, 1); Essex Co., Windsor, ~1.5km S Ojibway Prairie , 1–19.viii.2001, P. Pratt, prairie remnant, forest edge, malaise trap, (DEBU, 1); Fitzroy Hbr, 31.v.1965, D.D. Munroe, (CNC, 1); Grand Bend, 22.vi.1936, A.A. Wood, (CNC, 1); Hamilton, 3–17.vi.1982, M. Sanborne, (CNC, 1); same locality, 31.vii.1980, M. Sanborne, (CNC, 1); same locality, 7.vi.1981, M. Sanborne, (CNC, 4); same locality, 28.viii.1980, M. Sanborne, (CNC, 3); same locality, 14–19.vii.1981, M. Sanborne, (CNC, 1); same locality, 14–21.vi.1981, M. Sanborne, (CNC, 1); same locality, 9–19.ix.1981, M. Sanborne, (CNC, 1); same locality, 16.vii.1980, M. Sanborne, (CNC, 1); same locality, 28.vi-14.vii.1982, M. Sanborne, (CNC, 1); same locality, 23.vii.1980, M. Sanborne, Malaise trap, (CNC, 1); same locality, 27.vii-9.viii.1982, M. Sanborne, (CNC, 2); same locality, 23.v-3.vi.1982, M. Sanborne, (CNC, 1); same locality, 14–26.vii.1982, M. Sanborne, (CNC, 1); Lambton Co., Pinery Prov. Park , 20.vi.1994, J. Skevington, malaise Cedar trail oak savanna meadow, (DEBU, 1); same locality, 26.vi.1994, J. Skevington, malaise Cedar trail oak savanna meadow, (DEBU, 1); Ottawa, (CNC, 4); same locality, 4.viii, fungus, (CNC, 1); same locality, 22.viii.1912, Beaulieu, (CNC, 1); same locality, 27.viii.1912, Beaulieu, (MCZ, 4); Peel Co., Bolton, 4.v.1978, R.S. Anderson, under bark, (ROME, 1); Peel Co., Port Credit, 29.iv.1966, I.M. Smith, (ROME, 3); same locality, 18.vii.1965, I.M. Smith, (ROME, 1); same locality, 24.vii.1965, I.M. Smith, (ROME, 1); same locality, 16.iv.1966, I.M. Smith, (ROME, 2); Prince Edward Co., 30.iv.1922, Brimley, (CNC, 1); same locality, 2.vi.1940, Brimley, (CNC, 2) same locality, 1.vi.1930, Brimley, (CNC, 1); same locality, 31.v.1942, Brimley, (CNC, 1); same locality, 12.x.1919, Brimley, (CNC, 1); same locality, 29.vi.1941, J.F. Brimley, (FMNH, 1); Rainy River Dist., 22.vi.1924, J.F. Brimley, (CNC, 1); Reg. Mun. of Ottawa-Carleston Haldiman, 9.vi.1995, Brendon Larson, Twp. 5 km NW Centreton conch on dead Pop. Trem., (DEBU, 1); Rondeau Pr. Pk., Black Oak Trail, 5.vi.1985, A. Davies & J.M. Campbell, under wet moldy bark on dead tree, (CNC, 23); St. Thomas, 28.vii.1925, James, (CNC, 1); Strathroy, 20.vi.1934, H.F. Hudson, (CNC, 1); Sudbury, 1889, (CNC, 1); Toronto, 20.xi.1895, R.J. Crew, (CUIC, 2); same locality, 24.viii.1895, R.J. Crew, (CUIC, 5); same locality, 11.xi.1894, R.J. Crew, (CUIC, 1); same locality, 31.viii.1895, R.J. Crew, (CUIC, 1); same locality, 24.xii.1894, R.J. Crew, (CUIC, 2); same locality, 7.x.1978, R.S. Anderson, (ROME, 2); Dundas, 16.v.1979, Dael Morris, (DEBU, 1); Gananoque, 6.vii.1963, C.J. Edwards, (DEBU, 1); Georgetown, 27.v.1988, R. Gagne, Malaise trap, (DEBU, 1); Guelph, 9.v.1975, J.M. Heraty, (DEBU, 4); same locality, 15.vii.1975, Sandra Allan, (DEBU, 1); same locality, 25.vii.1982, W. Punchihewa, (DEBU, 1); same locality, 17.vii.1976, S.A. Marshall, (DEBU, 1); same locality, 10.v.1975, J. Umphrey, (DEBU, 4); Haulock, 9.vii.1977, Mccreadie, (DEBU, 1); Kingsville, 8.vii.1977, A.A. Konecny, (DEBU, 1); Lanark, 2.viii.1976, M.J. Sharkey, (DEBU, 1); London, W. Saunders, (DEBU, 1); Norval, 16.v.1980, Kevin Barber, (DEBU, 1); Toronto, 10.vii.1977, W.A. Attwater, (DEBU, 1); Vineland, 20.viii.1930, W.L. Putman, (DEBU, 1).

**QUEBEC**

Aylmer, Queen’s Park, 24.x.1923, C.B. Hutchings, (CNC, 1); Chelsea, 25.vi.1912, A. Gibson, (CNC, 1); Deux-Montagnes, Parc Paul-Sauvé, 9.iv.1989, S. Laplante, (SLC, 1); Dorval, 26.ix.1985, LeSage & Smetana, Ex. *Pleurotus ostreatus* in decid. forest, (CNC, 2); Gatineau, Eardley, 11.vi.1994, F. Genier, (CMNC, 1); Ile-de-Mtl, bois de Saraguay, 21.iv.1979, S. Laplante, (SLC, 3); Limbour Touraine, 12.viii.1974, R. Sexton, (CNC, 1); Masham Twp., Gatineau Co., 27.viii.1975, D.M. Wood, teneral, (CNC, 3); Meach Lk., Gatineau Park, 11.viii.1976, J.M. Campbell, (CNC, 1); Missisquoi, le Petit Pinacle, 24.iv.1979, S. Laplante, (SLC, 1); Montreal, 24.vii.1970, E.J. Kiteley, (CNC, 1); same locality, 1.vii.1970, E.J. Kiteley, fungus on log, (CNC, 1); same locality, 8.vi.1976, E.J. Kiteley, on fungus, stump, (CNC, 4); same locality, 12.vi.1981, E.J. Kiteley, on fungus, stump, (CNC, 1); same locality, 28.vi.1982, E.J. Kiteley, uv light trap, (CNC, 1); same locality, 12.viii.1972, E.J. Kiteley, on log fungus, (CNC, 2); same locality, 1.ix.1972, E.J. Kiteley, (CNC, 1); same locality, 11.vii.1982, E.J. Kiteley, uv light trap, (CNC, 1); same locality, (MCZ, 1); Pontiac, Eardley, 24.viii.1991, S. Laplante, polypore on branch of old *Quercus rubrus*, beaten, (SLC, 1); Terrebonne, 17.viii.1918, F. T. Scott, (LACM, 4); same locality, 17.viii.1918, (UAIC, 2); Vaudreuil, Rigaud, 16.vi.1990, S. Laplante, (SLC, 2).

**UNITED STATES OF AMERICA**

**ALABAMA**

*Bibb Co*.: Trio, 16.vi.1965, R. Freitag & D. Larson, (MUNC, 1); West Blocton, 21–22.vi.1977, John F. Reinert, Malaise trap, (USNM, 1).

*Greene Co*.: 5 mi. N.E. Eutaw, 9.vii.1950, T. Cohn, M. Cazier, 150 ft., (AMNH, 1).

*Lee Co*.: 8.i.1983, D.A. Rider, collected under bark, (LSU, 1); 0.3 mi. S. Co 26, 0.1 mi. W. US 29, 27.vi.1982, D.A. Rider, collected at light, (LSU, 1); Auburn, 7.iv.1972, E.J. Kiteley, (CNC, 1); same locality, 24.iv.1966, E.J. Kiteley, (CNC, 1); same locality, 30.iii.1969, E.J. Kiteley, under dead decid. tree bk., (CNC, 2); same locality, 8.iii.1968, E.J. Kiteley, under dead pine bark, (CNC, 1); same locality, 21.iv.1966, E.J. Kiteley, (CNC, 1); same locality, 18.iv.1954, H. Cunningham, (INHS, 1); same locality, 26.x.1952, H. Cunningham, (INHS, 1).

*Madison Co*.: Monte Sano St. Pk., 3–4.ix.1983, N.M. Downie, (FMNH, 1).

*Mobile Co*.: Mobile, vi.1927, Darlington, (MCZ, 2); same locality, 10.iii.1948, W. Rosenberg, (USNM, 1).

*Shelby Co*.: Alabaster, 30.iv.1954, (FMNH, 1).

**ARKANSAS**

*Faulkner Co*.: Bell Slough Wildlife Management Area, Mayflower near Lake Conway Spillway, 24.viii.1986, R.A.B. Leschen, *Meripilus giganteus*, (RLC, 2).

*Franklin Co*.: Shores Lake Camp, Ozark Nat. Forest, 3.vi.1968, N.T. Baker & E.J. Kochenderfer, (MSUC, 1).

*Fulton Co*.: Salem, 23.vi.1983, Carrs, lot 8, (DAPC, 2).

*Hempstead Co*.: Hope, 18.iii.1922, L. Knobel, (UMMZ, 1).

*Pulaski Co*.: Little Rock, 21–24.iv.1997, Brian Baldwin, malaise trap, (UGAC, 2); same locality, 22.vi.1997, Brian Baldwin, malaise trap, (UGAC, 1); same locality, 25.v.1997, B. Baldwin, Malaise trap, (BBC, 1); same locality and collector, 2.iv.1998, Malaise trap (BBC, 1); 8.vii.2001, Malaise trap, (BBC, 1); 24.viii.2001, Malaise trap, (BBC, 1); 3.iv.1999, Malaise trap, (BBC, 1); 3.x.1999, Malaise trap, (BBC, 1); 21.v.1999, Malaise trap, (BBC, 1); 13.iv.2001, flight intercept trap, (BBC, 1); 31.vii.2003, Malaise trap, (BBC, 1); 11.vi.2001, Malaise trap, (BBC, 1); 3.ix.1998, Malaise trap, (BBC, 1); 26.vii.1998, Malaise trap, (BBC, 1); 9.x.1998, Malaise trap, (BBC, 1); 9.ix.2001, Malaise trap, (BBC, 1); 13.vi.1997, Malaise trap, (BBC, 1).

*Counties unknown*: South West, Chas Palm, Ac. 5409, (AMNH, 4).

**CALIFORNIA**

H.W. Wenzel collection, (OSUC, 2).

*Riverside Co*., Indio, 24.ix.1977, L.D. Moore, (BYUC, 1).

*Shasta Co*.: Castle Crag, (CUIC, 1).

*Siskiyou Co*.: Klamath River, 16 km N. Happy Camp, 16.vii.1976, J.E. Rawlins, (CMNH, 1).

**CONNECTICUT**

*Litchfield Co*.: Cornwall, 20.ix.1921, K.F. Chamberlain, (LACM, 3); Salisbury, 21–25.viii.1989, M. O’Brien, (UMMZ, 2); same locality, 9.ix.1982, M. & A. O’Brien, at light, (UMMZ, 1); same locality, 28.viii.1978, A. & M. O’Brien, (UMMZ, 2).

*New Haven Co*.: North Branford (Northford: 22 Foote Hill Rd.) 41°23'23"N, 72°48'15"W, 25.vii.1995, M.K. Oliver, incandescent porch light, (MKOC, 1).

**DISTRICT OF COLUMBIA**

Ulke coll., (CMNH, 2).

**DELAWARE**

*New Castle Co*.: Newark, 11.iv.1905, C.O. Houghton, (INHS, 1).

**FLORIDA**

no other data, (INHS, 1).

*Alachua Co*.: 4 mi. N. of LaCrosse J. & E. Ranch, 11–13.ix.1989, L.R. Davis, Jr., (FSCA, 1); Gainesville, 23–30.iv.1987, FIT in oak forest, (DAPC, 5).

*Clay Co*.: Gold Head Branch St. Pk., 5.x.1994, C. Porter & L. Stange, turkey oak - rosemary, (FSCA, 1).

*Franklin Co*.: Apalachicola Natl. For., Wright Lake, 28.iv.1991, L.R. Davis, Jr., (FSCA, 1).

*Jackson Co*.: Torreya St. Pk., 25.iii.1986, Q.D. Wheeler, floodplain, ex fungi & under bark, (CUIC, 1).

*Liberty Co*.: Torreya St. Pk., 25.iii.1986, J. Parkinson, floodplane, (CUIC, 1); same locality, 19–23.iv.1984, E.G. & M.A. Riley, (EGRC, 5).

*Leon Co*.: Tallahassee, 21.iii.1982, C.W. O’Brien, light, (DAPC, 1).

*Orange Co*.: Rk Spr Rn St Res, 27.viii.1994, J.C. Longhurst, Spine/Oak scrub malaise trap, (UCFC, 1); same locality, 31.v.1995, S.M. Fullerton spring students, Spine/Oak scrub malaise trap, (UCFC, 1); UCF Orlando, 17.iii.1999, P. Russell & S. Fullerton, cypress forest malaise trap, (UCFC, 2); same locality, 16.x.1997, S.M. Fullerton, LLP-sand pine turkeyoak malaise trap, (UCFC, 1); same locality, 17.xi.1997, S.M. Fullerton, LLP-sand pine turkeyoak malaise trap, (UCFC, 1); same locality, 9.xi.1997, S.M. Fullerton, LLP-sand pine turkeyoak malaise trap, (UCFC, 1); same locality, 23.x.1997, S.M. Fullerton, LLP-sand pine turkeyoak malaise trap, (UCFC, 1); same locality, 7.viii.1997, S.M. Fullerton, LLP-sand pine turkeyoak malaise trap, (UCFC, 1); same locality, 5.iv.1999, P. Russell & S. Fullerton, pond pine comm. Dahoon holly malaise trap, (UCFC, 1); same locality, 2.I.1998, S.M. Fullerton, LLP-sand pine turkeyoak malaise trap, (UCFC, 1); same locality, 21.xi.1992, S.M. Fullerton, LLP-sand pine turkeyoak malaise trap, (UCFC, 1); same locality, 8.v.1993, S.M. Fullerton, LLP-sand pine turkeyoak malaise trap, (UCFC, 1); same locality, 23.viii.1992, S.M. Fullerton, LLP-sand pine turkeyoak malaise trap, (UCFC, 1); same locality, 27.ix.1992, S.M. Fullerton, LLP-sand pine turkeyoak malaise trap, (UCFC, 1); same locality, 10.x.1992, S.M. Fullerton, LLP-sand pine turkeyoak malaise trap, (UCFC, 1); same locality, 27.iii.1993, S.M. Fullerton, LLP-sand pine turkeyoak malaise trap, (UCFC, 1); same locality, 21.xii.1992, S.M. Fullerton, Sand pine-rosemary scrub malaise trap, (UCFC, 1); same locality, 24.xii.1992, S.M. Fullerton, Sand pine-rosemary scrub malaise trap, (UCFC, 1); same locality, 21.ix.1992, S.M. Fullerton, LLP-sand pine turkeyoak malaise trap, (UCFC, 1); same locality, 12.ix.1992, S.M. Fullerton, LLP-sand pine turkeyoak malaise trap, (UCFC, 2); same locality, 8.I.1993, S.M. Fullerton, LLP-sand pine turkeyoak malaise trap, (UCFC, 1); same locality, 31.v.1995, S.M. Fullerton, LLP-sand pine turkeyoak malaise trap, (UCFC, 1); same locality, 13.ii.1993, S.M. Fullerton, LLP-sand pine turkeyoak malaise trap, (UCFC, 1); same locality, 23.iv.1993, S.M. Fullerton, LLP-sand pine turkeyoak malaise trap, (UCFC, 1); same locality, 12.xi.1992, S.M. Fullerton, LLP-sand pine turkeyoak malaise trap, (UCFC, 2); same locality, 31.x.1992, S.M. Fullerton, LLP-sand pine turkeyoak malaise trap, (UCFC, 1); same locality, 8.v.1993, S.M. Fullerton, LLP-sand pine turkeyoak malaise trap, (UCFC, 1); same locality, 2.I.1993, S.M. Fullerton, LLP-sand pine turkeyoak malaise trap, (UCFC, 1); same locality, 28.xiii.1999, P. Russell, T. Smith & S. Fullerton, cypress forest malaise trap, (UCFC, 1).

*Wakulla Co*.: Apalachicola N. Forest, S.W. Tallahassee, 9.v.1985, J. Doyen & W. Tschinkel, (UCBC, 4).

**GEORGIA**

*Baker Co*.: Emory Un. Field Sta., Newton, 19.xi.1952, (CMNC, 1); same locality, 1.vii.1957, light, (CNC, 1).

*Bibb Co*.: 28.iii.1973, R.T. Franklin, (UGAC, 1).

*Bulloch Co*.: Statesboro, 24–27.v.1983, J.F. Burger, malaise, (DENH, 1).

*Charlton Co*.: 25.iii.1985, R. Morris, (UGAC, 1); 24.iv.1984, R. Morris, (UGAC, 1).

*Chattahoochee Co*.: Fort Benning, 7.vi.1942, J.H. Kremer, (FMNH, 1).

*Clarke Co*.: 1.1 mi SW Winterville, 24–28.vi.1994, C.L. Smith, Malaise trap, (UGAC, 1); same locality, 12–15.vii.1994, C.L. Smith, malaise trap, (UGAC, 2); Athens, 1–4.xii.1997, W.B. Sikora, Malaise trap, (UGAC, 1); same locality, 26.v.1929, P. W. Fattig, (UGAC, 1); same locality, 24.vi.1938, P. W. Fattig, (UGAC, 1); same locality, 30.vii.1938, P. W. Fattig, (UGAC, 1); same locality, 12.iii.1938, P. W. Fattig, (UGAC, 1); Whitehall Forest, 9–12.v.1988, C.L. Smith, ex: malaise trap, (UGAC, 1); same locality, 16–19.v.1988, C.L. Smith, ex: malaise trap, (UGAC, 1); same locality, 18.viii.1983, R. T. Franklin, ex: malaise trap, (UGAC, 1); Whitehall Forest, 5.ii.1976, R. Turnbow, under pine bark, (TAMU, 2).

*Coweta Co*.: 7 km. E. Newnan, 7.iv.1980, W.E. Steiner, (USNM, 1).

*DeKalb Co*.: Stone Mt., 23.v.1927, P. W. Fattig, (UGAC, 2); same locality, 9.vi.1928, P.W. Fattig, (FMNH, 1); same locality, 29.v.1929, P.W. Fattig, (FMNH, 1); same locality, 23.v.1927, P. W. Fattig, (UGAC, 1); same locality, 28.iv.1927, P. W. Fattig, (UGAC, 2).

*Floyd Co*.: Rome, 23.xi.1977, R. Wharton, (TAMU, 1).

*Fulton Co*.: Atlanta, 1.vii.1938, P.W. Fattig, (FMNH, 1).

*Greene Co*.: 15 km SE Watkinsville, 24.iii.1995, K. McCravey, ex: malaise trap, (UGAC, 1).

*Henry Co*.: 2.vii.1983, R.J. Beshear, (UGAC, 1).

*Oglethorpe Co*.: 3 mi SSW Arnoldsville, 31.v.1988, J. Jackson, ex: black light, (UGAC, 1).

*Paulding Co*.: Dallas, 19.vii.1942, P. W. Fattig, (UGAC, 1).

*Whitfield Co*.: Carbondale, 4.v.1996, D. Yanega, (INHS, 1).

*Counties unknown*: L.S. Walker S.P., 3.v.1964, L.J. Bayer, (WIRC, 2).

**IDAHO**

no other data, (MCZ, 1).

*Bonner Co*.: Priest Riv , 21.vi, Wickham, (USNM, 1); Sandpoint, 11.viii.1954, N.M. Downie, (FMNH, 2); same locality, 24.vii.1954, N.M. Downie, (AMNH, 3).

*Kootenai Co*.: Ceurd’Al, L.B. Woodruff, (AMNH, 1); Ceurd’Alene, vi, Wickham, (USNM, 1).

**ILLINOIS**

Ulke coll., (CMNH, 1); no other data, (INHS, 9); no other data, (MCZ, 2); [N. Ill.], (NDSU, 2).

*Alexander Co*.: Olive Branch, 7.x.1909, (FMNH, 3).

*Champaign Co*.: Brownfield Woods, 3 mi. NE Urbana, 18.iv.1977, C.T. Maier, (INHS, 6); same locality, 12.iv.1977, C.T. Maier, (INHS, 2); Homer, 27.iv.1907, (INHS, 1); Mahomet, 30.ix.1959, J.M. Campbell, (CNC, 1); Urbana, 23.v.1933, (FMNH, 1); same locality, 14.iv.1907, (INHS, 1).

*Clark Co*.: Rocky Branch, 13–24.vi.1991, M.A. Goodrich, Malaise trap, (DAPC, 1); same locality, 2–9.vii.1990, M.A. Goodrich, malaise trap, (EIUC, 1); same locality, 16.ix.1991, M.A. Goodrich & R.S. Hanley, (EIUC, 1); same locality, 29.v-2.vi.1991, M.A. Goodrich, malaise trap, (EIUC, 1); same locality, 8–19.viii.1991, M.A. Goodrich, malaise trap, (EIUC, 1); Westfield, 7.vi.1936, (EIUC, 1).

*Coles Co*.: 29.ix.1965, M.A. Goodrich, (EIUC, 3); 20.vii.1922, Williams, (EIUC, 1); 14.x.1972, R. Kearns, (EIUC, 1); Embar. River Bluff, 27.vii.1970, Decker, (EIUC, 1); Lakeview Park, 10–11.iv.1999, M.A. Goodrich, UV light trap, (EIUC, 1).

*Cook Co*.: E.B. Chope, (FMNH, 2); Midlothian, 13.v.1992, R. Garner, (FMNH, 1); Willow Springs, 17.vii.1907, E.B. Chope, (FMNH, 1).

*DeKalb Co*.: Somonauk, 17.v.1930, (FMNH, 1).

*Edgar Co*.: 4 mi SSE Kansas, 3–10.v.2000, M.A. Goodrich, malaise trap, (EIUC, 1).

*Jackson Co*.: 7 mi W. Carbondale, 15–22.v.1993, M.A. Goodrich & D.L. Wood, Malaise trap, (DAPC, 1).

*Jasper Co*.: 3.5 mi. W. Rose Hill, West bank of Embarras River, 5.v.1973, L.R. Davis, Jr., (FSCA, 16).

*La Salle Co*.: Buffalo Rock Sp, 25.iv.1994, J.W. Griffiths, malaise trap, (EIUC, 1); same locality, 1–8.viii.1994, J.W. Griffiths, malaise trap, (EIUC, 1); Starved Rock St. Pk., 25.iv.1970, V. Reaves, (FMNH, 1); Ottawa, 5.v.1946, F.G. Werner, (UAIC, 4).

*Ogle Co*.: Grand Detour, 2.ix.1932, E. Brundage Jr., (FMNH, 1).

*Piatt Co*.: White Heath, 29.x.1939, J.C. Dirks, rotten log No. 6, (INHS, 1).

*St. Clair Co*.: Kahokia, 1.vi.1919, (UMRM, 2).

*Sangamon Co*.: Springfield, 23.iii.1926, (FMNH, 1); same locality, 25.iv.1926, (FMNH, 4).

*Union Co*.: Pine Hills Field Sta., 15–22.v.1967, J.M. Campbell, berlese large mushroom on log site, (CNC, 7); Wolf Lake (Pine Hills), 26.vi.1958, H.S. Dybas, ex *Polyporus adustus*, (FMNH, 1); same locality, 26.vi.1958, H.S. Dybas, on dead beech bark among fungi, (FMNH, 2).

*Vermilion Co*.: Forest Glen F.P., 5 mi. SE Westville, 14–15.iv.1977, D.W. Webb, (INHS, 1); same locality, 19–20.iv.1977, D.W. Webb, (INHS, 2); same locality, 21–22.iv.1977, D.W. Webb, (INHS, 2).

*Wabash Co*.: Beall Woods N.P., 15.viii.1996, M.A. Goodrich, *Pleurotus ostreatus*, (DAPC, 2); same locality, 15–23.viii.1996, M.A. Goodrich, malaise trap, (DAPC, 1); same locality, 7–15.viii.1996, M.A. Goodrich, malaise trap, (DAPC, 1); same locality, 31.viii-7.ix.1995, M.A. Goodrich, Malaise trap, (DAPC, 1); same locality, 15.viii.1996, M.A. Goodrich, ex *Pleurotus ostreatus*, (EIUC, 1); same locality, 11–17.vii.1996, M.A. Goodrich, malaise trap, (EIUC, 1); same locality, 12–18.x.1996, M.A. Goodrich, malaise trap, (EIUC, 3); same locality, 27.vi-4.vii.1996, M.A. Goodrich, malaise trap, (EIUC, 1); same locality, 20.ix.1996, M.A. Goodrich, *Laetiporus sulphureus*, (EIUC, 2); same locality, 7–15.viii.1996, M.A. Goodrich, malaise trap, (EIUC, 2); same locality, 7–14.v.1997, M.A. Goodrich, Malaise trap, (EIUC, 2); same locality, 14–21.v.1997, M.A. Goodrich, Malaise trap, (EIUC, 2).

**INDIANA**

no other data, (CUIC, 1).

*Clinton Co*.: Michigan, 15.xi.1931, H. Seevers, (FMNH, 1).

*LaPorte Co*.: Michigan City, 15.v.1926, (FMNH, 1); Smith Sta., 14.x.1939, M. Bain, (FMNH, 1).

*Monroe Co*.: Bloomington, 4–5.vi.1980, F.N. Young, blt, (FSCA, 1); same locality, 28–30.v.1980, F.N. Young, blt, (FSCA, 1); same locality, 24.v.1990, F.N. Young, blt, (FSCA, 1); same locality, 30.vi.1990, F.N. Young, blt, (FSCA, 1); 1 mi. S. Lake Monroe, 1.ix.1985, R.S. Miller, (MTEC, 1).

*Parke Co*.: 1.viii.1964, N.M. Downie, (FMNH, 1); Turkey Run Sp, 17.vi.2001, L.E. Crofutt, (EIUC, 1).

*Porter Co*.: Indiana Dunes N.L., Miller Woods, 16.viii-19.ix.1995, R. Grundel, fit, (DAPC, 1); Chesterton, Coffee Creek, 5.x.1946, (UMMZ, 1).

*Putnam Co*.: 4 mi. W. Cloverdale, 3.iii.1988, N.K. & R.S. Miller, (MTEC, 1).

*Spencer Co*.: 2 mi. N. Dale, 24.v.1994, R.A. Androw & K. Will, (DAPC, 1).

*Tippecanoe Co*.: 7.vii.1970, N.M. Downie, (FMNH, 1); 24.vi.1977, N.M. Downie, (FMNH, 3); 25.iv.1954, N.M. Downie, (FMNH, 1); 13.v.1961, N.M. Downie, (USNM, 1).

*Vigo Co*.: 16.iii.1905, (FMNH, 1).

**IOWA**

*Dickinson Co*.: Spirit Lake, vi.1896, (WIRC, 1).

*Story Co*.: Ames, 22.v.1932, L.C. Glover, (DENH, 1).

**KANSAS**

no other data, (MCZ, 1).

**KENTUCKY**

no other data, (CMNH, 1); no other data, (MCZ, 1).

*Bath Co*.: Daniel Boone N.F. 4 mi from Clear Ck. Rec. Area on Rd, 5.xii.1988, R.M. Reeve, sift rotten stump, (DENH, 1).

*Henderson Co*.: Henderson, 10.iv.1921, M.Y. Marshall, (UMMZ, 1); same locality, 6.v.1923, M.Y. Marshall, (UMMZ, 1).

**LOUISIANA**

*E. Baton Rouge Par*.: Baton Rouge, 16.v.1983, E.G. Riley, coll. on carrion, (LSU, 1); same locality, 29.x.1986, E.E. Douglas, (LSU, 1).

*Rapides Par*.: Magnolia Rec Area, 15 mi. SW Alexandria, 4.x.1973, pine hardwood, (MCZ, 1); Alexandria, 15.viii.1965, R.M. Weselah, (BYUC, 10).

*Washington Par*.: Lee Mem. For., Sheridan, 10.v.1986, C.B. & J.E. Barr, MV & BL, (LSU, 1).

*parish unknown*: Fountainbleau S.P., 3.xii.1975, J. Liebherr, under *Pinus* bark, (UCBC, 1).

**MAINE**

*Androscoggin Co*.: Wales, 18.vi.1907, C.A. Frost, (MCZ, 1).

*Cumberland Co*.: Westbrook, 28.iv.1980, D. Jump, (USNM, 1).

*Kennebec Co*.: Monmouth, 27.vi.1912, C.A. Frost, (MCZ, 1).

*York Co*.: West Lebanon, 3–9.vi.1991, D.W. Barry, malaise, (DENH, 2); same locality, 5–9.vii.1990, D.W. Barry, UV trap, (DENH, 1); same locality, 24–30.vi.1991, D.W. Barry, UV light, (DENH, 1).

*Counties unknown*: Wissataquoik River, 25.vi.1901, (MCZ, 1).

**MARYLAND**

Ulke coll., (CMNH, 1); J.A. Spencer Collection 1874, (WIRC, 1).

*Anne Arudel Co*.: Edgewater, 27.vi-2.vii.1984, C.L. Staines, Jr., at black light, (USNM, 1).

*Calvert Co*.: Flag Ponds Nature Park, 9.vi.1987, A. & B. Norden, D. Williams, (USNM, 1); same locality, 20.vii.1987, A. & B. Norden, D. Williams, (USNM, 1).

*Cecil Co*.: Port Deposit, 24.iv.1977, D. Jump, (USNM, 1).

*Montgomery Co*.: 4 mi SW of Ashton, 28.v.1986, G.F. & J.F. Hevel, (USNM, 1); 9.vii.1918, E. Shoemaker, (USNM, 1); Plummer’s I, 10.vii.1902, Barber & Schwarz, (USNM, 1); same locality, 1.ix.1907, E.A. Schwarz, (USNM, 1); same locality, 15.viii.1905, E.A. Schwarz, (USNM, 1); same locality, 2.vii.1907, H.S. Barber, (USNM, 1); same locality, 1.ii.1907, E.A. Schwarz, (USNM, 1); Plummer’s I[Island?], 2.v.1908, W.L. McAtee, (USNM, 1); Plummers Island, 8–20.vii.1968, Paul J. Spangler, (USNM, 4); Plummers Island, (CUIC, 1).

*Prince George’s Co*.: Camp Springs, 19.vii.1979, Gary F. Hevel, Malaise trap, (USNM, 1); Baldensburg, 14.v.1974, M. Druckenbrod, rot lumb, (USNM, 1); Upper Marlboro, 12.v.1971, K. McKnight, Taken on *Pleurotus ostreatus*, (USNM, 1).

*Washington Co*.: 5 km. NE Boonsboro Greenbrier St. Pk., 8–10.viii.1986, W.E. Steiner, J.M. Swearingen, M.J. & R. Molineaux, Malaise trap in mixed forest, (USNM, 1).

*Worcester Co*.: Pocomoke St. Forest, 30.ix.1970, Robert Gordon, (USNM, 1).

*Counties unknown*: Williamspt, W.F. Pennington, (USNM, 2).

**MASSACHUSETTS**

*Hampden Co*.: Springfield, G. Dimmock, (MCZ, 1).

*Hampshire Co*.: Amherst, 20.xi.1967, R.D. Field, (AMNH, 1).

*Middlesex Co*.: Lowell, (MCZ, 1).

**MICHIGAN**

no other data, (FMNH, 4); no other data, (MCZ, 1).

*Alger Co*.: Onota Twp., 27.vii.1916, A. W. Andrews, (UMMZ, 1).

*Allegan Co*.: Saugatuck Dunes S.P., 24.vi.1984, M. & A. O’Brien, (UMMZ, 2).

*Alpena Co*.: Alpena, W.A. Nason, (INHS, 1).

*Arenac Co*.: Saginaw Bay, Charity Is., 25.vi.1910, A.W. Andrews, (UMMZ, 1).

*Berrien Co*.: Harbert, 13.vii.1974, J. Clough, (FMNH, 1); Harbert Dunes, 28.vii.1917, A.W. Andrews, (UMMZ, 2); Paw Paw Lake, 22.vii.1909, E. Liljeblad, (UMMZ, 1).

*Charlevoix Co*.: High Isl., 21.viii.1923, S. Moore, (UMMZ, 2).

*Cheboygan Co*.: 5.viii.1941, T.W. Porter, (MSUC, 1); Cheboygan, 8–10–1928, W. Clanton, (UMMZ, 1).

*Eaton Co*.: Eaton Rapids, 6.viii.1975, J. Liebherr, (UCBC, 1).

*Ingham Co*.: 19.vi.1947, R.L. Fischer, (MSUC, 1); E. Lansing, 12.iv.1954, R.L. Fischer, (MSUC, 1); same locality, 9.iii.1927, L.G. Gentner, (MSUC, 1); same locality, 26.ix.1964, B. & T. Hlavac, (MSUC, 1); same locality, 22.iv.1972, D.K. Young, (EGRC, 2); East Lansing, 26.ix.1964, B. & T. Hlavac, (FMNH, 1); same locality, 6.v.1947, D.M. Tuttle, (UAIC, 7); same locality, 12.iv.1940, D.M. Tuttle, (UAIC, 1).

*Lake Co*.: Big Star Lake, 30.vi.1976, R.L. Fischer, malaise, (MSUC, 1); Branch, 24.ix.1975, J. Liebherr, mature oak stand, (CUIC, 1); Branch, em. 29.ix.1975, J. Liebherr, mature oak stand, (CUIC, 3); same locality, 29.ix.1975, J. Lieberr, mature oak stand, (UCBC, 2).

*Lenawee Co*.: T8S, R2E, Sec. 32, 9.iv.1966, M.C. Nielsen, (MSUC, 1).

*Livingston Co*.: 5 mi. E. Brighton, Kensington Park, 24.ix.1955, G.H. Nelson, under log, (AMNH, 1).

*Marquette Co*.: Conway Lake, Huron Mtn. Club, 9.vii.1981, D. Gosling, (MSUC, 1); Ives Lake, Huron Mtn. Club, 9.vii.1981, D. Gosling, (MSUC, 2); same locality, 6.viii.1985, M. & A. O’Brien, (UMMZ, 1); same locality, 9.viii.1985, M. & A. O’Brien, (UMMZ, 1); same locality, 26.v.1986, M. & A. O’Brien, (UMMZ, 1).

*Midland Co*.: 13.vi.1957, R. & K. Dreisbach, (MSUC, 4); 11.v.1946, R.R. Dreisbach, (UCRC, 1).

*Oakland Co*.: 19.v.1940, (USNM, 1); 14.vi.1931, (UMMZ, 5); 5.xi.1922, S. Moore, (UMMZ, 1); Lakeville, 31.v.1931, S. Moore, (UMMZ, 2).

*Saginaw Co*.: 5.vii.1949, R.R. Dreisbach, (MSUC, 2).

*Schoolcraft Co*.: Pointe aux Barques, 20.viii.1923, S. Moore, (UMMZ, 2); Thompson, 10.ix.1923, S. Moore, (UMMZ, 6); county record only, 1.ix.1922, S. Moore, (UMMZ, 1).

*Wayne Co*.: 8 Mile Road, 15.iv.1922, S. Moore, (UMMZ, 1); Detroit, 14.vi.1911, (UMMZ, 1); same locality, 12.viii.1900, (UMMZ, 1); 2 mi. SW Plymouth, 26.v.1946, P. Brodkorb, (UMMZ, 1).

**MINNESOTA**

*Crow Wing Co*.: Brainerd, 6.vii.1957, E.J. Kiteley, (CNC, 4).

*Hennepin Co*.: Minneapolis, 12.vii.1955, E.J. Kiteley, (CNC, 1); same locality, 23.vi.1958, A. Raske, rotten log, (CNC, 2).

*Roseau Co*.: Roseau, 3.x.1970, S. Haslerud, (UCBC, 1).

*Sherborne Co*.: Elk River, 28.vi.1955, E.J. Kiteley, (CNC, 1).

*St. Louis Co*.: Duluth, Daggett, (LACM, 1).

**MISSISSIPPI**

*Adams Co*.: Natchez, 23.xi.1978, A.E. Zuccaro, (UMIC, 1).

*Lafayette Co*.: Oxford, 11.v.1980, P.K. Lago, (UMIC, 1).

*Perry Co*.: New Augusta, 27.x.1931, H. Dietrich, (CUIC, 1).

*Stone Co*.: Perkinston, 15.vii.1927, J.P. Kislanko, (CUIC, 1).

**MISSOURI**

H.W. Wenzel collection, (OSUC, 4).

*Randolph Co*.: 1 mi. E. Moberly, 22.v.1972, E.G. Riley, (EGRC, 2); same locality, 24.vii.1976, E.G. Riley, (EGRC, 1); same locality, 11.vi.1973, E.G. Riley, (EGRC, 2); same locality, 22.vii.1972, E.G. Riley, (EGRC, 1); Valley Park, 13.v.1919, (UMRM, 1).

*St. Charles Co*.: St. Charles, 27.v.1919, (UMRM, 1).

*St. Louis Co*.: St. Louis, Baden, (UMRM, 4); same locality, 30.v.1891, (UMRM, 1).

**MONTANA**

*Flathead Co*.: Glacier National Park N. Fork Flathead area Bowman Lake Road lodgepole hardburn T35N, R21W, Sec14, 16–23.vii.1991, M.A. Ivie, 1988 Red Bench Fire, (MTEC, 1); Glacier National Park West Glacier H.Q., 1–17.viii.1992, M.A. Ivie, (MTEC, 1).

*Lake Co*.: Flathead Lake Biol. Station, Yellow Bay, 20.vii-13.viii.1989, C.B. Barr & W.D. Shepard, collected on fungus, (CBC, 3).

*Lincoln Co*.: Yaak R. mouth, 4.vii.1991, D.L. Gustafson, (MTEC, 1).

**NEBRASKA**

Ulke coll., (CMNH, 1).

*Sarpy Co*.: Omaha, Fontnelle Forest, 2.viii.1948, Dreisbach, (MSUC, 1); Fontnelle For., 12–20.vii.1997, M.A. Goodrich & C.A. Springer, Malaise trap, (EIUC, 1); same locality, 2–9.v.1998, M.A. Goodrich & C.A. Springer, Malaise trap, (EIUC, 3); same locality, 27.vi.1998, M.A. Goodrich & C.A. Springer, Malaise trap, (EIUC, 1); same locality, 9–16.v.1998, M.A. Goodrich & C.A. Springer, Malaise trap, (EIUC, 5).

**NEW HAMPSHIRE**

*Carroll Co*.: 1 mi N Wonalancet E Pk. Spring Brk. 1900’, 5–14.vi.1985, D.S. Chandler, Malaise trap, (DENH, 1); Moultonboro, 31.vii.1975, malaise trap, (LSU, 1); N. Conway, vi, (MCZ, 2).

*Grafton Co*.: Bedell bridge S.P. @ Oliverian brook, 18.vi.1994, D.S. Chandler, UV trap, (DENH, 1); Franconia, A.T. Slosson, (AMNH, 1).

*Rockingham Co*.: Hampton, 27.iv.1924, S. Albert Shaw, (DENH, 1).

*Strafford Co*.: 1 mi SW Durham, 2–9.vii.1987, D.S. Chandler, (DENH, 1).

**NEW JERSEY**

no other data, (CUIC, 1).

*Bergen Co*.: Palisades, (CUIC, 2).

*Burlington Co*.: Medford Township Lake Pine, 14.viii.1974, D.C. Rentz, (USNM, 1); same locality, 25.vi.1975, D.C. Rentz, (USNM, 1).

*Monmouth Co*.: Sandy Hook, 28.ix.1910, (AMNH, 1).

**NEW YORK**

Ulke coll., (CMNH, 1); no other data, (CUIC, 1); no other data, (FMNH, 2); no other data, (MCZ, 2).

*Erie Co*.: Buffalo, (INHS, 1).

*Livingston Co*.: Letchworth State Park, 17–20.vi.1983, E.R. Hoebeke, (CUIC, 3).

*Monroe Co*.: Webster, Webster Park, 23.iii.1969, R. Duffield, (AMNH, 2).

*Nassau Co*.: Centerlisle, 20.x.1949, T.J. Spilman, (USNM, 1).

*Niagara Co*.: Olcott, 15.viii.1922, H. Dietrich, (CUIC, 35); same locality, 18.vi.1922, H. Dietrich, (CUIC, 9); same locality, 8.vi.1924, H. Dietrich, (CUIC, 1); same locality, 4.ix.1927, H. Dietrich, (CUIC, 3); same locality, 16.ix.1921, H. Dietrich, (CUIC, 1); same locality, 27.vi.1926, H. Dietrich, (CUIC, 4); same locality, 17.viii.1924, H. Dietrich, (CUIC, 1); same locality, 15.viii.1922, H. Dietrich, (UMMZ, 1).

*Onondaga Co*.: Elbridge, 2.ix.1940, N.M. Downie, (FMNH, 1); same locality, 7.ix.1941, (NDSU, 1).

*St. Lawrence Co*.: Rossie, 10–11.viii.1959, N.M. Downie, (FMNH, 3); Canton, 23–24.vii.1941, Downie, (FMNH, 2).

*Tompkins Co*.: 17.vi.1978, N.M. Downie, (FMNH, 1); 14–15.viii.1966, N.M. Downie, (FMNH, 2); 15.viii.1959, N.M. Downie, (FMNH, 1); 16.viii.1959, N.M. Downie, (AMNH, 2); 1.vi.1957, N.M. Downie, (USNM, 1); Ithaca, 13.vi.1983, E.R. Hoebeke, (CUIC, 2); same locality, 15.vi.1983, E.R. Hoebeke, (CUIC, 1); same locality, 9.vii.1986, E.R. Hoebeke, (CUIC, 1); Ithaca, Forest Home, 1–8.vi.1981, E.R. Hoebeke, flight intercept, (CUIC, 1).

*Warren Co*.: Lk. George, 19.viii.1970, Parsons, *Pleurotus* sp., (MCZ, 1); Groton, 2.vi.1953, N.M. Downie, (FMNH, 1); Ithaca, 17.v.1966, D. Stephan, in slimey fungus under pine bark, (CUIC, 2); same locality, 26.v.1966, on attic window, (CUIC, 1); same locality, 22.v.1966, near termite colony in rotten log, (CUIC, 1); same locality, 26.vi.1949, T.J. Spilman, (USNM, 1); Ithaca, Six Mile, 20.v.1939, (CUIC, 1).

*Wayne Co*.: 19.ix.1948, (USNM, 1).

*Wyoming Co*.: Pike, 15.viii.1901, C.A. Frost, (LACM, 1).

*Counties unknown*: Bear Mt., 25.vi.1945, C.H. Curran, (AMNH, 1); NY City & vicinity, (CUIC, 1); Greenwood Lake, (CUIC, 1).

**NORTH** **CAROLINA**

no other data, (CMNH, 8); H.W. Wenzel collection, (OSUC, 6).

*Buncombe Co*.: Asheville, 2.vi.1978, L.L. Lampert, (FSCA, 1); same locality, 7.viii.1974, L.L. Lampert, (FSCA, 1); Black Mt., N. Fork Swannansa, v, (MCZ, 1); Swannanoa Val., [8.vi], (AMNH, 1).

*Durham Co*.: Duke Forest, vi.1978, blacklight, (DAPC, 1); Duke Forest, Durham, vi.1970, S. Hughes-Schrader, at black light, (UCRC, 1); Durham, vi, S. Hughes-Schraeder, blacklight, (DAPC, 1).

*Gaston Co*.: 12.xi.1972, J.S. Ashe, ex bracket fungi, (TAMU, 1).

*Henderson Co*.: Fletcher, 20.viii.1974, L.L. Lampert, (FSCA, 1); same locality, 17.vi.1971, L.L. Lampert, (FSCA, 1).

*Hertford Co*.: 9.vi.1895, (MCZ, 1).

*Jackson Co*.: Balsam, 7.vii.1948, W. Rosenberg, (USNM, 1); same locality, 25.vi.1948, W. Rosenberg, (USNM, 1); Dillsboro, [viii.1955], (AMNH, 2).

*Wake Co*.: 29.v.1958, D.H. Habeck, (FSCA, 1); Raleigh, 1.xii.1952, B.K. Dozier, under oak bark, (FSCA, 1); same locality, 12.i.1906, R.Woglum, (UCRC, 1); same locality, 10.i.1906, R. Woglum, (UCRC, 1).

**NORTH DAKOTA**

*Bottineau Co*.: 14.iv.1963, Robert Gordon, (USNM, 1); Sec 26, Twp 164, R. 75, 27.vi.1967, J.D. Stein & J. Knudson, (NDSU, 1).

*Cass Co*.: 23.iv.1962, Robert Tetrault, (MTEC, 1).

*Richland Co*.: 4-H Camp, 7.v.1962, (NDSU, 1); Walcott Dunes, 19.v.1975, P.K. Lago, (PKLC, 1).

**OHIO**

no other data, (MCZ, 4).

*Ashtabula Co*.: Jefferson, viii.1908, R. Sim, (CUIC, 1); same locality, viii.1908, Robt. Sim, (OSUC, 2).

*Butler Co*.: Oxford, 5.v.1954, (CUIC, 1).

*Clark Co*.: Springfield Fen, 14–22.vi.1989, R.S. Miller & family, (MTEC, 1).

*Clermont Co*.: 20.vi.1963, R.D. Mathis, (OSUC, 1).

*Clinton Co*.: 18.vi.1962, R.D. Mathis, (OSUC, 1).

*Cuyahoga Co*.: Cleveland, Rocky River Reservation, 11.viii.1985, R. Davidson & H. Lee, (CMNH, 3).

*Delaware Co*.: jct. Deer Creek & Scioto River, 21.vii.1984, S.M. Clark, (MTEC, 1).

*Fairfield Co*.: 15.viii, D.J. & J.N. Knull, (OSUC, 1).

*Hocking Co*.: 10.ix.1954, D.J. & J.N. Knull, (OSUC, 1); Conkles Hollow St. Pk., 26.v.1980, M.A. & L.L. Ivie, (MTEC, 2).

*Jackson Co*.: Lake Katherine, 2–15.vii.1989, R.S. Miller & family, (MTEC, 1).

*Knox Co*.: Gambier, 27.vi.1975, D.S. Chandler, (UAIC, 1).

*Lake Co*.: Willoughby, 21.v.1965, J.C. Pallister, (AMNH, 1).

*Ross Co*.: Tar Hollow St. For., 24.v.1986, R.S. Miller, (MTEC, 2); same locality, 14–22.vi.1988, R.S. Miller & family, (MTEC, 1); same locality, 15.vi.1983, M.A. Ivie, (MTEC, 1).

*Union Co*.: 22.vii.1974, Q.D. Wheeler, (CUIC, 1).

*Vinton Co*.: Vinton Furness , 19.vi.1985, R.S. Miller, (MTEC, 1); Vinton Furness nr. Dundas, 19.vi.1985, R.S. Miller, (MTEC, 4); Lake Hope St. Park, 20.vi.1993, R.A. Androw, (DAPC, 3); same locality, 11.vii.1995, R.A. Androw, (DAPC, 1); same locality, 20.vi.1993, R.A. Androw, (DAPC, 3).

*Wayne Co*.: Wooster, 3.vi.1939, Amiet, (CMNH, 1).

**OKLAHOMA**

*Grady Co*.: Tuttle, 20.viii.2005, B. Baldwin, Malaise trap, (BBC, 1).

*Latimer Co*.: xii.1982, K. Stephan, (FSCA, 1); iv.1982, K. Stephan, (FSCA, 1); x.2002, K. Stephan, (TAMU, 3).

*Pushmataha Co*.: Clayton, 19.vi.1983, Carrs, lot 2, (DAPC, 1).

**OREGON**

Ulke coll., (CMNH, 2); no other data, (MCZ, 2).

*Douglas Co*.: 7 mi. NW Roseburg, 17.vii.1966, J.D. Vertrees, lite, (AMNH, 1). same locality, 6.viii.1966, J.D. Vertrees, (AMNH, 1).

*Josephine Co*.: Grants Pass, 3.vi.1968, K. J. Goeden, black light trap, (ODAC, 1); same locality, 28.vii.1966, K. J. Goeden, black light trap, (ODAC, 1).

*Klamath Co*.: Klamath R., W. of Keno, 24.iv.1962, Vertrees & Schuh, bark, (AMNH, 1).

*Lane Co*.: Oakridge, 6.vii.1964, S. McCleve, (SMC, 1); same locality, 31.viii.1965, S. McCleve, (SMC, 1).

*Wasco Co*.: The Dalles, 7.viii.1968, black light trap, (ODAC, 1).

**PENNSYLVANIA**

no other data, (INHS, 3), (MCZ, 4).

*Allegheny Co*.: (CMNH, 16); 14.viii.1894, E.A. Klages, (CUIC, 5); Pittsburgh, (CMNH, 5); same locality, 30.v, (CMNH, 2); same locality, vii, (CMNH, 5); same locality, 7.vi, (CMNH, 1); same locality, 20.v, (CMNH, 1); same locality, 9.vii, (CMNH, 1); same locality, 16.v, (CMNH, 2); same locality, 4.vii, (CMNH, 1); same locality, 24.vi, (CMNH, 1); same locality, 27.vi, (CMNH, 2); same locality, 30.v, (CMNH, 1); same locality, 20.vi, (CMNH, 1); same locality, 12.vi, (CMNH, 1); Allegheny [Co.], (MCZ, 2).

*Clearfield Co*.: Bethlehem, 20.v.1901, (FMNH, 3).

*Dauphin Co*.: Harrisburg, 2.ix.1940, V.M. Kirk, on goldenrod, (NDSU, 1).

*Westmoreland Co*.: Jeannette, (CMNH, 1); same locality, 21.vi, (CMNH, 4); same locality, v, (CMNH, 2); same locality, vii, (CMNH, 4); same locality, 22.vi, H.G. Klages, (CMNH, 1); same locality, 5.vii, H.G. Klages, (CMNH, 1); same locality, 23.vi, H.G. Klages, (CMNH, 1); same locality, 1.v, (CMNH, 1); same locality, 3.ix, H.G. Klages, (CMNH, 2); same locality, 13.vii, H.G. Klages, (CMNH, 1); same locality, 21.ix, H.G. Klages, (CMNH, 1); same locality, 2.ix, H.G. Klages, (CMNH, 1); same locality, 23.vi, H.G. Klages, (CMNH, 1); same locality, (CMNH, 9); same locality, (UMRM, 4); same locality, Knechtel, (UMRM, 1); 3 km NE Lower Burrell, 24.v.1987, W. Zanol, (CMNH, 1); 10 km S. Ligonier, Powdermill Biological Reserve, ca 400 m., 17.vi.1996, coll. on dead logs at night, D.A. Pollock, (DAPC, 1).

*York Co*.: 5 mi. NW Davidsburg, 8–11.v.1970, P. & P. Spangler, (USNM, 1).

**SOUTH CAROLINA**

no other data, (MCZ, 1).

*Abbeville Co*.: Antreville, 28.iv.1973, L.L. Lampert, under bark, (FSCA, 5).

*Beaufort Co*.: Parris Island, 2–9.iv.1981, J.F. Reinert, (FSCA, 2); same locality, 2.vii.1981, J.F. Reinert, (FSCA, 1).

*Florence Co*.: Florence, 30.iii.1954, V.M. Kirk, (NDSU, 1); same locality, 24.iv.1964, V.M. Kirk, (NDSU, 1); same locality, 30.vii.1956, V.M. Kirk, (NDSU, 1); same locality, 13.xi.1952, V.M. Kirk, under dead bark, (NDSU, 1); same locality, 23.x.1961, V.M. Kirk, under dead bark, (NDSU, 1); same locality, 18.vi.1962, V.M. Kirk, tanglefoot screen, (NDSU, 1); same locality, 6.vi.1954, V.M. Kirk, (NDSU, 1); same locality, 15.v.1957, V.M. Kirk, fungus, (NDSU, 1).

*Lancaster Co*.: Union, 6.iii.1961, (USNM, 1).

*Pickens Co*.: Clemson, 30.iv.1975, J.R. Ables, (TAMU, 1).

**SOUTH DAKOTA**

*Minnehaha Co*.: Sioux Falls, 4.vi.1966, V.M. Kirk, under stone, (NDSU, 1).

*Yankton Co*.: Yankton, 13.vii.1965, V.M. Kirk, fungus, (NDSU, 1).

**TEXAS**

*Anderson Co*.: Salmon, 1–12.x.1975, H.R. Burke, malaise trap, (TAMU, 1); same locality, 17.v-6.vi.1975, H.R. Burke, malaise trap, (TAMU, 1); Gus Engeling Wildlife Management Area, 4.v.2002, S.M. Clark & D.J. Cavan, (BYUC, 1).

*Angelina Co*.: Angelina Nat’l. Forest ca. 5.5 mi. SE Zavalla, 31°07'05"N, 94°20'01"W 5–18.ix.1996, Clarke, Menard & Riley, loblolly pine, managed; funnel trap, sample 244, (TAMU, 1).

*Briscoe Co*.: Quitaque, 21.iv.1987, Carrs, lot 1, (CARR, 1).

*Cass Co*.: Daingerfield St. Pk., 27.v.1977, Riley & LeDoux, (EGRC, 1).

*Cherokee Co*.: 2.iv.1939, R.E. Maxwell, (INHS, 1).

*Montgomery Co*.: Sam Houston Nat. Forest, 26.x.1963, J.C. Schaeffer, (TAMU, 1); The Woodlands, 20–26.vi.1978, J.E. Wappes, (TAMU, 1); same locality, 5–7.iv.1978, J.E. Wappes, (TAMU, 1).

*Polk Co*.: Moscow, 2.iv.1980, N.M. Downie, (FMNH, 2).

*Sabine Co*.: 9 mi. E. Hemphill, “Beech Bottom”, 6–16.viii.1989, R. Anderson & E. Morris, Malaise trap in beech-magnolia forest, (TAMU, 1); same locality, 8–21.v.1989, R. Anderson & E. Morris, Malaise trap in beech-magnolia forest, (TAMU, 1); same locality, 24.ix-7.x.1989, R. Anderson & E. Morris, Malaise trap in beech-magnolia forest, (TAMU, 5); same locality, 10–23.ix.1989, R. Anderson & E. Morris, Malaise trap in beech-magnolia forest, (TAMU, 1); same locality, 7–26.x.1989, R. Anderson & E. Morris, Malaise trap in beech-magnolia forest, (TAMU, 1); same locality, 5–17.vi.1989, R. Anderson & E. Morris, Malaise trap in beech-magnolia forest, (TAMU, 1); same locality, 13–24.vii.1989, R. Anderson & E. Morris, Malaise trap in beech-magnolia forest, (TAMU, 1); same locality, 13–18.iii.1989, R. Anderson & E. Morris, Malaise trap in beech-magnolia forest, (TAMU, 2).

*San Jacinto Co*.: Big Creek Scenic Area, 8.iii.1988, P.W. Kovarik, (TAMU, 3).

*Travis Co*.: Austin, BFL, 170 m, 30 16 55N 97 46 45W, C.R. Nelson, Malaise trap, 16–23.x.1990, (BYUC, 2); same locality and collector, 22–28.v.1990, (BYUC, 3); 15–21.v.1990, (BYUC, 2); 13–19.iii.1990, (BYUC, 1); 26–30.iv.1990, (BYUC, 1); 16–25.iv.1990, (BYUC, 1).

*Tyler Co*.: Kirby State Forest, 30°34'30"N, 94°25'03"W, 9–30.iii.2003, E. Riley, elevated flight intercept (TAMU, 1).

*Walker Co*.: Cobb Creek, Hwy 207, 10 mi. E. of Huntsville, 20.iv.1990, R.W. Baumann, (BYUC, 1).

*Counties unknown*: Hiwy 37 atRedR, 9.iv.1971, R&E Froeschner, (USNM, 1).

**UTAH**

*Washington Co*.: Schwits Indian Reservation, 20.viii.1920, V.M. Tanner, (BYUC, 1).

**VIRGINIA**

no other data, (MCZ, 1).

*Chesterfield Co*.: Pocahontas St. Pk., 29.vi.1992, S. Laplante, at uv light, (SLC, 1).

*Clarke Co*.: U. Va. Blandy Exp. Farm, 2 mi S Boyce 3905N 7810W, 6–20.viii.1993, D.R. Smith, Malaise trap, (USNM, 1); same locality, 11–25.v.1993, D.R. Smith, Malaise trap, (USNM, 3).

*Essex Co*.: 1 mi. SE Dunnsville 3752N 7648W, 4.viii-2.ix.1993, D.R. Smith, Malaise trap, (USNM, 1); same locality, 23.ix-19.x.1993, D.R. Smith, Malaise trap, (USNM, 1).

*Fairfax Co*.: near Annandale, 27.v.1981, David R. Smith, Malaise trap, (USNM, 1).

*Falls Church Co*.: Falls Church, (MCZ, 1).

*Lee Co*.: Stone Creek, (MCZ, 2).

*Loudoun Co*.: Purcellville, 4.vi.1979, E.J. Hambleton, (OSUC, 1).

**VERMONT**

*Rutland Co*.: Hydeville, 13.vii.1970, Parsons, ex. *Polyporus schweinitzii*, (MCZ, 1).

**WASHINGTON**

*Whatcom Co*.: Baker Lake Area 28 km N Concrete, 2–3.vii.1988

**WEST VIRGINIA**

*Braxton Co*.: 1 mi. S. Flatwoods, 26.v-1.vi.1995, M.D. Bombardiere, Lindgren funnel trap, (PPCD, 1).

*Greenbrier Co*.: White Sulphur, A. Fenyes, (MCZ, 1).

*Kanawha Co*.: Guthrie, 7.vii.1990, S.M. Clark, (PPCD, 1).

*Lewis Co*.: Jane Lew, 13–21.vi.1994, M.D. Bombardiere, Lindgren funnel trap, (PPCD, 1).

*Mineral Co*.: Hartmansville, 14–21.vi.1994, W.L. Drosselmeyer, Lindgren funnel trap, (PPCD, 1).

*Nicholas Co*.: Richwood, 4–5.viii.1992, S.F. Hutchinson, Lindgren funnel trap, Georgia Pacific lumber yard, (DAPC, 1).

*Randolph Co*.: Dailey, Coastal Lumber Yard, 20–21.vii.1992, S.F. Hutchinson, Lindgren funnel trap, (BYUC, 1).

**WISCONSIN**

*Bayfield Co*.: Bayfield, Wickham, (MCZ, 1).

*Dane Co*.: 27.v.1900, (UMRM, 2); 26.x.1900, W.S. Marshall, (WIRC, 1); 22.v.1899, (WIRC, 1); Missouri Rd., DNR woods, Deerfield, 17.vii.1988, B. Buchli, blacklight trap, (WIRC, 1); Nelson Rd., Lee Woods, Deerfield, 9.iv.1988, B. Buchli, rotting log, (WIRC, 1).

*Grant Co*.: T6N, R6W S17, 12–19.vii.1976, gypsy moth M.T., (WIRC, 1); same locality, 26.vii-9.viii.1976, gypsy moth M.T., (WIRC, 1); same locality, 7–14.vi.1976, gypsy moth M.T., (WIRC, 2); same locality, 22–29.vi.1977, gypsy moth M.T., (WIRC, 1).

*Iowa Co*.: T6N, R5E, S1, 7–14.vi.1976, gypsy moth M.T., (WIRC, 1).

*Oconto Co*.: Mountain, 20.vi.1935, (FMNH, 3).

*Racine Co*.: 7.ix.1953, D.H. Habeck, (FSCA, 1); 13.viii.1953, D.H. Habeck, (FSCA, 1).

*Vilas Co*.: Sayner, 18.ix.1926, (FMNH, 1).

**WYOMING**

*Counties unknown*: 5 mi. west of Custer, hwy 16, 12.ix.1973, L. Bezark, M. Ebertz, C. Kitayama, under bark of pine, (UCBC, 1).
